# Prevalence of chronic pain or analgesic use in children and young people and its long-term impact on substance misuse, mental illness, and prescription opioid use: a retrospective longitudinal cohort study

**DOI:** 10.1016/j.lanepe.2023.100763

**Published:** 2023-11-15

**Authors:** Andrew Lambarth, Michail Katsoulis, Chengsheng Ju, Alasdair Warwick, Rohan Takhar, Caroline Dale, David Prieto-Merino, Andrew Morris, Debajit Sen, Li Wei, Reecha Sofat

**Affiliations:** a Department of Clinical Pharmacology and Therapeutics, St George's University of London, London, UK; b St George's University Hospitals NHS Foundation Trust, Cranmer Terrace, London, UK; c MRC Unit for Lifelong Health and Ageing, Institute of Cardiovascular Science, University College London, London, UK; d Research Department of Practice and Policy, University College London School of Pharmacy, 29-39 Brunswick Square, London, WC1N 1AX, UK; e Institute of Cardiovascular Science, University College London, London, UK; f Moorfields Eye Hospital NHS Foundation Trust, London, UK; g Department of Pharmacology and Therapeutics, University of Liverpool, Liverpool, UK; h Facultad de Medicina, Universidad de Alcalá, Madrid, Spain; i Usher Institute, College of Medicine and Veterinary Medicine, The University of Edinburgh, Nine Edinburgh BioQuarter, 9 Little France Road, Edinburgh, EH16 4UX, UK; j Health Data Research UK, 215 Euston Road, London, NW1 2BE, UK; k University College London Hospitals NHS Foundation Trust, 235 Euston Rd, London, NW1 2BU, UK

**Keywords:** Chronic pain, Paediatric and adolescent health, Mental health, Substance misuse, Prescription opioids, Analgesic medicines

## Abstract

**Background:**

Epidemiological studies suggest chronic and recurrent pain affects around a quarter of children, while 8% report intense and frequent pain. The long-term implications of chronic pain in childhood are uncertain. Using electronic health records (EHRs) we used both disease codes and medicines prescription records to investigate the scale of chronic pain and long-term analgesic use in children and young people (CYP), and if chronic pain and/or use of analgesic medicines at an early age is associated with substance misuse, use of prescription opioids, and poor mental health in adulthood.

**Methods:**

We conducted a cohort study using data from IQVIA Medical Research Data UK. We identified individuals aged 2–24 with exposure to either a diagnostic code indicating chronic pain (diagnosis-exposed), repeat prescription for medicines commonly used to treat pain (prescription-exposed), or both. Follow-up began at 25, and the unexposed population acted as comparators. We calculated hazard ratios (HR) for mental health and substance misuse outcomes, and rate ratios (RR) for opioid prescriptions in adulthood. Additionally, we investigated which diagnoses, if any, were over-represented in the prescription-exposed subgroup.

**Findings:**

The cohort constituted 853,625 individuals; 146,431 had one or more of the exposures of interest (diagnosis-exposed = 115,101, prescription-exposed = 20,298, both-exposed = 11,032), leaving 707,194 as comparators. Median age at index exposure was 18.7 years (IQR 14.7–22.3). On average during follow-up, the pooled exposed group had, respectively, a 31% and 17% higher risk of adverse mental health and substance misuse outcomes (adjusted HR [95% CI] of 1.31 [1.29–1.32] and 1.17 [1.11–1.24]). Exposed individuals also received prescription opioids at double the rate of unexposed individuals on average during follow-up (adjusted RR 2.01 [95% CI 1.95–2.10]). Outcomes varied between exposure subgroups, with prescription- and both-exposure tending to have worse outcomes. Unlike these two subgroups, in the diagnosis-exposed subgroup we did not detect a greater risk of substance misuse.

**Interpretation:**

Chronic pain in CYP is associated with increased prescription opioid use and adverse mental health outcomes in adulthood, as is repeat prescription for analgesic medicines, but only the latter is also associated with substance misuse in adulthood. It is essential to avoid the harms of under-treating pain in CYP while giving due consideration to the risks posed by analgesic medicines. Early recognition of chronic pain in CYP and utilising non-pharmacological management options may help minimise overprescribing, and long-term reliance on dependence-forming-drugs.

**Funding:**

AL is an NIHR funded academic clinical fellow, and was supported by funding from 10.13039/501100008721UCLH Charities while carrying out this work. RS and DS are part of the Advanced Pain Discovery Platform and were supported by a UKRI and Versus Arthritis grant (MR/W002566/1) as part of the Consortium Against Pain Inequality. AW was supported by the 10.13039/100010269Wellcome Trust (220558/Z/20/Z).

**Research in context**

**Evidence before this study**

We searched PubMed without date or language restriction up to 5 October 2023. We used the terms ((“chronic pain”) OR (analgesi∗ OR painkiller OR opioid)) AND ((“child∗” OR “youth” OR “adolescen∗”) AND (“mental illness” OR “mental health” OR “substance misuse” OR “substance use disorder”)), looking for matches in article titles and abstracts. We screened the resulting 658 records to identify eligible original research articles which investigated adverse outcomes associated with chronic pain or analgesic exposure in children and young people. Evidence to date suggests experiencing pain as a child or young person (CYP) is associated with a number of adverse physical, psychological, and social outcomes in adulthood, including opioid misuse and mental illness. Medicines that are used to treat pain may themselves contribute to harm in many ways including, but not limited to, adverse drug reactions and subsequent drug dependence; exposures and harms due to opioids are among the most commonly studied. Despite evidence that both pain and the medicines used to treat it may be associated with similar outcomes, we identified a paucity of research that investigates their interplay and relative impact.

**Added value of this study**

We identified cases of chronic pain and exposure to repeat prescriptions for analgesic medicines within a large, nationally-representative database of coded primary care records. We found that exposure as a CYP to chronic pain, and/or a repeat prescription for a medicine used to treat pain, is associated with a higher risk of mental illness (a composite outcome including anxiety, depression, bipolar affective disorder, schizophrenia, referral to mental health services, eating disorders, self-harm or suicide, and personality disorders) and rate of prescription opioid use in adulthood. Importantly, while there was an increased risk of substance misuse (a composite outcome including misuse of, dependence on, or withdrawal from various specified or unspecified substances) within subgroups exposed to a repeat analgesic prescription, this was not the case among individuals exposed to a chronic pain diagnostic code alone. This suggests that, specifically, receipt of a repeat prescription for an analgesic medicine before the age of 24 is associated with substance misuse in adulthood. In a stratified analysis we found that even exposure to a repeat prescription for analgesic medicines which are not opioids, or thought to be dependence-forming, may be associated with increased prescription opioid use, and adverse substance misuse and mental health outcomes in later life.

We additionally demonstrated that individuals with intellectual disability and autistic spectrum disorder were over-represented among participants receiving repeat prescriptions for analgesic medicines in the absence of a diagnostic code for chronic pain which, if validated, may indicate overprescribing in this already vulnerable group.

**Implications of all the available evidence**

Young people with chronic pain are at risk of life-long adverse outcomes, and the way pain is managed while childhood and adolescent neurodevelopment is ongoing may have important implications for these outcomes. While uncontrolled pain may cause long-term harm, over-reliance on analgesic medicines from a young age may also predispose to life-long adversity. Clinicians should be aware of and aim to appropriately use specialist pain management services for CYP. They should also seek to recognise and avoid the risks of overprescribing analgesic medicines, both for those with pain, and also those with intellectual disability and/or autistic spectrum disorder.

## Introduction

Chronic pain is defined as pain that lasts more than three months. Whilst well recognised in adults, it is also prevalent in paediatric populations with some estimates as high as 30%.^1^^,^^2^ The World Health Organization (WHO) describe chronic pain in children as a ‘significant public health concern globally’,^3^ and in their 2021 Lancet commission on paediatric pain, Eccleston et al. set out four key transformational goals, one of which is to ‘make pain understood’.^4^ Therein, they recognise the paucity of research into the relationships between childhood chronic pain and ‘later biological development and health’. Extant literature in this field suggest exposure to chronic pain in early life is associated with a number of clinically and socially important adverse outcomes in adulthood including, but not limited to: opioid misuse,^5^ lower educational attainment,^6^ mental health diagnosis,^7^^,^^8^ and receipt of welfare benefits.^9^ It is also associated with a higher risk of pain in adulthood.^10^, ^11^, ^12^

Medicines used to relieve pain may also pose risks, and other studies have employed various methods to investigate associations between prescription opioid exposure in early life and subsequent substance misuse. Meich et al. found that legitimate prescription opioid use prior to high school graduation was independently associated with non-medical opioid use between ages 19 and 23.^13^ In contrast, McCabe et al. found that while, overall, prescription opioid exposure in adolescence was associated with non-medical prescription opioid use in later life, there was no detectable association between exclusively medical use of opioids in adolescence and symptoms of substance use disorder by the age of 35.^14^ Similarly, Quinn et al. found that prescription opioid exposure in adolescence or young adulthood was associated with an increased absolute risk of substance-associated morbidity within five years of first exposure.^15^ Importantly this latter study compared prescription opioid initiation with non-steroidal anti-inflammatory drug initiation to attempt to control for confounding by indication (i.e. pain). While both pain and the medicines used to treat it may be associated with adverse outcomes, to our knowledge no study has previously investigated health outcomes associated with both chronic pain and analgesic exposures simultaneously within a CYP population.

We therefore investigated how common chronic analgesic medicine use may be in children and young people (CYP), and if this was associated with, or independent of, an underlying chronic pain condition. We also sought to investigate if exposure to chronic pain and/or medications used to treat pain were associated with chronic use of prescription opioid medicines over a life-course, or adverse outcomes of substance misuse and poor mental health.

## Methods

### Data sources and population at risk

We performed a retrospective cohort study using electronic primary healthcare data from IQVIA Medical Research Data (IMRD) that incorporates data supplied by The Health Improvement Network (THIN), a propriety database of Cegedim SA. THIN is a database consisting of longitudinally-linked, de-identified health-records from over 500 participating primary care practices in the UK. The database contains records for approximately eleven-million individuals, and has been shown to be representative of the wider UK population.^16^

We selected a cohort including all individuals within the THIN database who turned 25 in or after 2001, and had a minimum of one year each of exposure window (between registration at a participating practice and 25th birthday) and follow-up time (between 25th birthday and the first of: transfer out practice, last data collection, or death).

### Delineation of exposure groups

We defined exposures as presence of either/both:

a) A diagnostic code considered strongly suggestive of chronic pain (diagnosis-exposure); this included any code explicitly describing pain as ‘intractable’, ‘persistent’, or ‘chronic’, as well as codes for neuropathic pain, dysmenorrhoea, engagement with specialist pain services, and any condition categorized as ‘chronic primary pain’ within ICD-11 (e.g. fibromyalgia, migraine)
b) A repeat prescription—i.e. a prescription order allowing a medicine to be dispensed at least twice, at stipulated intervals, without the need to consult the prescriber each time—for medicines usually used to treat pain (prescription-exposure)

Full details of diagnoses and medicines eligible as exposures are provided in the Supplementary Materials, Table S1.

Participants entered the exposed group when they had an exposure event while aged between 0 and 24 years (i.e. CYP). The unexposed cohort served as the comparator group, and follow-up began on the 25th birthday for all exposed and unexposed participants.

### Outcome definitions

We defined three clinical outcomes of interest in adulthood: mental illness (a composite outcome including codes for: anxiety, depression, bipolar affective disorder, schizophrenia, referral to mental health services, eating disorders, self-harm or suicide, and personality disorders), substance misuse (a composite outcome including codes indicating misuse of, dependence on, or withdrawal from various specified or unspecified substances), and prescription opioid use. Full code lists and descriptions of their implementation are provided in the Supplementary Material.

### Outcome analysis

For adverse mental health and substance misuse outcomes, we identified the first occurrence of a code implying a new or present outcome event, and used survival analysis to compare exposed and unexposed groups. We repeated each analysis with the exposed group stratified by exposure type, namely: prescription-exposed, diagnosis-exposed, and both-exposed. For each outcome, we plotted Kaplan–Meier survival curves, and used Cox proportional hazards (PH) models to calculate hazard ratios (HR), and then to adjust for baseline covariates.

We calculated adjusted rate ratios (RR) for opioid prescriptions during follow-up, excluding continuations of repeat prescription sequences that were initiated before the 25th birthday. We tested several different count models and present the model best suited to our data, namely the negative binomial.

Participants were considered to be at risk for outcome events from the 25th birthday onwards; this was the origin and the start of follow-up for the survival analysis. For Cox models, end time was either first occurrence of a relevant outcome event, death, transfer out of a participating practice, or end of database follow-up which would equate to the last data collection date. For recurrent events survival models, the end time was the first of either death, transfer out of a participating practice, or end of database follow-up (there was no terminal event). For our adjusted analyses we included the following baseline covariates: gender, deprivation, smoking status, alcohol use, body mass index, year of birth, prior mental illness, and prior substance misuse.

### Conditions disproportionately represented within the prescription-only subgroup

We explored conditions which were over- and under-represented within the prescription-exposure subgroup relative to the rest of the exposed group (i.e. the combined diagnosis-exposed and both-exposed groups) before the age of 25. First, we identified diagnoses using primary care code lists for phenotypes curated by Kuan et al.^17^; including only those which used Read V2 codes, this constituted 276 phenotypes, of which 263 remained after excluding any that shared codes with our list for chronic pain exposure (irritable bowel syndrome; stroke, not otherwise specified; disorders of autonomic nervous system; non-acute cystitis; post-viral fatigue syndrome, neurasthenia and fibromyalgia; diabetic neurological complications; dysmenorrhoea; enthesopathies & synovial disorders; intervertebral disc disorders; migraine; peripheral neuropathies; peripheral arterial disease; trigeminal neuralgia). Next, we calculated the period prevalence of these conditions within the prescription-only-exposed subgroup, and also in the remainder of the exposed group (a combination of diagnosis- and both-exposed subgroups). We calculated period prevalence odds ratios, 99% confidence intervals, and two-sided p-values using Fisher's exact test with mid-P correction. We used the Bonferroni method to correct significance estimates for multiple comparisons, and considered conditions which had a statistically significantly (p < 0.01 after multiple comparisons correction) higher or lower period prevalence within the prescription-exposed group to be, respectively, over- and under-represented within the subgroup.

### Model assumptions and sensitivity analyses

Model assumptions were tested using visual and statistical methods, including the linearity assumption for quantitative covariables, and the PH assumption for Cox models. We assessed the linearity of quantitative covariate using graphical and statistical methods, as well as testing the sensitivity of our models to the use of non-linear modelling methods. Details are provided in the Supplementary Materials, Section 8. Covariates which did not predict outcomes in a linear manner were modelled using restricted cubic splines with four knots, and automated algorithmic specification of knot position as implemented in the ‘rms’ R package.^18^ Where assessment of the slope of Schoenfeld residuals suggested violation of the PH assumption, assuming log–log survival plots did not suggest a severe violation, we elected to present these models with the interpretation of hazard estimates as ‘average hazard over follow-up’. We further assessed robustness of this interpretation in two ways: first by using inverse probability of treatment weighting to estimate adjusted non-parametric cumulative incidence curves with 10-year absolute risk differences, and second by stratification of covariates and exclusion of incomplete cases which resulted in models which satisfied the PH assumption.

To guide selection of appropriate count models we tested for overdispersion in our data using likelihood ratios as implemented in the odTest function within the ‘pcsl’ R package,^19^ and also tested for zero inflation. As a contrasting approach to count models, we also present the Cox-type recurrent events survival model described by Lin, Wei, Yang and Yin (LWYY) which uses recurrent time-to-event data to estimate rate ratios, and does not require a terminal event.^20^

For each outcome investigated, we undertook sensitivity analyses to assess the robustness of our findings to different models and missing data assumptions, and the headline results of these are presented in the Supplementary Materials, Section 6. Model selection was based on a combination of quantitative measures such as concordance index, satisfaction of model assumptions, and model interpretability.

We also performed sensitivity analyses sequentially excluding anti-neuropathic and over-the-counter medicines from the list of exposure-defining analgesics, and excluding all exposures occurring before the age of 2 (i.e. in infancy) and before the age of 10 (i.e. allowing only exposures in ‘young people’). Guided by the results of our investigation of disproportionately represented conditions, we also added several covariables to our adjusted models to assess whether between-subgroup differences could be explained by said disproportionate representation. These additional covariables were: prior diagnosis of cerebral palsy, epilepsy, autistic spectrum disorder, intellectual disability, and malignancy (all present/absent).

All analyses were performed in R, version 4.1.2.^21^ For Kaplan–Meier plots and Cox models we used the ‘survival’ and ‘survminer’ packages, and for LWYY recurrent events analysis we used the ‘reReg’ package.^22^, ^23^, ^24^

### Ethical considerations

Our protocol received approval from an independent Scientific Review Committee (reference number 20SRC074), and the NHS Health Research Authority Research Ethics Committee was informed.

### Role of the funding source

There was no specific funding for the conduct of this study. No funders had any involvement in the study design, the collection, analysis, and interpretation of data, the writing of the report, nor the decision to submit the paper for publication.

## Results

### Population characteristics

There were 853,625 individuals in the cohort. 146,431 (16.3%) individuals were included in the pooled-exposed group (diagnosis-exposed = 115,101, prescription-exposed = 20,298, both-exposed = 11,032), leaving 707,194 in the unexposed group [Table 1]. The median and interquartile range (IQR) of the age in the exposed group at index-exposure was 18.7 years (IQR 14.7–22.3). Median ages and interquartile range at index exposure for each subgroup were as follows: diagnosis-exposed, 18.8 (15.1–22.3); prescription-exposed, 19.2 (12.3–22.8); and both-exposed 16.6 (12.8–20.7). Two thirds of individuals with a diagnostic code suggestive of chronic pain were female; more specifically 75% for diagnosis-exposed, and 76% for both-exposed subgroups. However, those in the prescription-exposed subgroup were approximately gender-balanced. Exposed individuals tended to be born later than those in the unexposed group, which led us to incorporate year of birth in our adjusted models. Other factors including deprivation (using the Townsend quintile), smoking status, alcohol intake, and body mass index prior to index, whilst more balanced across subgroups, still varied significantly and were included as covariates in adjusted models.

**Table 1:** Summary of population characteristics.

Characteristic	Non-exposed N = 707,194^a^	Diagnosis-exposed N = 115,101^a^	Prescription-exposed N = 20,298^a^	Both-exposed N = 11,032^a^
Female gender	302,236 (43)	86,098 (75)	9385 (46)	8358 (76)
Townsend quintile				
1	41,033 (5.8)	8636 (7.5)	1236 (6.1)	806 (7.3)
2	38,904 (5.5)	7850 (6.8)	1394 (6.9)	863 (7.8)
3	44,631 (6.3)	9073 (7.9)	1797 (8.9)	1120 (10)
4	45,642 (6.5)	8969 (7.8)	1843 (9.1)	1186 (11)
5	37,466 (5.3)	7280 (6.3)	2169 (11)	1142 (10)
Unknown	499,518 (71)	73,293 (64)	11,859 (58)	5915 (54)
Smoking status				
Non-Smoker	325,281 (46)	55,109 (48)	10,086 (50)	5371 (49)
Ex-Smoker	47,673 (6.7)	14,283 (12)	1996 (9.8)	1555 (14)
Smoker	139,473 (20)	26,380 (23)	5036 (25)	2826 (26)
Unknown	194,767 (28)	19,329 (17)	3180 (16)	1280 (12)
Drinking status				
Non-Drinker	88,295 (12)	15,693 (14)	3222 (16)	1932 (18)
Ex-Drinker	4383 (0.6)	1222 (1.1)	245 (1.2)	219 (2.0)
Drinker	309,962 (44)	61,334 (53)	8220 (40)	5329 (48)
Unknown	304,554 (43)	36,852 (32)	8611 (42)	3552 (32)
Body mass index				
Ideal	245,455 (35)	46,644 (41)	6628 (33)	3938 (36)
Underweight	25,684 (3.6)	5065 (4.4)	822 (4.0)	525 (4.8)
Overweight	92,402 (13)	19,870 (17)	3187 (16)	2087 (19)
Obese	57,192 (8.1)	15,858 (14)	2832 (14)	2267 (21)
Unknown	286,461 (41)	27,664 (24)	6829 (34)	2215 (20)
Year of birth				
1976–1979	168,806 (24)	21,014 (18)	2712 (13)	1211 (11)
1980–1984	226,768 (32)	35,793 (31)	5564 (27)	2973 (27)
1985–1989	206,899 (29)	38,141 (33)	6800 (34)	4033 (37)
1990–1994	104,721 (15)	20,153 (18)	5222 (26)	2815 (26)
Prior substance misuse	13,240 (1.9)	2134 (1.9)	756 (3.7)	292 (2.6)
Prior mental illness	119,559 (17)	40,348 (35)	5561 (27)	4520 (41)

a n (%).

### Model selection and specification

BMI did not predict outcomes in a linear manner, so this was modelled non-linearly using restricted cubic splines. Missing BMI data necessitated exclusion of 323,169 (37.9%) individuals for the estimation of adjusted models. Year of birth appeared to satisfy linearity assumptions, and so was incorporated into the model as such, with values from 1 to 19 replacing 1976 to 1994. In our main Cox analyses for substance misuse and mental health outcomes the PH assumption was violated (global p-value <0.0001), and as such the interpretation of hazard ratios presented for these analyses should be as weighted averages of hazard ratios over the entirety of follow-up.^25^ The direction and statistical significance of these associations were robust to a complete cases sensitivity analysis which employed covariate stratification and did not violate the PH assumption (Supplementary Materials, Section 6). We identified marked overdispersion in our opioid prescription count data (p < 0.0001 for likelihood-ratio test to assess overdispersion). We therefore chose to use models which allow for overdispersion. There was also evidence of zero-inflation, and exclusion of zero values did not negate overdispersion. While the NB model appeared to accurately fit excess zeros (ratio of predicted vs actual zeros 1.00:1.00), we found the quasi-Poisson model underfitted zeros (ratio of predicted to observed zeros 0.69:1.00). Because the NB accurately fitted zeros we did not modify it to a zero-inflated model. The LWYY model relies on a different data structure so cannot be directly compared to count models. Detailed results of both analyses are presented in Supplementary Material Section 4.

### Survival analysis

The median follow-up time in the population at risk was 5.1 years (IQR 2.6–9.0). During follow-up, in total 11,644 individuals were identified as having a substance misuse event, and 143,838 individuals with a mental health outcome event; rates are summarized in Table 2. For the mental health outcome analysis, the total number right-censored was 709,787.1258 died, 277,425 transferred out of a participating practice, and 431,104 reached the end of database follow-up before experiencing an event of interest. For the substance misuse outcome analysis, 841,981 individuals were right-censored: 1732 died, 311,079 transferred out of a participating practice, and 529,170 reached the end of database follow-up.

**Table 2:** Event rates and incidence rate ratios for substance misuse and mental illness outcomes.

Outcome	Group	Number of events	Total follow-up time (years)	Event rate per 1000 PYs	Incidence rate ratio (95% CI)
Mental illness	Unexposed	105,978	3,881,872	27.3	1 (Ref)
	Diagnosis	30,162	541,690	55.7	2.04 (2.01–2.07)
	Prescription	4404	96,731	45.5	1.67 (1.62–1.72)
	Both	3294	44,871	73.4	2.69 (2.60–2.78)
	*Pooled exposed*	37,860	683,293	55.4	2.03 (2.01–2.05)
Substance misuse	Unexposed	9451	4,361,868	2.2	1 (Ref)
	Diagnosis	1392	684,068	2.0	0.94 (0.89–0.99)
	Prescription	572	113,999	5.0	2.32 (2.13–2.52)
	Both	229	58,171	3.9	1.82 (1.59–2.07)
	*Pooled exposed*	2193	856,237	2.6	1.18 (1.13–1.24)

PYs = person-years.

#### Substance misuse

**Fig. 1:**
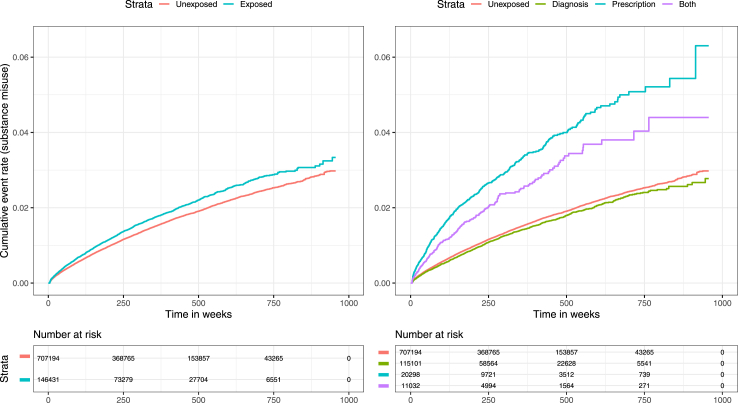
Kaplan–Meier curves showing cumulative substance misuse event rates within the pooled exposed and unexposed groups (left), and with the exposed group stratified by exposure type (right).

**Fig. 2:**
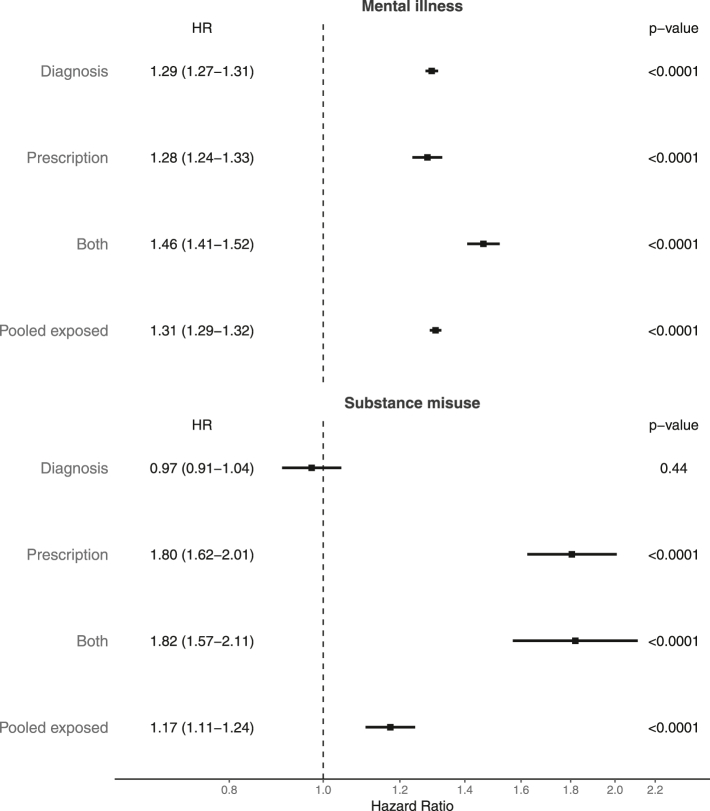
A forest plot summarizing the adjusted Cox proportional hazard ratios for substance misuse and mental health outcomes.

Pooled exposure (chronic pain and/or receipt of analgesic repeat prescription) was associated with substance misuse with a HR of 1.16 (95% CI 1.11–1.22) using an unadjusted PH model. With adjustment the HR marginally increased to 1.17 (95% CI 1.11–1.24). Stratifying by exposure subgroup, prescription-exposed and both-exposed subgroups had higher hazards vs the unexposed (1.81 [95% CI 1.62–2.01] and 1.82 [95% CI 1.57–2.11], respectively), however, we did not detect a greater hazard in the diagnosis-exposed subgroup (HR 0.97 [95% CI 0.91–1.04]). Kaplan–Meier curves demonstrating cumulative incidence of substance misuse are shown in Fig. 1, and a forest plot illustrating hazard ratios are shown in Fig. 2.

We further stratified diagnostic exposures by phenotype or group of phenotypes, and prescription exposures by class of analgesic (Supplementary Material, Table S11). In this approach, some phenotypes were associated with a higher adjusted hazard ratio for substance misuse, namely: chronic regional pain syndrome and/or erythromelalgia (HR 5.43 [95% CI 2.39–12.33]), and unspecified chronic pain (HR 1.37 [95% CI 1.00–1.88]). Weak opioids were the analgesic class associated with the greatest hazard (HR 2.34 [95% CI 1.98–2.76]), and exposure to a repeat prescription for paracetamol or an antidepressant also carried a higher hazard for substance misuse. However, correction of p-values for 22 comparisons suggested only exposures to weak opioids, paracetamol, and chronic regional pain syndrome and/or erythromelalgia carried a statistically significant greater hazard.

#### Mental illness

**Fig. 3:**
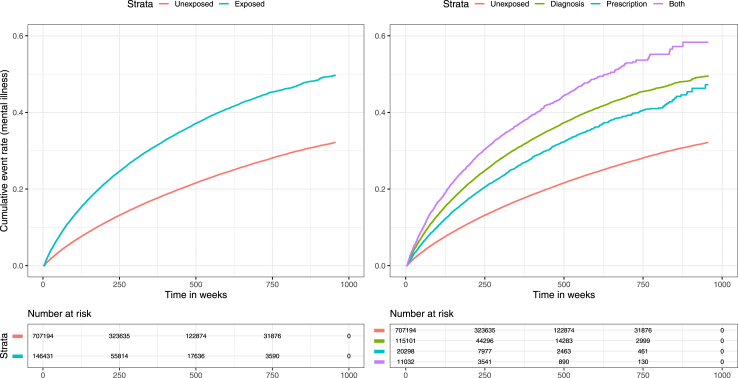
Kaplan–Meier curves showing cumulative mental illness event rates within the pooled exposed and unexposed groups (left), and with the exposed group stratified by exposure type (right).

Pooled exposure was associated with mental health outcomes with a HR of 1.97 (95% CI 1.94–1.99), attenuating to a HR of 1.31 (95% CI 1.29–1.32) after adjusting for covariates. When stratified by exposure subgroup, the highest adjusted HR was in the both-exposed group at 1.46 (95% CI 1.41–1.52); however all subgroups demonstrated evidence of an increased hazard for mental health outcomes. Kaplan–Meier curves demonstrating survival to first mental health event are shown in Fig. 3, and a forest plot illustrating hazard ratios is shown in Fig. 2.

#### Long-term prescription opioid usage

**Fig. 4:**
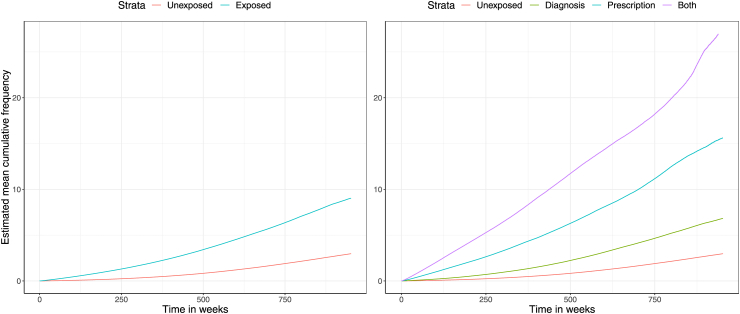
Mean cumulative frequency of opioid prescriptions within the pooled exposed and unexposed groups (left) and with the exposed group stratified by exposure type (right).

77,337 (9.1%) individuals in the cohort received at least one opioid prescription during follow-up, and there were 688,947 opioid prescription events in total. Unadjusted incidence rates for opioid prescription events were: 88 (unexposed), 223 (diagnosis-exposed), 639 (prescription-exposed), and 1169 (both-exposed) events per 1000 person-years (PYs). Adjustment for covariates using negative binomial and LWYY models attenuated but did not negate the substantially higher estimated rates within each of the exposure subgroups. The diagnosis-exposed subgroup had the lowest RR of the three exposure subgroups, with the NB model providing an estimate of 2.01 (95% CI 1.95–2.10) and the LWYY providing a more conservative estimate of 1.80 (95% CI 1.68–1.92). The both-exposed group had the highest RR estimates of 13.85 (95% CI 12.62–15.20) and 8.52 (7.76–9.36) with the NB and LWYY models, respectively. Mean cumulative event frequencies are shown in Fig. 4, and adjusted rate ratios are presented in Table 3. Further details are provided in the Supplementary Material, Table S12.

**Table 3:** Event rates for opioid prescriptions after the age of 24.

Stratum	Rate ratio; negative binomial model (95% CI)	p-value; negative binomial model	Rate ratio; LWYY model (95% CI)	p-value; LWYY model
Unexposed	1 (Ref)	NA	1 (Ref)	NA
Diagnosis	2.02 (1.95–2.10)	<0.0001	1.80 (1.68–1.92)	<0.0001
Prescription	8.16 (7.56–8.81)	<0.0001	5.90 (5.33–6.54)	<0.0001
Both	13.85 (12.62–15.20)	<0.0001	8.52 (7.76–9.36)	<0.0001
*Pooled exposed*	3.66 (3.55–3.78)	<0.0001	2.80 (2.65–2.97)	<0.0001

### Condition prevalence in the prescription-only exposure subgroup

**Fig. 5:**
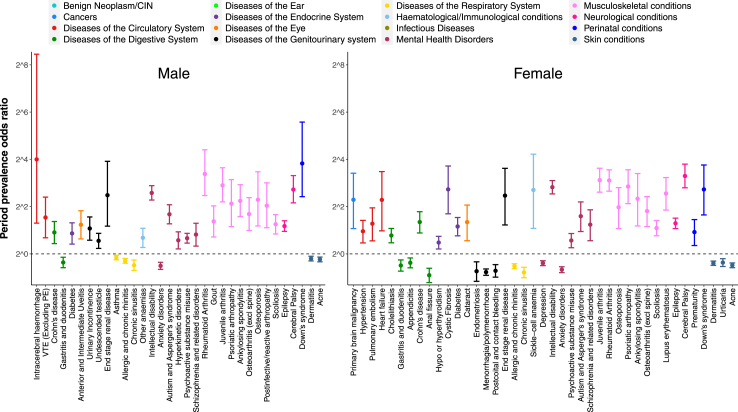
Conditions over- and under-represented within the prescription-only exposure subgroup; shown conditions were statistically significantly more (positive values) or less (negative values) prevalent within this subgroup compared to the rest of the exposed group. For this analysis, we defined statistical significance as a Fisher's exact test p-value <0.01, corrected for 228 (males) or 241 (females) comparisons. Error bars represent uncorrected 99% confidence limits.

Numerous conditions were over-represented within the prescription-exposed subgroup (26 among males, and 28 among females; summarized in Fig. 5), with musculoskeletal disease phenotypes constituting the greatest proportion of over-represented conditions. Phenotypes with potential autoimmune causes or associations were also over-represented in one or both genders, namely: Crohn's disease, uveitis, diabetes, thyroid disorders, and several musculoskeletal conditions.

We also noted a higher prevalence of intellectual disability, cerebral palsy, epilepsy, and autistic spectrum disorder in the prescription-only subgroup compared to the remainder of the exposed group. Down's syndrome and some commonly associated conditions (e.g. cataract, and undescended testicle) were also over-represented.

Several under-represented conditions were identified, including certain skin, respiratory, mental health, and gastrointestinal disorders. Among females, some gynaecological disorders were also under-represented. Overall, the number and magnitude of conditions which were under-represented was substantially smaller than those over-represented within the prescription-exposed subgroup.

### Sensitivity analyses

None of the sensitivity analyses meaningfully altered the observed associations between exposures and outcomes. Exclusion of certain analgesic drug groups did alter which conditions were found to be over-represented within the prescription-only exposed subgroup, although musculoskeletal disease phenotypes consistently made up the greatest proportion of these. Neither complete case analysis nor multiple imputation of missing data altered the direction of effect, nor negated the interpretation of findings in our core analyses (Adjusted substance misuse and mental health Cox models, and the negative binomial model for prescription opioid use). The results of all sensitivity analyses are presented in the Supplementary Material, Section 6.

## Discussion

Our results demonstrate associations between chronic pain in youth, and both mental illness and increased prescription opioid use in adulthood. By their 25th birthday, approximately 1 in 27 (3.67%) individuals in the cohort had received at least one repeat prescription for an analgesic medicine, and our analyses suggested that these ‘prescription-exposed’ individuals had a higher risk of substance misuse in later life, whereas this was not the case for those with a diagnosis of chronic pain in the absence of a repeat prescription for an analgesic medicine. This is the first study to explore outcomes of mental illness and substance misuse in CYP exposed to repeat prescriptions for analgesic medicines and chronic pain diagnoses in parallel.

Whilst it might be intuitive that chronic pain may increase the probability of mental illness, the data presented here provides evidence for this. Pain is unpleasant, and when severe, it can be profoundly disabling. Furthermore, conditions that cause pain may be stigmatised, as may chronic pain itself. Through this lens, chronic pain can be seen as prolonged or repeated exposure to stress and adversity, which are closely linked to mental health.^26^ There are also likely to be other important biological mechanisms that link the aetiology of pain and mental illness, for example genetic variants which may drive both primary pain conditions and mood and anxiety disorders.^27^^,^^28^ Specifically regarding the prescription-exposed subgroup, while our results may be explained by underlying chronic pain, multiple alternative or additional mechanisms may be implicated. For example, we identified substantial variation in the prevalence of a variety of conditions between exposure subgroups, and this may have impacted the observed associations. However, guided by this analysis, we performed a sensitivity analysis incorporating several additional covariates which did not meaningfully alter the results. Direct pharmacological effects offer a further possible explanation, and results from a recent mendelian randomisation study suggest a causal relationship may exist between the use of prescription opioids and the development of mood and anxiety disorders.^29^

Notable among our results, and in contrast to the findings of Groenewald et al.,^5^ is the lack of association between chronic pain identified by diagnosis-exposure and substance misuse in adulthood. This may be explained by our more diverse substance misuse outcome, which incorporates not just opioid misuse, but misuse of any substance. Similarly, chronic pain identified by a diagnostic code in EHRs is not equivalent to chronic pain identified by self-report or survey as used in this related work. Specifically, because substance misuse may be covert or sub-clinical, our use of routinely collected health data may have limited power to detect this association compared to survey response methods; this is supported by the relatively low frequency of substance misuse outcomes we identified compared to almost 30% of participants who reported prescription opioid misuse by age 34 in the work of Groenewald et al. Despite this, we were able to detect substantially greater hazards for substance misuse in the prescription- and both-exposed subgroups even though they had smaller sample sizes than the diagnosis-exposed subgroup. There are several plausible explanations for these disparate findings between subgroups. First, repeat prescription for an analgesic may indicate more frequent or severe pain, potentially effecting greater incentive to seek additional or alternative means to control it. Specifically, it is possible that the frequency of adverse outcomes studied in this paper are skewed heavily towards those with high impact chronic pain (HICP), and that those who have HICP are more likely to be prescribed analgesic medicines than other individuals with chronic pain. HICP is chronic pain that causes substantial interference with usual life and activities, and is also associated with the greatest healthcare utilization.^30^ Identification of not just pain chronicity but the impact of pain is important, but is challenging in routinely collected health data where even pain severity and duration can be difficult to ascertain. Despite this, identification of HICP in CYP based on interference has been attempted in at least one real world dataset, with the prevalence estimated to be 5% in the population studied.^31^ HICP was associated with more severe depressive and anxiety symptoms, as well as almost twice the number of missed days of school compared to those with chronic pain which was not determined to be ‘high impact’. The second possible explanation is that the comorbidities and predominant aetiology of pain may differ between subgroups. This appears likely given, for example, the markedly higher prevalence of inflammatory arthritides within the prescription-exposed subgroup. Third, the behavioural tendency to seek prescription pain medicines may simply correlate with the tendency to misuse illicit substances. Finally, prolonged use of analgesics may itself generate psychological or, in the case of opioids and some anti-neuropathic agents, physical dependence and addiction. Regardless of causality, receipt of a repeat prescription for an analgesic may be useful in predicting future substance misuse.

A recent systematic review and meta-analysis found that psychological therapies have the strongest evidence for chronic pain management in CYP.^32^ Despite this, pharmacological therapies are still the most commonly used in clinical practice, and they remain conditionally recommended by the WHO.^32^ Most likely, pharmacological measures will in many cases be logistically and economically preferable to psychological therapies, perhaps especially in resource-poor settings. Understanding the relationship between the use of analgesic medicines and health outcomes therefore has significant and global relevance. This has been a cause for much discussion and concern in particular because, in recent years, the incidence of prescription opioid use has greatly increased in many countries, including the UK.^33^ The potential harms of prescription opioids are well documented, and the utility of their long-term use in chronic pain is highly questionable, with a recent major guideline specifically recommending against the initiation of opioids in chronic primary pain.^34^^,^^35^ Despite this, we identified markedly increased rates of opioid prescribing among exposed individuals, in particular for those with prescription- or both-exposure. On the surface, the investigation of opioid prescription rates among those exposed to an analgesic might appear circular. However, it is important to understand how prescribing habits and patients' prior experiences may affect life-long healthcare behaviours and outcomes. Another of our findings which is to our knowledge novel is the association between exposure to certain non-opioid analgesics—with adjustment for exposure to various chronic pain conditions—and adverse substance misuse, mental health, and prescription opioid use outcomes in adulthood (Supplementary Material, Tables S11 and S13). While it should be emphasized that these results are from an unplanned supplementary analysis based on the results of this study, we suggest that, among CYP, first receipt of a repeat prescription for any conventional analgesic can be considered predictive of one or more adverse outcomes in adulthood. Notably, acute opioid prescription rates in adulthood were more similar in exposed and unexposed groups, while repeat prescriptions—the use of which is discouraged by the Faculty of Pain Medicine—were used at a far higher rate (data not shown).^36^ This may be due to ongoing or recurrent pain that responds well to opioids, and in these situations their use may not be wholly inappropriate. However, it is important to consider that the observed association may be explained by growing dependence-on and/or tolerance-to opioids, or a degree of ‘prescribing inertia’ and failure to appropriately de-prescribe. Importantly, these findings may themselves be linked to substance misuse and mental health outcomes. Breaking this cycle may therefore be an important intervention to address outcomes of substance misuse, mental illness, as well as over- and inappropriate prescribing.

A number of commonly painful musculoskeletal conditions, such as inflammatory arthritides, were over-represented within the prescription-exposed subgroup. Several of these conditions have documented associations with Crohn's disease, uveitis, diabetes, and/or thyroid disorders, and this may explain the coincident over-representation of these conditions.^37^ Sickle cell anaemia, another often-painful condition, was also over-represented in females. Among the other notable results was an unexpected and marked over-representation of intellectual disability, cerebral palsy, epilepsy, Down's syndrome, and autistic spectrum disorder (ASD) in the prescription-only subgroup. We make particular note of these findings given that pain in the intellectually disabled may often be mismanaged, having been described as under-recognised and under-treated among these vulnerable individuals.^38^ Our findings appear to be at odds with under-treatment, and could even represent overprescription. Overprescription of psychotropic medicines is well-documented in those with intellectual disability and ASD in particular, and it is possible that some analgesic agents may also be used in attempts to manage unexplained or challenging behaviour.^39^^,^^40^ Alternatively, because those with disabilities are more likely to be eligible for free prescriptions in the UK, it may be that they receive repeat prescriptions for medicines that can otherwise be purchased over the counter (OTC). Thus, these individuals may be disproportionately represented in analyses which use medicines as a proxy for a diagnosis. However, it should be noted that intellectual disability and cerebral palsy were still significantly more prevalent within the prescription-exposed group in a sensitivity analysis for which OTC medicines (paracetamol and weak NSAIDs) were excluded from the drug code list. It may be that chronic pain is more common in these individuals, for example intellectual disability is prevalent among children with cerebral palsy, which in turn is commonly associated with pain.^41^, ^42^, ^43^ Regardless, further investigation would appear warranted into not only under-treatment, but also the potential overprescription of analgesics in some of these vulnerable patient groups. Of additional note, while we expected over-representation of females within the prescription-exposed subgroup this is not what we observed, and is itself a notable finding. It may be because those in this exposure subgroup are more likely to have chronic secondary pain conditions, with the causative underlying conditions more evenly gender-distributed than often seen with primary pain conditions such as migraine or IBS. Alternatively, it may be due to increased analgesic seeking among males, or a greater tendency of physicians to prescribe analgesic medicines to males complaining of pain. Such a gender disparity in analgesic prescribing has been described in an emergency department context.^44^ However in general it is thought that the prevalence of analgesic use is higher among women, and this may be borne out in studies including non-prescription medicines.^45^

Primary care record databases are a useful means of allowing cost-effective exploration of long-term health associations over long time periods that may prohibit prospective methods. However, one limitation of the use of these data is the lack of sensitive diagnostic coding of some conditions, such as chronic pain.^46^ Indeed, the prevalence of chronic pain we observed is lower than would be expected based on the results of other epidemiological studies.^1^^,^^2^ This is likely due to both underdiagnosis, and an inherent methodological inability to detect pain managed without medical input within primary care data. Even if we assume that all of those with repeat prescriptions for analgesics have underlying chronic pain diagnoses, this prevalence estimate is still lower than has been found using more sensitive approaches such as survey-based methods. This may be due to conflation of chronic pain with a causative disease process (e.g. arthritis, peripheral neuropathy), rather than consideration of chronic pain as a distinct but related entity. Unreliable coding of chronic pain in primary care records presents challenges. First, failure to diagnose chronic pain creates a barrier to accessing evidence-based care and specialist services. Second, without a reliable way to define a chronic pain phenotype in health records, retrospective study is impeded; for example, studying the impact of chronic pain on other health outcomes, as well as the long-term use of medicines such as analgesics, which can be associated with harm.^34^^,^^47^^,^^48^ Potentially addressing some of these challenges, our finding that a variety of commonly painful conditions were over-represented within the prescription-exposed subgroup supports the possibility of leveraging ‘confounding by indication’, and for medicines data to be used as a proxy for disease. Inferring disease from medicines should be straightforward; where there is a medicine one should be able to infer its licenced indication. However, there are important accompanying challenges which are also illustrated here, where medicines may be used without a specific indication. Where there is a specific indication, this is often absent within coded records, or provided in the form of semi-structured natural language input which makes analysis substantially more complex. Knowing the range of indications for which such medicines can be used can overcome some of these challenges, and by exploring and quantifying this we have demonstrated a novel approach which may be valuable for iterative improvement of medicines-inferred disease phenotypes. This may be of use more broadly in EHR research, as a precursor to more time- and resource-intensive validation studies. Regarding the present study, irrespective of whether the prescription-exposed subgroup is truly representative of an undiagnosed chronic pain cohort, or whether the associations are causal or not, our findings suggest that exposure to a repeat prescription for analgesic medicines in youth may have independent value in predicting substance misuse, mental illness, and chronic or recurrent opioid use in adulthood.

### Strengths and weaknesses

Strengths of this research include the large sample size, correction for a number of covariates, and robustness of our results to various sensitivity analyses. Additionally, our exposed group consisted of a broad range of individuals with presumed chronic pain and also analgesic medicine exposure, and while this may allow some inferences to be made about chronic pain and analgesic repeat prescription exposure in general, it also results in a lack of resolution to identify specific subgroups whose outcomes may markedly differ, but be masked by pooling. We addressed this in part by performing supplementary analyses with sub-stratified exposures, although this included multiple comparisons (22 additional comparisons for each of the three core outcomes) and its results should therefore be interpreted with caution. Weaknesses of the study include the low event rate for substance misuse outcomes, which is likely under-estimated as these may often be sub-clinical or not recorded in the EHR. Additionally, we are not aware of validated algorithms for identifying paediatric chronic pain in electronic medical records using the Read V2 clinical coding system, and so were not able to use one. While other studies have used algorithms including prescription records,^46^ our approach using repeat prescriptions as an exposure has also not been validated, and, other than what we have elucidated by comparing condition prevalence between exposure groups, we have little certainty about the characteristics of individuals identified using this approach. Despite this, agreement was reached between clinicians that the presence of any of the included diagnostic codes was highly likely to represent chronic pain; all codes included either explicitly mention chronic or persistent pain, or relate to conditions of chronic pain under ICD-11 definitions. A validation study would complement and enhance the value of this work, but was not within the scope of our investigation. It may also be of value to formally assess for causality of the observed associations using path analysis, however such assessments may have other limitations which are similar to the present study. For example, another limitation is unaddressed confounding due to unknown or unmeasured factors such as patterns of associated health conditions which may be multi-dimensional, as well as social, psychological, and other more abstract circumstances such as severity of pain and stigmatisation, which are not easily ascertainable with the available data. Also inevitable is a degree of measurement error for exposures, follow-up durations, and time-to-outcome, which may affect the reliability of our results. Studies within other EHR databases which use algorithms incorporating diagnostic codes, prescription records, and pain scores, report achieving reasonable sensitivity and specificity (84.8% and 97.7%) for identifying chronic pain.^46^ Our approach has not been validated, and due to the absence of pain scores may be significantly less-sensitive. For prescription records, both as an exposure and an outcome, while structured and usually very reliable data are available, it is important to note that prescribing or dispensing a medicine does not guarantee an individual takes or is truly exposed to said medicine. Where an individual does take the medicine, it may not be for some time after the dispense date, also resulting in imprecision. Because of our interest in investigating outcomes beyond the age of 24, it was not possible to include any patients born after 1994, as they would have insufficient or zero follow-up time. While deficits in mortality reporting have been identified within THIN database prior to around the year 2000,^49^ the dataset has to some extent been validated as far back as 1986,^50^ and because the entirety of our follow-up period was beyond 2000 we do not expect immortal periods in pre-2000 data to materially affect our key results. Finally, it should be noted that our findings cannot be generalised to certain groups, such as those who do not seek medical input or use prescribed analgesic medicines, who may constitute a substantial proportion of the chronic pain population.

### Recommendations

The management of chronic or recurrent pain is complex, especially in CYP. It is essential that pain is recognised and addressed as best as possible, and this will often necessitate use of analgesic medicines. These medicines can certainly be used in a safe manner to effectively relieve some forms of pain, especially acute and nociceptive pain, and the harms of under-treating pain should not be overlooked. However, medicines carry their own risk of harm, and many are not licensed for use in paediatric patients. It may therefore be difficult to strike a balance between the risk of harms from unrelieved pain, and the risks posed by medicines. While pain may itself predispose to adverse outcomes, emotional functioning and the development of effective coping strategies are also thought to be instrumental resilience factors that may improve future outcomes for CYP with chronic pain.^51^ It is plausible that long-term reliance on analgesic medicines in early life may hinder the development of these strategies, and could result in self-medication, drug-seeking, dependence, and the transition to illicit alternatives if prescription drugs are later withdrawn or tolerance develops. Where they are used, whether on- or off-label, analgesic medicines should be considered judiciously, and with due regard for these potential harms, as well as alternative or additional treatment modalities that may be available. This is especially the case for chronic pain, where prolonged use of analgesic medicines may not only be less effective than alternative approaches, but also cumulative exposure may potentiate or magnify their harms. Interpreting our results through this broader lens, we suggest some steps that may improve long-term outcomes for CYP with chronic pain. First, clinicians should aim for timely and consistent recognition and coding of chronic pain in CYP, with particular improvements needed where it may ‘hide in plain sight’ secondary to a chronic organic condition. This is important because where pain is recognised and managed as chronic (rather than e.g. as recurrent acute pain or an arthritis flare), especially where it is identified as primary or nociplastic pain, clinicians may be more likely to engage with specialist services or consider alternatives to pharmacological/opioid treatments. The perceived need to issue a repeat prescription for an analgesic should perhaps prompt clinicians to reassess the nature of pain, and consider management alternatives. Second, improvements in access to specialist pain services and non-pharmacological management options for CYP could help patients to develop coping strategies which may have more longevity than analgesic medicines. Self-management approaches, including educational and psychotherapy-based interventions, may reduce the physical and psychosocial burden of chronic pain.^52^ Their use emphasizes the importance of non-pharmacological management options and may even help to reduce long-term opioid use.^52^^,^^53^ These strategies may be especially valuable in later childhood and adolescence: a time when there is commonly a transition of care for the management of chronic conditions.^54^^,^^55^ Greater access to self-management services and educational programs may therefore play an important role in improving outcomes among CYP with chronic pain. Finally, where possible, clinicians should aim to avoid using long-term opioids to manage chronic pain, especially via repeat prescriptions and in chronic primary pain.

### Conclusions

Chronic pain in CYP appears to be associated with adverse mental health and prescription opioid use outcomes in adulthood. Use of repeat prescriptions for analgesic medicines in early life is also associated with both of these adverse outcomes, but also substance misuse. The perceived need for a repeat prescription may be an important ‘wake-up call’ to indicate the transition to chronic pain, which may be even more underdiagnosed in CYP than in adults, and for which alternative management strategies may be advisable. Furthermore, such prescriptions may be more predictive of adverse outcomes than some chronic pain conditions themselves, but these associations may be non-causal, or even unrelated to pain. Medicines usage may be a useful way of inferring disease where diagnostic coding is unreliable or absent in EHRs. Timely recognition of chronic pain in CYP, and increasing access to specialist pain services, may be important steps in avoiding adverse outcomes in adulthood, including long-term reliance on pain medicines.

## Contributors

AL, RS, LW and DS conceptualised the study. RS and LW supervised the work. CJ and AL curated data, and RT provided technical support with data curation and analysis. AL, MK, CJ, AW, CD, MK, DPM, LW and RS developed and/or advised on analysis methodology. Formal analysis and data visualisation was performed by AL. AL and RS wrote the first draft, and MK, CJ, AW, CD, AM, DS, MK, DPM, and LW provided comments and contributed to critical revision of the manuscript. All authors read and approved the final draft.

## Data sharing statement

All diagnostic and drug code lists, as well as a description of their implementation, are provided in the accompanying Supplementary Material. Machine-readable code lists or a copy of the protocol will be provided by the corresponding author upon reasonable request.

## Declaration of interests

RS and DS are part of the Advanced Pain Discovery Platform and were supported by a UKRI and Versus Arthritis grant (MR/W002566/1) as part of the Consortium Against Pain Inequality. AW was supported by the Wellcome Trust during the preparation of this manuscript (220558/Z/20/Z). LW has received research funding from Hong Kong Innovation and Technology Commission, Diabetes UK, The Cure Parkinson's Trust, and BOPA-PRUK.
