# Supplementary material for: Prevalence of chronic pain or analgesic use in children and young people and its long-term impact on substance misuse, mental illness, and prescription opioid use: a retrospective longitudinal cohort study
Source: Lancet Reg Health Eur. 2023 Nov 15;35:100763. doi: 10.1016/j.lanepe.2023.100763 (PMC10730316; doi:10.1016/j.lanepe.2023.100763)
Supplement: Supplementary Material [file mmc1.docx]

Supplementary Material

Section 1: Sources and implementation details for diagnostic and drug code lists2

Section 2: Full diagnostic and drug code lists4

Chronic pain4

Mental illness5

Substance misuse17

Epilepsy22

Cerebral palsy24

Autistic spectrum disorder24

Intellectual disability24

Malignancy25

Smoking38

Analgesic drug codes40

Section 3: Mental health and substance misus**e** survival analysis (sub-stratified exposures)66

Section 4: Opioid prescription rates detailed results tables67

Section 5: Exposure subgroup condition prevalence comparison full results tables69

Section 6: Sensitivity analyses79

Mental illness outcomes79

Substance misuse outcomes81

Prescription opioid use outcomes83

Stratified complete cases Cox models and alternative missing data approaches (e.g. multiple imputation)85

Exposure-subgroup condition comparisons87

Section 7: STROBE checklist91

Section 8: Directed acyclic graph94

# Section 1: Description of diagnostic and drug code lists

Supplementary Table S1: sources and implementation of phenotype and covariate code lists

|  | Source | Adaptations/alterations | Implementation |
| --- | --- | --- | --- |
| Chronic pain | Newly-curated | N/A | Individual considered to have chronic pain as a child or young person if one of these codes exists within their medical record before their 25^th^ birthday. Groupings used for sub-stratified exposure analysis (by phenotype or group of phenotypes) are given in the column ‘condition’.  Notes: We systematically curated a list of Read V2 clinical codes whose description we perceived to be strongly suggestive of CP. Codes describing any site or cause of pain as ‘intractable’, ‘persistent’, or ‘chronic’ were included, as were those suggesting engagement with specialist pain services. Codes for neuropathic pain and dysmenorrhoea were included, as were those for conditions categorised under chronic primary pain within ICD-11.^2^ As such, eligible conditions included: chronic musculoskeletal pain, fibromyalgia, chronic regional pain syndrome, erythromelalgia, neuropathic pain, chronic headache, trigeminal neuralgia, primary bladder pain, chronic or recurrent abdominal pain (including irritable bowel syndrome), dysmenorrhea, psychogenic pain, primary orofacial pain, trigeminal autonomic cephalalgia, chronic breast pain, and chronic pain without a specified cause.  Codes for chronic conditions which can commonly cause secondary pain, but are not by definition conditions of chronic pain (e.g. sickle cell anaemia and inflammatory arthritides), were not included. This decision was motivated both by our hypothesis that individuals with chronic pain could be identified by prescription records, and to maintain as much specificity as possible at the potential cost of a more sensitive case definition. Our rationale was that those with an unremitting nociceptive cause of secondary pain, or with chronic pain after remission, may continue to receive analgesic medicines, and that those without ongoing pain would likely cease to receive or fill prescriptions. |
| Mental illness | Morgan et al 2018 (anxiety spectrum, depression, self-harm and suicide, bipolar affective disorder, mental health service referral, personality disorders, schizophrenia), accessed 03/03/2021. Link:  https://clinicalcodes.rss.mhs.man.ac.uk/medcodes/article/69/  Windfuhr et al 2016 (eating disorders), accessed 03/03/2021. Link:  https://clinicalcodes.rss.mhs.man.ac.uk/medcodes/article/38/ | Lists from two sources combined into one. Psychalgia, psychogenic pain, and related codes were excluded, and are considered as pain codes instead. | As a baseline covariate (prior mental illness present/absent prior to start of follow-up), if one of these codes exists within an individual’s medical record prior to the 25^th^ birthday, they are considered to have a prior mental illness. As an outcome event, first instance of one of these codes after the start of follow-up is considered the first mental illness event. |
| Substance misuse | Adapted from Gorton et al 2018, accessed 03/03/2021. Link: https://clinicalcodes.rss.mhs.man.ac.uk/medcodes/article/55/codelist/res55-substance_misuse/ | Additional codes were identified by browsing other published code lists and manual search of a Read V2 code browser. Added codes are marked with ‘New’ in the ‘Source’ column. | As a baseline covariate (prior substance misuse present/absent prior to start of follow-up), if any one of these codes exists within an individual’s medical record prior to the 25^th^ birthday, they are considered to have prior substance misuse. As an outcome event, first instance of one of the codes marked ‘FALSE’ in the column ‘Past’ is considered the first substance misuse event. |
| Epilepsy | Kuan et al 2019, accessed 02/01/2022. Link: https://portal.caliberresearch.org/phenotypes/kuan-epilepsy-wg2um5mf2unhq57ya44gzl | None | As a confounding/exclusionary diagnosis, presence of any one of these codes prior-to or up to six months post-exposure to a gabapentinoid. |
| Cerebral palsy | Kuan et al 2019, accessed 02/01/2022. Link: https://portal.caliberresearch.org/phenotypes/kuan-cerebral-palsy-lowh6jee99hfyv6wwxwa8j | None | As a baseline covariate (cerebral palsy present/absent prior to start of follow-up), if any one of these codes exists within an individual’s medical record prior to the 25^th^ birthday, they are considered to have prior cerebral palsy. |
| Autistic spectrum disorder | Kuan et al 2019, accessed 02/01/2022. Link: https://portal.caliberresearch.org/phenotypes/kuan-autism-ywsdewqkrrrzhmofaarkmp | None | As a baseline covariate (Autistic spectrum disorder present/absent prior to start of follow-up), if any one of these codes exists within an individual’s medical record prior to the 25^th^ birthday, they are considered to have prior autistic spectrum disorder. |
| Intellectual disability | Kuan et al 2019, accessed 02/01/2022. Link: https://portal.caliberresearch.org/phenotypes/kuan-intell-dz-nmjhav5bgsxc8ownrqqaes | None | As a baseline covariate (Intellectual disability present/absent prior to start of follow-up), if any one of these codes exists within an individual’s medical record prior to the 25^th^ birthday, they are considered to have prior intellectual disability. |
| Malignancy | Kuan et al 2019, accessed 02/01/2022. Link: https://phenotypes.healthdatagateway.org/phenotypes/ | All code lists including the string “Malignancy” in the title were combined into one list | As a baseline covariate (Prior malignancy present/absent prior to start of follow-up), if any one of these codes exists within an individual’s medical record prior to the 25^th^ birthday, they are considered to have prior malignancy. |
| Smoking | George et al 2013, accessed 02/01/2022. Link: https://www.caliberresearch.org/portal/show/smoking_status_gprd | Two codes categorized as ‘Ex or current smoker’ were instead categorized as ‘Unknown’ | As a baseline covariate, the most recent event prior to the 25^th^ birthday was used. Both diagnostic codes and associated health data were queried. Ex-smoker classification was contingent on a previous ‘Smoker’ or ‘Ex-smoker’ code existing in the medical record. Non-smoker classification was contingent on no previous ‘Smoker’ or ‘Ex-smoker’ codes. This procedure followed an algorithm recommended in IQVIA research guidance documentation. |
| Alcohol consumption | IQVIA research guidance documentation | N/A | As a baseline covariate, the most recent alcohol consumption status event recorded in the associated health data file prior to the 25^th^ birthday was used. |
| Body mass index | IQVIA research guidance documentation | N/A | As a baseline covariate, the most recent body mass index event recorded in the associated health data file prior to the 25^th^ birthday was used. Values <13 and >60 were excluded. |
| Analgesic medicines | Hand-curated from drug code dictionary provided by IQVIA | N/A | Individual considered exposed to a repeat prescription for an analgesic if any one of these codes existed (and was associated with a flag denoting a repeat prescription) within their medical record prior to the 25^th^ birthday. For gabapentinoids, repeat prescription events were not eligible exposures if there existed a code for mental illness or epilepsy (as defined above) prior-to or up to six months after the prescription event. For antidepressants, repeat prescription events were not eligible exposures if there existed a code for mental illness prior-to or up to six months after the prescription event. Groupings for sub-stratified exposure analysis (by class of analgesic) are given in the column ‘class’.  Notes: We included a list of drugs that are commonly prescribed for pain-relief, including (exhaustively) the following active substances: paracetamol, ibuprofen, diclofenac, naproxen, aspirin, codeine, dihydrocodeine, tramadol, oxycodone, morphine, fentanyl, buprenorphine, diamorphine, gabapentin, pregabalin, duloxetine, and amitriptyline. This list was reviewed by two clinicians and certain formulations were excluded if judged likely to be used for non-pain indications (e.g. buccal/sublingual buprenorphine).  We recognised that certain substances may have common alternative uses; pregabalin and gabapentin are often used in epilepsy and mental illness, and amitriptyline and duloxetine are also commonly used in mental illness. Therefore, prescriptions of these medicines were not considered eligible as exposures if there existed a relevant diagnosis of one these conditions prior-to or up to six months after the prescription. We also performed a sensitivity analysis excluding these agents altogether.  For anti-neuropathic drugs we included only agents recommended as initial treatment in NICE guidance (CG173). Other anti-neuropathic medicines were not included as these were not licensed for the management of neuropathic pain and/or were perceived to have important alternative indications (e.g. nortriptyline which is not licensed for neuropathic pain and can also be used for nocturnal enuresis). |
| Townsend quintile | IQVIA research guidance documentation | N/A | Townsend score is a measure of deprivation in a given geographical location based on four variables which correlate with deprivation. Higher scores indicate higher deprivation, and within the IMRD database individuals are assigned a Townsend quintile based on the score associated with the location of their registered practice at a given time. We identified the most recent Townsend quintile assigned to a given individual before their 25^th^ birthday when they entered follow-up, and this was used as their value for this covariate. |

# Section 2: Full diagnostic and drug code lists

# Supplementary Table S2: Chronic pain codes

| **description** | **code** | **condition** |
| --- | --- | --- |
| H/O: migraine | 1474.00 | migraine |
| H/O migraine with aura | 1474000 | migraine |
| History of irritable bowel syndrome | 14CF.00 | abdo |
| H/O: dysmenorrhoea | 1574.00 | menstrual |
| H/O: painful periods | 1574.11 | menstrual |
| Chronic low back pain | 16C9.00 | msk |
| Abdominal migraine - symptom | 1967.00 | migraine |
| Central post-stroke pain | 1M4..00 | neuropathic |
| Chronic pain | 1M52.00 | chronic_pain |
| Diabetic peripheral neuropathic pain | 1M8..00 | neuropathic |
| Intractable breast pain | 26BD.00 | breast |
| Persistent mastalgia | 26BF.00 | breast |
| Chronic pain review | 66n..00 | chronic_pain |
| Delivery of rehabilitation for pain syndromes | 7P21200 | chronic_pain |
| Refer to pain clinic | 8H69.00 | chronic_pain |
| Referral to back pain clinic | 8HTH.00 | msk |
| Private referral to pain management service | 8HVk.00 | chronic_pain |
| Pain management (specialty) | 9b8F.00 | chronic_pain |
| Seen in pain clinic | 9N1k.00 | chronic_pain |
| Under care of pain management specialist | 9NNh.00 | chronic_pain |
| Post-herpetic neuralgia | A531.11 | neuropathic |
| Postherpetic trigeminal neuralgia | A531200 | tgn |
| Postzoster neuralgia | A531500 | neuropathic |
| Chronic pain syndrome | E278.00 | chronic_pain |
| Psychogenic pain unspecified | E278000 | psychogenic |
| Psychalgia NOS | E278z00 | psychogenic |
| [X]Persistent somatoform pain disorder | Eu45400 | psychogenic |
| [X]Psychalgia | Eu45411 | psychogenic |
| [X]Psychogenic backache | Eu45412 | psychogenic |
| [X]Somatoform headache | Eu45413 | psychogenic |
| [X]Somatoform pain disorder | Eu45414 | psychogenic |
| [X]Chronic pain personality syndrome | Eu62y11 | psychogenic |
| Shoulder-hand syndrome | F173.00 | crps_erythromel |
| Migraine | F26..00 | migraine |
| Classical migraine | F260.00 | migraine |
| Common migraine | F261.00 | migraine |
| Atypical migraine | F261000 | migraine |
| Sick headache | F261100 | migraine |
| Common migraine NOS | F261z00 | migraine |
| Migraine variants | F262.00 | migraine |
| Cluster headache | F262000 | tac |
| Horton's (histamine) neuralgia | F262100 | tac |
| Abdominal migraine | F262200 | migraine |
| Basilar migraine | F262300 | migraine |
| Ophthalmic migraine | F262400 | migraine |
| Periodic migrainous neuralgia | F262500 | migraine |
| Periodic migrainous neuralgia | F262500 | migraine |
| Chronic paroxysmal hemicrania | F262700 | tac |
| Frequent episodic tension-type headache | F262A00 | other_headache |
| Chronic tension-type headache | F262B00 | other_headache |
| Shortlasting, unilateral, neuralgiform pain with conjunctival injection and tearing syndrome | F262C00 | tac |
| Shortlasting, unilateral, neuralgiform pain with conjunctival injection and tearing syndrome | F262C00 | tac |
| Paroxysmal hemicrania | F262D00 | tac |
| Trigeminal autonomic cephalalgia | F262E00 | tac |
| Migraine variant NOS | F262z00 | migraine |
| Other forms of migraine | F26y.00 | migraine |
| Hemiplegic migraine | F26y000 | migraine |
| Ophthalmoplegic migraine | F26y100 | migraine |
| Status migrainosus | F26y200 | migraine |
| Complicated migraine | F26y300 | migraine |
| Other forms of migraine NOS | F26yz00 | migraine |
| Migraine NOS | F26z.00 | migraine |
| Post-herpetic trigeminal neuralgia | F300.00 | tgn |
| Other specified trigeminal neuralgia | F301.00 | tgn |
| Tic douloureux | F301000 | tgn |
| Trigeminal neuralgia NOS | F301z00 | tgn |
| Glossopharyngeal neuralgia | F321.00 | neuropathic |
| Phantom limb syndrome with pain | F336000 | neuropathic |
| Causalgia | F344.00 | crps_erythromel |
| Complex regional pain syndrome type II | F347.00 | crps_erythromel |
| Morton's neuralgia | F356100 | neuropathic |
| Complex regional pain syndrome | F369.00 | crps_erythromel |
| Chronic regional pain syndrome | F369.11 | crps_erythromel |
| Chronic painful diabetic neuropathy | F372100 | neuropathic |
| [X]Chronic headache disorder | Fyu5E00 | other_headache |
| [X]Retrobulbar neuritis in diseases classified elsewhere | FyuJ100 | neuropathic |
| Erythromelalgia | G73y800 | crps_erythromel |
| Erythralgia | G73y811 | crps_erythromel |
| Temporomandibular joint-pain-dysfunction syndrome | J046400 | primary_orofacial |
| Burning mouth syndrome | J08zC00 | primary_orofacial |
| Irritable bowel syndrome | J521.11 | abdo |
| Irritable bowel syndrome with diarrhoea | J521000 | abdo |
| Irritable bowel syndrome characterised by constipation | J521100 | abdo |
| Irritable bowel syndrome characterised by alternating bowel habit | J521200 | abdo |
| Chronic interstitial cystitis | K151.00 | primary_bladder |
| Hunner's ulcer | K151000 | primary_bladder |
| Panmural fibrosis of bladder | K151100 | primary_bladder |
| Submucous cystitis | K151200 | primary_bladder |
| Chronic interstitial cystitis NOS | K151z00 | primary_bladder |
| Mittelschmerz - ovulation pain | K582.00 | menstrual |
| Painful menorrhoea | K583.11 | menstrual |
| Primary dysmenorrhoea | K583000 | menstrual |
| Secondary dysmenorrhoea | K583100 | menstrual |
| Migraine - menstrual | K584.11 | migraine |
| Postlaminectomy syndrome | N12A.00 | msk |
| Postlaminectomy syndrome of unspecified site | N12A000 | msk |
| Cervical postlaminectomy syndrome | N12A100 | msk |
| Thoracic postlaminectomy syndrome | N12A200 | msk |
| Lumbar postlaminectomy syndrome | N12A300 | msk |
| Postlaminectomy syndrome NOS | N12Az00 | msk |
| Prolapsed lumbar intervertebral disc with sciatica | N12C400 | msk |
| Lumbago with sciatica | N142000 | msk |
| Sciatica | N143.00 | neuropathic |
| Thoracic neuritis, unspecified | N144000 | neuropathic |
| Thoracic nerve root pain | N144011 | neuropathic |
| Scapulohumeral fibrositis | N212100 | fibromyalgia |
| Fibrositis unspecified | N240100 | fibromyalgia |
| Fibrositis of neck | N240500 | fibromyalgia |
| Fibrositis arm | N240600 | fibromyalgia |
| Neuralgia unspecified | N242000 | neuropathic |
| Neuropathic pain | N242300 | neuropathic |
| Fibromyalgia | N248.00 | fibromyalgia |
| Myofascial pain syndrome | N248000 | fibromyalgia |
| Piriformis syndrome | N248100 | neuropathic |
| Algoneurodystrophy | N337.00 | crps_erythromel |
| Algoneurodystrophy | N337.00 | crps_erythromel |
| Algodystrophy | N337.11 | crps_erythromel |
| Sudek's atrophy | N337100 | crps_erythromel |
| Reflex sympathetic dystrophy | N337112 | crps_erythromel |
| Algodystrophy of hand | N337200 | crps_erythromel |
| Algodystrophy of knee | N337300 | crps_erythromel |
| Algodystrophy of foot | N337400 | crps_erythromel |
| Algoneurodystrophy NOS | N337z00 | crps_erythromel |
| Complex regional pain syndrome type I | N33C.00 | crps_erythromel |
| [D]Chronic intractable pain | R00zC00 | chronic_pain |
| [D]Abdominal migraine | R090D00 | migraine |
| [D]Recurrent acute abdominal pain | R090E00 | abdo |
| [D]Functional abdominal pain syndrome | R090P00 | abdo |
| [X]Other chronic pain | Ryu7000 | chronic_pain |
| Irritable colon - irritable bowel syndrome | J521.00 | abdo |
| Management of irritable bowel syndrome | 8Cm..00 | abdo |
| Spastic colon | J521.13 | abdo |
| Migraine prophylaxis | 8B6N.00 | migraine |
| Migraine with aura | F260.11 | migraine |
| Migraine without aura | F261.11 | migraine |
| Migraine induced by oestrogen contraceptive | F262800 | migraine |
| Moebius' ophthalmoplegic migra | F26y111 | migraine |
| [X]Other migraine | Fyu5300 | migraine |
| [X]Psychogenic dysmenorrhoea | Eu45y11 | menstrual |
| Dysmenorrhoea | K583.00 | menstrual |
| Painful menstruation | K583.12 | menstrual |
| Period pains | K583.13 | menstrual |
| Spasmodic dysmenorrhoea | K583.14 | menstrual |
| H/O: trigeminal neuralgia | 1475.00 | tgn |

# Supplementary Table S3: Mental illness codes

| **description** | **code** | **source** |
| --- | --- | --- |
| o/e - panic attack | 225J.00 | Anxiety_spectrum |
| Antiphobic therapy | 8G52.00 | Anxiety_spectrum |
| anxiety management training | 8G94.00 | Anxiety_spectrum |
| referral for guided self-help for anxiety | 8HHp.00 | Anxiety_spectrum |
| Neurotic; personality and other nonpsychotic disorders | E2...00 | Anxiety_spectrum |
| Neurotic disorders | E20..00 | Anxiety_spectrum |
| anxiety states | E200.00 | Anxiety_spectrum |
| anxiety state unspecified | E200000 | Anxiety_spectrum |
| panic disorder | E200100 | Anxiety_spectrum |
| panic attack | E200111 | Anxiety_spectrum |
| generalised anxiety disorder | E200200 | Anxiety_spectrum |
| anxiety with depression | E200300 | Anxiety_spectrum |
| chronic anxiety | E200400 | Anxiety_spectrum |
| recurrent anxiety | E200500 | Anxiety_spectrum |
| anxiety state nos | E200z00 | Anxiety_spectrum |
| Hysteria | E201.00 | Anxiety_spectrum |
| Hysteria unspecified | E201000 | Anxiety_spectrum |
| Hysterical blindness | E201100 | Anxiety_spectrum |
| Hysterical deafness | E201200 | Anxiety_spectrum |
| Hysterical tremor | E201300 | Anxiety_spectrum |
| Hysterical paralysis | E201400 | Anxiety_spectrum |
| Hysterical seizures | E201500 | Anxiety_spectrum |
| Fit - hysterical | E201511 | Anxiety_spectrum |
| Other conversion disorder | E201600 | Anxiety_spectrum |
| Astasia - abasia; hysterical | E201611 | Anxiety_spectrum |
| Globus hystericus | E201612 | Anxiety_spectrum |
| Hysterical amnesia | E201700 | Anxiety_spectrum |
| Hysterical fugue | E201800 | Anxiety_spectrum |
| Multiple personality | E201900 | Anxiety_spectrum |
| Dissociative reaction unspecified | E201A00 | Anxiety_spectrum |
| Compensation neurosis | E201B00 | Anxiety_spectrum |
| Phantom pregnancy | E201C00 | Anxiety_spectrum |
| Hysteria NOS | E201z00 | Anxiety_spectrum |
| Aphonia - hysterical | E201z11 | Anxiety_spectrum |
| Ataxia - hysterical | E201z12 | Anxiety_spectrum |
| Ganser's syndrome - hysterical | E201z13 | Anxiety_spectrum |
| Phobic disorders | E202.00 | Anxiety_spectrum |
| Phobia unspecified | E202000 | Anxiety_spectrum |
| agoraphobia with panic attacks | E202100 | Anxiety_spectrum |
| Social phobic disorders | E202.11 | Anxiety_spectrum |
| Phobic anxiety | E202.12 | Anxiety_spectrum |
| Agoraphobia without mention of panic attacks | E202200 | Anxiety_spectrum |
| Social phobia; fear of eating in public | E202300 | Anxiety_spectrum |
| Social phobia; fear of public speaking | E202400 | Anxiety_spectrum |
| Social phobia; fear of public washing | E202500 | Anxiety_spectrum |
| Acrophobia | E202600 | Anxiety_spectrum |
| Animal phobia | E202700 | Anxiety_spectrum |
| Claustrophobia | E202800 | Anxiety_spectrum |
| Fear of crowds | E202900 | Anxiety_spectrum |
| Fear of flying | E202A00 | Anxiety_spectrum |
| Cancer phobia | E202B00 | Anxiety_spectrum |
| Dental phobia | E202C00 | Anxiety_spectrum |
| Fear of death | E202D00 | Anxiety_spectrum |
| Fear of pregnancy | E202E00 | Anxiety_spectrum |
| Phobic disorder NOS | E202z00 | Anxiety_spectrum |
| Weight fixation | E202z11 | Anxiety_spectrum |
| Neurasthenia - nervous debility | E205.00 | Anxiety_spectrum |
| Nervous exhaustion | E205.11 | Anxiety_spectrum |
| Depersonalisation syndrome | E206.00 | Anxiety_spectrum |
| Hypochondriasis | E207.00 | Anxiety_spectrum |
| Other neurotic disorders | E20y.00 | Anxiety_spectrum |
| Somatization disorder | E20y000 | Anxiety_spectrum |
| Briquet's disorder | E20y011 | Anxiety_spectrum |
| Writer's cramp neurosis | E20y100 | Anxiety_spectrum |
| Other occupational neurosis | E20y200 | Anxiety_spectrum |
| Psychasthenic neurosis | E20y300 | Anxiety_spectrum |
| Other neurotic disorder NOS | E20yz00 | Anxiety_spectrum |
| Neurotic disorder NOS | E20z.00 | Anxiety_spectrum |
| Nervous breakdown | E20z.11 | Anxiety_spectrum |
| Physiological malfunction arising from mental factors | E26..00 | Anxiety_spectrum |
| Psychogenic musculoskeletal symptoms | E260.00 | Anxiety_spectrum |
| Psychogenic paralysis | E260000 | Anxiety_spectrum |
| Psychogenic torticollis | E260100 | Anxiety_spectrum |
| Psychogenic musculoskeletal symptoms NOS | E260z00 | Anxiety_spectrum |
| Psychogenic respiratory symptoms | E261.00 | Anxiety_spectrum |
| Psychogenic air hunger | E261000 | Anxiety_spectrum |
| Psychogenic cough | E261100 | Anxiety_spectrum |
| Psychogenic hiccough | E261200 | Anxiety_spectrum |
| Psychogenic hyperventilation | E261300 | Anxiety_spectrum |
| Psychogenic yawning | E261400 | Anxiety_spectrum |
| Psychogenic aphonia | E261500 | Anxiety_spectrum |
| Psychogenic respiratory symptom NOS | E261z00 | Anxiety_spectrum |
| Psychogenic cardiovascular symptoms | E262.00 | Anxiety_spectrum |
| Cardiac neurosis | E262000 | Anxiety_spectrum |
| Neurocirculatory asthenia | E262200 | Anxiety_spectrum |
| Psychogenic cardiovascular disorder | E262300 | Anxiety_spectrum |
| Psychogenic cardiovascular symptom NOS | E262z00 | Anxiety_spectrum |
| Psychogenic skin symptoms | E263.00 | Anxiety_spectrum |
| Psychogenic pruritus | E263000 | Anxiety_spectrum |
| Psychogenic skin symptoms NOS | E263z00 | Anxiety_spectrum |
| Psychogenic gastrointestinal tract symptoms | E264.00 | Anxiety_spectrum |
| Psychogenic aerophagy | E264000 | Anxiety_spectrum |
| Air swallowing - excessive | E264011 | Anxiety_spectrum |
| Globus abdominalis | E264.11 | Anxiety_spectrum |
| Cyclical vomiting - psychogenic | E264200 | Anxiety_spectrum |
| Psychogenic diarrhoea | E264300 | Anxiety_spectrum |
| Psychogenic dyspepsia | E264400 | Anxiety_spectrum |
| Psychogenic constipation | E264500 | Anxiety_spectrum |
| Psychogenic gastrointestinal tract symptom NOS | E264z00 | Anxiety_spectrum |
| Psychogenic genitourinary tract symptoms | E265.00 | Anxiety_spectrum |
| Psychogenic vaginismus | E265100 | Anxiety_spectrum |
| Psychogenic dysmenorrhea | E265200 | Anxiety_spectrum |
| Psychogenic dysuria | E265300 | Anxiety_spectrum |
| Psychogenic genitourinary tract symptom NOS | E265z00 | Anxiety_spectrum |
| Psychogenic symptom of special sense organ | E267.00 | Anxiety_spectrum |
| Other psychogenic malfunction | E26y.00 | Anxiety_spectrum |
| Bruxism (teeth grinding) | E26y000 | Anxiety_spectrum |
| Other psychogenic malfunction NOS | E26yz00 | Anxiety_spectrum |
| Psychosomatic disorder NOS | E26z.00 | Anxiety_spectrum |
| Psychalgia | E278.00 | Anxiety_spectrum |
| Psychogenic pain unspecified | E278000 | Anxiety_spectrum |
| Psychogenic backache | E278200 | Anxiety_spectrum |
| Psychalgia NOS | E278z00 | Anxiety_spectrum |
| Acute reaction to stress | E28..00 | Anxiety_spectrum |
| acute panic state due to acute stress reaction | E280.00 | Anxiety_spectrum |
| Acute fugue state due to acute stress reaction | E281.00 | Anxiety_spectrum |
| Combat fatigue | E28..11 | Anxiety_spectrum |
| Acute stupor state due to acute stress reaction | E282.00 | Anxiety_spectrum |
| Other acute stress reactions | E283.00 | Anxiety_spectrum |
| Acute situational disturbance | E283000 | Anxiety_spectrum |
| Acute posttrauma stress state | E283100 | Anxiety_spectrum |
| Other acute stress reaction NOS | E283z00 | Anxiety_spectrum |
| Stress reaction causing mixed distrurbance of emotion/conduct | E284.00 | Anxiety_spectrum |
| Acute stress reaction NOS | E28z.00 | Anxiety_spectrum |
| Examination fear | E28z.11 | Anxiety_spectrum |
| Flying phobia | E28z.12 | Anxiety_spectrum |
| Stage fright | E28z.13 | Anxiety_spectrum |
| Adjustment reaction | E29..00 | Anxiety_spectrum |
| Grief reaction | E290000 | Anxiety_spectrum |
| Bereavement reaction | E290011 | Anxiety_spectrum |
| Adjustment reaction; predominant disturbance other emotions | E292.00 | Anxiety_spectrum |
| separation anxiety disorder | E292000 | Anxiety_spectrum |
| Adolescent emancipation disorder | E292100 | Anxiety_spectrum |
| Early adult emancipation disorder | E292200 | Anxiety_spectrum |
| Specific academic or work inhibition | E292300 | Anxiety_spectrum |
| Specific academic or work inhibition | E292311 | Anxiety_spectrum |
| Specific work inhibition | E292312 | Anxiety_spectrum |
| Adjustment reaction with anxious mood | E292400 | Anxiety_spectrum |
| Culture shock | E292500 | Anxiety_spectrum |
| Adjustment reaction with mixed disturbance of emotion | E292y00 | Anxiety_spectrum |
| Adjustment reaction with disturbance of other emotion NOS | E292z00 | Anxiety_spectrum |
| Adjustment reaction with aggression | E293000 | Anxiety_spectrum |
| Adjustment reaction with antisocial behaviour | E293100 | Anxiety_spectrum |
| Adjustment reaction with destructiveness | E293200 | Anxiety_spectrum |
| Adjustment reaction with disturbance emotion and conduct | E294.00 | Anxiety_spectrum |
| Other adjustment reactions | E29y.00 | Anxiety_spectrum |
| Concentration camp syndrome | E29y000 | Anxiety_spectrum |
| Other post-traumatic stress disorder | E29y100 | Anxiety_spectrum |
| Adjustment reaction with physical symptoms | E29y200 | Anxiety_spectrum |
| Elective mutism due to an adjustment reaction | E29y300 | Anxiety_spectrum |
| Adjustment reaction due to hospitalisation | E29y400 | Anxiety_spectrum |
| Other adjustment reaction with withdrawal | E29y500 | Anxiety_spectrum |
| Other adjustment reactions NOS | E29yz00 | Anxiety_spectrum |
| Adjustment reaction NOS | E29z.00 | Anxiety_spectrum |
| disturbance of anxiety and fearfulness childhood/adolescent | E2D0.00 | Anxiety_spectrum |
| childhood and adolescent overanxiousness disturbance | E2D0000 | Anxiety_spectrum |
| disturbance anxiety and fearfulness childhood/adolescent nos | E2D0z00 | Anxiety_spectrum |
| Other specified neuroses or other mental disorders | E2y..00 | Anxiety_spectrum |
| Neuroses or other mental disorder NOS | E2z..00 | Anxiety_spectrum |
| [X]Organic anxiety disorder | Eu05400 | Anxiety_spectrum |
| [x]persistant anxiety depression | Eu34114 | Anxiety_spectrum |
| [X]Neurotic; stress - related and somoform disorders | Eu4..00 | Anxiety_spectrum |
| [x]phobic anxiety disorders | Eu40.00 | Anxiety_spectrum |
| [X]Agoraphobia | Eu40000 | Anxiety_spectrum |
| [X]Agoraphobia without history of panic disorder | Eu40011 | Anxiety_spectrum |
| [x]panic disorder with agoraphobia | Eu40012 | Anxiety_spectrum |
| [X]Social phobias | Eu40100 | Anxiety_spectrum |
| [X]Social neurosis | Eu40112 | Anxiety_spectrum |
| [X]Specific (isolated) phobias | Eu40200 | Anxiety_spectrum |
| [X]Acrophobia | Eu40211 | Anxiety_spectrum |
| [X]Animal phobias | Eu40212 | Anxiety_spectrum |
| [X]Claustrophobia | Eu40213 | Anxiety_spectrum |
| [X]Simple phobia | Eu40214 | Anxiety_spectrum |
| [x]other phobic anxiety disorders | Eu40y00 | Anxiety_spectrum |
| [x]phobic anxiety disorder; unspecified | Eu40z00 | Anxiety_spectrum |
| [X]Phobia NOS | Eu40z11 | Anxiety_spectrum |
| [X]Phobic state NOS | Eu40z12 | Anxiety_spectrum |
| [x]other anxiety disorders | Eu41.00 | Anxiety_spectrum |
| [x]panic disorder [episodic paroxysmal anxiety] | Eu41000 | Anxiety_spectrum |
| [x]panic attack | Eu41011 | Anxiety_spectrum |
| [x]panic state | Eu41012 | Anxiety_spectrum |
| [x]generalized anxiety disorder | Eu41100 | Anxiety_spectrum |
| [x]anxiety neurosis | Eu41111 | Anxiety_spectrum |
| [x]anxiety reaction | Eu41112 | Anxiety_spectrum |
| [x]anxiety state | Eu41113 | Anxiety_spectrum |
| [x]mixed anxiety and depressive disorder | Eu41200 | Anxiety_spectrum |
| [x]mild anxiety depression | Eu41211 | Anxiety_spectrum |
| [x]other mixed anxiety disorders | Eu41300 | Anxiety_spectrum |
| [x]other specified anxiety disorders | Eu41y00 | Anxiety_spectrum |
| [x]anxiety hysteria | Eu41y11 | Anxiety_spectrum |
| [x]anxiety disorder; unspecified | Eu41z00 | Anxiety_spectrum |
| [x]anxiety nos | Eu41z11 | Anxiety_spectrum |
| [X]Reaction to severe stress; and adjustment disorders | Eu43.00 | Anxiety_spectrum |
| [X]Acute stress reaction | Eu43000 | Anxiety_spectrum |
| [X]Acute crisis reaction | Eu43011 | Anxiety_spectrum |
| [X]Acute reaction to stress | Eu43012 | Anxiety_spectrum |
| [X]Combat fatigue | Eu43013 | Anxiety_spectrum |
| [X]Crisis state | Eu43014 | Anxiety_spectrum |
| [X]Psychic shock | Eu43015 | Anxiety_spectrum |
| [X]Post - traumatic stress disorder | Eu43100 | Anxiety_spectrum |
| [X]Traumatic neurosis | Eu43111 | Anxiety_spectrum |
| [X]Adjustment disorders | Eu43200 | Anxiety_spectrum |
| [X]Culture shock | Eu43211 | Anxiety_spectrum |
| [X]Grief reaction | Eu43212 | Anxiety_spectrum |
| [X]Hospitalism in children | Eu43213 | Anxiety_spectrum |
| [X]Acute post-traumatic stress disorder follow military comb | Eu43300 | Anxiety_spectrum |
| [X]Chron post-traumatic stress disorder follow military comb | Eu43400 | Anxiety_spectrum |
| [X]Other reactions to severe stress | Eu43y00 | Anxiety_spectrum |
| [X]Reaction to severe stress; unspecified | Eu43z00 | Anxiety_spectrum |
| [X]Dissociative [conversion] disorders | Eu44.00 | Anxiety_spectrum |
| [X]Dissociative amnesia | Eu44000 | Anxiety_spectrum |
| [X]Dissociative fugue | Eu44100 | Anxiety_spectrum |
| [X]Conversion hysteria | Eu44.11 | Anxiety_spectrum |
| [X]Conversion reaction | Eu44.12 | Anxiety_spectrum |
| [X]Hysteria | Eu44.13 | Anxiety_spectrum |
| [X]Dissociative stupor | Eu44200 | Anxiety_spectrum |
| [X]Trance and possession disorders | Eu44300 | Anxiety_spectrum |
| [X]Dissociative motor disorders | Eu44400 | Anxiety_spectrum |
| [X]Psychogenic aphonia | Eu44411 | Anxiety_spectrum |
| [X]Psychogenic dysphonia | Eu44412 | Anxiety_spectrum |
| [X]Dissociative convulsions | Eu44500 | Anxiety_spectrum |
| [X]Pseudoseizures | Eu44511 | Anxiety_spectrum |
| [X]Dissociative anaesthesia and sensory loss | Eu44600 | Anxiety_spectrum |
| [X]Psychogenic deafness | Eu44611 | Anxiety_spectrum |
| [X]Mixed dissociative [conversion] disorders | Eu44700 | Anxiety_spectrum |
| [X]Other dissociative [conversion] disorders | Eu44y00 | Anxiety_spectrum |
| [X]Ganser's syndrome | Eu44y11 | Anxiety_spectrum |
| [X]Multiple personality | Eu44y12 | Anxiety_spectrum |
| [X]Psychogenic confusion | Eu44y13 | Anxiety_spectrum |
| [X]Psychogenic twilight state | Eu44y14 | Anxiety_spectrum |
| [X]Dissociative [conversion] disorder; unspecified | Eu44z00 | Anxiety_spectrum |
| [X]Somatoform disorders | Eu45.00 | Anxiety_spectrum |
| [X]Somatization disorder | Eu45000 | Anxiety_spectrum |
| [X]Multiple psychosomatic disorder | Eu45011 | Anxiety_spectrum |
| [X]Briquet's syndrome | Eu45012 | Anxiety_spectrum |
| [X]Undifferentiated somatoform disorder | Eu45100 | Anxiety_spectrum |
| [X]Undifferentiated psychosomatic disorder | Eu45111 | Anxiety_spectrum |
| [X]Hypochondriacal disorder | Eu45200 | Anxiety_spectrum |
| [X]Body dysmorphic disorder | Eu45211 | Anxiety_spectrum |
| [X]Dysmorphophobia nondelusional | Eu45212 | Anxiety_spectrum |
| [X]Hypochondriacal neurosis | Eu45213 | Anxiety_spectrum |
| [X]Hypochondriasis | Eu45214 | Anxiety_spectrum |
| [X]Nosophobia | Eu45215 | Anxiety_spectrum |
| [X]Somatoform autonomic dysfunction | Eu45300 | Anxiety_spectrum |
| [X]Cardiac neurosis | Eu45311 | Anxiety_spectrum |
| [X]Da Costa's syndrome | Eu45312 | Anxiety_spectrum |
| [X]Gastric neurosis | Eu45313 | Anxiety_spectrum |
| [X]Neurocirculatory asthenia | Eu45314 | Anxiety_spectrum |
| [X]Psychogenic cough | Eu45316 | Anxiety_spectrum |
| [X]Psychogenic diarrhoea | Eu45317 | Anxiety_spectrum |
| [X]Psychogenic dyspepsia | Eu45318 | Anxiety_spectrum |
| [X]Psychogenic dysuria | Eu45319 | Anxiety_spectrum |
| [X]Psychogenic flatulence | Eu45320 | Anxiety_spectrum |
| [X]Psychogenic hiccough | Eu45321 | Anxiety_spectrum |
| [X]Psychogenic hyperventilat | Eu45322 | Anxiety_spectrum |
| [X]Psychogenic freq micturit | Eu45323 | Anxiety_spectrum |
| [X]Psychogenic IBS | Eu45324 | Anxiety_spectrum |
| [X]Psychogenic pylorospasm | Eu45325 | Anxiety_spectrum |
| [X]Persistent somatoform pain disorder | Eu45400 | Anxiety_spectrum |
| [X]Psychalgia | Eu45411 | Anxiety_spectrum |
| [X]Psychogenic backache | Eu45412 | Anxiety_spectrum |
| [X]Psychogenic headache | Eu45413 | Anxiety_spectrum |
| [X]Somatoform pain disorder | Eu45414 | Anxiety_spectrum |
| [X]Globus pharyngeus | Eu45500 | Anxiety_spectrum |
| [X]Globus hystericus | Eu45511 | Anxiety_spectrum |
| [X]Other somatoform disorders | Eu45y00 | Anxiety_spectrum |
| [X]Psychogenic dysmenorrhoea | Eu45y11 | Anxiety_spectrum |
| [X]Globus hystericus | Eu45y12 | Anxiety_spectrum |
| [X]Psychogenic pruritis | Eu45y13 | Anxiety_spectrum |
| [X]Psychogenic torticollis | Eu45y14 | Anxiety_spectrum |
| [X]Somatoform disorder; unspecified | Eu45z00 | Anxiety_spectrum |
| [X]Psychosomatic disorder NOS | Eu45z11 | Anxiety_spectrum |
| [X]Other neurotic disorders | Eu46.00 | Anxiety_spectrum |
| [X]Neurasthenia | Eu46000 | Anxiety_spectrum |
| [X]Fatigue syndrome | Eu46011 | Anxiety_spectrum |
| [X]Depersonalization - derealization syndrome | Eu46100 | Anxiety_spectrum |
| [X]Other specified neurotic disorders | Eu46y00 | Anxiety_spectrum |
| [X]Briquet's disorder | Eu46y11 | Anxiety_spectrum |
| [X]Dhat syndrome | Eu46y12 | Anxiety_spectrum |
| [X]Occupational neurosis; including writer's cramp | Eu46y13 | Anxiety_spectrum |
| [X]Psychasthenia | Eu46y14 | Anxiety_spectrum |
| [X]Psychasthenia neurosis | Eu46y15 | Anxiety_spectrum |
| [X]Psychogenic syncope | Eu46y16 | Anxiety_spectrum |
| [X]Neurotic disorder; unspecified | Eu46z00 | Anxiety_spectrum |
| [X]Neurosis NOS | Eu46z11 | Anxiety_spectrum |
| [X]Dream anxiety disorder | Eu51511 | Anxiety_spectrum |
| [x]anxious [avoidant] personality disorder | Eu60600 | Anxiety_spectrum |
| [x]separation anxiety disorder of childhood | Eu93000 | Anxiety_spectrum |
| [X]Phobic anxiety disorder of childhood | Eu93100 | Anxiety_spectrum |
| [X]Social anxiety disorder of childhood | Eu93200 | Anxiety_spectrum |
| [X]Avoidant disorder childhood | Eu93211 | Anxiety_spectrum |
| [x]childhood overanxious disorder | Eu93y12 | Anxiety_spectrum |
| anxiety counselling | Z4L1.00 | Anxiety_spectrum |
| Post-traumatic mutism | ZS7C700 | Anxiety_spectrum |
| [X]Obsessive-compulsive neurosis | Eu42.12 | Anxiety_spectrum |
| [X]Predominantly obsessional thoughts or ruminations | Eu42000 | Anxiety_spectrum |
| [X]Predominantly compulsive acts [obsessional rituals] | Eu42100 | Anxiety_spectrum |
| [X]Mixed obsessional thoughts and acts | Eu42200 | Anxiety_spectrum |
| [X]Other obsessive-compulsive disorders | Eu42y00 | Anxiety_spectrum |
| [X]Obsessive-compulsive disorder, unspecified | Eu42z00 | Anxiety_spectrum |
| [X]Obsessive - compulsive disorder | Eu42.00 | Anxiety_spectrum |
| [X]Obsessive-compulsive personality disorder | Eu60513 | Anxiety_spectrum |
| manic mood | 1S42.00 | BPAD |
| bipolar affective disorder resolved | 212V.00 | BPAD |
| Affective psychoses | E11..00 | BPAD |
| manic disorder; single episode | E110.00 | BPAD |
| single manic episode; unspecified | E110000 | BPAD |
| single manic episode; mild | E110100 | BPAD |
| hypomanic psychoses | E110.11 | BPAD |
| single manic episode; moderate | E110200 | BPAD |
| single manic episode; severe without mention of psychosis | E110300 | BPAD |
| single manic episode; severe; with psychosis | E110400 | BPAD |
| single manic episode in full remission | E110600 | BPAD |
| manic disorder; single episode nos | E110z00 | BPAD |
| recurrent manic episodes | E111.00 | BPAD |
| recurrent manic episodes; unspecified | E111000 | BPAD |
| bipolar psychoses | E11..11 | BPAD |
| recurrent manic episodes; mild | E111100 | BPAD |
| recurrent manic episodes; moderate | E111200 | BPAD |
| manic psychoses | E11..13 | BPAD |
| recurrent manic episodes; severe without mention psychosis | E111300 | BPAD |
| recurrent manic episodes; severe; with psychosis | E111400 | BPAD |
| recurrent manic episodes; partial or unspecified remission | E111500 | BPAD |
| recurrent manic episodes; in full remission | E111600 | BPAD |
| recurrent manic episode nos | E111z00 | BPAD |
| bipolar affective disorder; currently manic | E114.00 | BPAD |
| bipolar affective disorder; currently manic; unspecified | E114000 | BPAD |
| bipolar affective disorder; currently manic; mild | E114100 | BPAD |
| manic-depressive - now manic | E114.11 | BPAD |
| bipolar affective disorder; currently manic; moderate | E114200 | BPAD |
| bipolar affect disord; currently manic; severe; no psychosis | E114300 | BPAD |
| bipolar affect disord; currently manic;severe with psychosis | E114400 | BPAD |
| bipolar affect disord;currently manic; part/unspec remission | E114500 | BPAD |
| bipolar affective disorder; currently manic; full remission | E114600 | BPAD |
| bipolar affective disorder; currently manic; nos | E114z00 | BPAD |
| bipolar affective disorder; currently depressed | E115.00 | BPAD |
| bipolar affective disorder; currently depressed; unspecified | E115000 | BPAD |
| bipolar affective disorder; currently depressed; mild | E115100 | BPAD |
| manic-depressive - now depressed | E115.11 | BPAD |
| bipolar affective disorder; currently depressed; moderate | E115200 | BPAD |
| bipolar affect disord; now depressed; severe; no psychosis | E115300 | BPAD |
| bipolar affect disord; now depressed; severe with psychosis | E115400 | BPAD |
| bipolar affect disord; now depressed; part/unspec remission | E115500 | BPAD |
| bipolar affective disorder; now depressed; in full remission | E115600 | BPAD |
| bipolar affective disorder; currently depressed; nos | E115z00 | BPAD |
| mixed bipolar affective disorder | E116.00 | BPAD |
| mixed bipolar affective disorder; unspecified | E116000 | BPAD |
| mixed bipolar affective disorder; mild | E116100 | BPAD |
| mixed bipolar affective disorder; moderate | E116200 | BPAD |
| mixed bipolar affective disorder; severe; without psychosis | E116300 | BPAD |
| mixed bipolar affective disorder; severe; with psychosis | E116400 | BPAD |
| mixed bipolar affective disorder; partial/unspec remission | E116500 | BPAD |
| mixed bipolar affective disorder; in full remission | E116600 | BPAD |
| mixed bipolar affective disorder; nos | E116z00 | BPAD |
| unspecified bipolar affective disorder | E117.00 | BPAD |
| unspecified bipolar affective disorder; unspecified | E117000 | BPAD |
| unspecified bipolar affective disorder; mild | E117100 | BPAD |
| unspecified bipolar affective disorder; moderate | E117200 | BPAD |
| unspecified bipolar affective disorder; severe; no psychosis | E117300 | BPAD |
| unspecified bipolar affective disorder;severe with psychosis | E117400 | BPAD |
| unspecified bipolar affect disord; partial/unspec remission | E117500 | BPAD |
| unspecified bipolar affective disorder; in full remission | E117600 | BPAD |
| unspecified bipolar affective disorder; nos | E117z00 | BPAD |
| other and unspecified manic-depressive psychoses | E11y.00 | BPAD |
| unspecified manic-depressive psychoses | E11y000 | BPAD |
| atypical manic disorder | E11y100 | BPAD |
| other mixed manic-depressive psychoses | E11y300 | BPAD |
| other and unspecified manic-depressive psychoses nos | E11yz00 | BPAD |
| [X]Mood - affective disorders | Eu3..00 | BPAD |
| [x]manic episode | Eu30.00 | BPAD |
| [x]hypomania | Eu30000 | BPAD |
| [x]mania without psychotic symptoms | Eu30100 | BPAD |
| [x]bipolar disorder; single manic episode | Eu30.11 | BPAD |
| [x]mania with psychotic symptoms | Eu30200 | BPAD |
| [x]mania with mood-congruent psychotic symptoms | Eu30211 | BPAD |
| [x]mania with mood-incongruent psychotic symptoms | Eu30212 | BPAD |
| [x]other manic episodes | Eu30y00 | BPAD |
| [x]manic episode; unspecified | Eu30z00 | BPAD |
| [x]mania nos | Eu30z11 | BPAD |
| [x]bipolar affective disorder | Eu31.00 | BPAD |
| [x]bipolar affective disorder; current episode hypomanic | Eu31000 | BPAD |
| [x]bipolar affect disorder cur epi manic wout psychotic symp | Eu31100 | BPAD |
| [x]manic-depressive illness | Eu31.11 | BPAD |
| [x]manic-depressive psychosis | Eu31.12 | BPAD |
| [x]manic-depressive reaction | Eu31.13 | BPAD |
| [x]bipolar affect disorder cur epi manic with psychotic symp | Eu31200 | BPAD |
| [x]bipolar affect disorder cur epi mild or moderate depressn | Eu31300 | BPAD |
| [x]bipol aff disord; curr epis sev depress; no psychot symp | Eu31400 | BPAD |
| [x]bipolar affect dis cur epi severe depres with psyc symp | Eu31500 | BPAD |
| [x]bipolar affective disorder; current episode mixed | Eu31600 | BPAD |
| [x]bipolar affective disorder; currently in remission | Eu31700 | BPAD |
| [x]bipolar affective disorder type i | Eu31800 | BPAD |
| [x]bipolar affective disorder type ii | Eu31900 | BPAD |
| [x]bipolar ii disorder | Eu31911 | BPAD |
| [x]other bipolar affective disorders | Eu31y00 | BPAD |
| [x]bipolar ii disorder | Eu31y11 | BPAD |
| [x]recurrent manic episodes | Eu31y12 | BPAD |
| [x]bipolar affective disorder; unspecified | Eu31z00 | BPAD |
| [X]Persistent mood affective disorders | Eu34.00 | BPAD |
| [X]Other persistent mood affective disorders | Eu34y00 | BPAD |
| [X]Persistent mood affective disorder; unspecified | Eu34z00 | BPAD |
| [X]Mixed affective episode | Eu3y011 | BPAD |
| [X]Other recurrent mood affective disorders | Eu3y100 | BPAD |
| [X]Affective psychosis NOS | Eu3z.11 | BPAD |
| depressed | 1B17.00 | Depression |
| puerperal depression | 62T1.00 | Depression |
| on full dose long term treatment depression - enh serv admin | 9kQ..00 | Depression |
| presenile dementia with depression | E001300 | Depression |
| Senile dementia with depression | E002100 | Depression |
| Arteriosclerotic dementia with depression | E004300 | Depression |
| depressive psychoses | E11..12 | Depression |
| single major depressive episode | E112.00 | Depression |
| single major depressive episode; unspecified | E112000 | Depression |
| single major depressive episode; mild | E112100 | Depression |
| agitated depression | E112.11 | Depression |
| endogenous depression first episode | E112.12 | Depression |
| endogenous depression first episode | E112.13 | Depression |
| endogenous depression | E112.14 | Depression |
| single major depressive episode; moderate | E112200 | Depression |
| single major depressive episode; severe; without psychosis | E112300 | Depression |
| single major depressive episode; severe; with psychosis | E112400 | Depression |
| single major depressive episode nos | E112z00 | Depression |
| recurrent major depressive episode | E113.00 | Depression |
| recurrent major depressive episodes; unspecified | E113000 | Depression |
| recurrent major depressive episodes; mild | E113100 | Depression |
| endogenous depression - recurrent | E113.11 | Depression |
| recurrent major depressive episodes; moderate | E113200 | Depression |
| recurrent major depressive episodes; severe; no psychosis | E113300 | Depression |
| recurrent major depressive episodes; severe; with psychosis | E113400 | Depression |
| recurrent depression | E113700 | Depression |
| recurrent major depressive episode nos | E113z00 | Depression |
| Seasonal affective disorder | E118.00 | Depression |
| atypical depressive disorder | E11y200 | Depression |
| masked depression | E11z200 | Depression |
| reactive depressive psychosis | E130.00 | Depression |
| psychotic reactive depression | E130.11 | Depression |
| agitated depression | E135.00 | Depression |
| anxiety with depression | E200300 | Depression |
| neurotic depression reactive type | E204.00 | Depression |
| Postnatal depression | E204.11 | Depression |
| brief depressive reaction | E290.00 | Depression |
| brief depressive reaction nos | E290z00 | Depression |
| prolonged depressive reaction | E291.00 | Depression |
| depressive disorder nec | E2B..00 | Depression |
| postviral depression | E2B0.00 | Depression |
| chronic depression | E2B1.00 | Depression |
| [x]post-schizophrenic depression | Eu20400 | Depression |
| [x]depressive episode | Eu32.00 | Depression |
| [x]mild depressive episode | Eu32000 | Depression |
| [x]moderate depressive episode | Eu32100 | Depression |
| [x]single episode of depressive reaction | Eu32.11 | Depression |
| [x]single episode of psychogenic depression | Eu32.12 | Depression |
| [x]single episode of reactive depression | Eu32.13 | Depression |
| [x]severe depressive episode without psychotic symptoms | Eu32200 | Depression |
| [X]Single episode agitated depressn w'out psychotic symptoms | Eu32211 | Depression |
| [x]single episode major depression w'out psychotic symptoms | Eu32212 | Depression |
| [x]single episode vital depression w'out psychotic symptoms | Eu32213 | Depression |
| [x]severe depressive episode with psychotic symptoms | Eu32300 | Depression |
| [x]single episode of major depression and psychotic symptoms | Eu32311 | Depression |
| [x]single episode of psychogenic depressive psychosis | Eu32312 | Depression |
| [x]single episode of psychotic depression | Eu32313 | Depression |
| [x]single episode of reactive depressive psychosis | Eu32314 | Depression |
| [x]mild depression | Eu32400 | Depression |
| [x]major depression; mild | Eu32500 | Depression |
| [x]major depression; moderately severe | Eu32600 | Depression |
| [x]major depression; severe without psychotic symptoms | Eu32700 | Depression |
| [x]major depression; severe with psychotic symptoms | Eu32800 | Depression |
| [X]Single major depr ep; severe with psych; psych in remiss | Eu32900 | Depression |
| [X]Recurr major depr ep; severe with psych; psych in remiss | Eu32A00 | Depression |
| [x]other depressive episodes | Eu32y00 | Depression |
| [x]atypical depression | Eu32y11 | Depression |
| [x]single episode of masked depression nos | Eu32y12 | Depression |
| [x]depressive episode; unspecified | Eu32z00 | Depression |
| [x]depression nos | Eu32z11 | Depression |
| [x]depressive disorder nos | Eu32z12 | Depression |
| [x]prolonged single episode of reactive depression | Eu32z13 | Depression |
| [x] reactive depression nos | Eu32z14 | Depression |
| [x]recurrent depressive disorder | Eu33.00 | Depression |
| [x]recurrent depressive disorder; current episode mild | Eu33000 | Depression |
| [x]recurrent depressive disorder; current episode moderate | Eu33100 | Depression |
| [x]recurrent episodes of depressive reaction | Eu33.11 | Depression |
| [x]recurrent episodes of psychogenic depression | Eu33.12 | Depression |
| [x]recurrent episodes of reactive depression | Eu33.13 | Depression |
| [x]seasonal depressive disorder | Eu33.14 | Depression |
| [X]SAD - Seasonal affective disorder | Eu33.15 | Depression |
| [X]Recurr depress disorder cur epi severe without psyc sympt | Eu33200 | Depression |
| [x]endogenous depression without psychotic symptoms | Eu33211 | Depression |
| [x]major depression; recurrent without psychotic symptoms | Eu33212 | Depression |
| [x]vital depression; recurrent without psychotic symptoms | Eu33214 | Depression |
| [X]Recurrent depress disorder cur epi severe with psyc symp | Eu33300 | Depression |
| [x]endogenous depression with psychotic symptoms | Eu33311 | Depression |
| [x]manic-depress psychosis;depressed type+psychotic symptoms | Eu33312 | Depression |
| [x]recurr severe episodes/major depression+psychotic symptom | Eu33313 | Depression |
| [x]recurr severe episodes/psychogenic depressive psychosis | Eu33314 | Depression |
| [x]recurrent severe episodes of psychotic depression | Eu33315 | Depression |
| [x]recurrent severe episodes/reactive depressive psychosis | Eu33316 | Depression |
| [x]recurrent depressive disorder; currently in remission | Eu33400 | Depression |
| [x]other recurrent depressive disorders | Eu33y00 | Depression |
| [x]recurrent depressive disorder; unspecified | Eu33z00 | Depression |
| [x]monopolar depression nos | Eu33z11 | Depression |
| [x]dysthymia | Eu34100 | Depression |
| [x]depressive neurosis | Eu34111 | Depression |
| [x]neurotic depression | Eu34113 | Depression |
| [x]persistant anxiety depression | Eu34114 | Depression |
| [x]recurrent brief depressive episodes | Eu3y111 | Depression |
| [x]mixed anxiety and depressive disorder | Eu41200 | Depression |
| [x]mild anxiety depression | Eu41211 | Depression |
| [X]Postnatal depression NOS | Eu53011 | Depression |
| [X]Postpartum depression NOS | Eu53012 | Depression |
| [x]depressive conduct disorder | Eu92000 | Depression |
| Intentional overdose of prescription only medication | 14K1.00 | Suicide_or_harm |
| Overdose of biological substance | SL...14 | Suicide_or_harm |
| Overdose of drug | SL...15 | Suicide_or_harm |
| Antidepressant poisoning | SL90.00 | Suicide_or_harm |
| Anti-depressant poisoning NOS | SL90z00 | Suicide_or_harm |
| Drug and medicament poisoning NOS | SLHz.00 | Suicide_or_harm |
| Suicide and selfinflicted injury | TK...00 | Suicide_or_harm |
| Suicide + selfinflicted poisoning by solid/liquid substances | TK0..00 | Suicide_or_harm |
| Suicide + selfinflicted poisoning by analgesic/antipyretic | TK00.00 | Suicide_or_harm |
| Suicide + selfinflicted poisoning by barbiturates | TK01.00 | Suicide_or_harm |
| Suicide and self inflicted injury by Amylobarbitone | TK01000 | Suicide_or_harm |
| Suicide and self inflicted injury by Barbitone | TK01100 | Suicide_or_harm |
| Suicide and self inflicted injury by Phenobarbitone | TK01400 | Suicide_or_harm |
| Suicide + selfinflicted poisoning by oth sedatives/hypnotics | TK02.00 | Suicide_or_harm |
| Suicide + selfinflicted poisoning tranquilliser/psychotropic | TK03.00 | Suicide_or_harm |
| Suicide + selfinflicted poisoning by other drugs/medicines | TK04.00 | Suicide_or_harm |
| Suicide + selfinflicted poisoning by drug or medicine NOS | TK05.00 | Suicide_or_harm |
| Suicide + selfinflicted poisoning by agricultural chemical | TK06.00 | Suicide_or_harm |
| Suicide + selfinflicted poisoning by corrosive/caustic subst | TK07.00 | Suicide_or_harm |
| Suicide + selfinflicted poisoning by solid/liquid subst NOS | TK0z.00 | Suicide_or_harm |
| Suicide + selfinflicted poisoning by gases in domestic use | TK1..00 | Suicide_or_harm |
| Suicide + selfinflicted poisoning by gas via pipeline | TK10.00 | Suicide_or_harm |
| Cause of overdose - deliberate | TK...11 | Suicide_or_harm |
| Suicide + selfinflicted poisoning by liquified petrol gas | TK11.00 | Suicide_or_harm |
| Injury - self-inflicted | TK...12 | Suicide_or_harm |
| Poisoning - self-inflicted | TK...13 | Suicide_or_harm |
| Suicide and self harm | TK...14 | Suicide_or_harm |
| Attempted suicide | TK...15 | Suicide_or_harm |
| Para-suicide | TK...17 | Suicide_or_harm |
| Suicide and selfinflicted poisoning by other utility gas | TK1y.00 | Suicide_or_harm |
| Suicide + selfinflicted poisoning by domestic gases NOS | TK1z.00 | Suicide_or_harm |
| Suicide + selfinflicted poisoning by other gases and vapours | TK2..00 | Suicide_or_harm |
| Suicide + selfinflicted poisoning by motor veh exhaust gas | TK20.00 | Suicide_or_harm |
| Suicide and selfinflicted poisoning by other carbon monoxide | TK21.00 | Suicide_or_harm |
| Suicide + selfinflicted poisoning by gases and vapours NOS | TK2z.00 | Suicide_or_harm |
| Suicide + selfinflicted injury by hang/strangulate/suffocate | TK3..00 | Suicide_or_harm |
| Suicide and selfinflicted injury by hanging | TK30.00 | Suicide_or_harm |
| Suicide + selfinflicted injury by suffocation by plastic bag | TK31.00 | Suicide_or_harm |
| Suicide + selfinflicted inj oth mean hang/strangle/suffocate | TK3y.00 | Suicide_or_harm |
| Suicide + selfinflicted inj by hang/strangle/suffocate NOS | TK3z.00 | Suicide_or_harm |
| Suicide and selfinflicted injury by drowning | TK4..00 | Suicide_or_harm |
| Suicide and selfinflicted injury by firearms and explosives | TK5..00 | Suicide_or_harm |
| Suicide and selfinflicted injury by shotgun | TK51.00 | Suicide_or_harm |
| Suicide and selfinflicted injury by hunting rifle | TK52.00 | Suicide_or_harm |
| Suicide and selfinflicted injury by other firearm | TK54.00 | Suicide_or_harm |
| Suicide and selfinflicted injury by firearms/explosives NOS | TK5z.00 | Suicide_or_harm |
| Suicide and selfinflicted injury by cutting and stabbing | TK6..00 | Suicide_or_harm |
| Suicide and selfinflicted injury by cutting | TK60.00 | Suicide_or_harm |
| Self inflicted lacerations to wrist | TK60100 | Suicide_or_harm |
| Slashed wrists self inflicted | TK60111 | Suicide_or_harm |
| Suicide and selfinflicted injury by stabbing | TK61.00 | Suicide_or_harm |
| Suicide and selfinflicted injury by cutting and stabbing NOS | TK6z.00 | Suicide_or_harm |
| Suicide and selfinflicted injury by jumping from high place | TK7..00 | Suicide_or_harm |
| Suicide+selfinflicted injury-jump from residential premises | TK70.00 | Suicide_or_harm |
| Suicide+selfinflicted injury-jump from oth manmade structure | TK71.00 | Suicide_or_harm |
| Suicide+selfinflicted injury-jump from natural sites | TK72.00 | Suicide_or_harm |
| Suicide+selfinflicted injury-jump from high place NOS | TK7z.00 | Suicide_or_harm |
| Suicide and selfinflicted injury by other means | TKx..00 | Suicide_or_harm |
| Suicide + selfinflicted injury-jump/lie before moving object | TKx0.00 | Suicide_or_harm |
| Suicide + selfinflicted injury-jumping before moving object | TKx0000 | Suicide_or_harm |
| Suicide and selfinflicted injury by burns or fire | TKx1.00 | Suicide_or_harm |
| Suicide and selfinflicted injury by scald | TKx2.00 | Suicide_or_harm |
| Suicide and selfinflicted injury by extremes of cold | TKx3.00 | Suicide_or_harm |
| Suicide and selfinflicted injury by electrocution | TKx4.00 | Suicide_or_harm |
| Suicide and selfinflicted injury by crashing motor vehicle | TKx5.00 | Suicide_or_harm |
| Suicide and selfinflicted injury by crashing of aircraft | TKx6.00 | Suicide_or_harm |
| Suicide and selfinflicted injury caustic subst; excl poison | TKx7.00 | Suicide_or_harm |
| Suicide and selfinflicted injury by other specified means | TKxy.00 | Suicide_or_harm |
| Suicide and selfinflicted injury by other means NOS | TKxz.00 | Suicide_or_harm |
| Late effects of selfinflicted injury | TKy..00 | Suicide_or_harm |
| Suicide and selfinflicted injury NOS | TKz..00 | Suicide_or_harm |
| [X]Intentional self-harm | U2...00 | Suicide_or_harm |
| [X]Intentional self poisoning/exposure to noxious substances | U20..00 | Suicide_or_harm |
| [X]Intent self poison/exposure to nonopioid analgesic | U200.00 | Suicide_or_harm |
| [X]Int self poison/exposure to nonopioid analgesic at home | U200000 | Suicide_or_harm |
| [X]Intent self poison nonopioid analgesic at res institut | U200100 | Suicide_or_harm |
| [X]Overdose - paracetamol | U200.11 | Suicide_or_harm |
| [X]Overdose - ibuprofen | U200.12 | Suicide_or_harm |
| [X]Overdose - aspirin | U200.13 | Suicide_or_harm |
| [X]Intent self pois nonopioid analgesic in street/highway | U200400 | Suicide_or_harm |
| [X]Intent self pois nonopioid analgesic trade/service area | U200500 | Suicide_or_harm |
| [X]Int self poison nonopioid analgesic other spec place | U200y00 | Suicide_or_harm |
| [X]Intent self poison nonopioid analgesic unspecif place | U200z00 | Suicide_or_harm |
| [X]Intent self poison/exposure to antiepileptic | U201.00 | Suicide_or_harm |
| [X]Int self poison/exposure to antiepileptic at home | U201000 | Suicide_or_harm |
| [X]Deliberate drug overdose / other poisoning | U20..11 | Suicide_or_harm |
| [X]Intent self poison antiepileptic unspecif place | U201z00 | Suicide_or_harm |
| [X]Intent self poison/exposure to sedative hypnotic | U202.00 | Suicide_or_harm |
| [X]Int self poison/exposure to sedative hypnotic at home | U202000 | Suicide_or_harm |
| [X]Overdose - sleeping tabs | U202.11 | Suicide_or_harm |
| [X]Overdose - diazepam | U202.12 | Suicide_or_harm |
| [X]Overdose - temazepam | U202.13 | Suicide_or_harm |
| [X]Overdose - nitrazepam | U202.15 | Suicide_or_harm |
| [X]Overdose - benzodiazepine | U202.16 | Suicide_or_harm |
| [X]Overdose - barbiturate | U202.17 | Suicide_or_harm |
| [X]Overdose - amobarbital | U202.18 | Suicide_or_harm |
| [X]Intent self pois sedative hypnotic in street/highway | U202400 | Suicide_or_harm |
| [X]Int self poison sedative hypnotic other spec place | U202y00 | Suicide_or_harm |
| [X]Intent self poison sedative hypnotic unspecif place | U202z00 | Suicide_or_harm |
| [X]Intent self poison/exposure to psychotropic drug | U204.00 | Suicide_or_harm |
| [X]Int self poison/exposure to psychotropic drug at home | U204000 | Suicide_or_harm |
| [X]Intent self poison psychotropic drug at res institut | U204100 | Suicide_or_harm |
| [X]Overdose - antidepressant | U204.11 | Suicide_or_harm |
| [X]Overdose - amitriptyline | U204.12 | Suicide_or_harm |
| [X]Overdose - SSRI | U204.13 | Suicide_or_harm |
| [X]Int self poison psychotropic drug other spec place | U204y00 | Suicide_or_harm |
| [X]Intent self poison psychotropic drug unspecif place | U204z00 | Suicide_or_harm |
| [X]Intent self poison/exposure to narcotic drug | U205.00 | Suicide_or_harm |
| [X]Int self poison/exposure to narcotic drug at home | U205000 | Suicide_or_harm |
| [X]Int self poison narcotic drug other spec place | U205y00 | Suicide_or_harm |
| [X]Intent self poison narcotic drug unspecif place | U205z00 | Suicide_or_harm |
| [X]Intent self poison/exposure to hallucinogen | U206.00 | Suicide_or_harm |
| [X]Intent self pois hallucinogen in street/highway | U206400 | Suicide_or_harm |
| [X]Intent self poison/exposure to oth autonomic drug | U207.00 | Suicide_or_harm |
| [X]Int self poison/exposure to oth autonomic drug at home | U207000 | Suicide_or_harm |
| [X]Intent self poison oth autonomic drug unspecif place | U207z00 | Suicide_or_harm |
| [X]Int self poison/exposure to other/unspec drug/medicament | U208.00 | Suicide_or_harm |
| [X]Int self poison/exposure to oth/unsp drug/medicam home | U208000 | Suicide_or_harm |
| [X]Intent self pois oth/unsp drug/medic in street/highway | U208400 | Suicide_or_harm |
| [X]Int self poison oth/unsp drug/medic other spec place | U208y00 | Suicide_or_harm |
| [X]Intent self poison oth/unsp drug/medic unspecif place | U208z00 | Suicide_or_harm |
| [X]Intent self poison/exposure to alcohol | U209.00 | Suicide_or_harm |
| [X]Int self poison alcohol other spec place | U209y00 | Suicide_or_harm |
| [X]Intent self poison alcohol unspecif place | U209z00 | Suicide_or_harm |
| [X]Intentional self poison organ solvent;halogen hydrocarb | U20A.00 | Suicide_or_harm |
| [X]Intent self pois organ solvent;halogen hydrocarb; home | U20A000 | Suicide_or_harm |
| [X]Self poisoning from glue solvent | U20A.11 | Suicide_or_harm |
| [X]Int self poison org solvent;halogen hydrocarb;in highway | U20A400 | Suicide_or_harm |
| [X]Int self pois org solv;halogen hydrocarb; unspec place | U20Az00 | Suicide_or_harm |
| [X]Intent self poison/exposure to other gas/vapour | U20B.00 | Suicide_or_harm |
| [X]Int self poison/exposure to other gas/vapour at home | U20B000 | Suicide_or_harm |
| [X]Self carbon monoxide poisoning | U20B.11 | Suicide_or_harm |
| [X]Int self poison other gas/vapour school/pub admin area | U20B200 | Suicide_or_harm |
| [X]Int self poison other gas/vapour other spec place | U20By00 | Suicide_or_harm |
| [X]Intent self poison other gas/vapour unspecif place | U20Bz00 | Suicide_or_harm |
| [X]Intent self poison/exposure to pesticide | U20C.00 | Suicide_or_harm |
| [X]Int self poison/exposure to pesticide at home | U20C000 | Suicide_or_harm |
| [X]Self poisoning with weedkiller | U20C.11 | Suicide_or_harm |
| [X]Self poisoning with paraquat | U20C.12 | Suicide_or_harm |
| [X]Int self poison pesticide other spec place | U20Cy00 | Suicide_or_harm |
| [X]Intent self poison/exposure to unspecif chemical | U20y.00 | Suicide_or_harm |
| [X]Int self poison/exposure to unspecif chemical at home | U20y000 | Suicide_or_harm |
| [X]Int self poison unspecif chemical school/pub admin area | U20y200 | Suicide_or_harm |
| [X]Intent self poison unspecif chemical unspecif place | U20yz00 | Suicide_or_harm |
| [X]Intent self harm by hanging strangulation / suffocation | U21..00 | Suicide_or_harm |
| [X]Intent self harm by hanging strangulat/suffocat occ home | U210.00 | Suicide_or_harm |
| [X]Self inflicted injury | U2...11 | Suicide_or_harm |
| [X]Intent self harm by hangng strangult/suffoct resid instit | U211.00 | Suicide_or_harm |
| [X]Injury - self-inflicted | U2...12 | Suicide_or_harm |
| [X]Inten slf harm hang strang/suffc sch oth ins/pub adm area | U212.00 | Suicide_or_harm |
| [X]Suicide | U2...13 | Suicide_or_harm |
| [X]Attempted suicide | U2...14 | Suicide_or_harm |
| [X]Para-suicide | U2...15 | Suicide_or_harm |
| [X]Intent self harm by hang strangl/suffc indust/constr area | U216.00 | Suicide_or_harm |
| [X]Intent self harm by hangng strangul/suffoct oth spec plce | U21y.00 | Suicide_or_harm |
| [X]Intent self harm by hangng strangul/suffoct unspecif plce | U21z.00 | Suicide_or_harm |
| [X]Intentional self harm by drowning and submersion | U22..00 | Suicide_or_harm |
| [X]Intent self harm by drowning/submersion occurrn at home | U220.00 | Suicide_or_harm |
| [X]Intent self harm by drowning/submersn occ resid instit'n | U221.00 | Suicide_or_harm |
| [X]Intent self harm by drown/submersn occ oth specif place | U22y.00 | Suicide_or_harm |
| [X]Intent self harm by drown/submersn occ unspecified place | U22z.00 | Suicide_or_harm |
| [X]Intent self harm by rifle shotgun/larger firearm disch | U24..00 | Suicide_or_harm |
| [X]Int self harm rifl s'gun/lrg frarm disch occ resid instit | U241.00 | Suicide_or_harm |
| [X]Int slf hrm rifl s'gun/lrg frarm dis sch/ins/pub adm area | U242.00 | Suicide_or_harm |
| [X]Intent self harm by other/unspecified firearm discharge | U25..00 | Suicide_or_harm |
| [X]Intent self harm oth/unspecif firearm disch occ at home | U250.00 | Suicide_or_harm |
| [X]Intentional self harm by explosive material | U26..00 | Suicide_or_harm |
| [X]Intentional self harm by smoke; fire and flames | U27..00 | Suicide_or_harm |
| [X]Intention self harm by smoke fire/flames occurrn at home | U270.00 | Suicide_or_harm |
| [X]Intent self harm by smoke fire/flame occ street/highway | U274.00 | Suicide_or_harm |
| [X]Intent self harm by smoke fire/flames occ unspecif place | U27z.00 | Suicide_or_harm |
| [X]Intentional self harm by steam hot vapours / hot objects | U28..00 | Suicide_or_harm |
| [X]Intent self harm by steam hot vapour/hot obj occ at home | U280.00 | Suicide_or_harm |
| [X]Intent self harm by steam hot vapour/obj occ unspec place | U28z.00 | Suicide_or_harm |
| [X]Intentional self harm by sharp object | U29..00 | Suicide_or_harm |
| [X]Intentional self harm by sharp object occurrence at home | U290.00 | Suicide_or_harm |
| [X]Intent self harm by sharp object occ resident instit'n | U291.00 | Suicide_or_harm |
| [X]Intention self harm by sharp object occ street/highway | U294.00 | Suicide_or_harm |
| [X]Intention self harm by sharp object occ oth specif place | U29y.00 | Suicide_or_harm |
| [X]Intentional self harm by sharp object occ unspecif place | U29z.00 | Suicide_or_harm |
| [X]Intentional self harm by blunt object | U2A..00 | Suicide_or_harm |
| [X]Intentional self harm by blunt object occurrence at home | U2A0.00 | Suicide_or_harm |
| [X]Intent self harm by blunt object occ resident instit'n | U2A1.00 | Suicide_or_harm |
| [X]Intent self harm by blunt object occ sports/athlet area | U2A3.00 | Suicide_or_harm |
| [X]Intentional self harm by jumping from a high place | U2B..00 | Suicide_or_harm |
| [X]Intent self harm by jumping from high place occ at home | U2B0.00 | Suicide_or_harm |
| [X]Intent self harm by jump from high place occ street/h'way | U2B4.00 | Suicide_or_harm |
| [X]Int self harm by jump from high place indust/constr area | U2B6.00 | Suicide_or_harm |
| [X]Int self harm by jump from high place occ oth specif plce | U2By.00 | Suicide_or_harm |
| [X]Int self harm by jump from high place occ unspecif place | U2Bz.00 | Suicide_or_harm |
| [X]Intent self harm by jumping / lying before moving object | U2C..00 | Suicide_or_harm |
| [X]Int self harm jump/lying befr mov obje occ resid instit'n | U2C1.00 | Suicide_or_harm |
| [X]Int self harm jump/lying befr mov obje occ street/highway | U2C4.00 | Suicide_or_harm |
| [X]Int self harm jump/lying bef mov obje occ oth specif plce | U2Cy.00 | Suicide_or_harm |
| [X]Intentional self harm by crashing of motor vehicle | U2D..00 | Suicide_or_harm |
| [X]Intent self harm by crash of motor vehicl occurrn at home | U2D0.00 | Suicide_or_harm |
| [X]Intent self harm by crash motor vehicl occ street/highway | U2D4.00 | Suicide_or_harm |
| [X]Intent self harm crash motor vehic occ indust/constr area | U2D6.00 | Suicide_or_harm |
| [X]Self mutilation | U2E..00 | Suicide_or_harm |
| [X]Intentional self harm by other specified means | U2y..00 | Suicide_or_harm |
| [X]Intentionl self harm by oth specif means occurrn at home | U2y0.00 | Suicide_or_harm |
| [X]Intent self harm by oth specif means occ resid instit'n | U2y1.00 | Suicide_or_harm |
| [X]Intent self harm by oth specif means occ unspecif place | U2yz.00 | Suicide_or_harm |
| [X]Intentional self harm by unspecified means | U2z..00 | Suicide_or_harm |
| [X]Intentional self harm by unspecif means occurrn at home | U2z0.00 | Suicide_or_harm |
| [X]Intent self harm by unspec mean occ sch/ins/pub adm area | U2z2.00 | Suicide_or_harm |
| [X]Intent self harm by unspecif means occ oth specif place | U2zy.00 | Suicide_or_harm |
| [X]Intent self harm by unspecif means occ at unspecif place | U2zz.00 | Suicide_or_harm |
| [X]Deliberate drug poisoning | U30..11 | Suicide_or_harm |
| [X]Hanging strangulation + suffocation undetermined intent | U41..00 | Suicide_or_harm |
| [X]Rifle shotgun+larger firearm discharge undetermin intent | U44..00 | Suicide_or_harm |
| [X]Other+unspecified firearm discharge undetermined intent | U45..00 | Suicide_or_harm |
| [X]Falling jumping/pushed from high place undeterm intent | U4B..00 | Suicide_or_harm |
| [X]Fall jump/push frm high plce undt intnt occ unspecif plce | U4Bz.00 | Suicide_or_harm |
| [X]Sequel intentn self-harm assault+event of undeterm intent | U72..00 | Suicide_or_harm |
| [X]Sequelae of intentional self-harm | U720.00 | Suicide_or_harm |
| HoNOS item 2 - non-accidental self injury | ZRLfC12 | Suicide_or_harm |
| Suicide intent score subscale - attempt circumstances | ZRn3.00 | Suicide_or_harm |
| Self-harm | ZX...00 | Suicide_or_harm |
| Self-injurious behaviour | ZX1..00 | Suicide_or_harm |
| Self-damage | ZX...11 | Suicide_or_harm |
| Biting self | ZX11.00 | Suicide_or_harm |
| Bites self | ZX11.11 | Suicide_or_harm |
| SIB - Self-injurious behaviour | ZX1..12 | Suicide_or_harm |
| Deliberate self-harm | ZX1..13 | Suicide_or_harm |
| Burning self | ZX12.00 | Suicide_or_harm |
| Cutting self | ZX13.00 | Suicide_or_harm |
| Cutting own wrists | ZX13100 | Suicide_or_harm |
| Cuts self | ZX13.11 | Suicide_or_harm |
| Drowning self | ZX15.00 | Suicide_or_harm |
| Hanging self | ZX18.00 | Suicide_or_harm |
| Hitting self | ZX19.00 | Suicide_or_harm |
| Punching self | ZX19100 | Suicide_or_harm |
| Slapping self | ZX19200 | Suicide_or_harm |
| Jumping from height | ZX1B.00 | Suicide_or_harm |
| Jumping from building | ZX1B100 | Suicide_or_harm |
| Jumping from bridge | ZX1B200 | Suicide_or_harm |
| Jumping from cliff | ZX1B300 | Suicide_or_harm |
| Nipping self | ZX1C.00 | Suicide_or_harm |
| Pinching self | ZX1E.00 | Suicide_or_harm |
| Scratches self | ZX1G.00 | Suicide_or_harm |
| Self-asphyxiation | ZX1H.00 | Suicide_or_harm |
| Self-strangulation | ZX1H100 | Suicide_or_harm |
| Self-suffocation | ZX1H200 | Suicide_or_harm |
| Self-scalding | ZX1I.00 | Suicide_or_harm |
| Self-electrocution | ZX1J.00 | Suicide_or_harm |
| Self-incineration | ZX1K.00 | Suicide_or_harm |
| Setting fire to self | ZX1K.11 | Suicide_or_harm |
| Setting self alight | ZX1K.12 | Suicide_or_harm |
| Self-mutilation | ZX1L.00 | Suicide_or_harm |
| Self-mutilation of hands | ZX1L100 | Suicide_or_harm |
| Self-mutilation of genitalia | ZX1L200 | Suicide_or_harm |
| Self-mutilation of penis | ZX1L300 | Suicide_or_harm |
| Self-mutilation of ears | ZX1L600 | Suicide_or_harm |
| [X]Self mutilation | ZX1LD00 | Suicide_or_harm |
| Shooting self | ZX1M.00 | Suicide_or_harm |
| Stabbing self | ZX1N.00 | Suicide_or_harm |
| Throwing self in front of train | ZX1Q.00 | Suicide_or_harm |
| Jumping under train | ZX1Q.11 | Suicide_or_harm |
| Throwing self in front of vehicle | ZX1R.00 | Suicide_or_harm |
| Throwing self onto floor | ZX1S.00 | Suicide_or_harm |
| Referral to eating disorders clinic | 8HTN.00 | Eating_disorder |
| Seen in eating disorder clinic | 9Nk9.00 | Eating_disorder |
| Anorexia nervosa | E271.00 | Eating_disorder |
| Other and unspecified non-organic eating disorders | E275.00 | Eating_disorder |
| Unspecified non-organic eating disorder | E275000 | Eating_disorder |
| Bulimia (non-organic overeating) | E275100 | Eating_disorder |
| Compulsive eating disorder | E275111 | Eating_disorder |
| Other specified non-organic eating disorder | E275y00 | Eating_disorder |
| Non-organic eating disorder NOS | E275z00 | Eating_disorder |
| [X]Eating disorders | Eu50.00 | Eating_disorder |
| [X]Anorexia nervosa | Eu50000 | Eating_disorder |
| [X]Atypical anorexia nervosa | Eu50100 | Eating_disorder |
| [X]Bulimia nervosa | Eu50200 | Eating_disorder |
| [X]Bulimia NOS | Eu50211 | Eating_disorder |
| [X]Hyperorexia nervosa | Eu50212 | Eating_disorder |
| [X]Atypical bulimia nervosa | Eu50300 | Eating_disorder |
| [X]Overeating associated with other psychological disturbncs | Eu50400 | Eating_disorder |
| [X]Psychogenic overeating | Eu50411 | Eating_disorder |
| [X]Other eating disorders | Eu50y00 | Eating_disorder |
| [X]Pica in adults | Eu50y11 | Eating_disorder |
| [X]Eating disorder; unspecified | Eu50z00 | Eating_disorder |
| Nocturnal sleep-related eating disorder | Fy05.00 | Eating_disorder |
| [D]Anorexia | R030.00 | Eating_disorder |
| [D]Anorexia NOS | R030z00 | Eating_disorder |
| [D]Polyphagia | R036.00 | Eating_disorder |
| [D]Excessive eating | R036000 | Eating_disorder |
| [D]Bulimia NOS | R036011 | Eating_disorder |
| [D]Hyperalimentation | R036100 | Eating_disorder |
| [D]Polyphagia NOS | R036z00 | Eating_disorder |
| Starvation | SN42100 | Eating_disorder |
| [X]Starvation | U1B3.11 | Eating_disorder |
| Eating disorder counselling | Z4B5.00 | Eating_disorder |
| Dietary advice for eating disorder | ZC2CD00 | Eating_disorder |
| Child refer- clinical psychol. | 64R7.11 | MHS_referral |
| Admit psychiatric emergency | 8H23.00 | MHS_referral |
| Emerg psychiatric admiss MHA | 8H23000 | MHS_referral |
| Admit psychogeriatric emergency | 8H2L.00 | MHS_referral |
| Emergency voluntary psychiatric admission Mental Health Act | 8H2T.00 | MHS_referral |
| Non-urgent psychiatric admisn. | 8H38.00 | MHS_referral |
| Non-urgent psychogeriatric admission | 8H3Q.00 | MHS_referral |
| Psychiatric referral | 8H49.00 | MHS_referral |
| Referral to psychogeriatrician | 8H4D.00 | MHS_referral |
| Referral to learning disabilities psychiatrist | 8H4f.00 | MHS_referral |
| Referral to child psychiatrist | 8H4P.00 | MHS_referral |
| Refer to psychologist | 8H7T.00 | MHS_referral |
| Referral to mental health team | 8Hc..00 | MHS_referral |
| Referral to community mental health team | 8Hc0.00 | MHS_referral |
| Referral to primary care mental health team | 8Hc2.00 | MHS_referral |
| Referral to non NHS mental health community service | 8HHn.00 | MHS_referral |
| Referral to older age communiy mental health team | 8HHo.00 | MHS_referral |
| Referral to child and adolescent psychiatry service | 8HHR.00 | MHS_referral |
| Referral to psychosis early intervention service | 8HHs.00 | MHS_referral |
| Referral to psychotherapist | 8HHT.00 | MHS_referral |
| Referral to primary care mental health gateway worker | 8HHu.00 | MHS_referral |
| Referral to primary care mental health graduate worker | 8HHv.00 | MHS_referral |
| Psychiatric self-referral | 8HJ3.00 | MHS_referral |
| Urgent referral to psychiatrist | 8HlB.00 | MHS_referral |
| Referral to forensic psychiatrist | 8HlD.00 | MHS_referral |
| Private referral to psychologist | 8HVi.00 | MHS_referral |
| Private referral to psychiatrist | 8HVO.00 | MHS_referral |
| Private referral to psychogeriatrician | 8HVS.00 | MHS_referral |
| Referral to psychiatrist | ZL5B.00 | MHS_referral |
| Referral to child and adolescent psychiatrist | ZL5B100 | MHS_referral |
| Referral to child psychiatrist | ZL5B111 | MHS_referral |
| Referral to forensic psychiatrist | ZL5B200 | MHS_referral |
| Referral to liaison psychiatrist | ZL5B300 | MHS_referral |
| Referral to rehabilitation psychiatrist | ZL5B400 | MHS_referral |
| Referral to psychiatrist for mental handicap | ZL5B500 | MHS_referral |
| Referral to nurse behavioural therapist | ZL62A00 | MHS_referral |
| Referral to nurse psychotherapist | ZL62B00 | MHS_referral |
| Referral to psychiatric nurse | ZL62E00 | MHS_referral |
| Referral to psychotherapist | ZL77.00 | MHS_referral |
| Referral to psychologist | ZL78.00 | MHS_referral |
| Refer to psychologist | ZL78.11 | MHS_referral |
| Personality disorders | E21..00 | Pesonality_disorder |
| Paranoid personality disorder | E210.00 | Pesonality_disorder |
| Affective personality disorder | E211.00 | Pesonality_disorder |
| Unspecified affective personality disorder | E211000 | Pesonality_disorder |
| Neurotic personality disorder | E21..11 | Pesonality_disorder |
| hypomanic personality disorder | E211100 | Pesonality_disorder |
| depressive personality disorder | E211200 | Pesonality_disorder |
| cyclothymic personality disorder | E211300 | Pesonality_disorder |
| Affective personality disorder NOS | E211z00 | Pesonality_disorder |
| schizoid personality disorder | E212.00 | Pesonality_disorder |
| unspecified schizoid personality disorder | E212000 | Pesonality_disorder |
| schizoid personality disorder nos | E212z00 | Pesonality_disorder |
| Explosive personality disorder | E213.00 | Pesonality_disorder |
| Compulsive personality disorders | E214.00 | Pesonality_disorder |
| Anankastic personality | E214000 | Pesonality_disorder |
| Anancastic personality | E214.11 | Pesonality_disorder |
| Compulsive personality disorder NOS | E214z00 | Pesonality_disorder |
| Histrionic personality disorders | E215.00 | Pesonality_disorder |
| Unspecified histrionic personality disorder | E215000 | Pesonality_disorder |
| Hysterical personality disorders | E215.11 | Pesonality_disorder |
| Histrionic personality disorder NOS | E215z00 | Pesonality_disorder |
| Inadequate personality disorder | E216.00 | Pesonality_disorder |
| Antisocial or sociopathic personality disorder | E217.00 | Pesonality_disorder |
| Other personality disorders | E21y.00 | Pesonality_disorder |
| Narcissistic personality disorder | E21y000 | Pesonality_disorder |
| Avoidant personality disorder | E21y100 | Pesonality_disorder |
| Borderline personality disorder | E21y200 | Pesonality_disorder |
| Passive-aggressive personality disorder | E21y300 | Pesonality_disorder |
| Eccentric personality disorder | E21y400 | Pesonality_disorder |
| Immature personality disorder | E21y500 | Pesonality_disorder |
| Masochistic personality disorder | E21y600 | Pesonality_disorder |
| Psychoneurotic personality disorder | E21y700 | Pesonality_disorder |
| Other personality disorder NOS | E21yz00 | Pesonality_disorder |
| Personality disorder NOS | E21z.00 | Pesonality_disorder |
| Psychopathic personality | E21z.11 | Pesonality_disorder |
| [X]Organic personality disorder | Eu06000 | Pesonality_disorder |
| [X]Organic pseudopsychopathic personality | Eu06011 | Pesonality_disorder |
| [x]pseudopsychopathic schizophrenia | Eu21.17 | Pesonality_disorder |
| [x]schizotypal personality disorder | Eu21.18 | Pesonality_disorder |
| [X]Affective personality disorder | Eu34011 | Pesonality_disorder |
| [x]depressive personality disorder | Eu34112 | Pesonality_disorder |
| [X]Specific personality disorders | Eu60.00 | Pesonality_disorder |
| [X]Paranoid personality disorder | Eu60000 | Pesonality_disorder |
| [X]Querulant personality disorder | Eu60013 | Pesonality_disorder |
| [X]Sensitive paranoid personality disorder | Eu60014 | Pesonality_disorder |
| [x]schizoid personality disorder | Eu60100 | Pesonality_disorder |
| [X]Dissocial personality disorder | Eu60200 | Pesonality_disorder |
| [X]Amoral personality disorder | Eu60211 | Pesonality_disorder |
| [X]Antisocial personality disorder | Eu60212 | Pesonality_disorder |
| [X]Asocial personality disorder | Eu60213 | Pesonality_disorder |
| [X]Psychopathic personality disorder | Eu60214 | Pesonality_disorder |
| [X]Sociopathic personality disorder | Eu60215 | Pesonality_disorder |
| [X]Emotionally unstable personality disorder | Eu60300 | Pesonality_disorder |
| [X]Aggressive personality disorder | Eu60311 | Pesonality_disorder |
| [X]Borderline personality disorder | Eu60312 | Pesonality_disorder |
| [X]Explosive personality disorder | Eu60313 | Pesonality_disorder |
| [X]Histrionic personality disorder | Eu60400 | Pesonality_disorder |
| [X]Hysterical personality disorder | Eu60411 | Pesonality_disorder |
| [X]Psychoinfantile personality disorder | Eu60412 | Pesonality_disorder |
| [X]Anankastic personality disorder | Eu60500 | Pesonality_disorder |
| [X]Compulsive personality disorder | Eu60511 | Pesonality_disorder |
| [X]Obsessional personality disorder | Eu60512 | Pesonality_disorder |
| [X]Obsessive-compulsive personality disorder | Eu60513 | Pesonality_disorder |
| [x]anxious [avoidant] personality disorder | Eu60600 | Pesonality_disorder |
| [X]Dependent personality disorder | Eu60700 | Pesonality_disorder |
| [X]Asthenic personality disorder | Eu60711 | Pesonality_disorder |
| [X]Inadequate personality disorder | Eu60712 | Pesonality_disorder |
| [X]Passive personality disorder | Eu60713 | Pesonality_disorder |
| [X]Self defeating personality disorder | Eu60714 | Pesonality_disorder |
| [X]Addictive personality | Eu60800 | Pesonality_disorder |
| [X]Other specific personality disorders | Eu60y00 | Pesonality_disorder |
| [X]Eccentric personality disorder | Eu60y11 | Pesonality_disorder |
| [X]Haltlose type personality disorder | Eu60y12 | Pesonality_disorder |
| [X]Immature personality disorder | Eu60y13 | Pesonality_disorder |
| [X]Narcissistic personality disorder | Eu60y14 | Pesonality_disorder |
| [X]Psychoneurotic personality disorder | Eu60y16 | Pesonality_disorder |
| [X]Personality disorder; unspecified | Eu60z00 | Pesonality_disorder |
| [X]Character neurosis NOS | Eu60z11 | Pesonality_disorder |
| [X]Pathological personality NOS | Eu60z12 | Pesonality_disorder |
| [X]Mixed and other personality disorders | Eu61.00 | Pesonality_disorder |
| [x]autistic psychopathy | Eu84511 | Pesonality_disorder |
| delusions | 1BH..00 | Schizophrenia |
| delusion of persecution | 1BH0.00 | Schizophrenia |
| grandiose delusions | 1BH1.00 | Schizophrenia |
| delusion | 1BH..11 | Schizophrenia |
| o/e - paranoid delusions | 225E.00 | Schizophrenia |
| o/e - delusion of persecution | 225F.00 | Schizophrenia |
| psychotic condition; insight present | 285..11 | Schizophrenia |
| Non-organic psychoses | E1...00 | Schizophrenia |
| Schizophrenic disorders | E10..00 | Schizophrenia |
| Simple schizophrenia | E100.00 | Schizophrenia |
| Unspecified schizophrenia | E100000 | Schizophrenia |
| Subchronic schizophrenia | E100100 | Schizophrenia |
| Schizophrenia simplex | E100.11 | Schizophrenia |
| Chronic schizophrenic | E100200 | Schizophrenia |
| Acute exacerbation of subchronic schizophrenia | E100300 | Schizophrenia |
| Acute exacerbation of chronic schizophrenia | E100400 | Schizophrenia |
| Schizophrenia in remission | E100500 | Schizophrenia |
| Simple schizophrenia NOS | E100z00 | Schizophrenia |
| Hebephrenic schizophrenia | E101.00 | Schizophrenia |
| Unspecified hebephrenic schizophrenia | E101000 | Schizophrenia |
| Acute exacerbation of chronic hebephrenic schizophrenia | E101400 | Schizophrenia |
| Hebephrenic schizophrenia in remission | E101500 | Schizophrenia |
| Hebephrenic schizophrenia NOS | E101z00 | Schizophrenia |
| catatonic schizophrenia | E102.00 | Schizophrenia |
| unspecified catatonic schizophrenia | E102000 | Schizophrenia |
| subchronic catatonic schizophrenia | E102100 | Schizophrenia |
| acute exacerbation of chronic catatonic schizophrenia | E102400 | Schizophrenia |
| Catatonic schizophrenia in remission | E102500 | Schizophrenia |
| catatonic schizophrenia nos | E102z00 | Schizophrenia |
| paranoid schizophrenia | E103.00 | Schizophrenia |
| unspecified paranoid schizophrenia | E103000 | Schizophrenia |
| subchronic paranoid schizophrenia | E103100 | Schizophrenia |
| Chronic paranoid schizophrenia | E103200 | Schizophrenia |
| Acute exacerbation of subchronic paranoid schizophrenia | E103300 | Schizophrenia |
| Acute exacerbation of chronic paranoid schizophrenia | E103400 | Schizophrenia |
| Paranoid schizophrenia in remission | E103500 | Schizophrenia |
| Paranoid schizophrenia NOS | E103z00 | Schizophrenia |
| Acute schizophrenic episode | E104.00 | Schizophrenia |
| Latent schizophrenia | E105.00 | Schizophrenia |
| Unspecified latent schizophrenia | E105000 | Schizophrenia |
| Chronic latent schizophrenia | E105200 | Schizophrenia |
| Latent schizophrenia in remission | E105500 | Schizophrenia |
| Latent schizophrenia NOS | E105z00 | Schizophrenia |
| Residual schizophrenia | E106.00 | Schizophrenia |
| Schizo-affective schizophrenia | E107.00 | Schizophrenia |
| Unspecified schizo-affective schizophrenia | E107000 | Schizophrenia |
| Subchronic schizo-affective schizophrenia | E107100 | Schizophrenia |
| cyclic schizophrenia | E107.11 | Schizophrenia |
| Chronic schizo-affective schizophrenia | E107200 | Schizophrenia |
| Acute exacerbation subchronic schizo-affective schizophrenia | E107300 | Schizophrenia |
| Acute exacerbation of chronic schizo-affective schizophrenia | E107400 | Schizophrenia |
| Schizo-affective schizophrenia in remission | E107500 | Schizophrenia |
| Schizo-affective schizophrenia NOS | E107z00 | Schizophrenia |
| Other schizophrenia | E10y.00 | Schizophrenia |
| atypical schizophrenia | E10y000 | Schizophrenia |
| coenesthopathic schizophrenia | E10y100 | Schizophrenia |
| cenesthopathic schizophrenia | E10y.11 | Schizophrenia |
| other schizophrenia nos | E10yz00 | Schizophrenia |
| schizophrenia nos | E10z.00 | Schizophrenia |
| Other and unspecified affective psychoses | E11z.00 | Schizophrenia |
| Unspecified affective psychoses NOS | E11z000 | Schizophrenia |
| Other affective psychosis NOS | E11zz00 | Schizophrenia |
| chronic paranoid psychosis | E121.00 | Schizophrenia |
| paraphrenia | E122.00 | Schizophrenia |
| shared paranoid disorder | E123.00 | Schizophrenia |
| paranoia querulans | E12y000 | Schizophrenia |
| paranoid psychosis nos | E12z.00 | Schizophrenia |
| Other nonorganic psychoses | E13..00 | Schizophrenia |
| reactive depressive psychosis | E130.00 | Schizophrenia |
| psychotic reactive depression | E130.11 | Schizophrenia |
| reactive psychoses | E13..11 | Schizophrenia |
| acute paranoid reaction | E133.00 | Schizophrenia |
| psychogenic paranoid psychosis | E134.00 | Schizophrenia |
| Nonorganic psychosis NOS | E13z.00 | Schizophrenia |
| Psychotic episode NOS | E13z.11 | Schizophrenia |
| Psychoses with origin in childhood | E14..00 | Schizophrenia |
| Disintegrative psychosis | E141.00 | Schizophrenia |
| Residual disintegrative psychoses | E141100 | Schizophrenia |
| Other childhood psychoses | E14y.00 | Schizophrenia |
| Other childhood psychoses NOS | E14yz00 | Schizophrenia |
| Child psychosis NOS | E14z.00 | Schizophrenia |
| Childhood schizophrenia NOS | E14z.11 | Schizophrenia |
| Other specified non-organic psychoses | E1y..00 | Schizophrenia |
| Non-organic psychosis NOS | E1z..00 | Schizophrenia |
| [X] Presenile psychosis NOS | Eu02z12 | Schizophrenia |
| [X]Symptomatic psychosis NOS | Eu0z.12 | Schizophrenia |
| [X]Schizophrenia; schizotypal and delusional disorders | Eu2..00 | Schizophrenia |
| [X]Schizophrenia | Eu20.00 | Schizophrenia |
| [X]Paranoid schizophrenia | Eu20000 | Schizophrenia |
| [X]Paraphrenic schizophrenia | Eu20011 | Schizophrenia |
| [X]Hebephrenic schizophrenia | Eu20100 | Schizophrenia |
| [X]Disorganised schizophrenia | Eu20111 | Schizophrenia |
| [X]Catatonic schizophrenia | Eu20200 | Schizophrenia |
| [X]Catatonic stupor | Eu20211 | Schizophrenia |
| [X]Schizophrenic catalepsy | Eu20212 | Schizophrenia |
| [X]Schizophrenic catatonia | Eu20213 | Schizophrenia |
| [X]Schizophrenic flexibilatis cerea | Eu20214 | Schizophrenia |
| [X]Undifferentiated schizophrenia | Eu20300 | Schizophrenia |
| [X]Atypical schizophrenia | Eu20311 | Schizophrenia |
| [X]Residual schizophrenia | Eu20500 | Schizophrenia |
| [X]Chronic undifferentiated schizophrenia | Eu20511 | Schizophrenia |
| [X]Simple schizophrenia | Eu20600 | Schizophrenia |
| [X]Other schizophrenia | Eu20y00 | Schizophrenia |
| [X]Schizophreniform disord NOS | Eu20y12 | Schizophrenia |
| [X]Schizophrenifrm psychos NOS | Eu20y13 | Schizophrenia |
| [X]Schizophrenia; unspecified | Eu20z00 | Schizophrenia |
| [X]Latent schizophrenic reaction | Eu21.11 | Schizophrenia |
| [X]Latent schizophrenia | Eu21.13 | Schizophrenia |
| [X]Prepsychotic schizophrenia | Eu21.14 | Schizophrenia |
| [X]Prodromal schizophrenia | Eu21.15 | Schizophrenia |
| [X]Pseudoneurotic schizophrenia | Eu21.16 | Schizophrenia |
| [X]Pseudopsychopathic schizophrenia | Eu21.17 | Schizophrenia |
| [X]Persistent delusional disorders | Eu22.00 | Schizophrenia |
| [X]Delusional disorder | Eu22000 | Schizophrenia |
| [X]Paranoid psychosis | Eu22011 | Schizophrenia |
| [X]Paranoid state | Eu22012 | Schizophrenia |
| [X]Paraphrenia - late | Eu22013 | Schizophrenia |
| [X]Sensitiver Beziehungswahn | Eu22014 | Schizophrenia |
| [X]Paranoia | Eu22015 | Schizophrenia |
| [X]Delusional misidentification syndrome | Eu22100 | Schizophrenia |
| [X]Capgras syndrome | Eu22111 | Schizophrenia |
| [X]Other persistent delusional disorders | Eu22y00 | Schizophrenia |
| [X]Delusional dysmorphophobia | Eu22y11 | Schizophrenia |
| [X]Involutional paranoid state | Eu22y12 | Schizophrenia |
| [X]Persistent delusional disorder; unspecified | Eu22z00 | Schizophrenia |
| [X]Acute and transient psychotic disorders | Eu23.00 | Schizophrenia |
| [X]Acute polymorphic psychot disord without symp of schizoph | Eu23000 | Schizophrenia |
| [X]Cycloid psychosis | Eu23012 | Schizophrenia |
| [X]Acute polymorphic psychot disord with symp of schizophren | Eu23100 | Schizophrenia |
| [X]Cycloid psychosis with symptoms of schizophrenia | Eu23112 | Schizophrenia |
| [X]Acute schizophrenia-like psychotic disorder | Eu23200 | Schizophrenia |
| [X]Brief schizophreniform disorder | Eu23211 | Schizophrenia |
| [X]Brief schizophrenifrm psych | Eu23212 | Schizophrenia |
| [X]Schizophrenic reaction | Eu23214 | Schizophrenia |
| [X]Other acute predominantly delusional psychotic disorders | Eu23300 | Schizophrenia |
| [X]Psychogenic paranoid psychosis | Eu23312 | Schizophrenia |
| [X]Other acute and transient psychotic disorders | Eu23y00 | Schizophrenia |
| [X]Acute and transient psychotic disorder; unspecified | Eu23z00 | Schizophrenia |
| [x]induced delusional disorder | Eu24.00 | Schizophrenia |
| [x]induced paranoid disorder | Eu24.12 | Schizophrenia |
| [x]induced psychotic disorder | Eu24.13 | Schizophrenia |
| [X]Schizoaffective disorders | Eu25.00 | Schizophrenia |
| [x]schizoaffective disorder; manic type | Eu25000 | Schizophrenia |
| [x]schizoaffective psychosis; manic type | Eu25011 | Schizophrenia |
| [x]schizophreniform psychosis; manic type | Eu25012 | Schizophrenia |
| [X]Schizoaffective disorder; depressive type | Eu25100 | Schizophrenia |
| [X]Schizoaffective psychosis; depressive type | Eu25111 | Schizophrenia |
| [x]schizophreniform psychosis; depressive type | Eu25112 | Schizophrenia |
| [X]Schizoaffective disorder; mixed type | Eu25200 | Schizophrenia |
| [x]cyclic schizophrenia | Eu25211 | Schizophrenia |
| [X]Mixed schizophrenic and affective psychosis | Eu25212 | Schizophrenia |
| [X]Other schizoaffective disorders | Eu25y00 | Schizophrenia |
| [X]Schizoaffective disorder; unspecified | Eu25z00 | Schizophrenia |
| [X]Schizoaffective psychosis NOS | Eu25z11 | Schizophrenia |
| [X]Other nonorganic psychotic disorders | Eu2y.00 | Schizophrenia |
| [x]chronic hallucinatory psychosis | Eu2y.11 | Schizophrenia |
| [X]Unspecified nonorganic psychosis | Eu2z.00 | Schizophrenia |
| [x]psychosis nos | Eu2z.11 | Schizophrenia |
| [x]severe depressive episode with psychotic symptoms | Eu32300 | Schizophrenia |
| [x]single episode of major depression and psychotic symptoms | Eu32311 | Schizophrenia |
| [x]single episode of psychogenic depressive psychosis | Eu32312 | Schizophrenia |
| [x]single episode of psychotic depression | Eu32313 | Schizophrenia |
| [x]single episode of reactive depressive psychosis | Eu32314 | Schizophrenia |
| [X]Single major depr ep; severe with psych; psych in remiss | Eu32900 | Schizophrenia |
| [X]Recurr major depr ep; severe with psych; psych in remiss | Eu32A00 | Schizophrenia |
| [X]Recurrent depress disorder cur epi severe with psyc symp | Eu33300 | Schizophrenia |
| [x]endogenous depression with psychotic symptoms | Eu33311 | Schizophrenia |
| [x]recurr severe episodes/major depression+psychotic symptom | Eu33313 | Schizophrenia |
| [x]recurr severe episodes/psychogenic depressive psychosis | Eu33314 | Schizophrenia |
| [x]recurrent severe episodes of psychotic depression | Eu33315 | Schizophrenia |
| [x]recurrent severe episodes/reactive depressive psychosis | Eu33316 | Schizophrenia |
| [x]hysterical psychosis | Eu44.14 | Schizophrenia |
| [x]symbiotic psychosis | Eu84314 | Schizophrenia |
| [X]Schizoid disorder of childhood | Eu84512 | Schizophrenia |
| [d]hallucinations; auditory | R001000 | Schizophrenia |
| [d]hallucinations; gustatory | R001100 | Schizophrenia |
| [d]hallucinations; olfactory | R001200 | Schizophrenia |
| [d]hallucinations; tactile | R001300 | Schizophrenia |
| [d]hallucinations nos | R001z00 | Schizophrenia |
| [x]other hallucinations | Ryu5300 | Schizophrenia |
| schizophrenic language | ZS7C611 | Schizophrenia |

# Supplementary Table S4: Substance misuse codes

| **description** | **code** | **source** | **past** |
| --- | --- | --- | --- |
| Drug user | 13c..00 | original | F |
| Injecting drug user | 13c0.00 | original | F |
| Intravenous drug user | 13c1.00 | original | F |
| Intramuscular drug user | 13c3.00 | original | F |
| Intranasal drug user | 13c4.00 | original | F |
| Substance misuse increased | 13c5.00 | original | F |
| Substance misuse decreased | 13c6.00 | original | F |
| Current drug user | 13c7.00 | original | F |
| Reduced drugs misuse | 13c8.00 | original | F |
| Subcutaneous drug user | 13c9.00 | original | F |
| Smokes drugs | 13cA.00 | original | F |
| Misuses drugs orally | 13cB.00 | original | F |
| Continuous use of drugs | 13cC.00 | original | F |
| Episodic use of drugs | 13cD.00 | original | F |
| Prolonged high dose use of cannabis | 13cE.00 | original | F |
| Preoccupied with substance misuse | 13cF.00 | original | F |
| Persistent substance misuse | 13cH.00 | original | F |
| Previously injecting drug user | 13cJ.00 | original | T |
| Substance misuse | 13cM.00 | original | F |
| Failed heroin detoxification | 146C.00 | original | F |
| H/O: drug abuse | 146F.00 | original | T |
| History of substance misuse | 1T...00 | original | T |
| H/O heroin misuse | 1T0..00 | original | T |
| H/O daily heroin misuse | 1T00.00 | original | T |
| H/O weekly heroin misuse | 1T01.00 | original | T |
| Previous history of heroin misuse | 1T02.00 | original | T |
| H/O infrequent heroin misuse | 1T03.00 | original | T |
| H/O methadone misuse | 1T1..00 | original | T |
| H/O daily methadone misuse | 1T10.00 | original | T |
| H/O weekly methadone misuse | 1T11.00 | original | T |
| H/O infrequent methadone misuse | 1T12.00 | original | T |
| Previous history of methadone misuse | 1T13.00 | original | T |
| H/O ecstasy misuse | 1T2..00 | original | T |
| H/O daily ecstasy misuse | 1T20.00 | original | T |
| H/O weekly ecstasy misuse | 1T21.00 | original | T |
| H/O infrequent ecstasy misuse | 1T22.00 | original | T |
| Previous history of ecstasy misuse | 1T23.00 | original | T |
| H/O benzodiazepine misuse | 1T3..00 | original | T |
| H/O daily benzodiazepine misuse | 1T30.00 | original | T |
| H/O weekly benzodiazepine misuse | 1T31.00 | original | T |
| H/O infrequent benzodiazepine misuse | 1T32.00 | original | T |
| Previous history of benzodiazepine misuse | 1T33.00 | original | T |
| H/O amphetamine misuse | 1T4..00 | original | T |
| H/O daily amphetamine misuse | 1T40.00 | original | T |
| H/O weekly amphetamine misuse | 1T41.00 | original | T |
| H/O infrequent amphetamine misuse | 1T42.00 | original | T |
| Previous history of amphetamine misuse | 1T43.00 | original | T |
| H/O cocaine misuse | 1T5..00 | original | T |
| H/O daily cocaine misuse | 1T50.00 | original | T |
| H/O weekly cocaine misuse | 1T51.00 | original | T |
| H/O infrequent cocaine misuse | 1T52.00 | original | T |
| Previous history of cocaine misuse | 1T53.00 | original | T |
| H/O crack cocaine misuse | 1T6..00 | original | T |
| H/O daily crack cocaine misuse | 1T60.00 | original | T |
| H/O weekly crack cocaine misuse | 1T61.00 | original | T |
| H/O infrequent crack cocaine misuse | 1T62.00 | original | T |
| Previous history of crack cocaine misuse | 1T63.00 | original | T |
| H/O hallucinogen misuse | 1T7..00 | original | T |
| Previous history of hallucinogen misuse | 1T73.00 | original | T |
| H/O cannabis misuse | 1T8..00 | original | T |
| H/O daily cannabis misuse | 1T80.00 | original | T |
| H/O weekly cannabis misuse | 1T81.00 | original | T |
| H/O infrequent cannabis misuse | 1T82.00 | original | T |
| Previous history of cannabis misuse | 1T83.00 | original | T |
| H/O solvent misuse | 1T9..00 | original | T |
| H/O daily solvent misuse | 1T90.00 | original | T |
| H/O weekly solvent misuse | 1T91.00 | original | T |
| H/O infrequent solvent misuse | 1T92.00 | original | T |
| Previous history of solvent misuse | 1T93.00 | original | T |
| H/O barbiturate misuse | 1TA..00 | original | T |
| Previous history of barbiturate misuse | 1TA3.00 | original | T |
| H/O major tranquilliser misuse | 1TB..00 | original | T |
| H/O daily major tranquilliser misuse | 1TB0.00 | original | T |
| H/O anti-depressant misuse | 1TC..00 | original | T |
| H/O daily anti-depressant misuse | 1TC0.00 | original | T |
| Previous history of anti-depressant misuse | 1TC3.00 | original | T |
| H/O opiate misuse | 1TD..00 | original | T |
| H/O daily opiate misuse | 1TD0.00 | original | T |
| H/O weekly opiate misuse | 1TD1.00 | original | T |
| H/O infrequent opiate misuse | 1TD2.00 | original | T |
| Previous history of opiate misuse | 1TD3.00 | original | T |
| Uses heroin on top of substitution therapy | 1TE..00 | original | F |
| Does not use heroin on top of substitution therapy | 1TF..00 | original | F |
| Drug misuse behaviour | 1V...00 | original | F |
| Misuses drugs | 1V0..00 | original | F |
| Occasional drug user | 1V00.00 | original | F |
| Long-term drug misuser | 1V01.00 | original | F |
| Poly-drug misuser | 1V02.00 | original | F |
| Notified addict | 1V07.00 | original | F |
| Smokes drugs in cigarette form | 1V08.00 | original | F |
| Smokes drugs through a pipe | 1V09.00 | original | F |
| Chases the dragon | 1V0A.00 | original | F |
| Sniffs drugs | 1V0B.00 | original | F |
| Drug addict | 1V0C.00 | original | F |
| Health problem secondary to drug misuse | 1V0E.00 | original | F |
| Age at starting drug misuse | 1V22.00 | original | F |
| Misused drugs in past | 1V26.00 | original | T |
| Shares drug equipment | 1V35.00 | original | F |
| Sharing of drug injecting equipment | 1V38.00 | original | F |
| Drug-related offending behaviour | 1V6..00 | original | F |
| Possession of drugs | 1V63.00 | original | F |
| Illicit drug use | 1V64.00 | original | F |
| Heroin misuse | 1V65.00 | original | F |
| Ecstasy misuse | 1V66.00 | original | F |
| Substance misuse structured counselling | 677T.00 | original | F |
| Delivery of rehabilitation for drug addiction | 7P22000 | original | F |
| Drug abuse monitoring | 8AA..00 | original | F |
| Drug addiction therapy | 8B23.00 | original | F |
| Drug addictn therap-methadone | 8B23.11 | original | F |
| Drug addiction detoxification therapy - methadone | 8B2N.00 | original | F |
| Drug addiction maintenance therapy - methadone | 8B2P.00 | original | F |
| Drug addiction maintenance therapy - buprenorphine | 8B2Q.00 | original | F |
| Drug addiction detoxification therapy - buprenorphine | 8B2R.00 | original | F |
| Opioid agonist substitution therapy | 8B2S.00 | original | F |
| Opioid antagonist therapy | 8B2T.00 | original | F |
| Opiate dependence detoxification | 8BAd.00 | original | F |
| Benzodiazepine dependence detoxification | 8BAo.00 | original | F |
| Drug dependence self detoxification | 8BAW.00 | original | F |
| Drug dependence home detoxification | 8BAX.00 | original | F |
| Referral to drug abuse counsellor | 8H7x.00 | original | F |
| Self referral to substance misuse service | 8Hh1.00 | original | F |
| Referral to substance misuse service | 8HkF.00 | original | F |
| Admission to substance misuse detoxification centre | 8Hq..00 | original | F |
| Drug addiction notification | 9G2..00 | original | F |
| Drug addict notific to CMO | 9G21.00 | original | F |
| Drug addict notific admin | 9G2..11 | original | F |
| Drug addict re-notific due | 9G22.00 | original | F |
| Drug addict re-notif to CMO | 9G23.00 | original | F |
| Drug addict-notify local SMR22 | 9G24.00 | original | F |
| Drug addiction notif NOS | 9G2Z.00 | original | F |
| Substance misuse monitoring | 9HC..00 | original | F |
| Follow up substance misuse assessment | 9HC1.00 | original | F |
| Substance misuse clinical management plan agreed | 9HC2.00 | original | F |
| Substance misuse clinical management plan reviewed | 9HC3.00 | original | F |
| Substance misuse treatment withdrawn | 9HC4.00 | original | F |
| Substance misuse treatment programme completed | 9HC5.00 | original | F |
| Substance misuse treatment declined | 9HC6.00 | original | F |
| SMR25a drug misuse initial assessment form | 9K4..00 | original | F |
| Drug misuse - enhanced services administration | 9k5..00 | original | F |
| Drug misuse - enhanced service completed | 9k50.00 | original | F |
| Shared care drug misuse treatment - enhanced services admin | 9k51.00 | original | F |
| Shared care drug misuse treatment | 9k51.11 | original | F |
| Drug misuse treatment primary care - enhanced services admin | 9k52.00 | original | F |
| Pharmacy attended for drug misuse - enhanced services admin | 9k53.00 | original | F |
| Drug misuse assessment declined - enhanced services administ | 9kS..00 | original | F |
| Seen in drug misuse clinic | 9N1yJ00 | original | F |
| DNA - Did not attend substance misuse clinic | 9N4i.00 | original | F |
| Declined consent for notification of drug misuse | 9NdN.00 | original | F |
| Seen in substance misuse clinic | 9No5.00 | original | F |
| Drug misuse clinic administration | 9s...00 | original | F |
| Drug dependence | E24..00 | original | F |
| Opioid type drug dependence | E240.00 | original | F |
| Unspecified opioid dependence | E240000 | original | F |
| Continuous opioid dependence | E240100 | original | F |
| Heroin dependence | E240.11 | original | F |
| Methadone dependence | E240.12 | original | F |
| Morphine dependence | E240.13 | original | F |
| Opium dependence | E240.14 | original | F |
| Episodic opioid dependence | E240200 | original | F |
| Opioid dependence in remission | E240300 | original | F |
| Opioid drug dependence NOS | E240z00 | original | F |
| Drug addiction | E24..11 | original | F |
| Librium dependence | E241.15 | original | F |
| Cocaine type drug dependence | E242.00 | original | F |
| Cocaine dependence; unspecified | E242000 | original | F |
| Cocaine dependence; continuous | E242100 | original | F |
| Cocaine dependence; episodic | E242200 | original | F |
| Cocaine dependence in remission | E242300 | original | T |
| Cocaine drug dependence NOS | E242z00 | original | F |
| Cannabis type drug dependence | E243.00 | original | F |
| Cannabis dependence; unspecified | E243000 | original | F |
| Cannabis dependence; continuous | E243100 | original | F |
| Hashish dependence | E243.11 | original | F |
| Hemp dependence | E243.12 | original | F |
| Marihuana dependence | E243.13 | original | F |
| Cannabis dependence; episodic | E243200 | original | F |
| Cannabis dependence in remission | E243300 | original | T |
| Cannabis drug dependence NOS | E243z00 | original | F |
| Amphetamine or other psychostimulant dependence | E244.00 | original | F |
| Amphetamine or psychostimulant dependence; unspecified | E244000 | original | F |
| Amfetamine or psychostimulant dependence; unspecified | E244011 | original | F |
| Amphetamine or psychostimulant dependence; continuous | E244100 | original | F |
| Psychostimulant dependence | E244.11 | original | F |
| Stimulant dependence | E244.12 | original | F |
| Amphetamine or psychostimulant dependence; episodic | E244200 | original | F |
| Amphetamine or psychostimulant dependence in remission | E244300 | original | T |
| Amphetamine or psychostimulant dependence NOS | E244z00 | original | F |
| Amfetamine or psychostimulant dependence NOS | E244z11 | original | F |
| Hallucinogen dependence | E245.00 | original | F |
| Hallucinogen dependence; unspecified | E245000 | original | F |
| Hallucinogen dependence; continuous | E245100 | original | F |
| LSD dependence | E245.11 | original | F |
| Lysergic acid diethylamide dependence | E245.12 | original | F |
| Hallucinogen dependence; episodic | E245200 | original | F |
| Hallucinogen dependence in remission | E245300 | original | T |
| Hallucinogen dependence NOS | E245z00 | original | F |
| Glue sniffing dependence | E246.00 | original | F |
| Glue sniffing dependence; unspecified | E246000 | original | F |
| Glue sniffing dependence; continuous | E246100 | original | F |
| Glue sniffing dependence; episodic | E246200 | original | F |
| Glue sniffing dependence in remission | E246300 | original | T |
| Glue sniffing dependence NOS | E246z00 | original | F |
| Other specified drug dependence; continuous | E247100 | original | F |
| Other specified drug dependence; episodic | E247200 | original | F |
| Other specified drug dependence in remission | E247300 | original | T |
| Combined opioid with other drug dependence | E248.00 | original | F |
| Combined opioid with other drug dependence; unspecified | E248000 | original | F |
| Combined opioid with other drug dependence; continuous | E248100 | original | F |
| Combined opioid with other drug dependence; episodic | E248200 | original | F |
| Combined opioid with other drug dependence in remission | E248300 | original | T |
| Combined opioid with other drug dependence NOS | E248z00 | original | F |
| Combined drug dependence; excluding opioids | E249.00 | original | F |
| Combined drug dependence; excluding opioid; unspecified | E249000 | original | F |
| Combined drug dependence; excluding opioid; continuous | E249100 | original | F |
| Combined drug dependence; excluding opioid; episodic | E249200 | original | F |
| Combined drug dependence; excluding opioid; in remission | E249300 | original | T |
| Combined drug dependence; excluding opioid; NOS | E249z00 | original | F |
| Ecstasy type drug dependence | E24A.00 | original | F |
| Drug dependence NOS | E24z.00 | original | F |
| Nondependent abuse of drugs | E25..00 | original | F |
| Nondependent cannabis abuse | E252.00 | original | F |
| Nondependent cannabis abuse; unspecified | E252000 | original | F |
| Nondependent cannabis abuse; continuous | E252100 | original | F |
| Nondependent cannabis abuse; episodic | E252200 | original | F |
| Nondependent cannabis abuse in remission | E252300 | original | T |
| Nondependent cannabis abuse NOS | E252z00 | original | F |
| Nondependent hallucinogen abuse | E253.00 | original | F |
| Nondependent hallucinogen abuse; unspecified | E253000 | original | F |
| Nondependent hallucinogen abuse; continuous | E253100 | original | F |
| Nondependent hallucinogen abuse; episodic | E253200 | original | F |
| Nondependent hallucinogen abuse in remission | E253300 | original | T |
| Nondependent hallucinogen abuse NOS | E253z00 | original | F |
| Nondependent hypnotic or anxiolytic abuse | E254.00 | original | F |
| Nondependent hypnotic or anxiolytic abuse; unspecified | E254000 | original | F |
| Nondependent hypnotic or anxiolytic abuse; continuous | E254100 | original | F |
| Barbiturate abuse | E254.11 | original | F |
| Hypnotic or anxiolytic abuse | E254.12 | original | F |
| Sedative abuse | E254.13 | original | F |
| Tranquilliser abuse | E254.14 | original | F |
| Nondependent hypnotic or anxiolytic abuse; episodic | E254200 | original | F |
| Nondependent hypnotic or anxiolytic abuse in remission | E254300 | original | T |
| Nondependent hypnotic or anxiolytic abuse NOS | E254z00 | original | F |
| Nondependent opioid abuse | E255.00 | original | F |
| Nondependent opioid abuse; unspecified | E255000 | original | F |
| Nondependent opioid abuse; continuous | E255100 | original | F |
| Nondependent opioid abuse; episodic | E255200 | original | F |
| Nondependent opioid abuse in remission | E255300 | original | T |
| Nondependent opioid abuse NOS | E255z00 | original | F |
| Nondependent cocaine abuse | E256.00 | original | F |
| Nondependent cocaine abuse; unspecified | E256000 | original | F |
| Nondependent cocaine abuse; continuous | E256100 | original | F |
| Nondependent cocaine abuse; episodic | E256200 | original | F |
| Nondependent cocaine abuse in remission | E256300 | original | T |
| Nondependent cocaine abuse NOS | E256z00 | original | F |
| Nondependent amphetamine or other psychostimulant abuse | E257.00 | original | F |
| Nondependent amphetamine/psychostimulant abuse; unspecified | E257000 | original | F |
| Nondependent amphetamine/psychostimulant abuse; continuous | E257100 | original | F |
| Psychostimulant abuse | E257.11 | original | F |
| Stimulant abuse | E257.12 | original | F |
| Nondependent amphetamine or psychostimulant abuse; episodic | E257200 | original | F |
| Nondependent amphetamine/psychostimulant abuse in remission | E257300 | original | T |
| Nondependent amphetamine or psychostimulant abuse NOS | E257z00 | original | F |
| Nondependent antidepressant type drug abuse | E258.00 | original | F |
| Nondependent antidepressant type drug abuse NOS | E258z00 | original | F |
| Nondependent mixed drug abuse | E259.00 | original | F |
| Nondependent mixed drug abuse; unspecified | E259000 | original | F |
| Nondependent mixed drug abuse; continuous | E259100 | original | F |
| Nondependent mixed drug abuse; episodic | E259200 | original | F |
| Nondependent mixed drug abuse in remission | E259300 | original | T |
| Misuse of prescription only drugs | E259400 | original | F |
| Nondependent mixed drug abuse NOS | E259z00 | original | F |
| Nondependent other drug abuse | E25y.00 | original | F |
| Nondependent other drug abuse; unspecified | E25y000 | original | F |
| Nondependent other drug abuse; continuous | E25y100 | original | F |
| Analgesic abuse | E25y.11 | original | F |
| Steroid abuse | E25y.13 | original | F |
| Nondependent other drug abuse; episodic | E25y200 | original | F |
| Nondependent other drug abuse in remission | E25y300 | original | T |
| Nondependent other drug abuse NOS | E25yz00 | original | F |
| Misuse of drugs NOS | E25z.00 | original | F |
| Other adjustment reaction with withdrawal | E29y500 | original | F |
| [X]Mental and behav dis due to use opioids: dependence syndr | Eu11200 | original | F |
| [X]Drug addiction - opioids | Eu11211 | original | F |
| [X]Heroin addiction | Eu11212 | original | F |
| [X]Mental and behav dis due to use opioids: withdrawal state | Eu11300 | original | F |
| [X]Cold turkey; opiate withdrawal | Eu11311 | original | F |
| [X]Men & behav dis due opioid: withdrawl state with delirium | Eu11400 | original | F |
| [X]Mental & behav dis due to use opioids: psychotic disorder | Eu11500 | original | F |
| [X]Mental and behav dis due to use opioids: amnesic syndrome | Eu11600 | original | F |
| [X]Men & beh dis due opioids: resid & late-onset psychot dis | Eu11700 | original | F |
| [X]Men & behav dis due to use opioids: oth men & behav dis | Eu11y00 | original | F |
| [X]Ment & behav dis due use opioids: unsp ment & behav dis | Eu11z00 | original | F |
| [X]Mental and behavioural disorders due to use cannabinoids | Eu12.00 | original | F |
| [X]Mental & behav dis due cannabinoids: acute intoxication | Eu12000 | original | F |
| [X]Mental and behav dis due to use cannabinoids: harmful use | Eu12100 | original | F |
| [X]Mental and behav dis due to cannabinoids: dependence synd | Eu12200 | original | F |
| [X]Drug addiction - cannabis | Eu12211 | original | F |
| [X]Mental and behav dis due cannabinoids: withdrawal state | Eu12300 | original | F |
| [X]Mental & behav dis due to cannabinoids: psychotic disordr | Eu12500 | original | F |
| [X]Mental and behav dis due to use cannabinoids: amnesic syn | Eu12600 | original | F |
| [X]Mnt/bh dis due cannabinds: resid & late-onset psychot dis | Eu12700 | original | F |
| [X]Men/behav dis due to use cannabinoids: oth men/behav disd | Eu12y00 | original | F |
| [X]Ment/behav dis due use cannabinoids: unsp ment/behav disd | Eu12z00 | original | F |
| [X]Mental and behavioural dis due use sedatives/hypnotics | Eu13.00 | original | F |
| [X]Mental & behav dis due seds/hypntcs: acute intoxication | Eu13000 | original | F |
| [X]Mental and behav dis due to use seds/hypntcs: harmful use | Eu13100 | original | F |
| [X]Mental and behav dis due to seds/hypntcs: dependence synd | Eu13200 | original | F |
| [X]Drug addiction- sedative / hypnotics | Eu13211 | original | F |
| [X]Mental and behav dis due seds/hypntcs: withdrawal state | Eu13300 | original | F |
| [X]Men & beh dis due seds/hypns: withdrwl state wth delirium | Eu13400 | original | F |
| [X]Mental & behav dis due to seds/hypntcs: psychotic disordr | Eu13500 | original | F |
| [X]Mental and behavioural disorders due to use of cocaine | Eu14.00 | original | F |
| [X]Mental & behav dis due to use cocaine: acute intoxication | Eu14000 | original | F |
| [X]Mental and behav dis due to use of cocaine: harmful use | Eu14100 | original | F |
| [X]Mental and behav dis due to use cocaine: dependence syndr | Eu14200 | original | F |
| [X]Drug addiction - cocaine | Eu14211 | original | F |
| [X]Mental and behav dis due to use cocaine: withdrawal state | Eu14300 | original | F |
| [X]Mental & behav dis due to use cocaine: psychotic disorder | Eu14500 | original | F |
| [X]Men & beh dis due cocaine: resid & late-onset psychot dis | Eu14700 | original | F |
| [X]Mental & behav disorder due other stimulants inc caffein | Eu15.00 | original | F |
| [X]Mnt/beh dis due oth stim inc caffein: acute intoxication | Eu15000 | original | F |
| [X]Ment/behav dis due to use oth stims inc caff: harmful use | Eu15100 | original | F |
| [X]Mental and behav dis oth stim inc caffein: dependnce synd | Eu15200 | original | F |
| [X]Drug addiction-other stimul | Eu15211 | original | F |
| [X]Mnt/behav dis other stimlnts inc caffeine: withdrwl state | Eu15300 | original | F |
| [X]Mental/behav dis oth stims inc caffeine: psychotic dis | Eu15500 | original | F |
| [X]Mnt/bh dis oth stm inc caffne resid/late-onset psycht dis | Eu15700 | original | F |
| [X]Ment/beh dis oth stims inc caffeine: unsp ment/behav disd | Eu15z00 | original | F |
| [X]Mental and behavioural disorders due to use hallucinogens | Eu16.00 | original | F |
| [X]Mental & behav dis due hallucinogens: acute intoxicatn | Eu16000 | original | F |
| [X]Mental and behav dis due to use hallucinogens: harmfl use | Eu16100 | original | F |
| [X]Mental and behav dis due to hallucinogens: dependence syn | Eu16200 | original | F |
| [X]Drug addiction - hallucinogen | Eu16211 | original | F |
| [X]Mental and behav dis due hallucinogens: withdrawal state | Eu16300 | original | F |
| [X]Mental & behav dis due to hallucinogens: psychotic disord | Eu16500 | original | F |
| [X]Mnt/bh dis due hallucngns: resid & late-onset psychot dis | Eu16700 | original | F |
| [X]Post hallucinogen perception disorder | Eu16711 | original | F |
| [X]Ment/behav dis due use hallucinogens: unsp ment/behav dis | Eu16z00 | original | F |
| [X]Mental and behav dis due to use tobacco: dependence syndr | Eu17200 | original | F |
| [X]Mental and behav dis due to use tobacco: withdrawal state | Eu17300 | original | F |
| [X]Mental & behav disorders due to use of volatile solvents | Eu18.00 | original | F |
| [X]Mental & behav dis due vol solvents: acute intoxication | Eu18000 | original | F |
| [X]Mental and behav dis due volatile solvents: harmful use | Eu18100 | original | F |
| [X]Mental and behav dis due to vol solvents: dependence synd | Eu18200 | original | F |
| [X]Drug addiction - solvent | Eu18211 | original | F |
| [X]Men & beh dis vol solvents: withdrawal state wth delirium | Eu18400 | original | F |
| [X]Mental & behav dis due to vol solvents: psychotic disordr | Eu18500 | original | F |
| [X]Ment/behav dis due use vol solvents: unsp ment/behav dis | Eu18z00 | original | F |
| [X]Men & behav disorder multiple drug use/psychoactive subst | Eu19.00 | original | F |
| [X]Mental/behav dis multi drg use/psychoac subs: acute intox | Eu19000 | original | F |
| [X]Mental and behav dis mlti drg/oth psychoa sbs: harmfl use | Eu19100 | original | F |
| [X]Mental and behav dis mlti/oth psych sbs: dependence syndr | Eu19200 | original | F |
| [X]Drug addiction NOS | Eu19211 | original | F |
| [X]Mental and behav dis mlti/oth psychoa sbs: withdrwl state | Eu19300 | original | F |
| [X]Mnt/bh dis mlti drg use/oth psy sbs: wthdr state + dlrium | Eu19400 | original | F |
| [X]Ment/behav dis mlti drug use/oth psyc sbs: psychotc dis | Eu19500 | original | F |
| [X]Mental/behav dis multi drg use/oth psy sbs: amnesic syndr | Eu19600 | original | F |
| [X]Men/beh dis mlt drg use/oth subs: resid/late psychot dis | Eu19700 | original | F |
| [X]Ment/beh dis multi drug use/oth psy sbs unsp mnt/beh dis | Eu19z00 | original | F |
| [X]Mental and behavioural disorders due use of crack cocaine | Eu1A.00 | original | F |
| [X]Ment behav dis due use crack cocaine: acute intoxication | Eu1A000 | original | F |
| [X]Mental behav disorders due use crack cocaine: harmful use | Eu1A100 | original | F |
| [X]Mental behav disorders due use crack cocaine: depend synd | Eu1A200 | original | F |
| [X]Mental behav disord due crack cocaine: withdrawal state | Eu1A300 | original | F |
| [X]Mental behav disord due crack cocaine: psychotic disorder | Eu1A500 | original | F |
| [X]Ment behav dis due crack cocaine: unsp ment and behav dis | Eu1Az00 | original | F |
| [X]Abuse of non-dependence-producing substances | Eu55.00 | original | F |
| [X]Abuse of steroids or hormones | Eu55.13 | original | F |
| Drug dependence in pregnancy; childbirth and the puerperium | L183.00 | original | F |
| Drug dependence during pregnancy - baby delivered | L183100 | original | F |
| Pregnancy and drug dependence | L183.11 | original | F |
| Drug dependence during pregnancy - baby not yet delivered | L183300 | original | F |
| Drug dependence during pregnancy/childbirth/puerperium NOS | L183z00 | original | F |
| Drug withdrawal regime | Z192100 | original | F |
| Substitute prescribing | Z1Q6.00 | original | F |
| Methadone maintenance | Z1Q6200 | original | F |
| Methadone therapy | Z1Q6212 | original | F |
| Heroin maintenance | Z1Q6214 | original | F |
| Dexamphetamine maintenance | Z1Q6300 | original | F |
| Substance abuse counselling | Z416.00 | original | F |
| Advice on drugs of addiction | ZG23200 | original | F |
| Cocaine abuse assessment profile | ZR3Z.00 | original | F |
| CAAP - Cocaine abuse assessment profile | ZR3Z.11 | original | F |
| DAST - Drug abuse screening test | ZRBK.11 | original | F |
| Severity of opiate dependence questionnaire | ZRk7.00 | original | F |
| SODQ - Severity of opiate dependence questionnaire | ZRk7.11 | original | F |
| Substance use disorder diagnostic schedule | ZRl..00 | original | F |
| SUDDS - Substance use disorder diagnostic schedule | ZRl..11 | original | F |
| Maudsley addiction profile | ZRVn.00 | original | F |
| MAP - Maudsley addiction profile | ZRVn.11 | original | F |
| [V]Personal history of psychoactive substance abuse | ZV11400 | original | T |
| [V]Personal history of drug abuse by injection | ZV11500 | original | T |
| Novel psychoactive substance misuse | 13cM000 | new | F |
| Opioid analgesic dependence | 13cM100 | new | F |
| H/O novel psychoactive substance misuse | 1TG..00 | new | T |
| Substance misuse assessment | 38C3.00 | new | F |
| Initial substance misuse assessment | 9HC0.00 | new | F |
| Substance misuse treatment not available | 9HC7.00 | new | F |
| Family intervention for substance misuse | 8G22.00 | new | F |
| Never injecting drug user | 13c2.00 | new | F |
| Craving for drugs | 1P6..00 | new | F |
| Craves for drugs | 1P60.00 | new | F |
| Abnormal craving for drugs | 1P62.00 | new | F |
| Excessive craving for drugs | 1P63.00 | new | F |
| Irresistible craving for drugs | 1P64.00 | new | F |
| Misuses drugs sublingually | 1V03.00 | new | F |
| Misuses drugs rectally | 1V04.00 | new | F |
| Misuses drugs vaginally | 1V05.00 | new | F |
| Amount of money spent per day on drug habit | 1V0D.00 | new | F |
| Time devoted to drug related activities | 1V1..00 | new | F |
| Time spent obtaining drugs | 1V10.00 | new | F |
| Time spent taking drugs | 1V11.00 | new | F |
| Time spent recovering from drugs | 1V12.00 | new | F |
| Compulsive drug taking | 1P31.00 | new | F |
| Referral to drugs worker | 8Hl6.00 | new | F |
| Seen by community drug team | 9Nlb.00 | new | F |
| Hypnotic or anxiolytic dependence | E241.00 | new | F |
| Hypnotic or anxiolytic dependence in remission | E241300 | new | T |
| Hypnotic or anxiolytic dependence NOS | E241z00 | new | F |
| Hypnotic or anxiolytic dependence, continuous | E241100 | new | F |
| Hypnotic or anxiolytic dependence, episodic | E241200 | new | F |
| Hypnotic or anxiolytic dependence, unspecified | E241000 | new | F |
| Injects drugs intramuscularly | 1V31.00 | new | F |
| Injects drugs subcutaneously | 1V30.00 | new | F |
| LSD reaction | E253.12 | new | F |
| Neck injector | 1V32.00 | new | F |
| Other drug psychoses | E02y.00 | new | F |
| Other drug psychoses NOS | E02yz00 | new | F |
| Other specified drug dependence | E247.00 | new | F |
| Other specified drug dependence, unspecified | E247000 | new | F |
| Pathological drug intoxication | E022.00 | new | F |
| Priority of drug-related activities | 1V4..00 | new | F |
| Reinduction to buprenorphine maintenance therapy | 8BE1.00 | new | F |
| Reinduction to methadone maintenance therapy | 8BE0.00 | new | F |
| Routine of drug-related activities | 1V5..00 | new | F |
| Sedative dependence | E241.16 | new | F |
| Substance misuse management stopped - self withdrawal | 8BAc.00 | new | T |
| Time since stopped drug misuse | 1V23.00 | new | T |
| Urine buprenorphine positive | 46Qr000 | new | F |
| Urine methadone positive | 46QB100 | new | F |
| Valium dependence | E241.17 | new | F |
| [V]Drug abuse counselling and surveillance | ZV6D700 | new | F |
| [X] Adverse reaction to methadone | U605012 | new | F |
| [X]Mental & behav dis due to use opioids: acute intoxication | Eu11000 | new | F |
| [X]Mental and behav dis due to use of opioids: harmful use | Eu11100 | new | F |
| [X]Mental and behavioural disorders due to psychoactive subs | Eu1..00 | new | F |
| [X]Mental and behavioural disorders due to use of opioids | Eu11.00 | new | F |
| Absinthe addiction | E247.11 | new | F |
| Abstinent from drug misuse on maintenance replacement | 13r3.00 | new | T |
| Abstinent from drug misuse when receiving blocking therapy | 13r4.00 | new | T |
| Adverse reaction to methadone | TJ51.00 | new | F |
| Anxiolytic dependence | E241.11 | new | F |
| 'Bad trips' | E253.11 | new | F |
| Barbiturate dependence | E241.12 | new | F |
| Benzodiazepine dependence | E241.13 | new | F |
| Buprenorphine maintenance therapy | 8B2M.00 | new | F |
| Current non recreational drug user | 13cK.00 | new | F |
| Detoxification dependence drug | 8BA9.00 | new | F |
| Diazepam dependence | E241.14 | new | F |
| Drug dependence therapy | 8B23.13 | new | F |
| Drug injecting equipment hygiene | 1V37.00 | new | F |
| Drug injection behaviour | 1V3..00 | new | F |
| Drug psychoses | E02..00 | new | F |
| Drug psychosis NOS | E02z.00 | new | F |
| Drug withdrawal syndrome | E020.00 | new | F |
| Drug-induced delirium | E02y000 | new | F |
| Drug-induced dementia | E02y100 | new | F |
| Drug-induced depressive state | E02y300 | new | F |
| Drug-induced hallucinosis | E021100 | new | F |
| Drug-induced paranoia or hallucinatory state NOS | E021z00 | new | F |
| Drug-induced paranoia or hallucinatory states | E021.00 | new | F |
| Drug-induced paranoid state | E021000 | new | F |
| Drug-induced personality disorder | E02y400 | new | F |
| FP10(MDA) issued | 8B23.12 | new | F |
| Drug addiction maintenance therapy - naltrexone | 8B23000 | new | F |
| Drug addiction maintenance therapy - lofexidine | 8B23100 | new | F |
| Drug addiction maintenance therapy - buprenorphine | 8B2Q.00 | new | F |
| [X]Mental and behavioural disorders due to use of other stimulants, including caffeine: dependence syndrome | Eu15200 | new | F |
| [X]Mental and behavioural disorders due to use of other stimulants, including caffeine: dependence syndrome | Eu15211 | new | F |
| [X]Mental and behavioural disorders due to use of other stimulants, including caffeine: withdrawal state with delirium | Eu15400 | new | F |
| [X]Mental and behavioural disorders due to use of other stimulants, including caffeine: amnesic syndrome | Eu15600 | new | F |
| [X]Mental and behavioural disorders due to use of other stimulants, including caffeine: other mental and behavioural disorders | Eu15y00 | new | F |
| [X]Mental and behavioural disorders due to use of hallucinogens: withdrawal state with delirium | Eu16400 | new | F |
| [X]Mental and behavioural disorders due to use of hallucinogens: amnesic syndrome | Eu16600 | new | F |
| [X]Mental and behavioural disorders due to use of hallucinogens: other mental and behavioural disorders | Eu16y00 | new | F |

# Supplementary Table S5: Epilepsy codes

| **description** | **code** |
| --- | --- |
| H/O: epilepsy | 1473.00 |
| Transient epileptic amnesia | 1B1W.00 |
| Epilepsy confirmed | 1O30.00 |
| Epilepsy resolved | 2126000 |
| Epilepsy resolved | 212J.00 |
| Contraceptive advice for patients with epilepsy | 6110.00 |
| Epilepsy associated problems | 6674.00 |
| Epilepsy monitoring | 667..00 |
| Epilepsy drug side effects | 6677.00 |
| Epilepsy treatment changed | 6678.00 |
| Epilepsy treatment started | 6679.00 |
| Epilepsy treatment stopped | 667A.00 |
| Nocturnal epilepsy | 667B.00 |
| Epilepsy control good | 667C.00 |
| Epilepsy control poor | 667D.00 |
| Epilepsy care arrangement | 667E.00 |
| Seizure free >12 months | 667F.00 |
| Epilepsy restricts employment | 667G.00 |
| Epilepsy prevents employment | 667H.00 |
| Epilepsy impairs education | 667J.00 |
| Epilepsy limits activities | 667K.00 |
| Epilepsy does not limit activities | 667L.00 |
| Epilepsy management plan given | 667M.00 |
| Epilepsy severity | 667N.00 |
| No seizures on treatment | 667P.00 |
| 1 to 12 seizures a year | 667Q.00 |
| 2 to 4 seizures a month | 667R.00 |
| 1 to 7 seizures a week | 667S.00 |
| Daily seizures | 667T.00 |
| Many seizures a day | 667V.00 |
| Emergency epilepsy treatment since last appointment | 667W.00 |
| No epilepsy drug side effects | 667X.00 |
| Epilepsy monitoring NOS | 667Z.00 |
| Pregnancy advice for patients with epilepsy | 67AF.00 |
| Pre-conception advice for patients with epilepsy | 67IJ000 |
| Epilepsy medication review | 8BIF.00 |
| Epilepsy monitoring verbal invite | 9Of3.00 |
| Epilepsy monitoring telephone invite | 9Of4.00 |
| Epilepsy monitoring call first letter | 9Of5.00 |
| Epilepsy monitoring call second letter | 9Of6.00 |
| Epilepsy monitoring call third letter | 9Of7.00 |
| [X]Schizophrenia-like psychosis in epilepsy | Eu05212 |
| [X]Epileptic psychosis NOS | Eu05y11 |
| [X]Limbic epilepsy personality | Eu06013 |
| [X]Acquired aphasia with epilepsy [Landau - Kleffner] | Eu80300 |
| Progressive myoclonic epilepsy | F132100 |
| Unverricht - Lundborg disease | F132111 |
| Myoclonic encephalopathy | F132200 |
| Dyssynergia cerebellaris myoclonica | F142200 |
| Petit mal (minor) epilepsy | F250000 |
| Epileptic absences | F250011 |
| Pykno-epilepsy | F250100 |
| Epileptic seizures - atonic | F250200 |
| Epileptic seizures - akinetic | F250300 |
| Juvenile absence epilepsy | F250400 |
| Lennox-Gastaut syndrome | F250500 |
| Generalised nonconvulsive epilepsy | F250.00 |
| Other specified generalised nonconvulsive epilepsy | F250y00 |
| Generalised nonconvulsive epilepsy NOS | F250z00 |
| Grand mal (major) epilepsy | F251000 |
| Tonic-clonic epilepsy | F251011 |
| Neonatal myoclonic epilepsy | F251100 |
| Otohara syndrome | F251111 |
| Epileptic seizures - clonic | F251200 |
| Epileptic seizures - myoclonic | F251300 |
| Epileptic seizures - tonic | F251400 |
| Tonic-clonic epilepsy | F251500 |
| Generalised convulsive epilepsy | F251.00 |
| Other specified generalised convulsive epilepsy | F251y00 |
| Generalised convulsive epilepsy NOS | F251z00 |
| Petit mal status | F252.00 |
| Grand mal status | F253.00 |
| Status epilepticus | F253.11 |
| Temporal lobe epilepsy | F254000 |
| Psychomotor epilepsy | F254100 |
| Psychosensory epilepsy | F254200 |
| Limbic system epilepsy | F254300 |
| Epileptic automatism | F254400 |
| Complex partial epileptic seizure | F254500 |
| Partial epilepsy with impairment of consciousness | F254.00 |
| Partial epilepsy with impairment of consciousness NOS | F254z00 |
| Jacksonian, focal or motor epilepsy | F255000 |
| Focal epilepsy | F255011 |
| Motor epilepsy | F255012 |
| Sensory induced epilepsy | F255100 |
| Somatosensory epilepsy | F255200 |
| Visceral reflex epilepsy | F255300 |
| Partial epilepsy with autonomic symptoms | F255311 |
| Visual reflex epilepsy | F255400 |
| Unilateral epilepsy | F255500 |
| Simple partial epileptic seizure | F255600 |
| Partial epilepsy without impairment of consciousness | F255.00 |
| Partial epilepsy without impairment of consciousness OS | F255y00 |
| Partial epilepsy without impairment of consciousness NOS | F255z00 |
| Hypsarrhythmia | F256000 |
| Salaam attacks | F256100 |
| Infantile spasms | F256.00 |
| Lightning spasms | F256.11 |
| West syndrome | F256.12 |
| Infantile spasms NOS | F256z00 |
| Kojevnikov's epilepsy | F257.00 |
| Post-ictal state | F258.00 |
| Early infant epileptic encephalopathy wth suppression bursts | F259.00 |
| Ohtahara syndrome | F259.11 |
| Juvenile myoclonic epilepsy | F25A.00 |
| Alcohol-induced epilepsy | F25B.00 |
| Drug-induced epilepsy | F25C.00 |
| Menstrual epilepsy | F25D.00 |
| Stress-induced epilepsy | F25E.00 |
| Epilepsy | F25..00 |
| Photosensitive epilepsy | F25F.00 |
| Severe myoclonic epilepsy in infancy | F25G.00 |
| Dravet syndrome | F25G.11 |
| Status epilepticus, unspecified | F25X.00 |
| Cursive (running) epilepsy | F25y000 |
| Gelastic epilepsy | F25y100 |
| Locl-rlt(foc)(part)idiop epilep&epilptic syn seiz locl onset | F25y200 |
| Complex partial status epilepticus | F25y300 |
| Benign Rolandic epilepsy | F25y400 |
| Panayiotopoulos syndrome | F25y500 |
| Other forms of epilepsy | F25y.00 |
| Other forms of epilepsy NOS | F25yz00 |
| Epilepsy NOS | F25z.00 |
| Fit (in known epileptic) NOS | F25z.11 |
| [X]Other generalized epilepsy and epileptic syndromes | Fyu5000 |
| [X]Other epilepsy | Fyu5100 |
| [X]Other status epilepticus | Fyu5200 |
| [X]Status epilepticus, unspecified | Fyu5900 |

# Supplementary Table S6: Cerebral palsy codes

| **description** | **code** |
| --- | --- |
| Gross Motor Function Classification System Cerebral Palsy | 38Gw.00 |
| Athetoid cerebral palsy | F137000 |
| Vogt's disease | F137011 |
| Double athetosis | F137100 |
| Congenital athetosis | F137111 |
| Athetoid cerebral palsy | F137.11 |
| Athetosis - congenital | F137.12 |
| Congenital paraplegia | F230000 |
| Cerebral palsy with spastic diplegia | F230100 |
| Spastic diplegic cerebral palsy | F230111 |
| Congenital diplegia | F230.00 |
| Paraplegia - congenital | F230.11 |
| Congenital diplegia NOS | F230z00 |
| Congenital hemiplegia | F231.00 |
| Congenital quadriplegia | F232.00 |
| Tetraplegia - congenital | F232.11 |
| Congenital monoplegia | F233.00 |
| Congenital spastic foot | F233.11 |
| Infantile hemiplegia NOS | F234.00 |
| Congenital cerebral palsy | F23..00 |
| Congenital spastic cerebral palsy | F23..11 |
| Infantile cerebral palsy | F23..12 |
| Littles disease | F23..13 |
| Cerebral atonia | F23..14 |
| Ataxic infantile cerebral palsy | F23y000 |
| Flaccid infantile cerebral palsy | F23y100 |
| Spastic cerebral palsy | F23y200 |
| Dyskinetic cerebral palsy | F23y300 |
| Ataxic diplegic cerebral palsy | F23y400 |
| Worster-Drought syndrome | F23y500 |
| Congenital suprabulbar paresis | F23y511 |
| Choreoathetoid cerebral palsy | F23y600 |
| Other congenital cerebral palsy | F23y.00 |
| Other infantile cerebral palsy NOS | F23yz00 |
| Congenital cerebral palsy NOS | F23z.00 |
| Spastic quadriplegic cerebral palsy | F2B0.00 |
| Spastic hemiplegic cerebral palsy | F2B1.00 |
| Cerebral palsy | F2B..00 |
| Other cerebral palsy | F2By.00 |
| Cerebral palsy NOS | F2Bz.00 |
| [X]Other infantile cerebral palsy | Fyu9000 |
| [X]Cerebral palsy and other paralytic syndromes | Fyu9.00 |
| Cerebral palsy, not congenital or infantile, acute | G669.00 |

# Supplementary Table S7: Autistic spectrum disorder codes

| **description** | **code** |
| --- | --- |
| Active infantile autism | E140000 |
| Residual infantile autism | E140100 |
| Infantile autism | E140.00 |
| Kanner's syndrome | E140.11 |
| Autism | E140.12 |
| Childhood autism | E140.13 |
| Infantile autism NOS | E140z00 |
| [X]Childhood autism | Eu84000 |
| [X]Autistic disorder | Eu84011 |
| [X]Infantile autism | Eu84012 |
| [X]Kanner's syndrome | Eu84014 |
| [X]Atypical autism | Eu84100 |
| [X]Mental retardation with autistic features | Eu84112 |
| [X]Asperger's syndrome | Eu84500 |
| [X]Autistic psychopathy | Eu84511 |
| [X]Autistic spectrum disorder | Eu84z11 |

# Supplementary Table S8: Intellectual disability codes

| **description** | **code** |
| --- | --- |
| On learning disability register | 918e.00 |
| Preferred place of death: learning disability unit | 94Z9.00 |
| Learning disabilities health action plan declined | 9HB0.00 |
| Learning disabilities health action plan offered | 9HB1.00 |
| Learning disabilities health action plan reviewed | 9HB2.00 |
| Learning disabilities health assessment | 9HB3.00 |
| Learning disabilities health action plan completed | 9HB4.00 |
| Learning disabilities annual health assessment | 9HB5.00 |
| Learning disabilities annual health assessment declined | 9HB6.00 |
| Learning disabilities annual health check declined | 9HB6.11 |
| Did not attend learning disabilities annual health assessmnt | 9HB7.00 |
| Did not attend learning disabilities annual health check | 9HB7.11 |
| Learning disabilities administration status | 9HB..00 |
| Learning disability annual health check verbal invitation | 9mA0.00 |
| Learning disability annual health check telephone invitation | 9mA1.00 |
| Learning disability annual health check invtation 1st letter | 9mA2000 |
| Learning disability annual health check invtation 2nd letter | 9mA2100 |
| Learning disability annual health check invtation 3rd letter | 9mA2200 |
| Learning disability annual health check letter invitation | 9mA2.00 |
| Learning disability annual health check invitation | 9mA..00 |
| Other specific learning difficulty | E2F2.00 |
| Mild mental retardation, IQ in range 50-70 | E30..00 |
| Educationally subnormal | E30..11 |
| Feeble-minded | E30..12 |
| Moron | E30..13 |
| Moderate mental retardation, IQ in range 35-49 | E310.00 |
| Imbecile | E310.11 |
| Severe mental retardation, IQ in range 20-34 | E311.00 |
| Profound mental retardation with IQ less than 20 | E312.00 |
| Idiocy | E312.11 |
| Other specified mental retardation | E31..00 |
| Other specified mental retardation NOS | E31z.00 |
| Mental retardation | E3...00 |
| Other specified mental retardation | E3y..00 |
| Mental retardation NOS | E3z..00 |
| [X]Mld mental retard with statement no or min impairm behav | Eu70000 |
| [X]Mld mental retard sig impairment behav req attent/treatmt | Eu70100 |
| [X]Mild mental retardation | Eu70.00 |
| [X]Mild mental subnormality | Eu70.12 |
| [X]Mild mental retardation, other impairments of behaviour | Eu70y00 |
| [X]Mild mental retardation without mention impairment behav | Eu70z00 |
| [X]Mod mental retard with statement no or min impairm behav | Eu71000 |
| [X]Mod mental retard sig impairment behav req attent/treatmt | Eu71100 |
| [X]Moderate mental retardation | Eu71.00 |
| [X]Moderate mental subnormality | Eu71.11 |
| [X]Mod retard oth behav impair | Eu71y00 |
| [X]Mod mental retardation without mention impairment behav | Eu71z00 |
| [X]Sev mental retard with statement no or min impairm behav | Eu72000 |
| [X]Sev mental retard sig impairment behav req attent/treatmt | Eu72100 |
| [X]Severe mental retardation | Eu72.00 |
| [X]Severe mental subnormality | Eu72.11 |
| [X]Severe mental retardation, other impairments of behaviour | Eu72y00 |
| [X]Sev mental retardation without mention impairment behav | Eu72z00 |
| [X]Profound ment retrd wth statement no or min impairm behav | Eu73000 |
| [X]Profound ment retard sig impairmnt behav req attent/treat | Eu73100 |
| [X]Profound mental retardation | Eu73.00 |
| [X]Profound mental subnormality | Eu73.11 |
| [X]Profound mental retardation, other impairments of behavr | Eu73y00 |
| [X]Prfnd mental retardation without mention impairment behav | Eu73z00 |
| [X]Mental retardation | Eu7..00 |
| [X]Oth mental retard with statement no or min impairm behav | Eu7y000 |
| [X]Oth mental retard sig impairment behav req attent/treatmt | Eu7y100 |
| [X]Other mental retardation | Eu7y.00 |
| [X]Other mental retardation, other impairments of behaviour | Eu7yy00 |
| [X]Other mental retardation without mention impairment behav | Eu7yz00 |
| [X]Unsp mental retard with statement no or min impairm behav | Eu7z000 |
| [X]Unsp mentl retard sig impairment behav req attent/treatmt | Eu7z100 |
| [X]Unspecified mental retardation | Eu7z.00 |
| [X]Mental deficiency NOS | Eu7z.11 |
| [X]Mental subnormality NOS | Eu7z.12 |
| [X]Unspecified mental retardatn, other impairments of behav | Eu7zy00 |
| [X]Unsp mental retardation without mention impairment behav | Eu7zz00 |
| [X]Moderate learning disability | Eu81400 |
| [X]Severe learning disability | Eu81500 |
| [X]Mild learning disability | Eu81600 |
| [X]Profound learning disability | Eu81700 |
| [X]Specific learning disability | Eu81800 |
| [X]Developmental disorder of scholastic skills, unspecified | Eu81z00 |
| [X]Learning disability NOS | Eu81z11 |
| [X]Learning disorder NOS | Eu81z12 |
| [X]Learn acquisition disab NOS | Eu81z13 |
| [X]Mental retardation with autistic features | Eu84112 |
| [X]Overactive disorder assoc mental retard/stereotype movts | Eu84400 |

# Supplementary Table S9: Malignancy codes

| **description** | **code** |
| --- | --- |
| Malignant neoplasm of intrahepatic bile ducts | B151.00 |
| Malignant neoplasm of interlobular bile ducts | B151000 |
| Malignant neoplasm of intrahepatic biliary passages | B151200 |
| Malignant neoplasm of intrahepatic gall duct | B151400 |
| Malignant neoplasm of intrahepatic bile ducts NOS | B151z00 |
| Malignant neoplasm of extrahepatic bile ducts | B161.00 |
| Malignant neoplasm of cystic duct | B161000 |
| Malignant neoplasm of hepatic duct | B161100 |
| Malignant neoplasm of common bile duct | B161200 |
| Carcinoma common bile duct | B161211 |
| Malignant neoplasm of sphincter of Oddi | B161300 |
| Malignant neoplasm of extrahepatic bile ducts NOS | B161z00 |
| Malignant neoplasm of ampulla of Vater | B162.00 |
| Malignant neoplasm, overlapping lesion of biliary tract | B163.00 |
| [M]Cholangiocarcinoma | BB5D100 |
| [M]Bile duct carcinoma | BB5D111 |
| [M]Bile duct cystadenocarcinoma | BB5D300 |
| [M]Combined hepatocellular carcinoma and cholangiocarcinoma | BB5D700 |
| [M]Hepatocholangiocarcinoma | BB5D711 |
| Malignant neoplasm of urinary bladder | B49..00 |
| Malignant neoplasm of trigone of urinary bladder | B490.00 |
| Malignant neoplasm of dome of urinary bladder | B491.00 |
| Malignant neoplasm of lateral wall of urinary bladder | B492.00 |
| Malignant neoplasm of anterior wall of urinary bladder | B493.00 |
| Malignant neoplasm of posterior wall of urinary bladder | B494.00 |
| Malignant neoplasm of bladder neck | B495.00 |
| Malignant neoplasm of ureteric orifice | B496.00 |
| Malignant neoplasm of urachus | B497.00 |
| Local recurrence of malignant tumour of urinary bladder | B498.00 |
| Malignant neoplasm of other site of urinary bladder | B49y.00 |
| Malignant neoplasm, overlapping lesion of bladder | B49y000 |
| Malignant neoplasm of urinary bladder NOS | B49z.00 |
| [V]Personal history of malignant neoplasm of bladder | ZV10511 |
| Malignant neoplasm of bone and articular cartilage | B30..00 |
| Malignant neoplasm of bones of skull and face | B300.00 |
| Malignant neoplasm of ethmoid bone | B300000 |
| Malignant neoplasm of frontal bone | B300100 |
| Malignant neoplasm of malar bone | B300200 |
| Malignant neoplasm of nasal bone | B300300 |
| Malignant neoplasm of occipital bone | B300400 |
| Malignant neoplasm of orbital bone | B300500 |
| Malignant neoplasm of parietal bone | B300600 |
| Malignant neoplasm of sphenoid bone | B300700 |
| Malignant neoplasm of temporal bone | B300800 |
| Malignant neoplasm of zygomatic bone | B300900 |
| Malignant neoplasm of maxilla | B300A00 |
| Malignant neoplasm of turbinate | B300B00 |
| Malignant neoplasm of vomer | B300C00 |
| Malignant neoplasm of bones of skull and face NOS | B300z00 |
| Malignant neoplasm of mandible | B301.00 |
| Malignant neoplasm of vertebral column | B302.00 |
| Malignant neoplasm of cervical vertebra | B302000 |
| Malignant neoplasm of thoracic vertebra | B302100 |
| Malignant neoplasm of lumbar vertebra | B302200 |
| Malignant neoplasm of vertebral column NOS | B302z00 |
| Malignant neoplasm of ribs, sternum and clavicle | B303.00 |
| Malignant neoplasm of rib | B303000 |
| Malignant neoplasm of sternum | B303100 |
| Malignant neoplasm of clavicle | B303200 |
| Malignant neoplasm of costal cartilage | B303300 |
| Malignant neoplasm of costo-vertebral joint | B303400 |
| Malignant neoplasm of xiphoid process | B303500 |
| Malignant neoplasm of rib, sternum and clavicle NOS | B303z00 |
| Malignant neoplasm of scapula and long bones of upper arm | B304.00 |
| Malignant neoplasm of scapula | B304000 |
| Malignant neoplasm of acromion | B304100 |
| Malignant neoplasm of humerus | B304200 |
| Malignant neoplasm of radius | B304300 |
| Malignant neoplasm of ulna | B304400 |
| Malig neop of scapula and long bones of upper arm NOS | B304z00 |
| Malignant neoplasm of hand bones | B305.00 |
| Malignant neoplasm of carpal bones | B305.11 |
| Malignant neoplasm of metacarpal bones | B305.12 |
| Malignant neoplasm of carpal bone - scaphoid | B305000 |
| Malignant neoplasm of carpal bone - lunate | B305100 |
| Malignant neoplasm of third metacarpal bone | B305A00 |
| Malignant neoplasm of fifth metacarpal bone | B305C00 |
| Malignant neoplasm of phalanges of hand | B305D00 |
| Malignant neoplasm of hand bones NOS | B305z00 |
| Malignant neoplasm of pelvic bones, sacrum and coccyx | B306.00 |
| Malignant neoplasm of ilium | B306000 |
| Malignant neoplasm of ischium | B306100 |
| Malignant neoplasm of pubis | B306200 |
| Malignant neoplasm of sacral vertebra | B306300 |
| Malignant neoplasm of coccygeal vertebra | B306400 |
| Malignant sacral teratoma | B306500 |
| Malignant neoplasm of pelvis, sacrum or coccyx NOS | B306z00 |
| Malignant neoplasm of long bones of leg | B307.00 |
| Malignant neoplasm of femur | B307000 |
| Malignant neoplasm of fibula | B307100 |
| Malignant neoplasm of tibia | B307200 |
| Malignant neoplasm of long bones of leg NOS | B307z00 |
| Malignant neoplasm of short bones of leg | B308.00 |
| Malignant neoplasm of talus | B308100 |
| Malignant neoplasm of calcaneum | B308200 |
| Malignant neoplasm of medial cuneiform | B308300 |
| Malignant neoplasm of first metatarsal bone | B308800 |
| Malignant neoplasm of fourth metatarsal bone | B308B00 |
| Malignant neoplasm of phalanges of foot | B308D00 |
| Malignant neoplasm of short bones of leg NOS | B308z00 |
| Malignant neoplasm/overlap lesion/bone+articulr cartilage | B30W.00 |
| Malignant neoplasm/bones+articular cartilage/limb,unspfd | B30X.00 |
| Malignant neoplasm of bone and articular cartilage NOS | B30z.00 |
| Osteosarcoma | B30z000 |
| [M]Juxtacortical osteogenic sarcoma | BBV..11 |
| [M]Parosteal osteosarcoma | BBV..12 |
| [M]Periosteal osteogenic sarcoma | BBV..13 |
| [M]Osteosarcoma NOS | BBV1.00 |
| [M]Osteoblastic sarcoma | BBV1.11 |
| [M]Osteochondrosarcoma | BBV1.12 |
| [M]Osteogenic sarcoma NOS | BBV1.13 |
| [M]Chondroblastic osteosarcoma | BBV2.00 |
| [M]Fibroblastic osteosarcoma | BBV3.00 |
| [M]Telangiectatic osteosarcoma | BBV4.00 |
| [M]Osteosarcoma in Paget's disease of bone | BBV5.00 |
| [M]Myxoid chondrosarcoma | BBV9.00 |
| [M] Small cell osteosarcoma | BBVA.00 |
| [M]Chondrosarcoma NOS | BBW4.00 |
| [M]Fibrochondrosarcoma | BBW4.11 |
| [M]Juxtacortical chondrosarcoma | BBW6.00 |
| [M]Chondroblastoma, malignant | BBW8.00 |
| [M]Mesenchymal chondrosarcoma | BBW9.00 |
| [M]Giant cell tumour of bone, malignant | BBX1.00 |
| [M]Giant cell bone sarcoma | BBX1.11 |
| [M]Osteoclastoma, malignant | BBX1.12 |
| [M]Ewing's sarcoma | BBY0.00 |
| [M]Endothelial bone sarcoma | BBY0.11 |
| [M]Adamantinoma of long bones | BBY1.00 |
| [M]Tibial adamantinoma | BBY1.11 |
| [M]Odontogenic tumour, malignant | BBZ2.00 |
| [M]Intraosseous carcinoma | BBZ2.11 |
| [M]Ameloblastic odontosarcoma | BBZC.00 |
| [M]Ameloblastoma, malignant | BBZG.00 |
| [M]Adamantinoma, malignant | BBZG.11 |
| [M]Ameloblastic fibrosarcoma | BBZN.00 |
| [M]Odontogenic fibrosarcoma | BBZN.11 |
| [X]Malignant neoplasm of bone and articular cartilage | Byu3.00 |
| [X]Malignant neoplasm/bones+articular cartilage/limb,unspfd | Byu3100 |
| [X]Malignant neoplasm/overlap lesion/bone+articulr cartilage | Byu3200 |
| [X]Malignant neoplasm/bone+articular cartilage, unspecified | Byu3300 |
| [V]Personal history of malignant neoplasm of bone | ZV10y11 |
| Malignant neoplasm of colon | B13..00 |
| Malignant neoplasm of hepatic flexure of colon | B130.00 |
| Malignant neoplasm of transverse colon | B131.00 |
| Malignant neoplasm of descending colon | B132.00 |
| Malignant neoplasm of sigmoid colon | B133.00 |
| Malignant neoplasm of caecum | B134.00 |
| Carcinoma of caecum | B134.11 |
| Malignant neoplasm of appendix | B135.00 |
| Malignant neoplasm of ascending colon | B136.00 |
| Malignant neoplasm of splenic flexure of colon | B137.00 |
| Malignant neoplasm, overlapping lesion of colon | B138.00 |
| Hereditary nonpolyposis colon cancer | B139.00 |
| Malignant neoplasm of other specified sites of colon | B13y.00 |
| Malignant neoplasm of colon NOS | B13z.00 |
| Colonic cancer | B13z.11 |
| Malignant neoplasm of rectum, rectosigmoid junction and anus | B14..00 |
| Malignant neoplasm of rectosigmoid junction | B140.00 |
| Malignant neoplasm of rectum | B141.00 |
| Carcinoma of rectum | B141.11 |
| Rectal carcinoma | B141.12 |
| Malignant neoplasm of anal canal | B142.00 |
| Anal carcinoma | B142.11 |
| Malignant neoplasm of cloacogenic zone | B142000 |
| Malignant neoplasm of anus unspecified | B143.00 |
| Malig neop other site rectum, rectosigmoid junction and anus | B14y.00 |
| Malignant neoplasm rectum,rectosigmoid junction and anus NOS | B14z.00 |
| Cancer of bowel | B1z0.11 |
| [V]Personal history of malignant neoplasm of anus | ZV10011 |
| [V]Personal history of malignant neoplasm of large intestine | ZV10014 |
| [V]Personal history of malignant neoplasm of rectum | ZV10017 |
| Malignant neoplasm of brain | B51..00 |
| Cerebral tumour - malignant | B51..11 |
| Malignant neoplasm cerebrum (excluding lobes and ventricles) | B510.00 |
| Malignant neoplasm of basal ganglia | B510000 |
| Malignant neoplasm of cerebral cortex | B510100 |
| Malignant neoplasm of globus pallidus | B510300 |
| Malignant neoplasm of hypothalamus | B510400 |
| Malignant neoplasm of thalamus | B510500 |
| Malignant neoplasm of cerebrum NOS | B510z00 |
| Malignant neoplasm of frontal lobe | B511.00 |
| Malignant neoplasm of temporal lobe | B512.00 |
| Malignant neoplasm of hippocampus | B512000 |
| Malignant neoplasm of temporal lobe NOS | B512z00 |
| Malignant neoplasm of parietal lobe | B513.00 |
| Malignant neoplasm of occipital lobe | B514.00 |
| Malignant neoplasm of cerebral ventricles | B515.00 |
| Malignant neoplasm of choroid plexus | B515000 |
| Malignant neoplasm of cerebellum | B516.00 |
| Malignant neoplasm of brain stem | B517.00 |
| Malignant neoplasm of cerebral peduncle | B517000 |
| Malignant neoplasm of medulla oblongata | B517100 |
| Malignant neoplasm of midbrain | B517200 |
| Malignant neoplasm of pons | B517300 |
| Malignant neoplasm of brain stem NOS | B517z00 |
| Malignant neoplasm of other parts of brain | B51y.00 |
| Malignant neoplasm of corpus callosum | B51y000 |
| Malignant neoplasm, overlapping lesion of brain | B51y200 |
| Malignant neoplasm of other part of brain NOS | B51yz00 |
| Malignant neoplasm of brain NOS | B51z.00 |
| Malignant neoplasm of cranial nerves | B520.00 |
| Malignant neoplasm of olfactory bulb | B520000 |
| Malignant neoplasm of optic nerve | B520100 |
| Malignant neoplasm of acoustic nerve | B520200 |
| Malignant neoplasm of cranial nerves NOS | B520z00 |
| Malignant neoplasm of cerebral meninges | B521.00 |
| Malignant neoplasm of cerebral pia mater | B521200 |
| Malignant neoplasm of cerebral meninges NOS | B521z00 |
| Malignant neoplasm of spinal cord | B522.00 |
| Malignant neoplasm of spinal meninges | B523.00 |
| Malignant neoplasm of spinal meninges NOS | B523z00 |
| Malignant neoplasm of cauda equina | B525.00 |
| Malig neopl, overlap lesion brain & other part of CNS | B52W.00 |
| Malignant neoplasm of meninges, unspecified | B52X.00 |
| Malignant neoplasm pituitary gland and craniopharyngeal duct | B542.00 |
| Malignant neoplasm of pituitary gland | B542000 |
| Malignant neoplasm of craniopharyngeal duct | B542100 |
| Malig neop pituitary gland or craniopharyngeal duct NOS | B542z00 |
| Malignant neoplasm of pineal gland | B543.00 |
| Malignant neoplasm of carotid body | B544.00 |
| Malignant neoplasm of aortic body and other paraganglia | B545.00 |
| Malignant neoplasm of glomus jugulare | B545000 |
| Malignant neoplasm of aortic body | B545100 |
| Malignant neoplasm of coccygeal body | B545200 |
| Malignant neoplasm of aortic body or paraganglia NOS | B545z00 |
| [M]Astrocytoma NOS | BBbB.00 |
| [M]Astrocytic glioma | BBbB.11 |
| [M]Astrocytoma, anaplastic type | BBbC.00 |
| [M]Gemistocytic astrocytoma | BBbE.00 |
| [M]Fibrillary astrocytoma | BBbF.00 |
| [M]Pilocytic astrocytoma | BBbG.00 |
| [M]Juvenile astrocytoma | BBbG.11 |
| [M]Piloid astrocytoma | BBbG.12 |
| [M]Spongioblastoma NOS | BBbH.00 |
| [M]Astroblastoma | BBbK.00 |
| [M]Glioblastoma NOS | BBbL.00 |
| [M]Glioblastoma multiforme | BBbL.11 |
| [M]Giant cell glioblastoma | BBbM.00 |
| [M]Oligodendroglioma NOS | BBbQ.00 |
| [M]Oligodendroglioma, anaplastic type | BBbR.00 |
| [M]Oligodendroblastoma | BBbS.00 |
| [M]Medulloblastoma NOS | BBbT.00 |
| [M]Desmoplastic medulloblastoma | BBbU.00 |
| [M]Medullomyoblastoma | BBbV.00 |
| [M]Cerebellar sarcoma NOS | BBbW.00 |
| [M]Pleomorphic xanthoastrocytoma | BBbZ.00 |
| [M]Glioma NOS | BBbz.00 |
| [M]Medulloepithelioma NOS | BBc2.00 |
| [M]Glioneuroma | BBc6.11 |
| [M]Neuroastrocytoma | BBc7.11 |
| [M]Olfactory neurogenic tumour | BBcA.00 |
| [M]Meningioma, malignant | BBd2.00 |
| [M]Meningothelial sarcoma | BBd2.12 |
| [M]Papillary meningioma | BBdA.00 |
| [X]Malignant neoplasm/other and unspecified cranial nerves | ByuA000 |
| [X]Malignant neoplasm/central nervous system, unspecified | ByuA100 |
| [X]Malignant neoplasm of meninges, unspecified | ByuA200 |
| [X]Malig neopl, overlap lesion brain & other part of CNS | ByuA300 |
| [V]Personal history of malignant neoplasm of brain | ZV10y12 |
| Malignant neoplasm of female breast | B34..00 |
| Ca female breast | B34..11 |
| Malignant neoplasm of nipple and areola of female breast | B340.00 |
| Malignant neoplasm of nipple of female breast | B340000 |
| Malignant neoplasm of areola of female breast | B340100 |
| Malignant neoplasm of nipple or areola of female breast NOS | B340z00 |
| Malignant neoplasm of central part of female breast | B341.00 |
| Malignant neoplasm of upper-inner quadrant of female breast | B342.00 |
| Malignant neoplasm of lower-inner quadrant of female breast | B343.00 |
| Malignant neoplasm of upper-outer quadrant of female breast | B344.00 |
| Malignant neoplasm of lower-outer quadrant of female breast | B345.00 |
| Malignant neoplasm of axillary tail of female breast | B346.00 |
| Malignant neoplasm, overlapping lesion of breast | B347.00 |
| Malignant neoplasm of other site of female breast | B34y.00 |
| Malignant neoplasm of ectopic site of female breast | B34y000 |
| Malignant neoplasm of other site of female breast NOS | B34yz00 |
| Malignant neoplasm of female breast NOS | B34z.00 |
| Malignant neoplasm of male breast | B35..00 |
| Malignant neoplasm of nipple and areola of male breast | B350.00 |
| Malignant neoplasm of nipple of male breast | B350000 |
| Malignant neoplasm of areola of male breast | B350100 |
| Malignant neoplasm of other site of male breast | B35z.00 |
| Malignant neoplasm of ectopic site of male breast | B35z000 |
| Malignant neoplasm of male breast NOS | B35zz00 |
| Local recurrence of malignant tumour of breast | B36..00 |
| Carcinoma in situ of breast | B830.00 |
| Lobular carcinoma in situ of breast | B830000 |
| Intraductal carcinoma in situ of breast | B830100 |
| [M]Infiltrating duct carcinoma | BB91.00 |
| [M]Duct carcinoma NOS | BB91.11 |
| [M]Intraductal papillary adenocarcinoma with invasion | BB91000 |
| [M]Infiltrating duct and lobular carcinoma | BB91100 |
| [M]Comedocarcinoma, noninfiltrating | BB92.00 |
| [M]Comedocarcinoma NOS | BB93.00 |
| [M]Juvenile breast carcinoma | BB94.00 |
| [M]Secretory breast carcinoma | BB94.11 |
| [M]Noninfiltrating intraductal papillary adenocarcinoma | BB96.00 |
| [M]Paget's disease, mammary | BB9J.00 |
| [M]Paget's disease, breast | BB9J.11 |
| [M]Paget's disease and infiltrating breast duct carcinoma | BB9K.00 |
| [M]Paget's disease and intraductal carcinoma of breast | BB9K000 |
| [M]Intracystic carcinoma NOS | BB9M.00 |
| [X]Malignant neoplasm of breast | Byu6.00 |
| [X]Other carcinoma in situ of breast | ByuFG00 |
| [V]Personal history of malignant neoplasm of breast | ZV10300 |
| Malignant neoplasm of cervix uteri | B41..00 |
| Cervical carcinoma (uterus) | B41..11 |
| Malignant neoplasm of endocervix | B410.00 |
| Malignant neoplasm of endocervical canal | B410000 |
| Malignant neoplasm of endocervical gland | B410100 |
| Malignant neoplasm of endocervix NOS | B410z00 |
| Malignant neoplasm of exocervix | B411.00 |
| Malignant neoplasm, overlapping lesion of cervix uteri | B412.00 |
| Malignant neoplasm of other site of cervix | B41y.00 |
| Malignant neoplasm of cervical stump | B41y000 |
| Malignant neoplasm of squamocolumnar junction of cervix | B41y100 |
| Malignant neoplasm of other site of cervix NOS | B41yz00 |
| Malignant neoplasm of cervix uteri NOS | B41z.00 |
| [V]Personal history of malignant neoplasm of cervix uteri | ZV10411 |
| Renal malignant neoplasm | B4A..11 |
| Malignant neoplasm of kidney parenchyma | B4A0.00 |
| Hypernephroma | B4A0000 |
| Malignant neoplasm of renal pelvis | B4A1.00 |
| Malignant neoplasm of renal calyces | B4A1000 |
| Malignant neoplasm of ureteropelvic junction | B4A1100 |
| Malignant neoplasm of renal pelvis NOS | B4A1z00 |
| Malignant neoplasm of ureter | B4A2.00 |
| [M]Renal cell carcinoma | BB5a000 |
| [M]Grawitz tumour | BB5a011 |
| [M]Hypernephroma | BB5a012 |
| [V]Personal history of malignant neoplasm of kidney | ZV10512 |
| [V]Personal history of malignant neoplasm of kidney | ZV10513 |
| Primary malignant neoplasm of liver | B150.00 |
| Primary carcinoma of liver | B150000 |
| Hepatoblastoma of liver | B150100 |
| Primary angiosarcoma of liver | B150200 |
| Hepatocellular carcinoma | B150300 |
| Primary malignant neoplasm of liver NOS | B150z00 |
| Malignant neoplasm of liver unspecified | B152.00 |
| [M]Hepatocellular carcinoma NOS | BB5D500 |
| [M]Hepatoma NOS | BB5D511 |
| [M]Hepatoma, malignant | BB5D512 |
| [M]Liver cell carcinoma | BB5D513 |
| [M]Combined hepatocellular carcinoma and cholangiocarcinoma | BB5D700 |
| [M]Hepatocholangiocarcinoma | BB5D711 |
| [M]Hepatocellular carcinoma, fibrolamellar | BB5D800 |
| [X]Other specified carcinomas of liver | Byu1100 |
| [V]Personal history of malignant neoplasm of liver | ZV10015 |
| Malignant neoplasm of trachea, bronchus and lung | B22..00 |
| Malignant neoplasm of trachea | B220.00 |
| Malignant neoplasm of mucosa of trachea | B220100 |
| Malignant neoplasm of trachea NOS | B220z00 |
| Malignant neoplasm of main bronchus | B221.00 |
| Malignant neoplasm of carina of bronchus | B221000 |
| Malignant neoplasm of hilus of lung | B221100 |
| Malignant neoplasm of main bronchus NOS | B221z00 |
| Malignant neoplasm of upper lobe, bronchus or lung | B222.00 |
| Pancoast's syndrome | B222.11 |
| Malignant neoplasm of upper lobe bronchus | B222000 |
| Malignant neoplasm of upper lobe of lung | B222100 |
| Malignant neoplasm of upper lobe, bronchus or lung NOS | B222z00 |
| Malignant neoplasm of middle lobe, bronchus or lung | B223.00 |
| Malignant neoplasm of middle lobe bronchus | B223000 |
| Malignant neoplasm of middle lobe of lung | B223100 |
| Malignant neoplasm of middle lobe, bronchus or lung NOS | B223z00 |
| Malignant neoplasm of lower lobe, bronchus or lung | B224.00 |
| Malignant neoplasm of lower lobe bronchus | B224000 |
| Malignant neoplasm of lower lobe of lung | B224100 |
| Malignant neoplasm of lower lobe, bronchus or lung NOS | B224z00 |
| Malignant neoplasm of overlapping lesion of bronchus & lung | B225.00 |
| Malignant neoplasm of other sites of bronchus or lung | B22y.00 |
| Malignant neoplasm of bronchus or lung NOS | B22z.00 |
| Lung cancer | B22z.11 |
| [M]Bronchiolo-alveolar adenocarcinoma | BB5S200 |
| [M]Alveolar cell carcinoma | BB5S211 |
| [M]Bronchiolar carcinoma | BB5S212 |
| [M]Alveolar adenocarcinoma | BB5S400 |
| [X]Malignant neoplasm of bronchus or lung, unspecified | Byu2000 |
| [V]Personal history of malig neop of trachea/bronchus/lung | ZV10100 |
| [V]Personal history of malignant neoplasm of bronchus | ZV10111 |
| [V]Personal history of malignant neoplasm of lung | ZV10112 |
| H/O Malignant melanoma | 1425000.0 |
| Breslow depth staging for melanoma | 4M3..00 |
| Clark melanoma level 1 | 4M70.00 |
| Clark melanoma level 2 | 4M71.00 |
| Clark melanoma level 3 | 4M72.00 |
| Clark melanoma level 4 | 4M73.00 |
| Clark melanoma level 5 | 4M74.00 |
| Excision of melanoma | 7G03J00 |
| Malignant melanoma of skin | B32..00 |
| Malignant melanoma of lip | B320.00 |
| Malignant melanoma of eyelid including canthus | B321.00 |
| Malignant melanoma of ear and external auricular canal | B322.00 |
| Malignant melanoma of auricle (ear) | B322000 |
| Malignant melanoma of external auditory meatus | B322100 |
| Malignant melanoma of ear and external auricular canal NOS | B322z00 |
| Malignant melanoma of other and unspecified parts of face | B323.00 |
| Malignant melanoma of external surface of cheek | B323000 |
| Malignant melanoma of chin | B323100 |
| Malignant melanoma of eyebrow | B323200 |
| Malignant melanoma of forehead | B323300 |
| Malignant melanoma of external surface of nose | B323400 |
| Malignant melanoma of temple | B323500 |
| Malignant melanoma of face NOS | B323z00 |
| Malignant melanoma of scalp and neck | B324.00 |
| Malignant melanoma of scalp | B324000 |
| Malignant melanoma of neck | B324100 |
| Malignant melanoma of scalp and neck NOS | B324z00 |
| Malignant melanoma of trunk (excluding scrotum) | B325.00 |
| Malignant melanoma of axilla | B325000 |
| Malignant melanoma of breast | B325100 |
| Malignant melanoma of buttock | B325200 |
| Malignant melanoma of groin | B325300 |
| Malignant melanoma of perianal skin | B325400 |
| Malignant melanoma of perineum | B325500 |
| Malignant melanoma of umbilicus | B325600 |
| Malignant melanoma of back | B325700 |
| Malignant melanoma of chest wall | B325800 |
| Malignant melanoma of trunk, excluding scrotum, NOS | B325z00 |
| Malignant melanoma of upper limb and shoulder | B326.00 |
| Malignant melanoma of shoulder | B326000 |
| Malignant melanoma of upper arm | B326100 |
| Malignant melanoma of fore-arm | B326200 |
| Malignant melanoma of hand | B326300 |
| Malignant melanoma of finger | B326400 |
| Malignant melanoma of thumb | B326500 |
| Malignant melanoma of upper limb or shoulder NOS | B326z00 |
| Malignant melanoma of lower limb and hip | B327.00 |
| Malignant melanoma of hip | B327000 |
| Malignant melanoma of thigh | B327100 |
| Malignant melanoma of knee | B327200 |
| Malignant melanoma of popliteal fossa area | B327300 |
| Malignant melanoma of lower leg | B327400 |
| Malignant melanoma of ankle | B327500 |
| Malignant melanoma of heel | B327600 |
| Malignant melanoma of foot | B327700 |
| Malignant melanoma of toe | B327800 |
| Malignant melanoma of great toe | B327900 |
| Malignant melanoma of lower limb or hip NOS | B327z00 |
| Malignant melanoma of other specified skin site | B32y.00 |
| Overlapping malignant melanoma of skin | B32y000 |
| Malignant melanoma of skin NOS | B32z.00 |
| [M]Malignant melanoma NOS | BBE1.00 |
| [M]Melanocarcinoma | BBE1.11 |
| [M]Melanoma NOS | BBE1.12 |
| [M]Melanosarcoma NOS | BBE1.13 |
| [M]Malignant melanoma, regressing | BBE1000 |
| [M]Desmoplastic melanoma, malignant | BBE1100 |
| [M]Nodular melanoma | BBE2.00 |
| [M]Balloon cell melanoma | BBE4.00 |
| [M]Amelanotic melanoma | BBEA.00 |
| [M]Malignant melanoma in junctional naevus | BBEC.00 |
| [M]Hutchinson's melanotic freckle | BBEF.00 |
| [M]Lentigo maligna | BBEF.11 |
| [M]Malignant melanoma in Hutchinson's melanotic freckle | BBEG.00 |
| [M]Lentigo maligna melanoma | BBEG.11 |
| [M]Acral lentiginous melanoma, malignant | BBEG000 |
| [M]Superficial spreading melanoma | BBEH.00 |
| [M]Malignant melanoma in giant pigmented naevus | BBEM.00 |
| [M]Juvenila melanoma | BBEN.11 |
| [M]Epithelioid cell melanoma | BBEP.00 |
| [M]Spindle cell melanoma NOS | BBEQ.00 |
| [M]Spindle cell melanoma, type B | BBES.00 |
| [M]Mixed epithelioid and spindle melanoma | BBET.00 |
| [X]Malignant melanoma of other+unspecified parts of face | Byu4000 |
| [X]Malignant melanoma of skin, unspecified | Byu4100 |
| [M]Mesothelioma, malignant | BBP1.00 |
| [M]Sarcomatoid mesothelioma | BBP3.11 |
| [M]Epithelioid mesothelioma, malignant | BBP5.00 |
| [M]Mesothelioma, biphasic type, malignant | BBP7.00 |
| Malignant neoplasms of independent (primary) multiple sites | B592.00 |
| [X]Malignant neoplasms/independent (primary) multiple sites | ByuE.00 |
| [X]Malignant neoplasms/independent(primary)multiple sites | ByuE000 |
| Malignant neoplasm of oesophagus | B10..00 |
| Malignant neoplasm of cervical oesophagus | B100.00 |
| Malignant neoplasm of thoracic oesophagus | B101.00 |
| Malignant neoplasm of abdominal oesophagus | B102.00 |
| Malignant neoplasm of upper third of oesophagus | B103.00 |
| Malignant neoplasm of middle third of oesophagus | B104.00 |
| Malignant neoplasm of lower third of oesophagus | B105.00 |
| Malignant neoplasm, overlapping lesion of oesophagus | B106.00 |
| Siewert type I adenocarcinoma | B107.00 |
| Malignant neoplasm of other specified part of oesophagus | B10y.00 |
| Malignant neoplasm of oesophagus NOS | B10z.00 |
| Oesophageal cancer | B10z.11 |
| [V]Personal history of malignant neoplasm of oesophagus | ZV10016 |
| Malignant neoplasm of lip, oral cavity and pharynx | B0...00 |
| Carcinoma of lip, oral cavity and pharynx | B0...11 |
| Malignant neoplasm of lip | B00..00 |
| Carcinoma of lip | B00..11 |
| Malignant neoplasm of upper lip, vermilion border | B000.00 |
| Malignant neoplasm of upper lip, external | B000000 |
| Malignant neoplasm of upper lip, lipstick area | B000100 |
| Malignant neoplasm of upper lip, vermilion border NOS | B000z00 |
| Malignant neoplasm of lower lip, vermilion border | B001.00 |
| Malignant neoplasm of lower lip, external | B001000 |
| Malignant neoplasm of lower lip, lipstick area | B001100 |
| Malignant neoplasm of lower lip, vermilion border NOS | B001z00 |
| Malignant neoplasm of upper lip, inner aspect | B002.00 |
| Malignant neoplasm of upper lip, frenulum | B002100 |
| Malignant neoplasm of upper lip, mucosa | B002200 |
| Malignant neoplasm of upper lip, oral aspect | B002300 |
| Malignant neoplasm of upper lip, inner aspect NOS | B002z00 |
| Malignant neoplasm of lower lip, inner aspect | B003.00 |
| Malignant neoplasm of lower lip, buccal aspect | B003000 |
| Malignant neoplasm of lower lip, frenulum | B003100 |
| Malignant neoplasm of lower lip, mucosa | B003200 |
| Malignant neoplasm of lower lip, oral aspect | B003300 |
| Malignant neoplasm of lower lip, inner aspect NOS | B003z00 |
| Malignant neoplasm of lip unspecified, inner aspect | B004.00 |
| Malignant neoplasm of lip unspecified, buccal aspect | B004000 |
| Malignant neoplasm of lip unspecified, mucosa | B004200 |
| Malignant neoplasm of lip, oral aspect | B004300 |
| Malignant neoplasm of commissure of lip | B005.00 |
| Malignant neoplasm of overlapping lesion of lip | B006.00 |
| Malignant neoplasm of lip, unspecified | B007.00 |
| Malignant neoplasm of lip, unspecified, external | B00z000 |
| Malignant neoplasm of lip, unspecified, lipstick area | B00z100 |
| Malignant neoplasm of lip, vermilion border NOS | B00zz00 |
| Malignant neoplasm of tongue | B01..00 |
| Malignant neoplasm of base of tongue | B010.00 |
| Malignant neoplasm of posterior third of tongue | B010.11 |
| Malignant neoplasm of base of tongue dorsal surface | B010000 |
| Malignant neoplasm of fixed part of tongue NOS | B010z00 |
| Malignant neoplasm of dorsal surface of tongue | B011.00 |
| Malignant neoplasm of midline of tongue | B011100 |
| Malignant neoplasm of dorsum of tongue NOS | B011z00 |
| Malignant neoplasm of tongue, tip and lateral border | B012.00 |
| Malignant neoplasm of ventral surface of tongue | B013.00 |
| Malignant neoplasm of anterior 2/3 of tongue ventral surface | B013000 |
| Malignant neoplasm of frenulum linguae | B013100 |
| Malignant neoplasm of ventral tongue surface NOS | B013z00 |
| Malignant neoplasm of anterior 2/3 of tongue unspecified | B014.00 |
| Malignant neoplasm of tongue, junctional zone | B015.00 |
| Malignant neoplasm of lingual tonsil | B016.00 |
| Malignant overlapping lesion of tongue | B017.00 |
| Malignant neoplasm of other sites of tongue | B01y.00 |
| Malignant neoplasm of tongue NOS | B01z.00 |
| Malignant neoplasm of major salivary glands | B02..00 |
| Malignant neoplasm of parotid gland | B020.00 |
| Malignant neoplasm of submandibular gland | B021.00 |
| Malignant neoplasm of sublingual gland | B022.00 |
| Malignant neoplasm of other major salivary glands | B02y.00 |
| Malignant neoplasm of major salivary gland NOS | B02z.00 |
| Malignant neoplasm of gum | B03..00 |
| Malignant neoplasm of upper gum | B030.00 |
| Malignant neoplasm of lower gum | B031.00 |
| Malignant neoplasm of other sites of gum | B03y.00 |
| Malignant neoplasm of gum NOS | B03z.00 |
| Malignant neoplasm of floor of mouth | B04..00 |
| Malignant neoplasm of anterior portion of floor of mouth | B040.00 |
| Malignant neoplasm of lateral portion of floor of mouth | B041.00 |
| Malignant neoplasm, overlapping lesion of floor of mouth | B042.00 |
| Malignant neoplasm of other sites of floor of mouth | B04y.00 |
| Malignant neoplasm of floor of mouth NOS | B04z.00 |
| Malignant neoplasm of other and unspecified parts of mouth | B05..00 |
| Malignant neoplasm of cheek mucosa | B050.00 |
| Malignant neoplasm of buccal mucosa | B050.11 |
| Malignant neoplasm of vestibule of mouth | B051.00 |
| Malignant neoplasm of upper buccal sulcus | B051000 |
| Malignant neoplasm of lower buccal sulcus | B051100 |
| Malignant neoplasm of hard palate | B052.00 |
| Malignant neoplasm of soft palate | B053.00 |
| Malignant neoplasm of uvula | B054.00 |
| Malignant neoplasm of palate unspecified | B055.00 |
| Malignant neoplasm of junction of hard and soft palate | B055000 |
| Malignant neoplasm of roof of mouth | B055100 |
| Malignant neoplasm of palate NOS | B055z00 |
| Malignant neoplasm of retromolar area | B056.00 |
| Malignant neoplasm of other specified mouth parts | B05y.00 |
| Malignant neoplasm of mouth NOS | B05z.00 |
| Malignant neoplasm of oropharynx | B06..00 |
| Malignant neoplasm of tonsil | B060.00 |
| Malignant neoplasm of faucial tonsil | B060000 |
| Malignant neoplasm of palatine tonsil | B060100 |
| Malignant neoplasm of overlapping lesion of tonsil | B060200 |
| Malignant neoplasm tonsil NOS | B060z00 |
| Malignant neoplasm of tonsillar fossa | B061.00 |
| Malignant neoplasm of tonsillar pillar | B062.00 |
| Malignant neoplasm of faucial pillar | B062000 |
| Malignant neoplasm of glossopalatine fold | B062100 |
| Malignant neoplasm of palatoglossal arch | B062200 |
| Malignant neoplasm of palatopharyngeal arch | B062300 |
| Malignant neoplasm of tonsillar fossa NOS | B062z00 |
| Malignant neoplasm of vallecula | B063.00 |
| Malignant neoplasm of anterior epiglottis | B064.00 |
| Malignant neoplasm of epiglottis, free border | B064000 |
| Malignant neoplasm of glossoepiglottic fold | B064100 |
| Malignant neoplasm of anterior epiglottis NOS | B064z00 |
| Malignant neoplasm of junctional region of epiglottis | B065.00 |
| Malignant neoplasm of lateral wall of oropharynx | B066.00 |
| Malignant neoplasm of posterior wall of oropharynx | B067.00 |
| Malignant neoplasm of oropharynx, other specified sites | B06y.00 |
| Malignant neoplasm of other specified site of oropharynx NOS | B06yz00 |
| Malignant neoplasm of oropharynx NOS | B06z.00 |
| Malignant neoplasm of nasopharynx | B07..00 |
| Malignant neoplasm of roof of nasopharynx | B070.00 |
| Malignant neoplasm of posterior wall of nasopharynx | B071.00 |
| Malignant neoplasm of adenoid | B071000 |
| Malignant neoplasm of pharyngeal tonsil | B071100 |
| Malignant neoplasm of posterior wall of nasopharynx NOS | B071z00 |
| Malignant neoplasm of lateral wall of nasopharynx | B072.00 |
| Malignant neoplasm of pharyngeal recess | B072000 |
| Malignant neoplasm of lateral wall of nasopharynx NOS | B072z00 |
| Malignant neoplasm of anterior wall of nasopharynx | B073.00 |
| Malignant neoplasm of nasopharyngeal soft palate surface | B073100 |
| Malignant neoplasm posterior margin nasal septum and choanae | B073200 |
| Malignant neoplasm of anterior wall of nasopharynx NOS | B073z00 |
| Malignant neoplasm, overlapping lesion of nasopharynx | B074.00 |
| Malignant neoplasm of other specified site of nasopharynx | B07y.00 |
| Malignant neoplasm of nasopharynx NOS | B07z.00 |
| Malignant neoplasm of hypopharynx | B08..00 |
| Malignant neoplasm of postcricoid region | B080.00 |
| Malignant neoplasm of pyriform sinus | B081.00 |
| Malignant neoplasm aryepiglottic fold, hypopharyngeal aspect | B082.00 |
| Malignant neoplasm of posterior pharynx | B083.00 |
| Malignant neoplasm of other specified hypopharyngeal site | B08y.00 |
| Malignant neoplasm of hypopharynx NOS | B08z.00 |
| Malig neop other/ill-defined sites lip, oral cavity, pharynx | B0z..00 |
| Malignant neoplasm of pharynx unspecified | B0z0.00 |
| Malignant neoplasm of Waldeyer's ring | B0z1.00 |
| Malignant neoplasm of laryngopharynx | B0z2.00 |
| Malignant neoplasm of other sites lip, oral cavity, pharynx | B0zy.00 |
| Malignant neoplasm of lip, oral cavity and pharynx NOS | B0zz.00 |
| [M]Basal cell adenocarcinoma | BB5y000 |
| [X]Malignant neoplasm of lip, oral cavity and pharynx | Byu0.00 |
| [V]Personal history of malignant neoplasm of tongue | ZV10019 |
| [V]Personal history of malignant neoplasm of tongue | ZV10y16 |
| Lymphoma stage I | 4M20.00 |
| Lymphoma stage II | 4M21.00 |
| Lymphoma stage III | 4M22.00 |
| Lymphoma stage IV | 4M23.00 |
| Excision malignant skin tumour | 7G03K00 |
| HIV disease resulting in Kaposi's sarcoma | A789500 |
| HIV disease resulting in Kaposi sarcoma | A789511 |
| Malignant neoplasm of small intestine and duodenum | B12..00 |
| Malignant neoplasm of duodenum | B120.00 |
| Malignant neoplasm of jejunum | B121.00 |
| Malignant neoplasm of ileum | B122.00 |
| Malignant neoplasm of Meckel's diverticulum | B123.00 |
| Malignant neoplasm, overlapping lesion of small intestine | B124.00 |
| Malignant neoplasm of other specified site small intestine | B12y.00 |
| Malignant neoplasm of small intestine NOS | B12z.00 |
| Malignant neoplasm of liver and intrahepatic bile ducts | B15..00 |
| Malignant neoplasm of liver and intrahepatic bile ducts NOS | B15z.00 |
| Malignant neoplasm gallbladder and extrahepatic bile ducts | B16..00 |
| Malignant neoplasm of gallbladder | B160.00 |
| Carcinoma gallbladder | B160.11 |
| Malignant neoplasm other gallbladder/extrahepatic bile duct | B16y.00 |
| Malignant neoplasm gallbladder/extrahepatic bile ducts NOS | B16z.00 |
| Malignant neoplasm of retroperitoneum and peritoneum | B18..00 |
| Malignant neoplasm of retroperitoneum | B180.00 |
| Malignant neoplasm of perinephric tissue | B180100 |
| Malignant neoplasm of retrocaecal tissue | B180200 |
| Malignant neoplasm of retroperitoneum NOS | B180z00 |
| Overlapping malign lesion of retroperitoneum and peritoneum | B182.00 |
| Malignant neoplasm of specified parts of peritoneum | B18y.00 |
| Malignant neoplasm of mesocaecum | B18y100 |
| Malignant neoplasm of mesorectum | B18y200 |
| Malignant neoplasm of omentum | B18y300 |
| Malignant neoplasm of parietal peritoneum | B18y400 |
| Malignant neoplasm of pelvic peritoneum | B18y500 |
| Malignant neoplasm of the pouch of Douglas | B18y600 |
| Malignant neoplasm of mesentery | B18y700 |
| Malignant neoplasm of specified parts of peritoneum NOS | B18yz00 |
| Malignant neoplasm of retroperitoneum and peritoneum NOS | B18z.00 |
| Malig neop oth/ill-defined sites digestive tract/peritoneum | B1z..00 |
| Malignant neoplasm of intestinal tract, part unspecified | B1z0.00 |
| Malignant neoplasm of spleen NEC | B1z1.00 |
| Angiosarcoma of spleen | B1z1000 |
| Fibrosarcoma of spleen | B1z1100 |
| Malignant neoplasm of spleen NOS | B1z1z00 |
| Malignant neoplasm, overlapping lesion of digestive system | B1z2.00 |
| Malignant neoplasm other spec digestive tract and peritoneum | B1zy.00 |
| Malignant neoplasm of digestive tract and peritoneum NOS | B1zz.00 |
| Malig neop nasal cavities, middle ear and accessory sinuses | B20..00 |
| Malignant neoplasm of nasal cavities | B200.00 |
| Malignant neoplasm of cartilage of nose | B200000 |
| Malignant neoplasm of nasal conchae | B200100 |
| Malignant neoplasm of septum of nose | B200200 |
| Malignant neoplasm of vestibule of nose | B200300 |
| Malignant neoplasm of nasal cavities NOS | B200z00 |
| Malig neop auditory tube, middle ear and mastoid air cells | B201.00 |
| Malignant neoplasm of auditory (Eustachian) tube | B201000 |
| Malignant neoplasm of tympanic cavity | B201100 |
| Malignant neoplasm of tympanic antrum | B201200 |
| Malignant neoplasm of mastoid air cells | B201300 |
| Malig neop auditory tube, middle ear, mastoid air cells NOS | B201z00 |
| Malignant neoplasm of maxillary sinus | B202.00 |
| Malignant neoplasm of ethmoid sinus | B203.00 |
| Malignant neoplasm of frontal sinus | B204.00 |
| Malignant neoplasm of sphenoidal sinus | B205.00 |
| Malignant neoplasm, overlapping lesion of accessory sinuses | B206.00 |
| Malig neop other site nasal cavity, middle ear and sinuses | B20y.00 |
| Malignant neoplasm of accessory sinus NOS | B20z.00 |
| Malignant neoplasm of larynx | B21..00 |
| Malignant neoplasm of glottis | B210.00 |
| Malignant neoplasm of supraglottis | B211.00 |
| Malignant neoplasm of subglottis | B212.00 |
| Malignant neoplasm of laryngeal cartilage | B213.00 |
| Malignant neoplasm of arytenoid cartilage | B213000 |
| Malignant neoplasm of cricoid cartilage | B213100 |
| Malignant neoplasm of cuneiform cartilage | B213200 |
| Malignant neoplasm of thyroid cartilage | B213300 |
| Malignant neoplasm of laryngeal cartilage NOS | B213z00 |
| Malignant neoplasm, overlapping lesion of larynx | B214.00 |
| Malignant neoplasm of epiglottis NOS | B215.00 |
| Malignant neoplasm of larynx, other specified site | B21y.00 |
| Malignant neoplasm of larynx NOS | B21z.00 |
| Malignant neoplasm of pleura | B23..00 |
| Malignant neoplasm of parietal pleura | B230.00 |
| Malignant neoplasm of visceral pleura | B231.00 |
| Malignant neoplasm of other specified pleura | B23y.00 |
| Malignant neoplasm of pleura NOS | B23z.00 |
| Malignant neoplasm of thymus, heart and mediastinum | B24..00 |
| Malignant neoplasm of thymus | B240.00 |
| Malignant neoplasm of heart | B241.00 |
| Malignant neoplasm of endocardium | B241000 |
| Malignant neoplasm of myocardium | B241200 |
| Malignant neoplasm of pericardium | B241300 |
| Malignant neoplasm of heart NOS | B241z00 |
| Malignant neoplasm of anterior mediastinum | B242.00 |
| Malignant neoplasm of posterior mediastinum | B243.00 |
| Malignant neoplasm of mediastinum, part unspecified | B24X.00 |
| Malig neop of other site of heart, thymus and mediastinum | B24y.00 |
| Malignant neoplasm of heart, thymus and mediastinum NOS | B24z.00 |
| Malig neo, overlapping lesion of heart, mediastinum & pleura | B25..00 |
| Malignant neoplasm, overlap lesion of resp & intrathor orgs | B26..00 |
| Malig neop other/ill-defined sites resp/intrathoracic organs | B2z..00 |
| Malig neop of upper respiratory tract, part unspecified | B2z0.00 |
| Malignant neoplasm of other site of respiratory tract | B2zy.00 |
| Malignant neoplasm of respiratory tract NOS | B2zz.00 |
| Malig neop of connective and soft tissue head, face and neck | B310.00 |
| Malignant neoplasm of soft tissue of head | B310000 |
| Malignant neoplasm of soft tissue of face | B310100 |
| Malignant neoplasm of soft tissue of neck | B310200 |
| Malignant neoplasm of cartilage of ear | B310300 |
| Malignant neoplasm of tarsus of eyelid | B310400 |
| Malignant neoplasm soft tissues of cervical spine | B310500 |
| Malig neop connective and soft tissue head, face, neck NOS | B310z00 |
| Malig neop connective and soft tissue upper limb/shoulder | B311.00 |
| Malignant neoplasm of connective and soft tissue of shoulder | B311000 |
| Malignant neoplasm of connective and soft tissue, upper arm | B311100 |
| Malignant neoplasm of connective and soft tissue of fore-arm | B311200 |
| Malignant neoplasm of connective and soft tissue of hand | B311300 |
| Malignant neoplasm of connective and soft tissue of finger | B311400 |
| Malignant neoplasm of connective and soft tissue of thumb | B311500 |
| Malig neop connective soft tissue upper limb/shoulder NOS | B311z00 |
| Malig neop of connective and soft tissue of hip and leg | B312.00 |
| Malignant neoplasm of connective and soft tissue of hip | B312000 |
| Malig neop of connective and soft tissue thigh and upper leg | B312100 |
| Malig neop connective and soft tissue of popliteal space | B312200 |
| Malig neop of connective and soft tissue of lower leg | B312300 |
| Malignant neoplasm of connective and soft tissue of foot | B312400 |
| Malignant neoplasm of connective and soft tissue of toe | B312500 |
| Malig neop connective and soft tissue hip and leg NOS | B312z00 |
| Malignant neoplasm of connective and soft tissue of thorax | B313.00 |
| Malignant neoplasm of connective and soft tissue of axilla | B313000 |
| Malignant neoplasm of diaphragm | B313100 |
| Malignant neoplasm of great vessels | B313200 |
| Malig neoplasm of connective and soft tissues of thor spine | B313300 |
| Malig neop of connective and soft tissue of thorax NOS | B313z00 |
| Malignant neoplasm of connective and soft tissue of abdomen | B314.00 |
| Malig neop of connective and soft tissue of abdominal wall | B314000 |
| Malig neoplasm of connective and soft tissues of lumb spine | B314100 |
| Malig neop of connective and soft tissue of abdomen NOS | B314z00 |
| Malignant neoplasm of connective and soft tissue of pelvis | B315.00 |
| Malignant neoplasm of connective and soft tissue of buttock | B315000 |
| Malig neop of connective and soft tissue of inguinal region | B315100 |
| Malignant neoplasm of connective and soft tissue of perineum | B315200 |
| Malig neopl of connective and soft tissue - sacrum or coccyx | B315300 |
| Malig neop of connective and soft tissue of pelvis NOS | B315z00 |
| Malig neop of connective and soft tissue trunk unspecified | B316.00 |
| Malig neop connective and soft tissue other specified site | B31y.00 |
| Malignant neoplasm of connective and soft tissue, site NOS | B31z.00 |
| Kaposi's sarcoma of soft tissue | B31z000 |
| Malignant neoplasm of placenta | B42..00 |
| Choriocarcinoma | B420.00 |
| Malignant neoplasm of ovary and other uterine adnexa | B44..00 |
| Malignant neoplasm of fallopian tube | B441.00 |
| Malignant neoplasm of broad ligament | B442.00 |
| Malignant neoplasm of parametrium | B443.00 |
| Malignant neoplasm of other site of uterine adnexa | B44y.00 |
| Malignant neoplasm of uterine adnexa NOS | B44z.00 |
| Malig neop of other and unspecified female genital organs | B45..00 |
| Malignant neoplasm of vagina | B450.00 |
| Malignant neoplasm of vaginal vault | B450100 |
| Malignant neoplasm of vagina NOS | B450z00 |
| Malignant neoplasm of labia majora | B451.00 |
| Malignant neoplasm of greater vestibular (Bartholin's) gland | B451000 |
| Malignant neoplasm of labia majora NOS | B451z00 |
| Malignant neoplasm of labia minora | B452.00 |
| Malignant neoplasm of clitoris | B453.00 |
| Malignant neoplasm of vulva unspecified | B454.00 |
| Primary vulval cancer | B454.11 |
| Malignant neoplasm/overlapping lesion/feml genital organs | B45X.00 |
| Malignant neoplasm of other specified female genital organ | B45y.00 |
| Malignant neoplasm of overlapping lesion of vulva | B45y000 |
| Malignant neoplasm of female genital organ NOS | B45z.00 |
| Malignant neoplasm of prepuce (foreskin) | B480.00 |
| Malignant neoplasm of glans penis | B481.00 |
| Malignant neoplasm of body of penis | B482.00 |
| Malignant neoplasm of penis, part unspecified | B483.00 |
| Malignant neoplasm of epididymis | B484.00 |
| Malignant neoplasm of spermatic cord | B485.00 |
| Malignant neoplasm of scrotum | B486.00 |
| Malignant neoplasm, overlapping lesion of penis | B487.00 |
| Malignant neoplasm of other male genital organ | B48y.00 |
| Malignant neoplasm of seminal vesicle | B48y000 |
| Malignant neoplasm of tunica vaginalis | B48y100 |
| Malignant neoplasm, overlapping lesion male genital orgs | B48y200 |
| Malignant neoplasm of other male genital organ NOS | B48yz00 |
| Malignant neoplasm of penis and other male genital organ NOS | B48z.00 |
| Malig neop of kidney and other unspecified urinary organs | B4A..00 |
| Malignant neoplasm of urethra | B4A3.00 |
| Malignant neoplasm of paraurethral glands | B4A4.00 |
| Malignant neoplasm of other urinary organs | B4Ay.00 |
| Malignant neoplasm of overlapping lesion of urinary organs | B4Ay000 |
| Malignant neoplasm of kidney or urinary organs NOS | B4Az.00 |
| Malignant neoplasm of other and unspecified sites | B5...00 |
| Carcinoma of other and unspecified sites | B5...11 |
| Malignant neoplasm of eye | B50..00 |
| Malig neop eyeball excl conjunctiva, cornea, retina, choroid | B500.00 |
| Malignant neoplasm of ciliary body | B500000 |
| Malignant neoplasm of iris | B500100 |
| Malignant neoplasm of crystalline lens | B500200 |
| Malignant neoplasm of eyeball NOS | B500z00 |
| Malignant neoplasm of orbit | B501.00 |
| Malignant neoplasm of connective tissue of orbit | B501000 |
| Malignant neoplasm of orbit NOS | B501z00 |
| Malignant neoplasm of lacrimal gland | B502.00 |
| Malignant neoplasm of conjunctiva | B503.00 |
| Malignant neoplasm of cornea | B504.00 |
| Malignant neoplasm of retina | B505.00 |
| Malignant neoplasm of choroid | B506.00 |
| Malignant neoplasm of lacrimal duct | B507.00 |
| Malignant neoplasm of lacrimal sac | B507000 |
| Malignant neoplasm of nasolacrimal duct | B507100 |
| Malignant neoplasm, overlapping lesion of eye and adnexa | B508.00 |
| Malignant neoplasm of other specified site of eye | B50y.00 |
| Malignant neoplasm of eye NOS | B50z.00 |
| Malig neop of other and unspecified parts of nervous system | B52..00 |
| Malig neopl peripheral nerves and autonomic nervous system | B524.00 |
| Malignant neoplasm of peripheral nerves of head, face & neck | B524000 |
| Malignant neoplasm of peripheral nerve,upp limb,incl should | B524100 |
| Malignant neoplasm of peripheral nerve of low limb, incl hip | B524200 |
| Malignant neoplasm of peripheral nerve of thorax | B524300 |
| Malignant neoplasm of peripheral nerve of abdomen | B524400 |
| Malignant neoplasm of peripheral nerve of pelvis | B524500 |
| Malignant neoplasm,overlap lesion periph nerve & auton ns | B524600 |
| Mal neoplasm/periph nerves+autonomic nervous system,unspc | B524W00 |
| Malignant neoplasm of other specified part of nervous system | B52y.00 |
| Malignant neoplasm of nervous system NOS | B52z.00 |
| Malignant neoplasm of adrenal gland | B540.00 |
| Phaeochromocytoma | B540.11 |
| Malignant neoplasm of adrenal cortex | B540000 |
| Malignant neoplasm of adrenal medulla | B540100 |
| Malignant neoplasm of adrenal gland NOS | B540z00 |
| Malignant neoplasm of parathyroid gland | B541.00 |
| Malignant neoplasm-pluriglandular involvement,unspecified | B54X.00 |
| Malignant neoplasm of other specified endocrine gland | B54y.00 |
| Malig neop of endocrine gland or related structure NOS | B54z.00 |
| Malignant neoplasm of head, neck and face | B550.00 |
| Malignant neoplasm of head NOS | B550000 |
| Malignant neoplasm of cheek NOS | B550100 |
| Malignant neoplasm of nose NOS | B550200 |
| Malignant neoplasm of jaw NOS | B550300 |
| Malignant neoplasm of neck NOS | B550400 |
| Malignant neoplasm of supraclavicular fossa NOS | B550500 |
| Malignant neoplasm of head, neck and face NOS | B550z00 |
| Malignant neoplasm of thorax | B551.00 |
| Malignant neoplasm of axilla NOS | B551000 |
| Malignant neoplasm of chest wall NOS | B551100 |
| Malignant neoplasm of intrathoracic site NOS | B551200 |
| Malignant neoplasm of thorax NOS | B551z00 |
| Malignant neoplasm of abdomen | B552.00 |
| Malignant neoplasm of pelvis | B553.00 |
| Malignant neoplasm of inguinal region NOS | B553000 |
| Malignant neoplasm of presacral region | B553100 |
| Malignant neoplasm of sacrococcygeal region | B553200 |
| Malignant neoplasm of pelvis NOS | B553z00 |
| Malignant neoplasm of upper limb NOS | B554.00 |
| Malignant neoplasm of lower limb NOS | B555.00 |
| Malignant neoplasm of other specified sites | B55y.00 |
| Malignant neoplasm of back NOS | B55y000 |
| Malignant neoplasm of trunk NOS | B55y100 |
| Malignant neoplasm of flank NOS | B55y200 |
| Malignant neoplasm of specified site NOS | B55yz00 |
| Malignant neoplasm of other and ill defined site NOS | B55z.00 |
| Malignant neoplasm of unspecified site | B59..00 |
| Other malignant neoplasm NOS | B591.00 |
| Kaposi's sarcoma of multiple organs | B592X00 |
| Primary malignant neoplasm of unknown site | B593.00 |
| Malignant tumour of unknown origin | B595.00 |
| Malignant neoplasm of unspecified site NOS | B59z.00 |
| Kaposi's sarcoma, unspecified | B59zX00 |
| Malignant neoplasm of other and unspecified site OS | B5y..00 |
| Malignant neoplasm of other and unspecified site NOS | B5z..00 |
| Malignant histiocytosis | B623.00 |
| Malignant histiocytosis of unspecified site | B623000 |
| Malignant histiocytosis of lymph nodes head, face and neck | B623100 |
| Malignant histiocytosis of intra-abdominal lymph nodes | B623300 |
| Malignant histiocytosis NOS | B623z00 |
| Letterer-Siwe disease | B625.00 |
| Histiocytosis X (acute, progressive) | B625.11 |
| Letterer-Siwe disease of unspecified sites | B625000 |
| Letterer-Siwe disease of intrathoracic lymph nodes | B625200 |
| Letterer-Siwe disease of lymph nodes of multiple sites | B625800 |
| Letterer-Siwe disease NOS | B625z00 |
| Malignant mast cell tumours | B626.00 |
| Mast cell malignancy of unspecified site | B626000 |
| Mast cell malignancy of lymph nodes inguinal region and leg | B626500 |
| Mast cell malignancy of lymph nodes of multiple sites | B626800 |
| Malignant mast cell tumour NOS | B626z00 |
| Malignant immunoproliferative small intestinal disease | B62x500 |
| True histiocytic lymphoma | B62x600 |
| Malignant neoplasms of lymphoid and histiocytic tissue NOS | B62z.00 |
| Unspec malig neop lymphoid/histiocytic of unspecified site | B62z000 |
| Unspec malig neop lymphoid/histiocytic lymph node head/neck | B62z100 |
| Unspec malig neop lymphoid/histiocytic of intrathoracic node | B62z200 |
| Unspec malig neop lymphoid/histiocytic intra-abdominal nodes | B62z300 |
| Unspec malig neop lymphoid/histiocytic lymph node axilla/arm | B62z400 |
| Unspec malig neop lymphoid/histiocytic nodes inguinal/leg | B62z500 |
| Unspec malig neop lymphoid/histiocytic of intrapelvic nodes | B62z600 |
| Unspec malig neop lymphoid/histiocytic of multiple sites | B62z800 |
| Lymphoid and histiocytic malignancy NOS | B62zz00 |
| Immunoproliferative neoplasm | B62zz11 |
| Other immunoproliferative neoplasms | B63y.00 |
| Immunoproliferative neoplasm or myeloma NOS | B63z.00 |
| Malignant neoplasm lymphatic or haematopoietic tissue OS | B6y..00 |
| Malignant neoplasm lymphatic or haematopoietic tissue NOS | B6z..00 |
| Kaposi's sarcoma of lymph nodes | B6z0.00 |
| [M]Adenocarcinoma, intestinal type | BB57.00 |
| [M]Adrenal cortical carcinoma | BB5h100 |
| [M]Haemangiosarcoma | BBT1.00 |
| [M]Haemangioendothelioma, malignant | BBT7100 |
| [M]Aesthesioneuroblastoma | BBcC.00 |
| [M]Olfactory neuroblastoma | BBcC.11 |
| [M]Olfactory neuroepithelioma | BBcD.11 |
| [M]True histiocytic lymphoma | BBm4.00 |
| [X]Malignant neoplasm of intestinal tract, part unspecified | Byu1200 |
| [X]Malignant neoplsm/ill-defin sites within digestive system | Byu1300 |
| [X]Malignant neoplasm/ill-defined sites within resp system | Byu2400 |
| [X]Malignant neoplasm of mediastinum, part unspecified | Byu2500 |
| [X]Melanoma and other malignant neoplasms of skin | Byu4.00 |
| [X]Kaposi's sarcoma, unspecified | Byu5300 |
| [X]Malignant neoplasm/peripheral nerves of trunk,unspecified | Byu5400 |
| [X]Mal neoplasm/overlap les/periph nerv+autonomic nerv systm | Byu5500 |
| [X]Malignant neoplasm of peritoneum, unspecified | Byu5700 |
| [X]Mal neoplasm/connective+soft tissue of trunk,unspecified | Byu5800 |
| [X]Malignant neoplasm/connective + soft tissue,unspecified | Byu5900 |
| [X]Kaposi's sarcoma of other sites | Byu5B00 |
| [X]Malignant neoplasm of uterine adnexa, unspecified | Byu7000 |
| [X]Malignant neoplasm/other specified female genital organs | Byu7100 |
| [X]Malignant neoplasm of female genital organ, unspecified | Byu7300 |
| [X]Malignant neoplasm/other specified male genital organs | Byu8000 |
| [X]Malignant neoplasm of male genital organ, unspecified | Byu8200 |
| [X]Malignant neoplasm of urinary organ, unspecified | Byu9000 |
| [X]Malignant neoplasm of thyroid and other endocrine glands | ByuB.00 |
| [X]Malignant neoplasm of endocrine gland, unspecified | ByuB100 |
| [X]Malignant neoplasm of ill-defined, secondary and unspeci | ByuC.00 |
| [X]Malignant neoplasm of other specified sites | ByuC000 |
| [X]Malignant neoplasm/overlap lesion/other+ill-defined sites | ByuC100 |
| [X]2ndry malignant neoplasm/oth+unspec parts/nervous system | ByuC600 |
| [X]Malignant neoplasm without specification of site | ByuC800 |
| [X]Other malignant immunoproliferative diseases | ByuD400 |
| [X]Oth spcf mal neoplsm/lymphoid,haematopoietic+rltd tissue | ByuDA00 |
| [X]Mal neoplasm/lymphoid,haematopoietic+related tissu,unspcf | ByuDB00 |
| Hand - Schuller - Christian disease | C37y000 |
| Eosinophilic granuloma | C37y100 |
| Histiocytosis X , chronic | C37y500 |
| Histiocytosis X , unspecified | C37y600 |
| [V]Personal history of malig neop of gastrointestinal tract | ZV10000 |
| [V]Personal history of malig neop of gastrointestinal tract | ZV10012 |
| Malignant neoplasm of ovary | B440.00 |
| Cancer of ovary | B440.11 |
| [M]Endometrioid carcinoma | BB5j200 |
| [M]Endometrioid adenofibroma, malignant | BB5j500 |
| [M]Struma ovarii, malignant | BBQA100 |
| Anaemia in ovarian carcinoma | D212000 |
| [V]Personal history of malignant neoplasm of ovary | ZV10414 |
| Malignant neoplasm of pancreas | B17..00 |
| Malignant neoplasm of head of pancreas | B170.00 |
| Malignant neoplasm of body of pancreas | B171.00 |
| Malignant neoplasm of tail of pancreas | B172.00 |
| Malignant neoplasm of pancreatic duct | B173.00 |
| Malignant neoplasm of Islets of Langerhans | B174.00 |
| Malignant neoplasm, overlapping lesion of pancreas | B175.00 |
| Malignant neoplasm of other specified sites of pancreas | B17y.00 |
| Malignant neoplasm of ectopic pancreatic tissue | B17y000 |
| Malignant neoplasm of specified site of pancreas NOS | B17yz00 |
| Malignant neoplasm of pancreas NOS | B17z.00 |
| [M]Islet cell carcinoma | BB5B100 |
| [M]Insulinoma, malignant | BB5B300 |
| [M]Glucagonoma, malignant | BB5B500 |
| [M]Mixed islet cell and exocrine adenocarcinoma | BB5B600 |
| [M]Gastrinoma, malignant | BB5C100 |
| H/O: prostate cancer | 1427000.0 |
| Gleason grading of prostate cancer | 4M0..00 |
| Gleason prostate grade 2-4 (low) | 4M00.00 |
| Gleason prostate grade 5-7 (medium) | 4M01.00 |
| Gleason prostate grade 8-10 (high) | 4M02.00 |
| Radical cystoprostatourethrectomy | 7B20000 |
| Radical cystoprostatectomy | 7B20200 |
| Radical prostatectomy - unspecified excision of pelvic nodes | 7B36000 |
| Radical prostatectomy without pelvic node excision | 7B36500 |
| Radical prostatectomy with pelvic node sampling | 7B36600 |
| Radical prostatectomy with pelvic lymphadenectomy | 7B36700 |
| Malignant neoplasm of prostate | B46..00 |
| [V]Personal history of malignant neoplasm of prostate | ZV10415 |
| Excision biopsy of rodent ulcer | 7G05600 |
| Excision biopsy of basal cell carcinoma | 7G05D00 |
| Other malignant neoplasm of skin | B33..00 |
| Basal cell carcinoma | B33..11 |
| Rodent ulcer | B33..13 |
| Malignant neoplasm of sebaceous gland | B33..14 |
| Malignant neoplasm of sweat gland | B33..15 |
| Epithelioma basal cell | B33..16 |
| Malignant neoplasm of skin of lip | B330.00 |
| Malignant neoplasm of eyelid including canthus | B331.00 |
| Malignant neoplasm of canthus | B331000 |
| Malignant neoplasm of upper eyelid | B331100 |
| Malignant neoplasm of lower eyelid | B331200 |
| Malignant neoplasm skin of ear and external auricular canal | B332.00 |
| Malignant neoplasm of skin of auricle (ear) | B332000 |
| Malignant neoplasm of skin of external auditory meatus | B332100 |
| Malignant neoplasm of pinna NEC | B332200 |
| Malig neop skin of ear and external auricular canal NOS | B332z00 |
| Malignant neoplasm skin of other and unspecified parts face | B333.00 |
| Malignant neoplasm of skin of cheek, external | B333000 |
| Malignant neoplasm of skin of chin | B333100 |
| Malignant neoplasm of skin of eyebrow | B333200 |
| Malignant neoplasm of skin of forehead | B333300 |
| Malignant neoplasm of skin of nose (external) | B333400 |
| Malignant neoplasm of skin of temple | B333500 |
| Malignant neoplasm skin other and unspec part of face NOS | B333z00 |
| Malignant neoplasm of scalp and skin of neck | B334.00 |
| Malignant neoplasm of scalp | B334000 |
| Malignant neoplasm of skin of neck | B334100 |
| Malignant neoplasm of scalp or skin of neck NOS | B334z00 |
| Malignant neoplasm of skin of trunk, excluding scrotum | B335.00 |
| Malignant neoplasm of skin of axillary fold | B335000 |
| Malignant neoplasm of skin of chest, excluding breast | B335100 |
| Malignant neoplasm of skin of breast | B335200 |
| Malignant neoplasm of skin of abdominal wall | B335300 |
| Malignant neoplasm of skin of umbilicus | B335400 |
| Malignant neoplasm of skin of groin | B335500 |
| Malignant neoplasm of skin of perineum | B335600 |
| Malignant neoplasm of skin of back | B335700 |
| Malignant neoplasm of skin of buttock | B335800 |
| Malignant neoplasm of perianal skin | B335900 |
| Malignant neoplasm of skin of scapular region | B335A00 |
| Malignant neoplasm of skin of trunk, excluding scrotum, NOS | B335z00 |
| Malignant neoplasm of skin of upper limb and shoulder | B336.00 |
| Malignant neoplasm of skin of shoulder | B336000 |
| Malignant neoplasm of skin of upper arm | B336100 |
| Malignant neoplasm of skin of fore-arm | B336200 |
| Malignant neoplasm of skin of hand | B336300 |
| Malignant neoplasm of skin of finger | B336400 |
| Malignant neoplasm of skin of thumb | B336500 |
| Malignant neoplasm of skin of upper limb or shoulder NOS | B336z00 |
| Malignant neoplasm of skin of lower limb and hip | B337.00 |
| Malignant neoplasm of skin of hip | B337000 |
| Malignant neoplasm of skin of thigh | B337100 |
| Malignant neoplasm of skin of knee | B337200 |
| Malignant neoplasm of skin of popliteal fossa area | B337300 |
| Malignant neoplasm of skin of lower leg | B337400 |
| Malignant neoplasm of skin of ankle | B337500 |
| Malignant neoplasm of skin of heel | B337600 |
| Malignant neoplasm of skin of foot | B337700 |
| Malignant neoplasm of skin of toe | B337800 |
| Malignant neoplasm of skin of great toe | B337900 |
| Malignant neoplasm of skin of lower limb or hip NOS | B337z00 |
| Squamous cell carcinoma of skin | B338.00 |
| Dermatofibrosarcoma protuberans | B339.00 |
| Malignant neoplasm overlapping lesion of skin | B33X.00 |
| Malignant neoplasm of other specified skin sites | B33y.00 |
| Malignant neoplasm of skin NOS | B33z.00 |
| Squamous cell carcinoma of skin NOS | B33z.11 |
| [M]Squamous cell carcinoma of skin NOS | BB2A.13 |
| [M]Basal cell neoplasms | BB3..00 |
| [M]Basal cell tumour | BB30.00 |
| [M]Basal cell carcinoma NOS | BB31.00 |
| [M]Multicentric basal cell carcinoma | BB32.00 |
| [M]Basal cell carcinoma, morphoea type | BB33.00 |
| [M]Basal cell carcinoma, fibroepithelial type | BB34.00 |
| [M]Basosquamous carcinoma | BB35.00 |
| [M]Metatypical carcinoma | BB36.00 |
| [M]Superficial basal cell carcinoma | BB3C.00 |
| [M]Basal cell carcinoma, nodular | BB3D.00 |
| [M]Basal cell carcinoma, micronodular | BB3E.00 |
| [M]Basal cell carcinoma, infiltrative | BB3F.00 |
| [M]Pigmented basal cell carcinoma | BB3G.00 |
| [M]Basal cell neoplasm NOS | BB3z.00 |
| [M]Skin appendage carcinoma | BB60100 |
| [M]Sweat gland adenocarcinoma | BB61200 |
| [M]Apocrine adenocarcinoma | BB62100 |
| [M]Sebaceous adenocarcinoma | BB69100 |
| [X]Oth malignant neoplasm/skin of oth+unspecfd parts of face | Byu4200 |
| [X]Malignant neoplasm of skin, unspecified | Byu4300 |
| [X]Malignant neoplasm overlapping lesion of skin | Byu5A00 |
| [V]Personal history of malignant neoplasm of skin | ZV10y14 |
| Malignant neoplasm of stomach | B11..00 |
| Malignant neoplasm of cardia of stomach | B110.00 |
| Malignant neoplasm of cardiac orifice of stomach | B110000 |
| Malignant neoplasm of cardio-oesophageal junction of stomach | B110100 |
| Malignant neoplasm of gastro-oesophageal junction | B110111 |
| Malignant neoplasm of cardia of stomach NOS | B110z00 |
| Malignant neoplasm of pylorus of stomach | B111.00 |
| Malignant neoplasm of prepylorus of stomach | B111000 |
| Malignant neoplasm of pyloric canal of stomach | B111100 |
| Malignant neoplasm of pylorus of stomach NOS | B111z00 |
| Malignant neoplasm of pyloric antrum of stomach | B112.00 |
| Malignant neoplasm of fundus of stomach | B113.00 |
| Malignant neoplasm of body of stomach | B114.00 |
| Malignant neoplasm of lesser curve of stomach unspecified | B115.00 |
| Malignant neoplasm of greater curve of stomach unspecified | B116.00 |
| Malignant neoplasm, overlapping lesion of stomach | B117.00 |
| Siewert type II adenocarcinoma | B118.00 |
| Siewert type III adenocarcinoma | B119.00 |
| Malignant neoplasm of other specified site of stomach | B11y.00 |
| Malignant neoplasm of anterior wall of stomach NEC | B11y000 |
| Malignant neoplasm of posterior wall of stomach NEC | B11y100 |
| Malignant neoplasm of other specified site of stomach NOS | B11yz00 |
| Malignant neoplasm of stomach NOS | B11z.00 |
| [V]Personal history of malignant neoplasm of stomach | ZV10018 |
| Malignant neoplasm of testis | B47..00 |
| Malignant neoplasm of undescended testis | B470.00 |
| Seminoma of undescended testis | B470200 |
| Teratoma of undescended testis | B470300 |
| Malignant neoplasm of undescended testis NOS | B470z00 |
| Malignant neoplasm of descended testis | B471.00 |
| Seminoma of descended testis | B471000 |
| Teratoma of descended testis | B471100 |
| Malignant neoplasm of descended testis NOS | B471z00 |
| Malignant neoplasm of testis NOS | B47z.00 |
| Seminoma of testis | B47z.11 |
| Teratoma of testis | B47z.12 |
| [V]Personal history of malignant neoplasm of testis | ZV10416 |
| Malignant neoplasm of thyroid gland | B53..00 |
| [V]Personal history of malignant neoplasm of thyroid | ZV10y15 |
| Malignant neoplasm of uterus, part unspecified | B40..00 |
| Malignant neoplasm of body of uterus | B43..00 |
| Malignant neoplasm of corpus uteri, excluding isthmus | B430.00 |
| Malignant neoplasm of cornu of corpus uteri | B430000 |
| Malignant neoplasm of fundus of corpus uteri | B430100 |
| Malignant neoplasm of endometrium of corpus uteri | B430200 |
| Malignant neoplasm of endometrium | B430211 |
| Malignant neoplasm of myometrium of corpus uteri | B430300 |
| Malignant neoplasm of corpus uteri NOS | B430z00 |
| Malignant neoplasm of isthmus of uterine body | B431.00 |
| Malignant neoplasm of lower uterine segment | B431000 |
| Malignant neoplasm of isthmus of uterine body NOS | B431z00 |
| Malignant neoplasm of overlapping lesion of corpus uteri | B432.00 |
| Malignant neoplasm of other site of uterine body | B43y.00 |
| Malignant neoplasm of body of uterus NOS | B43z.00 |
| [V]Personal history of malignant neoplasm of uterine body | ZV10417 |
| Secondary and unspecified malignant neoplasm of lymph nodes | B56..00 |
| Lymph node metastases | B56..11 |
| Secondary and unspec malig neop lymph nodes head/face/neck | B560.00 |
| Secondary and unspec malig neop of superficial parotid LN | B560000 |
| Secondary and unspec malignant neoplasm mastoid lymph nodes | B560100 |
| Secondary and unspec malig neop superficial cervical LN | B560200 |
| Secondary and unspec malignant neoplasm occipital lymph node | B560300 |
| Secondary and unspec malig neop deep parotid lymph nodes | B560400 |
| Secondary and unspec malig neop submandibular lymph nodes | B560500 |
| Secondary and unspec malig neop of facial lymph nodes | B560600 |
| Secondary and unspec malig neop submental lymph nodes | B560700 |
| Secondary and unspec malig neop anterior cervical LN | B560800 |
| Secondary and unspec malig neop deep cervical LN | B560900 |
| Secondary unspec malig neop lymph nodes head/face/neck NOS | B560z00 |
| Secondary and unspec malig neop intrathoracic lymph nodes | B561.00 |
| Secondary and unspec malig neop internal mammary lymph nodes | B561000 |
| Secondary and unspec malig neop intercostal lymph nodes | B561100 |
| Secondary and unspec malig neop diaphragmatic lymph nodes | B561200 |
| Secondary and unspec malig neop ant mediastinal lymph nodes | B561300 |
| Secondary and unspec malig neop post mediastinal lymph nodes | B561400 |
| Secondary and unspec malig neop paratracheal lymph nodes | B561500 |
| Secondary and unspec malig neop superfic tracheobronchial LN | B561600 |
| Secondary and unspec malig neop inferior tracheobronchial LN | B561700 |
| Secondary and unspec malig neop bronchopulmonary lymph nodes | B561800 |
| Secondary and unspec malig neop pulmonary lymph nodes | B561900 |
| Secondary and unspec malig neop intrathoracic LN NOS | B561z00 |
| Secondary and unspec malig neop intra-abdominal lymph nodes | B562.00 |
| Secondary and unspec malig neop coeliac lymph nodes | B562000 |
| Secondary and unspec malig neop superficial mesenteric LN | B562100 |
| Secondary and unspec malig neop inferior mesenteric LN | B562200 |
| Secondary and unspec malig neop common iliac lymph nodes | B562300 |
| Secondary and unspec malig neop external iliac lymph nodes | B562400 |
| Secondary and unspec malig neop intra-abdominal LN NOS | B562z00 |
| Secondary and unspec malig neop axilla and upper limb LN | B563.00 |
| Secondary and unspec malig neop axillary lymph nodes | B563000 |
| Secondary and unspec malig neop supratrochlear lymph nodes | B563100 |
| Secondary and unspec malig neop infraclavicular lymph nodes | B563200 |
| Secondary and unspec malig neop pectoral lymph nodes | B563300 |
| Secondary and unspec malig neop axilla and upper limb LN NOS | B563z00 |
| Secondary and unspec malig neop inguinal and lower limb LN | B564.00 |
| Secondary and unspec malig neop superficial inguinal LN | B564000 |
| Secondary and unspec malig neop deep inguinal lymph nodes | B564100 |
| Secondary and unspec malig neop of inguinal and leg LN NOS | B564z00 |
| Secondary and unspec malig neop intrapelvic lymph nodes | B565.00 |
| Secondary and unspec malig neop internal iliac lymph nodes | B565000 |
| Secondary and unspec malig neop circumflex iliac LN | B565200 |
| Secondary and unspec malig neop sacral lymph nodes | B565300 |
| Secondary and unspec malig neop intrapelvic LN NOS | B565z00 |
| Secondary and unspec malig neop lymph nodes multiple sites | B56y.00 |
| Secondary and unspec malig neop lymph nodes NOS | B56z.00 |
| [X]2ndry+unspcf malignant neoplasm lymph nodes/multi regions | ByuC200 |
| Secondary malignant neoplasm of adrenal gland | B587.00 |
| Secondary malignant neoplasm of bone and bone marrow | B585.00 |
| Pathological fracture due to metastatic bone disease | B585000 |
| Secondary malignant neoplasm of small intestine and duodenum | B574.00 |
| Secondary malignant neoplasm of duodenum | B574000 |
| Secondary malignant neoplasm of jejunum | B574100 |
| Secondary malignant neoplasm of ileum | B574200 |
| Secondary malig neop of small intestine or duodenum NOS | B574z00 |
| Secondary malignant neoplasm of large intestine and rectum | B575.00 |
| Secondary malignant neoplasm of colon | B575000 |
| Secondary malignant neoplasm of rectum | B575100 |
| Secondary malig neop of large intestine or rectum NOS | B575z00 |
| Secondary malignant neoplasm of brain and spinal cord | B583.00 |
| Secondary malignant neoplasm of brain | B583000 |
| Secondary malignant neoplasm of spinal cord | B583100 |
| Cerebral metastasis | B583200 |
| Secondary malignant neoplasm of brain or spinal cord NOS | B583z00 |
| Secondary malignant neoplasm of lung | B570.00 |
| Pain from metastases | 1D18.00 |
| X-ray metastasis control | 5136.00 |
| Secondary malig neop of respiratory and digestive systems | B57..00 |
| Metastases of respiratory and/or digestive systems | B57..11 |
| Secondary carcinoma of respiratory and/or digestive systems | B57..12 |
| Secondary malignant neoplasm of mediastinum | B571.00 |
| Secondary malignant neoplasm of other respiratory organs | B573.00 |
| Secondary malignant neoplasm of other digestive organ | B57y.00 |
| Secondary malig neop of respiratory or digestive system NOS | B57z.00 |
| Secondary malignant neoplasm of other specified sites | B58..00 |
| Secondary carcinoma of other specified sites | B58..11 |
| Secondary malignant neoplasm of kidney | B580.00 |
| Secondary malignant neoplasm of other urinary organs | B581.00 |
| Secondary malignant neoplasm of ureter | B581000 |
| Secondary malignant neoplasm of bladder | B581100 |
| Secondary malignant neoplasm of urethra | B581200 |
| Secondary malignant neoplasm of other urinary organ NOS | B581z00 |
| Secondary malignant neoplasm of skin | B582.00 |
| Secondary malignant neoplasm of skin of head | B582000 |
| Secondary malignant neoplasm of skin of face | B582100 |
| Secondary malignant neoplasm of skin of neck | B582200 |
| Secondary malignant neoplasm of skin of trunk | B582300 |
| Secondary malignant neoplasm of skin of shoulder and arm | B582400 |
| Secondary malignant neoplasm of skin of hip and leg | B582500 |
| Secondary malignant neoplasm of skin of breast | B582600 |
| Secondary malignant neoplasm of skin NOS | B582z00 |
| Secondary malignant neoplasm of other part of nervous system | B584.00 |
| Secondary malignant neoplasm of ovary | B586.00 |
| Secondary malignant neoplasm of other specified sites | B58y.00 |
| Secondary malignant neoplasm of breast | B58y000 |
| Secondary malignant neoplasm of uterus | B58y100 |
| Secondary malignant neoplasm of cervix uteri | B58y200 |
| Secondary cancer of the cervix | B58y211 |
| Secondary malignant neoplasm of vagina | B58y300 |
| Secondary malignant neoplasm of vulva | B58y400 |
| Secondary cancer of the vulva | B58y411 |
| Secondary malignant neoplasm of prostate | B58y500 |
| Secondary malignant neoplasm of testis | B58y600 |
| Secondary malignant neoplasm of penis | B58y700 |
| Secondary malignant neoplasm of epididymis and vas deferens | B58y800 |
| Secondary malignant neoplasm of tongue | B58y900 |
| Secondary malignant neoplasm of other specified site NOS | B58yz00 |
| Secondary malignant neoplasm of other specified site NOS | B58z.00 |
| Disseminated malignancy NOS | B590.00 |
| Carcinomatosis | B590.11 |
| Secondary malignant neoplasm of unknown site | B594.00 |
| [M]Neoplasm, metastatic | BB03.00 |
| [M]Secondary neoplasm | BB03.11 |
| [M]Carcinoma, metastatic, NOS | BB13.00 |
| [M]Secondary carcinoma | BB13.11 |
| [M]Squamous cell carcinoma, metastatic NOS | BB2B.00 |
| [M]Adenocarcinoma, metastatic, NOS | BB53.00 |
| [M]Metastatic signet ring cell carcinoma | BB85100 |
| [M]No microscopic confirmation tumour, clinically metastatic | BBy2.00 |
| [X]Secondary malignant neoplasm/oth+unspc respiratory organs | ByuC300 |
| [X]Secondary malignant neoplasm/oth+unspcfd digestive organs | ByuC400 |
| [X]2ndry malignant neoplasm/bladder+oth+unsp urinary organs | ByuC500 |
| [X]2ndry malignant neoplasm/oth+unspec parts/nervous system | ByuC600 |
| [X]Secondary malignant neoplasm of other specified sites | ByuC700 |
| Secondary malig neop of retroperitoneum and peritoneum | B576.00 |
| Secondary malignant neoplasm of retroperitoneum | B576000 |
| Secondary malignant neoplasm of peritoneum | B576100 |
| Malignant ascites | B576200 |
| Secondary malig neop of retroperitoneum or peritoneum NOS | B576z00 |
| Secondary malignant neoplasm of pleura | B572.00 |

# Smoking

| **description** | **code** | **category** |
| --- | --- | --- |
| Never smoked tobacco | 1371.00 | Non smoker |
| Non-smoker | 1371.11 | Non smoker |
| Current non-smoker | 137L.00 | Non smoker |
| No smokers in the household | 13WK.00 | Non smoker |
| Non-smoker annual review - enhanced services administration | 9kn..00 | Non smoker |
| Ex-trivial smoker (<1/day) | 1377.00 | Ex smoker |
| Ex-light smoker (1-9/day) | 1378.00 | Ex smoker |
| Ex-moderate smoker (10-19/day) | 1379.00 | Ex smoker |
| Ex-heavy smoker (20-39/day) | 137A.00 | Ex smoker |
| Ex-very heavy smoker (40+/day) | 137B.00 | Ex smoker |
| Ex-smoker - amount unknown | 137F.00 | Ex smoker |
| Stopped smoking | 137K.00 | Ex smoker |
| Ex pipe smoker | 137N.00 | Ex smoker |
| Ex cigar smoker | 137O.00 | Ex smoker |
| Ex smoker | 137S.00 | Ex smoker |
| Date ceased smoking | 137T.00 | Ex smoker |
| Ex-cigarette smoker | 137j.00 | Ex smoker |
| Ex-smoker annual review - enhanced services administration | 9km..00 | Ex smoker |
| Tobacco dependence in remission | E251300 | Ex smoker |
| Smokers' cough | H310100 | Ex or current smoker (Unknown) |
| [V]Personal history of tobacco abuse | ZV11600 | Ex or current smoker (Unknown) |
| Smoker - amount smoked | 137..11 | Current smoker |
| Trivial smoker - < 1 cig/day | 1372.00 | Current smoker |
| Occasional smoker | 1372.11 | Current smoker |
| Light smoker - 1-9 cigs/day | 1373.00 | Current smoker |
| Moderate smoker - 10-19 cigs/d | 1374.00 | Current smoker |
| Heavy smoker - 20-39 cigs/day | 1375.00 | Current smoker |
| Very heavy smoker - 40+cigs/d | 1376.00 | Current smoker |
| Keeps trying to stop smoking | 137C.00 | Current smoker |
| Trying to give up smoking | 137G.00 | Current smoker |
| Pipe smoker | 137H.00 | Current smoker |
| Cigar smoker | 137J.00 | Current smoker |
| Rolls own cigarettes | 137M.00 | Current smoker |
| Cigarette smoker | 137P.00 | Current smoker |
| Smoker | 137P.11 | Current smoker |
| Smoking started | 137Q.00 | Current smoker |
| Smoking restarted | 137Q.11 | Current smoker |
| Current smoker | 137R.00 | Current smoker |
| Smoking reduced | 137V.00 | Current smoker |
| Cigarette consumption | 137X.00 | Current smoker |
| Cigar consumption | 137Y.00 | Current smoker |
| Tobacco consumption NOS | 137Z.00 | Current smoker |
| Pipe tobacco consumption | 137a.00 | Current smoker |
| Ready to stop smoking | 137b.00 | Current smoker |
| Thinking about stopping smoking | 137c.00 | Current smoker |
| Not interested in stopping smoking | 137d.00 | Current smoker |
| Smoking restarted | 137e.00 | Current smoker |
| Reason for restarting smoking | 137f.00 | Current smoker |
| Minutes from waking to first tobacco consumption | 137h.00 | Current smoker |
| Smoking cessation milestones | 13p..00 | Current smoker |
| Negotiated date for cessation of smoking | 13p0.00 | Current smoker |
| Smoking status at 4 weeks | 13p1.00 | Current smoker |
| Smoking status between 4 and 52 weeks | 13p2.00 | Current smoker |
| Smoking status at 52 weeks | 13p3.00 | Current smoker |
| Smoking free weeks | 13p4.00 | Current smoker |
| Smoking cessation programme start date | 13p5.00 | Current smoker |
| Carbon monoxide reading at 4 weeks | 13p6.00 | Current smoker |
| Fagerstrom test for nicotine dependence | 38DH.00 | Current smoker |
| Pregnancy smoking advice | 67A3.00 | Current smoker |
| Brief intervention for smoking cessation | 67H6.00 | Current smoker |
| Smoking cessation therapy | 745H.00 | Current smoker |
| Nicotine replacement therapy using nicotine patches | 745H000 | Current smoker |
| Nicotine replacement therapy using nicotine gum | 745H100 | Current smoker |
| Nicotine replacement therapy using nicotine inhalator | 745H200 | Current smoker |
| Nicotine replacement therapy using nicotine lozenges | 745H300 | Current smoker |
| Smoking cessation drug therapy | 745H400 | Current smoker |
| Other specified smoking cessation therapy | 745Hy00 | Current smoker |
| Smoking cessation therapy NOS | 745Hz00 | Current smoker |
| Nicotine replacement therapy | 8B2B.00 | Current smoker |
| Over the counter nicotine replacement therapy | 8B3Y.00 | Current smoker |
| Nicotine replacement therapy provided free | 8B3f.00 | Current smoker |
| Nicotine replacement therapy provided by community pharmacis | 8BP3.00 | Current smoker |
| Smoking cessation advice | 8CAL.00 | Current smoker |
| Smoking cessation advice provided by community pharmacist | 8CAg.00 | Current smoker |
| Referral to smoking cessation advisor | 8H7i.00 | Current smoker |
| Stop smoking face to face follow-up | 8HBM.00 | Current smoker |
| Referral to stop-smoking clinic | 8HTK.00 | Current smoker |
| Referral to NHS stop smoking service | 8HkQ.00 | Current smoker |
| Nicotine replacement therapy contraindicated | 8I2I.00 | Current smoker |
| Bupropion contraindicated | 8I2J.00 | Current smoker |
| Nicotine replacement therapy refused | 8I39.00 | Current smoker |
| Bupropion refused | 8I3M.00 | Current smoker |
| Seen by smoking cessation advisor | 9N2k.00 | Current smoker |
| DNA - Did not attend smoking cessation clinic | 9N4M.00 | Current smoker |
| Anti-smoking monitoring admin. | 9OO..00 | Current smoker |
| Stop smoking clinic admin. | 9OO..11 | Current smoker |
| Stop smoking monitoring admin. | 9OO..12 | Current smoker |
| Attends stop smoking monitor. | 9OO1.00 | Current smoker |
| Refuses stop smoking monitor | 9OO2.00 | Current smoker |
| Stop smoking monitor default | 9OO3.00 | Current smoker |
| Stop smoking monitor 1st lettr | 9OO4.00 | Current smoker |
| Stop smoking monitor 2nd lettr | 9OO5.00 | Current smoker |
| Stop smoking monitor 3rd lettr | 9OO6.00 | Current smoker |
| Stop smoking monitor verb.inv. | 9OO7.00 | Current smoker |
| Stop smoking monitor phone inv | 9OO8.00 | Current smoker |
| Stop smoking monitoring delete | 9OO9.00 | Current smoker |
| Stop smoking monitor.chck done | 9OOA.00 | Current smoker |
| Stop smoking monitor admin.NOS | 9OOZ.00 | Current smoker |
| Smoking cessation - enhanced services administration | 9kc..00 | Current smoker |
| Smoking cessatn monitor template complet - enhanc serv admin | 9kc0.00 | Current smoker |
| Current smoker annual review - enhanced services admin | 9ko..00 | Current smoker |
| Nicotine withdrawal | E023.00 | Current smoker |
| Tobacco dependence | E251.00 | Current smoker |
| Tobacco dependence, unspecified | E251000 | Current smoker |
| Tobacco dependence, continuous | E251100 | Current smoker |
| Tobacco dependence NOS | E251z00 | Current smoker |
| [X]Mental and behavioural disorder due to use of tobacco | Eu17.00 | Current smoker |
| [X]Mental and behav dis due to use of tobacco: harmful use | Eu17100 | Current smoker |
| Advice on smoking | ZG23300 | Current smoker |
| Fagerstrom test for nicotine dependence | ZRBm200 | Current smoker |
| FTND - Fagerstrom test for nicotine dependence | ZRBm211 | Current smoker |
| Motives for smoking scale | ZRaM.00 | Current smoker |
| Occasions for smoking scale | ZRao.00 | Current smoker |
| Reasons for smoking scale | ZRh4.00 | Current smoker |
| RFS - Reasons for smoking scale | ZRh4.11 | Current smoker |
| [V]Tobacco use | ZV4K000 | Current smoker |
| [V]Tobacco abuse counselling | ZV6D800 | Current smoker |

# Supplementary Table S10: Analgesic drug codes

| **drugcode** | **description** | **class** |
| --- | --- | --- |
| 99824992 | Amitriptyline 100 mg tab | antidepressant |
| 94077990 | Amitriptyline 10mg tablets | antidepressant |
| 96326979 | Amitriptyline 10mg tablets | antidepressant |
| 96328979 | Amitriptyline 10mg tablets | antidepressant |
| 97223998 | Amitriptyline 10mg tablets | antidepressant |
| 99861990 | Amitriptyline 10mg tablets | antidepressant |
| 99863990 | Amitriptyline 10mg tablets | antidepressant |
| 99864990 | Amitriptyline 10mg tablets | antidepressant |
| 99866990 | Amitriptyline 10mg tablets | antidepressant |
| 99868990 | Amitriptyline 10mg tablets | antidepressant |
| 99869990 | Amitriptyline 10mg tablets | antidepressant |
| 99870990 | Amitriptyline 10mg tablets | antidepressant |
| 99871990 | Amitriptyline 10mg tablets | antidepressant |
| 81085998 | Amitriptyline 10mg/5ml oral solution | antidepressant |
| 47944978 | Amitriptyline 10mg/5ml oral solution sugar free | antidepressant |
| 81084998 | Amitriptyline 10mg/5ml oral suspension | antidepressant |
| 92808996 | Amitriptyline 10mg/5ml sugar free oral solution | antidepressant |
| 98067988 | Amitriptyline 10mg/5ml sugar free oral solution | antidepressant |
| 98128998 | Amitriptyline 10mg/5ml sugar free oral solution | antidepressant |
| 96924998 | Amitriptyline 10mg/ml injection | antidepressant |
| 70292979 | Amitriptyline 15mg/5ml oral solution | antidepressant |
| 70290979 | Amitriptyline 2.5mg/5ml oral solution | antidepressant |
| 99826992 | Amitriptyline 200 mg tab | antidepressant |
| 96925998 | Amitriptyline 25mg modified-release capsules | antidepressant |
| 94076990 | Amitriptyline 25mg tablets | antidepressant |
| 94374990 | Amitriptyline 25mg tablets | antidepressant |
| 94771990 | Amitriptyline 25mg tablets | antidepressant |
| 96323979 | Amitriptyline 25mg tablets | antidepressant |
| 97223997 | Amitriptyline 25mg tablets | antidepressant |
| 99861989 | Amitriptyline 25mg tablets | antidepressant |
| 99862990 | Amitriptyline 25mg tablets | antidepressant |
| 99863989 | Amitriptyline 25mg tablets | antidepressant |
| 99864989 | Amitriptyline 25mg tablets | antidepressant |
| 99865990 | Amitriptyline 25mg tablets | antidepressant |
| 99866989 | Amitriptyline 25mg tablets | antidepressant |
| 99867989 | Amitriptyline 25mg tablets | antidepressant |
| 99868989 | Amitriptyline 25mg tablets | antidepressant |
| 99869988 | Amitriptyline 25mg tablets | antidepressant |
| 99870989 | Amitriptyline 25mg tablets | antidepressant |
| 99871989 | Amitriptyline 25mg tablets | antidepressant |
| 26539978 | Amitriptyline 25mg/5ml oral solution sugar free | antidepressant |
| 92808997 | Amitriptyline 25mg/5ml oral solution sugar free | antidepressant |
| 96891992 | Amitriptyline 25mg/5ml oral solution sugar free | antidepressant |
| 98067990 | Amitriptyline 25mg/5ml oral solution sugar free | antidepressant |
| 99825992 | Amitriptyline 300 mg tab | antidepressant |
| 96925997 | Amitriptyline 50mg modified-release capsules | antidepressant |
| 94075990 | Amitriptyline 50mg tablets | antidepressant |
| 96319979 | Amitriptyline 50mg tablets | antidepressant |
| 97223996 | Amitriptyline 50mg tablets | antidepressant |
| 99863988 | Amitriptyline 50mg tablets | antidepressant |
| 99864988 | Amitriptyline 50mg tablets | antidepressant |
| 99866988 | Amitriptyline 50mg tablets | antidepressant |
| 99868988 | Amitriptyline 50mg tablets | antidepressant |
| 99869989 | Amitriptyline 50mg tablets | antidepressant |
| 99870988 | Amitriptyline 50mg tablets | antidepressant |
| 99871988 | Amitriptyline 50mg tablets | antidepressant |
| 92808998 | Amitriptyline 50mg/5ml oral solution sugar free | antidepressant |
| 98067989 | Amitriptyline 50mg/5ml oral solution sugar free | antidepressant |
| 81026979 | Amitriptyline 5mg/5ml oral solution | antidepressant |
| 81024979 | Amitriptyline 5mg/5ml oral suspension | antidepressant |
| 94067992 | Amitriptyline 75 mg tab | antidepressant |
| 96925996 | Amitriptyline 75mg modified-release capsules | antidepressant |
| 98129998 | Amitriptyline hydrochloride 100mg/10ml injection | antidepressant |
| 98130998 | Amitriptyline hydrochloride 10mg tablets | antidepressant |
| 98150998 | Amitriptyline hydrochloride 10mg tablets | antidepressant |
| 98138998 | Amitriptyline hydrochloride 25mg modified release capsules | antidepressant |
| 98130997 | Amitriptyline hydrochloride 25mg tablets | antidepressant |
| 98150997 | Amitriptyline hydrochloride 25mg tablets | antidepressant |
| 98138997 | Amitriptyline hydrochloride 50mg modified release capsules | antidepressant |
| 98130996 | Amitriptyline hydrochloride 50mg tablets | antidepressant |
| 98150996 | Amitriptyline hydrochloride 50mg tablets | antidepressant |
| 98129997 | Amitriptyline hydrochloride 75mg modified release capsules | antidepressant |
| 96942992 | Ammonium chlor.& morphine double strengt mix | strong_opioid |
| 96943992 | Ammonium chloride & morphine mix | strong_opioid |
| 96950992 | Aspirin & codeine 75 mg tab | weak_opioid |
| 96111992 | Aspirin & codeine paed 75 mg tab | weak_opioid |
| 95861992 | Aspirin & paracetamol tab | weak_nsaid |
| 93318998 | Aspirin 300mg / paracetamol 200mg dispersible tablets sugar free | weak_nsaid |
| 94612998 | Aspirin 500mg / Codeine 8mg dispersible tablets sugar free | weak_opioid |
| 96585992 | Aspirin 500mg / Codeine 8mg dispersible tablets sugar free | weak_opioid |
| 99737992 | Aspirin/codeine phosphate/paracetamol 250 mg tab | weak_opioid |
| 99737992 | Aspirin/codeine phosphate/paracetamol 250 mg tab | weak_opioid |
| 99899992 | Aspirin/codeine phosphate/paracetamol 300 mg tab | weak_opioid |
| 99899992 | Aspirin/codeine phosphate/paracetamol 300 mg tab | weak_opioid |
| 99751992 | Aspirin/paracetamol tab | weak_nsaid |
| 98394990 | Bismuth with morphine mixture | strong_opioid |
| 19045978 | Buprenorphine 10micrograms/hour transdermal patches | strong_opioid |
| 21437978 | Buprenorphine 10micrograms/hour transdermal patches | strong_opioid |
| 22980978 | Buprenorphine 10micrograms/hour transdermal patches | strong_opioid |
| 29528978 | Buprenorphine 10micrograms/hour transdermal patches | strong_opioid |
| 29947978 | Buprenorphine 10micrograms/hour transdermal patches | strong_opioid |
| 29948978 | Buprenorphine 10micrograms/hour transdermal patches | strong_opioid |
| 38530978 | Buprenorphine 10micrograms/hour transdermal patches | strong_opioid |
| 53094979 | Buprenorphine 10micrograms/hour transdermal patches | strong_opioid |
| 86613998 | Buprenorphine 10micrograms/hour transdermal patches | strong_opioid |
| 86616998 | Buprenorphine 10micrograms/hour transdermal patches | strong_opioid |
| 29733978 | Buprenorphine 15micrograms/hour transdermal patches | strong_opioid |
| 25998978 | Buprenorphine 15micrograms/hour transdermal patches | strong_opioid |
| 37791978 | Buprenorphine 15micrograms/hour transdermal patches | strong_opioid |
| 37792978 | Buprenorphine 15micrograms/hour transdermal patches | strong_opioid |
| 14545978 | Buprenorphine 16mg/0.32ml prolonged-release solution for injection pre-filled syringes | strong_opioid |
| 21438978 | Buprenorphine 20micrograms/hour transdermal patches | strong_opioid |
| 22981978 | Buprenorphine 20micrograms/hour transdermal patches | strong_opioid |
| 29530978 | Buprenorphine 20micrograms/hour transdermal patches | strong_opioid |
| 29949978 | Buprenorphine 20micrograms/hour transdermal patches | strong_opioid |
| 29950978 | Buprenorphine 20micrograms/hour transdermal patches | strong_opioid |
| 38531978 | Buprenorphine 20micrograms/hour transdermal patches | strong_opioid |
| 53095979 | Buprenorphine 20micrograms/hour transdermal patches | strong_opioid |
| 86612998 | Buprenorphine 20micrograms/hour transdermal patches | strong_opioid |
| 86615998 | Buprenorphine 20micrograms/hour transdermal patches | strong_opioid |
| 14543978 | Buprenorphine 24mg/0.48ml prolonged-release solution for injection pre-filled syringes | strong_opioid |
| 85404998 | Buprenorphine 300micrograms/1ml solution for injection ampoules | strong_opioid |
| 85405998 | Buprenorphine 300micrograms/1ml solution for injection ampoules | strong_opioid |
| 99070998 | Buprenorphine 300micrograms/1ml solution for injection ampoules | strong_opioid |
| 19119978 | Buprenorphine 35micrograms/hour transdermal patches | strong_opioid |
| 21234978 | Buprenorphine 35micrograms/hour transdermal patches | strong_opioid |
| 24846978 | Buprenorphine 35micrograms/hour transdermal patches | strong_opioid |
| 29311978 | Buprenorphine 35micrograms/hour transdermal patches | strong_opioid |
| 33916978 | Buprenorphine 35micrograms/hour transdermal patches | strong_opioid |
| 33917978 | Buprenorphine 35micrograms/hour transdermal patches | strong_opioid |
| 55720978 | Buprenorphine 35micrograms/hour transdermal patches | strong_opioid |
| 79704978 | Buprenorphine 35micrograms/hour transdermal patches | strong_opioid |
| 86733979 | Buprenorphine 35micrograms/hour transdermal patches | strong_opioid |
| 86734979 | Buprenorphine 35micrograms/hour transdermal patches | strong_opioid |
| 86735979 | Buprenorphine 35micrograms/hour transdermal patches | strong_opioid |
| 89304998 | Buprenorphine 35micrograms/hour transdermal patches | strong_opioid |
| 91029998 | Buprenorphine 35micrograms/hour transdermal patches | strong_opioid |
| 21233978 | Buprenorphine 52.5micrograms/hour transdermal patches | strong_opioid |
| 24845978 | Buprenorphine 52.5micrograms/hour transdermal patches | strong_opioid |
| 29310978 | Buprenorphine 52.5micrograms/hour transdermal patches | strong_opioid |
| 31603978 | Buprenorphine 52.5micrograms/hour transdermal patches | strong_opioid |
| 33915978 | Buprenorphine 52.5micrograms/hour transdermal patches | strong_opioid |
| 46271978 | Buprenorphine 52.5micrograms/hour transdermal patches | strong_opioid |
| 79703978 | Buprenorphine 52.5micrograms/hour transdermal patches | strong_opioid |
| 86730979 | Buprenorphine 52.5micrograms/hour transdermal patches | strong_opioid |
| 88976998 | Buprenorphine 52.5micrograms/hour transdermal patches | strong_opioid |
| 91721998 | Buprenorphine 52.5micrograms/hour transdermal patches | strong_opioid |
| 19047978 | Buprenorphine 5micrograms/hour transdermal patches | strong_opioid |
| 21439978 | Buprenorphine 5micrograms/hour transdermal patches | strong_opioid |
| 22982978 | Buprenorphine 5micrograms/hour transdermal patches | strong_opioid |
| 29532978 | Buprenorphine 5micrograms/hour transdermal patches | strong_opioid |
| 29951978 | Buprenorphine 5micrograms/hour transdermal patches | strong_opioid |
| 29952978 | Buprenorphine 5micrograms/hour transdermal patches | strong_opioid |
| 38532978 | Buprenorphine 5micrograms/hour transdermal patches | strong_opioid |
| 53096979 | Buprenorphine 5micrograms/hour transdermal patches | strong_opioid |
| 86614998 | Buprenorphine 5micrograms/hour transdermal patches | strong_opioid |
| 86617998 | Buprenorphine 5micrograms/hour transdermal patches | strong_opioid |
| 24844978 | Buprenorphine 70micrograms/hour transdermal patches | strong_opioid |
| 33914978 | Buprenorphine 70micrograms/hour transdermal patches | strong_opioid |
| 79702978 | Buprenorphine 70micrograms/hour transdermal patches | strong_opioid |
| 86722979 | Buprenorphine 70micrograms/hour transdermal patches | strong_opioid |
| 88975998 | Buprenorphine 70micrograms/hour transdermal patches | strong_opioid |
| 91522998 | Buprenorphine 70micrograms/hour transdermal patches | strong_opioid |
| 14537978 | Buprenorphine 8mg/0.16ml prolonged-release solution for injection pre-filled syringes | strong_opioid |
| 95933997 | Buprenorphine hcl 300micrograms injection | strong_opioid |
| 96701998 | Chlormezanone with paracetamol tablets | paracetamol |
| 98863990 | Chloroform and morphine tincture | strong_opioid |
| 80426979 | Codeine 10mg/5ml oral solution | weak_opioid |
| 80424979 | Codeine 10mg/5ml oral suspension | weak_opioid |
| 54657979 | Codeine 15mg suppositories | weak_opioid |
| 91748990 | Codeine 15mg tablets | weak_opioid |
| 93054990 | Codeine 15mg tablets | weak_opioid |
| 93309992 | Codeine 15mg tablets | weak_opioid |
| 95205990 | Codeine 15mg tablets | weak_opioid |
| 95600979 | Codeine 15mg tablets | weak_opioid |
| 95602979 | Codeine 15mg tablets | weak_opioid |
| 96953990 | Codeine 15mg tablets | weak_opioid |
| 98634988 | Codeine 15mg tablets | weak_opioid |
| 98922998 | Codeine 15mg tablets | weak_opioid |
| 99639990 | Codeine 15mg tablets | weak_opioid |
| 99640990 | Codeine 15mg tablets | weak_opioid |
| 99651990 | Codeine 15mg tablets | weak_opioid |
| 98921998 | Codeine 25mg/5ml oral solution | weak_opioid |
| 99292989 | Codeine 25mg/5ml oral solution | weak_opioid |
| 99638990 | Codeine 25mg/5ml oral solution | weak_opioid |
| 58275979 | Codeine 30mg suppositories | weak_opioid |
| 52664979 | Codeine 30mg tablets | weak_opioid |
| 92087990 | Codeine 30mg tablets | weak_opioid |
| 95594979 | Codeine 30mg tablets | weak_opioid |
| 95605990 | Codeine 30mg tablets | weak_opioid |
| 96953989 | Codeine 30mg tablets | weak_opioid |
| 98045990 | Codeine 30mg tablets | weak_opioid |
| 98574990 | Codeine 30mg tablets | weak_opioid |
| 98634990 | Codeine 30mg tablets | weak_opioid |
| 98922997 | Codeine 30mg tablets | weak_opioid |
| 99639988 | Codeine 30mg tablets | weak_opioid |
| 99640989 | Codeine 30mg tablets | weak_opioid |
| 99651989 | Codeine 30mg tablets | weak_opioid |
| 98094997 | Codeine 30mg/1ml solution for injection ampoules | weak_opioid |
| 69926979 | Codeine 30mg/5ml oral solution | weak_opioid |
| 80420979 | Codeine 5mg/5ml oral suspension | weak_opioid |
| 86979998 | Codeine 6.75mg/5ml oral solution | weak_opioid |
| 98922996 | Codeine 60mg tablets | weak_opioid |
| 99639989 | Codeine 60mg tablets | weak_opioid |
| 99640988 | Codeine 60mg tablets | weak_opioid |
| 99651988 | Codeine 60mg tablets | weak_opioid |
| 98094998 | Codeine 60mg/1ml solution for injection ampoules | weak_opioid |
| 97188992 | Codeine 8mg & paracetamol 500mg sup | weak_opioid |
| 97188992 | Codeine 8mg & paracetamol 500mg sup | weak_opioid |
| 89898998 | Codeine 8mg with aspirin 500mg soluble tablets | weak_opioid |
| 97181992 | Codeine co soluble tab | weak_opioid |
| 95895992 | Codeine co tab | weak_opioid |
| 95852992 | Codeine phos/ibuprofen sr (20mg/300mg) tab | weak_opioid |
| 95852992 | Codeine phos/ibuprofen sr (20mg/300mg) tab | weak_opioid |
| 99518998 | Codeine phosphate & kaolin 10mg+3g/10ml mixture | weak_opioid |
| 96165992 | Codeine phosphate / magnesium chloride mg mix | weak_opioid |
| 96997997 | Codeine phosphate 15mg/5ml diabetic oral solution | weak_opioid |
| 98553997 | Codeine phosphate 30mg with paracetamol 500mg effervescent powder sugar free | weak_opioid |
| 98553997 | Codeine phosphate 30mg with paracetamol 500mg effervescent powder sugar free | weak_opioid |
| 98553998 | Codeine phosphate 60mg with paracetamol 1000mg effervescent powder sugar free | weak_opioid |
| 98553998 | Codeine phosphate 60mg with paracetamol 1000mg effervescent powder sugar free | weak_opioid |
| 85166998 | Codeine phosphate oral solution | weak_opioid |
| 98856990 | Codeine phosphate powder | weak_opioid |
| 94103998 | Codeine phosphate with dicycloverine with salts liquid | weak_opioid |
| 94105998 | Codeine phosphate with dicycloverine with salts liquid | weak_opioid |
| 94267998 | Codeine phosphate with menthol 15mg/5ml oral solution | weak_opioid |
| 94269998 | Codeine phosphate with menthol 15mg/5ml oral solution | weak_opioid |
| 88376998 | Codeine phosphate with other ingredient pastilles | weak_opioid |
| 97189992 | Codeine phosphate/paracetamol/sodium cit 8 mg cap | weak_opioid |
| 97189992 | Codeine phosphate/paracetamol/sodium cit 8 mg cap | weak_opioid |
| 95899992 | Codeine soluble tab | weak_opioid |
| 81062998 | Co-dydramol (dihydrocodeine and paracetamol) 10mg with 500mg/5ml oral suspension | weak_opioid |
| 81062998 | Co-dydramol (dihydrocodeine and paracetamol) 10mg with 500mg/5ml oral suspension | weak_opioid |
| 96376997 | Co-dydramol (dihydrocodeine and paracetamol) 7.46mg with 500mg tablets | weak_opioid |
| 96376997 | Co-dydramol (dihydrocodeine and paracetamol) 7.46mg with 500mg tablets | weak_opioid |
| 85781979 | Decongestant with Paracetamol tablets | paracetamol |
| 86628998 | Dexibuprofen 300mg tablets | weak_nsaid |
| 86635998 | Dexibuprofen 300mg tablets | weak_nsaid |
| 86624998 | Dexibuprofen 400mg tablets | weak_nsaid |
| 86629998 | Dexibuprofen 400mg tablets | weak_nsaid |
| 96636992 | Diamorphine 1 gm sup | strong_opioid |
| 97287992 | Diamorphine 1 mg inj | strong_opioid |
| 97286992 | Diamorphine 1.5 gm inj | strong_opioid |
| 46919978 | Diamorphine 1.6mg/dose nasal spray | strong_opioid |
| 97279992 | Diamorphine 10 mg sup | strong_opioid |
| 97278992 | Diamorphine 100 mg sup | strong_opioid |
| 63195979 | Diamorphine 100mg powder for solution for injection ampoules | strong_opioid |
| 94208997 | Diamorphine 100mg powder for solution for injection ampoules | strong_opioid |
| 95566979 | Diamorphine 100mg powder for solution for injection ampoules | strong_opioid |
| 97002998 | Diamorphine 100mg powder for solution for injection ampoules | strong_opioid |
| 99236990 | Diamorphine 100mg powder for solution for injection ampoules | strong_opioid |
| 63193979 | Diamorphine 10mg powder for solution for injection ampoules | strong_opioid |
| 92195990 | Diamorphine 10mg powder for solution for injection ampoules | strong_opioid |
| 94209996 | Diamorphine 10mg powder for solution for injection ampoules | strong_opioid |
| 95573979 | Diamorphine 10mg powder for solution for injection ampoules | strong_opioid |
| 95575979 | Diamorphine 10mg powder for solution for injection ampoules | strong_opioid |
| 97003997 | Diamorphine 10mg powder for solution for injection ampoules | strong_opioid |
| 97667989 | Diamorphine 10mg powder for solution for injection ampoules | strong_opioid |
| 98331989 | Diamorphine 10mg powder for solution for injection ampoules | strong_opioid |
| 99636989 | Diamorphine 10mg powder for solution for injection ampoules | strong_opioid |
| 99646989 | Diamorphine 10mg powder for solution for injection ampoules | strong_opioid |
| 94209998 | Diamorphine 10mg tablets | strong_opioid |
| 97681998 | Diamorphine 10mg tablets | strong_opioid |
| 98083998 | Diamorphine 10mg tablets | strong_opioid |
| 93427997 | Diamorphine 10mg/5ml oral solution | strong_opioid |
| 93431997 | Diamorphine 10mg/5ml oral solution | strong_opioid |
| 97273992 | Diamorphine 15 mg inj | strong_opioid |
| 84078998 | Diamorphine 15mg powder for solution for injection ampoules | strong_opioid |
| 93427996 | Diamorphine 15mg/5ml oral solution | strong_opioid |
| 93431996 | Diamorphine 15mg/5ml oral solution | strong_opioid |
| 96193992 | Diamorphine 2.5 gm inj | strong_opioid |
| 97272992 | Diamorphine 20 mg eli | strong_opioid |
| 97277992 | Diamorphine 20 mg sup | strong_opioid |
| 97276992 | Diamorphine 200 mg sup | strong_opioid |
| 96190992 | Diamorphine 25 mg sup | strong_opioid |
| 96192992 | Diamorphine 3 gm inj | strong_opioid |
| 96189992 | Diamorphine 30 mg sup | strong_opioid |
| 63191979 | Diamorphine 30mg powder for solution for injection ampoules | strong_opioid |
| 94208998 | Diamorphine 30mg powder for solution for injection ampoules | strong_opioid |
| 95571979 | Diamorphine 30mg powder for solution for injection ampoules | strong_opioid |
| 97003996 | Diamorphine 30mg powder for solution for injection ampoules | strong_opioid |
| 97667988 | Diamorphine 30mg powder for solution for injection ampoules | strong_opioid |
| 99636988 | Diamorphine 30mg powder for solution for injection ampoules | strong_opioid |
| 99646988 | Diamorphine 30mg powder for solution for injection ampoules | strong_opioid |
| 70120979 | Diamorphine 3mg/5ml oral solution | strong_opioid |
| 94246998 | Diamorphine 3mg/5ml oral solution | strong_opioid |
| 97285992 | Diamorphine 4 gm inj | strong_opioid |
| 97274992 | Diamorphine 40 mg sup | strong_opioid |
| 97288992 | Diamorphine 5 mg sup | strong_opioid |
| 97289992 | Diamorphine 50 mg sup | strong_opioid |
| 63189979 | Diamorphine 500mg powder for solution for injection ampoules | strong_opioid |
| 94208996 | Diamorphine 500mg powder for solution for injection ampoules | strong_opioid |
| 95563979 | Diamorphine 500mg powder for solution for injection ampoules | strong_opioid |
| 97002997 | Diamorphine 500mg powder for solution for injection ampoules | strong_opioid |
| 63187979 | Diamorphine 5mg powder for solution for injection ampoules | strong_opioid |
| 92196990 | Diamorphine 5mg powder for solution for injection ampoules | strong_opioid |
| 92327990 | Diamorphine 5mg powder for solution for injection ampoules | strong_opioid |
| 94209997 | Diamorphine 5mg powder for solution for injection ampoules | strong_opioid |
| 95577979 | Diamorphine 5mg powder for solution for injection ampoules | strong_opioid |
| 95578979 | Diamorphine 5mg powder for solution for injection ampoules | strong_opioid |
| 97003998 | Diamorphine 5mg powder for solution for injection ampoules | strong_opioid |
| 99646990 | Diamorphine 5mg powder for solution for injection ampoules | strong_opioid |
| 93427998 | Diamorphine 5mg/5ml oral solution | strong_opioid |
| 93431998 | Diamorphine 5mg/5ml oral solution | strong_opioid |
| 97275992 | Diamorphine 60 mg sup | strong_opioid |
| 96194992 | Diamorphine 64 mg inj | strong_opioid |
| 46916978 | Diamorphine 720micrograms/dose nasal spray | strong_opioid |
| 46917978 | Diamorphine 720micrograms/dose nasal spray | strong_opioid |
| 96188992 | Diamorphine 75 mg sup | strong_opioid |
| 92936998 | Diamorphine hydrochloride 100mg powder for injection solution | strong_opioid |
| 92937997 | Diamorphine hydrochloride 10mg powder for injection solution | strong_opioid |
| 96191992 | Diamorphine hydrochloride 20 mg inj | strong_opioid |
| 93487996 | Diamorphine hydrochloride 30mg powder for injection solution | strong_opioid |
| 93426998 | Diamorphine hydrochloride 3mg/5ml oral solution | strong_opioid |
| 92936997 | Diamorphine hydrochloride 500mg powder for injection solution | strong_opioid |
| 92937998 | Diamorphine hydrochloride 5mg powder for injection solution | strong_opioid |
| 97284992 | Diamorphine hydrochloride 60 mg inj | strong_opioid |
| 94247998 | Diamorphine hydrochloride bpc 1973 3mg/5ml oral solution | strong_opioid |
| 98852990 | Diamorphine hydrochloride powder | strong_opioid |
| 96402998 | Dichloralphenazone with paracetamol oral solution | paracetamol |
| 26468978 | Diclofenac 0.074% mouthwash sugar free | strong_nsaid |
| 26469978 | Diclofenac 0.074% mouthwash sugar free | strong_nsaid |
| 10119978 | Diclofenac 0.74mg/ml mouthwash sugar free | strong_nsaid |
| 7722978 | Diclofenac 1% gel | strong_nsaid |
| 9257978 | Diclofenac 1% gel | strong_nsaid |
| 9258978 | Diclofenac 1% gel | strong_nsaid |
| 86533998 | Diclofenac 1% patches | strong_nsaid |
| 86534998 | Diclofenac 1% transdermal patches | strong_nsaid |
| 86954998 | Diclofenac 1.16% gel | strong_nsaid |
| 88106998 | Diclofenac 1.16% gel | strong_nsaid |
| 91452979 | Diclofenac 1.16% gel | strong_nsaid |
| 91453979 | Diclofenac 1.16% gel | strong_nsaid |
| 91454979 | Diclofenac 1.16% gel | strong_nsaid |
| 91455979 | Diclofenac 1.16% gel | strong_nsaid |
| 91456979 | Diclofenac 1.16% gel | strong_nsaid |
| 91457979 | Diclofenac 1.16% gel | strong_nsaid |
| 91458979 | Diclofenac 1.16% gel | strong_nsaid |
| 91460979 | Diclofenac 1.16% gel | strong_nsaid |
| 91462979 | Diclofenac 1.16% gel | strong_nsaid |
| 93237998 | Diclofenac 1.16% gel | strong_nsaid |
| 93238998 | Diclofenac 1.16% gel | strong_nsaid |
| 89404998 | Diclofenac 100mg suppositories | strong_nsaid |
| 91414998 | Diclofenac 100mg suppositories | strong_nsaid |
| 95993990 | Diclofenac 100mg suppositories | strong_nsaid |
| 96399998 | Diclofenac 100mg suppositories | strong_nsaid |
| 98692996 | Diclofenac 100mg suppositories | strong_nsaid |
| 85130998 | Diclofenac 10mg dispersible tablets | strong_nsaid |
| 80260979 | Diclofenac 10mg/5ml oral solution | strong_nsaid |
| 80258979 | Diclofenac 10mg/5ml oral suspension | strong_nsaid |
| 89405998 | Diclofenac 12.5mg suppositories | strong_nsaid |
| 96399997 | Diclofenac 12.5mg suppositories | strong_nsaid |
| 98689998 | Diclofenac 12.5mg suppositories | strong_nsaid |
| 70514979 | Diclofenac 12.5mg/5ml oral solution | strong_nsaid |
| 70512979 | Diclofenac 12.5mg/5ml oral suspension | strong_nsaid |
| 45829978 | Diclofenac 140mg medicated plasters | strong_nsaid |
| 45830978 | Diclofenac 140mg medicated plasters | strong_nsaid |
| 92072998 | Diclofenac 16mg/ml topical solution | strong_nsaid |
| 92183998 | Diclofenac 16mg/ml topical solution | strong_nsaid |
| 97781998 | Diclofenac 16mg/ml topical solution | strong_nsaid |
| 46953978 | Diclofenac 2.32% gel | strong_nsaid |
| 83636978 | Diclofenac 2.32% gel | strong_nsaid |
| 83637978 | Diclofenac 2.32% gel | strong_nsaid |
| 89405997 | Diclofenac 25mg suppositories | strong_nsaid |
| 92604998 | Diclofenac 25mg suppositories | strong_nsaid |
| 98691996 | Diclofenac 25mg suppositories | strong_nsaid |
| 85332998 | Diclofenac 4% cutaneous spray | strong_nsaid |
| 91912979 | Diclofenac 50mg dispersible tablets sugar free | strong_nsaid |
| 93538998 | Diclofenac 50mg dispersible tablets sugar free | strong_nsaid |
| 89405996 | Diclofenac 50mg suppositories | strong_nsaid |
| 91950979 | Diclofenac 50mg suppositories | strong_nsaid |
| 92604997 | Diclofenac 50mg suppositories | strong_nsaid |
| 99644998 | Diclofenac 50mg suppositories | strong_nsaid |
| 70506979 | Diclofenac 50mg/5ml oral solution | strong_nsaid |
| 70557979 | Diclofenac 50mg/5ml oral suspension | strong_nsaid |
| 84152998 | Diclofenac 75mg/2ml solution for injection vials | strong_nsaid |
| 89398998 | Diclofenac 75mg/3ml solution for injection ampoules | strong_nsaid |
| 93669998 | Diclofenac 75mg/3ml solution for injection ampoules | strong_nsaid |
| 96550992 | Diclofenac 75mg/3ml solution for injection ampoules | strong_nsaid |
| 96815992 | Diclofenac 75mg/3ml solution for injection ampoules | strong_nsaid |
| 97295992 | Diclofenac 75mg/3ml solution for injection ampoules | strong_nsaid |
| 97809990 | Diclofenac 75mg/3ml solution for injection ampoules | strong_nsaid |
| 98691998 | Diclofenac 75mg/3ml solution for injection ampoules | strong_nsaid |
| 70504979 | Diclofenac 75mg/5ml oral suspension | strong_nsaid |
| 19180978 | Diclofenac 75mg/ml solution for injection ampoules | strong_nsaid |
| 81721998 | Diclofenac potassium 12.5mg tablets | strong_nsaid |
| 81987998 | Diclofenac potassium 12.5mg tablets | strong_nsaid |
| 83600998 | Diclofenac potassium 12.5mg tablets | strong_nsaid |
| 83604998 | Diclofenac potassium 12.5mg tablets | strong_nsaid |
| 89852998 | Diclofenac potassium 25mg tablets | strong_nsaid |
| 91420998 | Diclofenac potassium 25mg tablets | strong_nsaid |
| 91629979 | Diclofenac potassium 25mg tablets | strong_nsaid |
| 89852997 | Diclofenac potassium 50mg tablets | strong_nsaid |
| 91420997 | Diclofenac potassium 50mg tablets | strong_nsaid |
| 91619979 | Diclofenac potassium 50mg tablets | strong_nsaid |
| 91620979 | Diclofenac potassium 50mg tablets | strong_nsaid |
| 91624979 | Diclofenac potassium 50mg tablets | strong_nsaid |
| 91626979 | Diclofenac potassium 50mg tablets | strong_nsaid |
| 92229990 | Diclofenac potassium 50mg tablets | strong_nsaid |
| 92276990 | Diclofenac potassium 50mg tablets | strong_nsaid |
| 9259978 | Diclofenac sodium 1% gel | strong_nsaid |
| 85115998 | Diclofenac sodium 1% gel | strong_nsaid |
| 88456998 | Diclofenac sodium 100mg modified release tablets | strong_nsaid |
| 89171998 | Diclofenac sodium 100mg modified release tablets | strong_nsaid |
| 89336997 | Diclofenac sodium 100mg modified release tablets | strong_nsaid |
| 89511998 | Diclofenac sodium 100mg modified release tablets | strong_nsaid |
| 93005992 | Diclofenac sodium 100mg modified release tablets | strong_nsaid |
| 73230978 | Diclofenac sodium 100mg modified-release capsules | strong_nsaid |
| 81312998 | Diclofenac sodium 100mg modified-release capsules | strong_nsaid |
| 83441998 | Diclofenac sodium 100mg modified-release capsules | strong_nsaid |
| 87144998 | Diclofenac sodium 100mg modified-release capsules | strong_nsaid |
| 91897979 | Diclofenac sodium 100mg modified-release capsules | strong_nsaid |
| 91899979 | Diclofenac sodium 100mg modified-release capsules | strong_nsaid |
| 93072998 | Diclofenac sodium 100mg modified-release capsules | strong_nsaid |
| 93216996 | Diclofenac sodium 100mg modified-release capsules | strong_nsaid |
| 94539990 | Diclofenac sodium 100mg modified-release capsules | strong_nsaid |
| 94654990 | Diclofenac sodium 100mg modified-release capsules | strong_nsaid |
| 81021998 | Diclofenac sodium 100mg modified-release tablets | strong_nsaid |
| 82518998 | Diclofenac sodium 100mg modified-release tablets | strong_nsaid |
| 83470998 | Diclofenac sodium 100mg modified-release tablets | strong_nsaid |
| 85849998 | Diclofenac sodium 100mg modified-release tablets | strong_nsaid |
| 88414997 | Diclofenac sodium 100mg modified-release tablets | strong_nsaid |
| 87593998 | Diclofenac sodium 100mg modified-release tablets | strong_nsaid |
| 88021998 | Diclofenac sodium 100mg modified-release tablets | strong_nsaid |
| 88093998 | Diclofenac sodium 100mg modified-release tablets | strong_nsaid |
| 88294998 | Diclofenac sodium 100mg modified-release tablets | strong_nsaid |
| 88520998 | Diclofenac sodium 100mg modified-release tablets | strong_nsaid |
| 88840998 | Diclofenac sodium 100mg modified-release tablets | strong_nsaid |
| 88881998 | Diclofenac sodium 100mg modified-release tablets | strong_nsaid |
| 89162998 | Diclofenac sodium 100mg modified-release tablets | strong_nsaid |
| 89167998 | Diclofenac sodium 100mg modified-release tablets | strong_nsaid |
| 89368997 | Diclofenac sodium 100mg modified-release tablets | strong_nsaid |
| 90080997 | Diclofenac sodium 100mg modified-release tablets | strong_nsaid |
| 90793997 | Diclofenac sodium 100mg modified-release tablets | strong_nsaid |
| 91049997 | Diclofenac sodium 100mg modified-release tablets | strong_nsaid |
| 91519998 | Diclofenac sodium 100mg modified-release tablets | strong_nsaid |
| 91616998 | Diclofenac sodium 100mg modified-release tablets | strong_nsaid |
| 92650997 | Diclofenac sodium 100mg modified-release tablets | strong_nsaid |
| 92952996 | Diclofenac sodium 100mg modified-release tablets | strong_nsaid |
| 96400996 | Diclofenac sodium 100mg modified-release tablets | strong_nsaid |
| 96641990 | Diclofenac sodium 100mg modified-release tablets | strong_nsaid |
| 96881990 | Diclofenac sodium 100mg modified-release tablets | strong_nsaid |
| 97765988 | Diclofenac sodium 100mg modified-release tablets | strong_nsaid |
| 98690998 | Diclofenac sodium 100mg modified-release tablets | strong_nsaid |
| 99410996 | Diclofenac sodium 100mg modified-release tablets | strong_nsaid |
| 83078998 | Diclofenac sodium 25mg gastro-resistant tablets | strong_nsaid |
| 85154998 | Diclofenac sodium 25mg gastro-resistant tablets | strong_nsaid |
| 88296998 | Diclofenac sodium 25mg gastro-resistant tablets | strong_nsaid |
| 89344998 | Diclofenac sodium 25mg gastro-resistant tablets | strong_nsaid |
| 89419998 | Diclofenac sodium 25mg gastro-resistant tablets | strong_nsaid |
| 90512998 | Diclofenac sodium 25mg gastro-resistant tablets | strong_nsaid |
| 91213998 | Diclofenac sodium 25mg gastro-resistant tablets | strong_nsaid |
| 92184998 | Diclofenac sodium 25mg gastro-resistant tablets | strong_nsaid |
| 92952998 | Diclofenac sodium 25mg gastro-resistant tablets | strong_nsaid |
| 93110998 | Diclofenac sodium 25mg gastro-resistant tablets | strong_nsaid |
| 93215998 | Diclofenac sodium 25mg gastro-resistant tablets | strong_nsaid |
| 93216998 | Diclofenac sodium 25mg gastro-resistant tablets | strong_nsaid |
| 94659998 | Diclofenac sodium 25mg gastro-resistant tablets | strong_nsaid |
| 96531990 | Diclofenac sodium 25mg gastro-resistant tablets | strong_nsaid |
| 96890990 | Diclofenac sodium 25mg gastro-resistant tablets | strong_nsaid |
| 96966990 | Diclofenac sodium 25mg gastro-resistant tablets | strong_nsaid |
| 97022990 | Diclofenac sodium 25mg gastro-resistant tablets | strong_nsaid |
| 97156989 | Diclofenac sodium 25mg gastro-resistant tablets | strong_nsaid |
| 97682998 | Diclofenac sodium 25mg gastro-resistant tablets | strong_nsaid |
| 97704998 | Diclofenac sodium 25mg gastro-resistant tablets | strong_nsaid |
| 97765990 | Diclofenac sodium 25mg gastro-resistant tablets | strong_nsaid |
| 98569990 | Diclofenac sodium 25mg gastro-resistant tablets | strong_nsaid |
| 98692998 | Diclofenac sodium 25mg gastro-resistant tablets | strong_nsaid |
| 99213990 | Diclofenac sodium 25mg gastro-resistant tablets | strong_nsaid |
| 99232990 | Diclofenac sodium 25mg gastro-resistant tablets | strong_nsaid |
| 99234990 | Diclofenac sodium 25mg gastro-resistant tablets | strong_nsaid |
| 99410998 | Diclofenac sodium 25mg gastro-resistant tablets | strong_nsaid |
| 99644990 | Diclofenac sodium 25mg gastro-resistant tablets | strong_nsaid |
| 96400998 | Diclofenac sodium 25mg tablets | strong_nsaid |
| 91709998 | Diclofenac sodium 3% gel | strong_nsaid |
| 97781997 | Diclofenac sodium 3% gel | strong_nsaid |
| 60596979 | Diclofenac sodium 4% spray | strong_nsaid |
| 80916998 | Diclofenac sodium 4% spray | strong_nsaid |
| 82868998 | Diclofenac sodium 4% spray | strong_nsaid |
| 83109998 | Diclofenac sodium 4% spray | strong_nsaid |
| 91908979 | Diclofenac sodium 50mg dispersible tablets | strong_nsaid |
| 91909979 | Diclofenac sodium 50mg dispersible tablets | strong_nsaid |
| 91911979 | Diclofenac sodium 50mg dispersible tablets | strong_nsaid |
| 91913979 | Diclofenac sodium 50mg dispersible tablets | strong_nsaid |
| 93540998 | Diclofenac sodium 50mg dispersible tablets | strong_nsaid |
| 83556978 | Diclofenac sodium 50mg gastro-resistant / misoprostol 200microgram tablets | strong_nsaid |
| 91901979 | Diclofenac sodium 50mg gastro-resistant / Misoprostol 200microgram tablets | strong_nsaid |
| 93085998 | Diclofenac sodium 50mg gastro-resistant / Misoprostol 200microgram tablets | strong_nsaid |
| 93086998 | Diclofenac sodium 50mg gastro-resistant / Misoprostol 200microgram tablets | strong_nsaid |
| 83077998 | Diclofenac sodium 50mg gastro-resistant tablets | strong_nsaid |
| 88094997 | Diclofenac sodium 50mg gastro-resistant tablets | strong_nsaid |
| 88296997 | Diclofenac sodium 50mg gastro-resistant tablets | strong_nsaid |
| 89344997 | Diclofenac sodium 50mg gastro-resistant tablets | strong_nsaid |
| 89419997 | Diclofenac sodium 50mg gastro-resistant tablets | strong_nsaid |
| 90151998 | Diclofenac sodium 50mg gastro-resistant tablets | strong_nsaid |
| 91213997 | Diclofenac sodium 50mg gastro-resistant tablets | strong_nsaid |
| 91747998 | Diclofenac sodium 50mg gastro-resistant tablets | strong_nsaid |
| 91920979 | Diclofenac sodium 50mg gastro-resistant tablets | strong_nsaid |
| 92185998 | Diclofenac sodium 50mg gastro-resistant tablets | strong_nsaid |
| 92952997 | Diclofenac sodium 50mg gastro-resistant tablets | strong_nsaid |
| 93110997 | Diclofenac sodium 50mg gastro-resistant tablets | strong_nsaid |
| 93215997 | Diclofenac sodium 50mg gastro-resistant tablets | strong_nsaid |
| 93216997 | Diclofenac sodium 50mg gastro-resistant tablets | strong_nsaid |
| 93352990 | Diclofenac sodium 50mg gastro-resistant tablets | strong_nsaid |
| 94659997 | Diclofenac sodium 50mg gastro-resistant tablets | strong_nsaid |
| 96135990 | Diclofenac sodium 50mg gastro-resistant tablets | strong_nsaid |
| 96531989 | Diclofenac sodium 50mg gastro-resistant tablets | strong_nsaid |
| 96890989 | Diclofenac sodium 50mg gastro-resistant tablets | strong_nsaid |
| 96966989 | Diclofenac sodium 50mg gastro-resistant tablets | strong_nsaid |
| 97022989 | Diclofenac sodium 50mg gastro-resistant tablets | strong_nsaid |
| 97049989 | Diclofenac sodium 50mg gastro-resistant tablets | strong_nsaid |
| 97156990 | Diclofenac sodium 50mg gastro-resistant tablets | strong_nsaid |
| 97704997 | Diclofenac sodium 50mg gastro-resistant tablets | strong_nsaid |
| 97765989 | Diclofenac sodium 50mg gastro-resistant tablets | strong_nsaid |
| 98346990 | Diclofenac sodium 50mg gastro-resistant tablets | strong_nsaid |
| 98569989 | Diclofenac sodium 50mg gastro-resistant tablets | strong_nsaid |
| 98692997 | Diclofenac sodium 50mg gastro-resistant tablets | strong_nsaid |
| 99213989 | Diclofenac sodium 50mg gastro-resistant tablets | strong_nsaid |
| 99232989 | Diclofenac sodium 50mg gastro-resistant tablets | strong_nsaid |
| 99234989 | Diclofenac sodium 50mg gastro-resistant tablets | strong_nsaid |
| 99410997 | Diclofenac sodium 50mg gastro-resistant tablets | strong_nsaid |
| 99644989 | Diclofenac sodium 50mg gastro-resistant tablets | strong_nsaid |
| 96400997 | Diclofenac sodium 50mg tablets | strong_nsaid |
| 83555978 | Diclofenac sodium 75mg gastro-resistant / misoprostol 200microgram tablets | strong_nsaid |
| 90358998 | Diclofenac sodium 75mg gastro-resistant / Misoprostol 200microgram tablets | strong_nsaid |
| 91892979 | Diclofenac sodium 75mg gastro-resistant / Misoprostol 200microgram tablets | strong_nsaid |
| 91896979 | Diclofenac sodium 75mg gastro-resistant / Misoprostol 200microgram tablets | strong_nsaid |
| 93085997 | Diclofenac sodium 75mg gastro-resistant / Misoprostol 200microgram tablets | strong_nsaid |
| 60486979 | Diclofenac sodium 75mg gastro-resistant modified-release capsules | strong_nsaid |
| 92668998 | Diclofenac sodium 75mg gastro-resistant modified-release capsules | strong_nsaid |
| 73232978 | Diclofenac sodium 75mg modified-release capsules | strong_nsaid |
| 78165978 | Diclofenac sodium 75mg modified-release capsules | strong_nsaid |
| 81313998 | Diclofenac sodium 75mg modified-release capsules | strong_nsaid |
| 83612998 | Diclofenac sodium 75mg modified-release capsules | strong_nsaid |
| 84291978 | Diclofenac sodium 75mg modified-release capsules | strong_nsaid |
| 87145998 | Diclofenac sodium 75mg modified-release capsules | strong_nsaid |
| 91953979 | Diclofenac sodium 75mg modified-release capsules | strong_nsaid |
| 92652998 | Diclofenac sodium 75mg modified-release capsules | strong_nsaid |
| 93072997 | Diclofenac sodium 75mg modified-release capsules | strong_nsaid |
| 94540990 | Diclofenac sodium 75mg modified-release capsules | strong_nsaid |
| 94655990 | Diclofenac sodium 75mg modified-release capsules | strong_nsaid |
| 98970998 | Diclofenac sodium 75mg modified-release capsules | strong_nsaid |
| 81619998 | Diclofenac sodium 75mg modified-release tablets | strong_nsaid |
| 83609998 | Diclofenac sodium 75mg modified-release tablets | strong_nsaid |
| 85848998 | Diclofenac sodium 75mg modified-release tablets | strong_nsaid |
| 88414998 | Diclofenac sodium 75mg modified-release tablets | strong_nsaid |
| 87595998 | Diclofenac sodium 75mg modified-release tablets | strong_nsaid |
| 88022998 | Diclofenac sodium 75mg modified-release tablets | strong_nsaid |
| 88092998 | Diclofenac sodium 75mg modified-release tablets | strong_nsaid |
| 88295998 | Diclofenac sodium 75mg modified-release tablets | strong_nsaid |
| 88309998 | Diclofenac sodium 75mg modified-release tablets | strong_nsaid |
| 88520997 | Diclofenac sodium 75mg modified-release tablets | strong_nsaid |
| 88882998 | Diclofenac sodium 75mg modified-release tablets | strong_nsaid |
| 89052998 | Diclofenac sodium 75mg modified-release tablets | strong_nsaid |
| 89176998 | Diclofenac sodium 75mg modified-release tablets | strong_nsaid |
| 89336998 | Diclofenac sodium 75mg modified-release tablets | strong_nsaid |
| 89368998 | Diclofenac sodium 75mg modified-release tablets | strong_nsaid |
| 89390998 | Diclofenac sodium 75mg modified-release tablets | strong_nsaid |
| 89395998 | Diclofenac sodium 75mg modified-release tablets | strong_nsaid |
| 89516998 | Diclofenac sodium 75mg modified-release tablets | strong_nsaid |
| 90080998 | Diclofenac sodium 75mg modified-release tablets | strong_nsaid |
| 90628998 | Diclofenac sodium 75mg modified-release tablets | strong_nsaid |
| 90793998 | Diclofenac sodium 75mg modified-release tablets | strong_nsaid |
| 91049998 | Diclofenac sodium 75mg modified-release tablets | strong_nsaid |
| 91583998 | Diclofenac sodium 75mg modified-release tablets | strong_nsaid |
| 91889979 | Diclofenac sodium 75mg modified-release tablets | strong_nsaid |
| 92384998 | Diclofenac sodium 75mg modified-release tablets | strong_nsaid |
| 92650998 | Diclofenac sodium 75mg modified-release tablets | strong_nsaid |
| 93006992 | Diclofenac sodium 75mg modified-release tablets | strong_nsaid |
| 96399996 | Diclofenac sodium 75mg modified-release tablets | strong_nsaid |
| 96531988 | Diclofenac sodium 75mg modified-release tablets | strong_nsaid |
| 97138990 | Diclofenac sodium 75mg modified-release tablets | strong_nsaid |
| 97147990 | Diclofenac sodium 75mg modified-release tablets | strong_nsaid |
| 98060998 | Diclofenac sodium 75mg modified-release tablets | strong_nsaid |
| 98569988 | Diclofenac sodium 75mg modified-release tablets | strong_nsaid |
| 98691997 | Diclofenac sodium 75mg modified-release tablets | strong_nsaid |
| 99232988 | Diclofenac sodium 75mg modified-release tablets | strong_nsaid |
| 84151998 | Diclofenac sodium 75mg/2ml injection | strong_nsaid |
| 86911998 | Dihydrocodeine 10mg with paracetamol 500mg/5ml oral suspension sugar free | weak_opioid |
| 86911998 | Dihydrocodeine 10mg with paracetamol 500mg/5ml oral suspension sugar free | weak_opioid |
| 96379997 | Dihydrocodeine 10mg/5ml oral solution | weak_opioid |
| 96730989 | Dihydrocodeine 10mg/5ml oral solution | weak_opioid |
| 80216979 | Dihydrocodeine 10mg/5ml oral suspension | weak_opioid |
| 96375996 | Dihydrocodeine 120mg modified-release tablets | weak_opioid |
| 96377996 | Dihydrocodeine 120mg modified-release tablets | weak_opioid |
| 70555979 | Dihydrocodeine 15mg/5ml oral solution | weak_opioid |
| 92776990 | Dihydrocodeine 30mg tablets | weak_opioid |
| 96379998 | Dihydrocodeine 30mg tablets | weak_opioid |
| 96947990 | Dihydrocodeine 30mg tablets | weak_opioid |
| 97001990 | Dihydrocodeine 30mg tablets | weak_opioid |
| 98059990 | Dihydrocodeine 30mg tablets | weak_opioid |
| 98174990 | Dihydrocodeine 30mg tablets | weak_opioid |
| 98349990 | Dihydrocodeine 30mg tablets | weak_opioid |
| 98568990 | Dihydrocodeine 30mg tablets | weak_opioid |
| 99624990 | Dihydrocodeine 30mg tablets | weak_opioid |
| 99626990 | Dihydrocodeine 30mg tablets | weak_opioid |
| 99627990 | Dihydrocodeine 30mg tablets | weak_opioid |
| 99628990 | Dihydrocodeine 30mg tablets | weak_opioid |
| 70553979 | Dihydrocodeine 30mg/5ml oral solution | weak_opioid |
| 83478998 | Dihydrocodeine 40mg tablets | weak_opioid |
| 96379996 | Dihydrocodeine 40mg tablets | weak_opioid |
| 98875996 | Dihydrocodeine 40mg tablets | weak_opioid |
| 93671998 | Dihydrocodeine 50mg/1ml solution for injection ampoules | weak_opioid |
| 96375998 | Dihydrocodeine 60mg modified-release tablets | weak_opioid |
| 96377998 | Dihydrocodeine 60mg modified-release tablets | weak_opioid |
| 96375997 | Dihydrocodeine 90mg modified-release tablets | weak_opioid |
| 96377997 | Dihydrocodeine 90mg modified-release tablets | weak_opioid |
| 98875997 | Dihydrocodeine tartrate 10mg/5ml elixir | weak_opioid |
| 97265992 | Dihydrocodeine tartrate 30mg tablets | weak_opioid |
| 98875998 | Dihydrocodeine tartrate 30mg tablets | weak_opioid |
| 98874998 | Dihydrocodeine tartrate 50mg/1ml injection | weak_opioid |
| 97305992 | Dihydrocodeine tartrate/aspirin 300 mg tab | weak_opioid |
| 98018996 | Dihydrocodeine with paracetamol 20mg with 500mg effervescent tablets | weak_opioid |
| 98018996 | Dihydrocodeine with paracetamol 20mg with 500mg effervescent tablets | weak_opioid |
| 86503998 | Dihydrocodeine with paracetamol 7.46mg with 500mg effervescent tablets | weak_opioid |
| 86503998 | Dihydrocodeine with paracetamol 7.46mg with 500mg effervescent tablets | weak_opioid |
| 94421998 | Dihydrocodeine with paracetamol 7.46mg+500mg tablets | weak_opioid |
| 94421998 | Dihydrocodeine with paracetamol 7.46mg+500mg tablets | weak_opioid |
| 90249998 | Dihydrocodeine with paracetamol forte 30mg with 500mg effervescent tablets | weak_opioid |
| 90249998 | Dihydrocodeine with paracetamol forte 30mg with 500mg effervescent tablets | weak_opioid |
| 87335998 | Duloxetine 20mg gastro-resistant capsules | antidepressant |
| 87337998 | Duloxetine 20mg gastro-resistant capsules | antidepressant |
| 20477978 | Duloxetine 30mg gastro-resistant capsules | antidepressant |
| 39667978 | Duloxetine 30mg gastro-resistant capsules | antidepressant |
| 46227978 | Duloxetine 30mg gastro-resistant capsules | antidepressant |
| 86997998 | Duloxetine 30mg gastro-resistant capsules | antidepressant |
| 86999998 | Duloxetine 30mg gastro-resistant capsules | antidepressant |
| 89023979 | Duloxetine 30mg gastro-resistant capsules | antidepressant |
| 17416978 | Duloxetine 30mg/5ml oral suspension | antidepressant |
| 87334998 | Duloxetine 40mg gastro-resistant capsules | antidepressant |
| 87336998 | Duloxetine 40mg gastro-resistant capsules | antidepressant |
| 23031978 | Duloxetine 60mg gastro-resistant capsules | antidepressant |
| 20479978 | Duloxetine 60mg gastro-resistant capsules | antidepressant |
| 37600978 | Duloxetine 60mg gastro-resistant capsules | antidepressant |
| 39671978 | Duloxetine 60mg gastro-resistant capsules | antidepressant |
| 39672978 | Duloxetine 60mg gastro-resistant capsules | antidepressant |
| 51109978 | Duloxetine 60mg gastro-resistant capsules | antidepressant |
| 86996998 | Duloxetine 60mg gastro-resistant capsules | antidepressant |
| 86998998 | Duloxetine 60mg gastro-resistant capsules | antidepressant |
| 17414978 | Duloxetine 60mg/5ml oral suspension | antidepressant |
| 90306997 | Fentanyl 1.2mg lozenges | strong_opioid |
| 92207996 | Fentanyl 1.2mg lozenges | strong_opioid |
| 90306996 | Fentanyl 1.6mg lozenges | strong_opioid |
| 91006998 | Fentanyl 1.6mg lozenges | strong_opioid |
| 83193998 | Fentanyl 100microgram buccal tablets sugar free | strong_opioid |
| 83198998 | Fentanyl 100microgram buccal tablets sugar free | strong_opioid |
| 83228998 | Fentanyl 100microgram sublingual tablets sugar free | strong_opioid |
| 83234998 | Fentanyl 100microgram sublingual tablets sugar free | strong_opioid |
| 85345998 | Fentanyl 100micrograms/2ml solution for injection ampoules | strong_opioid |
| 81879998 | Fentanyl 100micrograms/dose nasal spray | strong_opioid |
| 82614998 | Fentanyl 100micrograms/dose nasal spray | strong_opioid |
| 82617998 | Fentanyl 100micrograms/dose nasal spray | strong_opioid |
| 46309978 | Fentanyl 100micrograms/hour transdermal patches | strong_opioid |
| 74464978 | Fentanyl 100micrograms/hour transdermal patches | strong_opioid |
| 81222998 | Fentanyl 100micrograms/hour transdermal patches | strong_opioid |
| 84141998 | Fentanyl 100micrograms/hour transdermal patches | strong_opioid |
| 83001998 | Fentanyl 100micrograms/hour transdermal patches | strong_opioid |
| 83015998 | Fentanyl 100micrograms/hour transdermal patches | strong_opioid |
| 84068998 | Fentanyl 100micrograms/hour transdermal patches | strong_opioid |
| 84136998 | Fentanyl 100micrograms/hour transdermal patches | strong_opioid |
| 85104998 | Fentanyl 100micrograms/hour transdermal patches | strong_opioid |
| 86590998 | Fentanyl 100micrograms/hour transdermal patches | strong_opioid |
| 86985998 | Fentanyl 100micrograms/hour transdermal patches | strong_opioid |
| 92591998 | Fentanyl 100micrograms/hour transdermal patches | strong_opioid |
| 92592998 | Fentanyl 100micrograms/hour transdermal patches | strong_opioid |
| 93845990 | Fentanyl 100micrograms/hour transdermal patches | strong_opioid |
| 33834978 | Fentanyl 12micrograms/hour transdermal patches | strong_opioid |
| 46162978 | Fentanyl 12micrograms/hour transdermal patches | strong_opioid |
| 74379978 | Fentanyl 12micrograms/hour transdermal patches | strong_opioid |
| 78243979 | Fentanyl 12micrograms/hour transdermal patches | strong_opioid |
| 81226998 | Fentanyl 12micrograms/hour transdermal patches | strong_opioid |
| 82606998 | Fentanyl 12micrograms/hour transdermal patches | strong_opioid |
| 84140998 | Fentanyl 12micrograms/hour transdermal patches | strong_opioid |
| 85109998 | Fentanyl 12micrograms/hour transdermal patches | strong_opioid |
| 86455998 | Fentanyl 12micrograms/hour transdermal patches | strong_opioid |
| 86456998 | Fentanyl 12micrograms/hour transdermal patches | strong_opioid |
| 75849978 | Fentanyl 133microgram sublingual tablets sugar free | strong_opioid |
| 83629978 | Fentanyl 2.5mg/50ml solution for infusion vials | strong_opioid |
| 79312978 | Fentanyl 200microgram buccal films sugar free | strong_opioid |
| 83192998 | Fentanyl 200microgram buccal tablets sugar free | strong_opioid |
| 83197998 | Fentanyl 200microgram buccal tablets sugar free | strong_opioid |
| 90307998 | Fentanyl 200microgram lozenges | strong_opioid |
| 92592997 | Fentanyl 200microgram lozenges | strong_opioid |
| 83227998 | Fentanyl 200microgram sublingual tablets sugar free | strong_opioid |
| 83233998 | Fentanyl 200microgram sublingual tablets sugar free | strong_opioid |
| 82613998 | Fentanyl 200micrograms/dose nasal spray | strong_opioid |
| 82616998 | Fentanyl 200micrograms/dose nasal spray | strong_opioid |
| 46315978 | Fentanyl 25micrograms/hour transdermal patches | strong_opioid |
| 74467978 | Fentanyl 25micrograms/hour transdermal patches | strong_opioid |
| 81225998 | Fentanyl 25micrograms/hour transdermal patches | strong_opioid |
| 84139998 | Fentanyl 25micrograms/hour transdermal patches | strong_opioid |
| 83004998 | Fentanyl 25micrograms/hour transdermal patches | strong_opioid |
| 83018998 | Fentanyl 25micrograms/hour transdermal patches | strong_opioid |
| 84071998 | Fentanyl 25micrograms/hour transdermal patches | strong_opioid |
| 84144998 | Fentanyl 25micrograms/hour transdermal patches | strong_opioid |
| 85108998 | Fentanyl 25micrograms/hour transdermal patches | strong_opioid |
| 86593998 | Fentanyl 25micrograms/hour transdermal patches | strong_opioid |
| 86988998 | Fentanyl 25micrograms/hour transdermal patches | strong_opioid |
| 90671979 | Fentanyl 25micrograms/hour transdermal patches | strong_opioid |
| 90674979 | Fentanyl 25micrograms/hour transdermal patches | strong_opioid |
| 90675979 | Fentanyl 25micrograms/hour transdermal patches | strong_opioid |
| 92593998 | Fentanyl 25micrograms/hour transdermal patches | strong_opioid |
| 92594998 | Fentanyl 25micrograms/hour transdermal patches | strong_opioid |
| 93847990 | Fentanyl 25micrograms/hour transdermal patches | strong_opioid |
| 75847978 | Fentanyl 267microgram sublingual tablets sugar free | strong_opioid |
| 83226998 | Fentanyl 300microgram sublingual tablets sugar free | strong_opioid |
| 83232998 | Fentanyl 300microgram sublingual tablets sugar free | strong_opioid |
| 78349978 | Fentanyl 37.5microgram/hour transdermal patches | strong_opioid |
| 78350978 | Fentanyl 37.5microgram/hour transdermal patches | strong_opioid |
| 79310978 | Fentanyl 400microgram buccal films sugar free | strong_opioid |
| 83191998 | Fentanyl 400microgram buccal tablets sugar free | strong_opioid |
| 83196998 | Fentanyl 400microgram buccal tablets sugar free | strong_opioid |
| 90307997 | Fentanyl 400microgram lozenges | strong_opioid |
| 92592996 | Fentanyl 400microgram lozenges | strong_opioid |
| 83225998 | Fentanyl 400microgram sublingual tablets sugar free | strong_opioid |
| 83231998 | Fentanyl 400microgram sublingual tablets sugar free | strong_opioid |
| 81878998 | Fentanyl 400micrograms/dose nasal spray | strong_opioid |
| 81880998 | Fentanyl 400micrograms/dose nasal spray | strong_opioid |
| 84156998 | Fentanyl 40micrograms/dose transdermal system | strong_opioid |
| 85342998 | Fentanyl 500micrograms/10ml solution for injection ampoules | strong_opioid |
| 85344998 | Fentanyl 500micrograms/10ml solution for injection ampoules | strong_opioid |
| 96550998 | Fentanyl 500micrograms/10ml solution for injection ampoules | strong_opioid |
| 96552998 | Fentanyl 500micrograms/10ml solution for injection ampoules | strong_opioid |
| 97416992 | Fentanyl 500micrograms/10ml solution for injection ampoules | strong_opioid |
| 82615998 | Fentanyl 50micrograms/dose nasal spray | strong_opioid |
| 82618998 | Fentanyl 50micrograms/dose nasal spray | strong_opioid |
| 46313978 | Fentanyl 50micrograms/hour transdermal patches | strong_opioid |
| 74466978 | Fentanyl 50micrograms/hour transdermal patches | strong_opioid |
| 79732978 | Fentanyl 50micrograms/hour transdermal patches | strong_opioid |
| 81224998 | Fentanyl 50micrograms/hour transdermal patches | strong_opioid |
| 84138998 | Fentanyl 50micrograms/hour transdermal patches | strong_opioid |
| 83003998 | Fentanyl 50micrograms/hour transdermal patches | strong_opioid |
| 83017998 | Fentanyl 50micrograms/hour transdermal patches | strong_opioid |
| 84070998 | Fentanyl 50micrograms/hour transdermal patches | strong_opioid |
| 84143998 | Fentanyl 50micrograms/hour transdermal patches | strong_opioid |
| 85107998 | Fentanyl 50micrograms/hour transdermal patches | strong_opioid |
| 86592998 | Fentanyl 50micrograms/hour transdermal patches | strong_opioid |
| 86987998 | Fentanyl 50micrograms/hour transdermal patches | strong_opioid |
| 90669979 | Fentanyl 50micrograms/hour transdermal patches | strong_opioid |
| 92593997 | Fentanyl 50micrograms/hour transdermal patches | strong_opioid |
| 92594997 | Fentanyl 50micrograms/hour transdermal patches | strong_opioid |
| 93835990 | Fentanyl 50micrograms/hour transdermal patches | strong_opioid |
| 75845978 | Fentanyl 533microgram sublingual tablets sugar free | strong_opioid |
| 83190998 | Fentanyl 600microgram buccal tablets sugar free | strong_opioid |
| 83195998 | Fentanyl 600microgram buccal tablets sugar free | strong_opioid |
| 90307996 | Fentanyl 600microgram lozenges | strong_opioid |
| 92207998 | Fentanyl 600microgram lozenges | strong_opioid |
| 83224998 | Fentanyl 600microgram sublingual tablets sugar free | strong_opioid |
| 83230998 | Fentanyl 600microgram sublingual tablets sugar free | strong_opioid |
| 46311978 | Fentanyl 75micrograms/hour transdermal patches | strong_opioid |
| 74465978 | Fentanyl 75micrograms/hour transdermal patches | strong_opioid |
| 79729978 | Fentanyl 75micrograms/hour transdermal patches | strong_opioid |
| 81223998 | Fentanyl 75micrograms/hour transdermal patches | strong_opioid |
| 84137998 | Fentanyl 75micrograms/hour transdermal patches | strong_opioid |
| 84142998 | Fentanyl 75micrograms/hour transdermal patches | strong_opioid |
| 83002998 | Fentanyl 75micrograms/hour transdermal patches | strong_opioid |
| 83016998 | Fentanyl 75micrograms/hour transdermal patches | strong_opioid |
| 84069998 | Fentanyl 75micrograms/hour transdermal patches | strong_opioid |
| 85106998 | Fentanyl 75micrograms/hour transdermal patches | strong_opioid |
| 86591998 | Fentanyl 75micrograms/hour transdermal patches | strong_opioid |
| 86986998 | Fentanyl 75micrograms/hour transdermal patches | strong_opioid |
| 90661979 | Fentanyl 75micrograms/hour transdermal patches | strong_opioid |
| 92593996 | Fentanyl 75micrograms/hour transdermal patches | strong_opioid |
| 92594996 | Fentanyl 75micrograms/hour transdermal patches | strong_opioid |
| 93539990 | Fentanyl 75micrograms/hour transdermal patches | strong_opioid |
| 93846990 | Fentanyl 75micrograms/hour transdermal patches | strong_opioid |
| 79308978 | Fentanyl 800microgram buccal films sugar free | strong_opioid |
| 83189998 | Fentanyl 800microgram buccal tablets sugar free | strong_opioid |
| 83194998 | Fentanyl 800microgram buccal tablets sugar free | strong_opioid |
| 90306998 | Fentanyl 800microgram lozenges | strong_opioid |
| 92207997 | Fentanyl 800microgram lozenges | strong_opioid |
| 83223998 | Fentanyl 800microgram sublingual tablets sugar free | strong_opioid |
| 83229998 | Fentanyl 800microgram sublingual tablets sugar free | strong_opioid |
| 79311978 | Fentanyl buccal film 200mcg sachets | strong_opioid |
| 84154998 | Fentanyl iontophoretic transdermal 40mcg/dose system | strong_opioid |
| 96551998 | Fentanyl with droperidol 500microgramwith2.5mg/ml injection | strong_opioid |
| 20009978 | Gabapentin 10% gel | gabapentinoid |
| 33976978 | Gabapentin 100mg capsules | gabapentinoid |
| 92872990 | Gabapentin 100mg capsules | gabapentinoid |
| 93051990 | Gabapentin 100mg capsules | gabapentinoid |
| 93812990 | Gabapentin 100mg capsules | gabapentinoid |
| 94123990 | Gabapentin 100mg capsules | gabapentinoid |
| 94167990 | Gabapentin 100mg capsules | gabapentinoid |
| 94834998 | Gabapentin 100mg capsules | gabapentinoid |
| 94835998 | Gabapentin 100mg capsules | gabapentinoid |
| 95045979 | Gabapentin 100mg capsules | gabapentinoid |
| 95049979 | Gabapentin 100mg capsules | gabapentinoid |
| 95161990 | Gabapentin 100mg capsules | gabapentinoid |
| 95190990 | Gabapentin 100mg capsules | gabapentinoid |
| 80032979 | Gabapentin 100mg/5ml oral solution | gabapentinoid |
| 80030979 | Gabapentin 100mg/5ml oral suspension | gabapentinoid |
| 69153979 | Gabapentin 150mg/5ml oral solution | gabapentinoid |
| 69149979 | Gabapentin 200mg/5ml oral solution | gabapentinoid |
| 53063979 | Gabapentin 250mg/5ml oral solution | gabapentinoid |
| 53266979 | Gabapentin 250mg/5ml oral solution | gabapentinoid |
| 53267979 | Gabapentin 250mg/5ml oral solution | gabapentinoid |
| 86485998 | Gabapentin 250mg/5ml oral solution | gabapentinoid |
| 89157979 | Gabapentin 250mg/5ml oral solution | gabapentinoid |
| 93743990 | Gabapentin 250mg/5ml oral solution | gabapentinoid |
| 81991998 | Gabapentin 250mg/5ml oral suspension | gabapentinoid |
| 33975978 | Gabapentin 300mg capsules | gabapentinoid |
| 92871990 | Gabapentin 300mg capsules | gabapentinoid |
| 93811990 | Gabapentin 300mg capsules | gabapentinoid |
| 94834997 | Gabapentin 300mg capsules | gabapentinoid |
| 94835997 | Gabapentin 300mg capsules | gabapentinoid |
| 95032979 | Gabapentin 300mg capsules | gabapentinoid |
| 95159990 | Gabapentin 300mg capsules | gabapentinoid |
| 95189990 | Gabapentin 300mg capsules | gabapentinoid |
| 80027979 | Gabapentin 300mg/5ml oral solution | gabapentinoid |
| 80023979 | Gabapentin 300mg/5ml oral suspension | gabapentinoid |
| 90424998 | Gabapentin 300mg+600mg pack | gabapentinoid |
| 94834996 | Gabapentin 400mg capsules | gabapentinoid |
| 94835996 | Gabapentin 400mg capsules | gabapentinoid |
| 95158990 | Gabapentin 400mg capsules | gabapentinoid |
| 95188990 | Gabapentin 400mg capsules | gabapentinoid |
| 64705979 | Gabapentin 400mg/5ml oral solution | gabapentinoid |
| 86362998 | Gabapentin 400mg/5ml oral suspension | gabapentinoid |
| 80021979 | Gabapentin 450mg/5ml oral solution | gabapentinoid |
| 69145979 | Gabapentin 500mg/5ml oral solution | gabapentinoid |
| 69143979 | Gabapentin 500mg/5ml oral suspension | gabapentinoid |
| 80017979 | Gabapentin 50mg/5ml oral solution | gabapentinoid |
| 80015979 | Gabapentin 50mg/5ml oral suspension | gabapentinoid |
| 30578978 | Gabapentin 6% cream | gabapentinoid |
| 62446979 | Gabapentin 6% gel | gabapentinoid |
| 90426998 | Gabapentin 600mg tablets | gabapentinoid |
| 92463990 | Gabapentin 600mg tablets | gabapentinoid |
| 95157990 | Gabapentin 600mg tablets | gabapentinoid |
| 95187990 | Gabapentin 600mg tablets | gabapentinoid |
| 98989998 | Gabapentin 600mg tablets | gabapentinoid |
| 90425998 | Gabapentin 600mg tablets and gabapentin 300mg capsules | gabapentinoid |
| 64951979 | Gabapentin 600mg/5ml oral solution | gabapentinoid |
| 64949979 | Gabapentin 600mg/5ml oral suspension | gabapentinoid |
| 90426997 | Gabapentin 800mg tablets | gabapentinoid |
| 95186990 | Gabapentin 800mg tablets | gabapentinoid |
| 98989997 | Gabapentin 800mg tablets | gabapentinoid |
| 85753998 | Gabapentin oral solution | gabapentinoid |
| 98429998 | Ibuprofen & codeine phosphate 300mg+20mg modified release tablets | weak_opioid |
| 98429998 | Ibuprofen & codeine phosphate 300mg+20mg modified release tablets | weak_opioid |
| 39055978 | Ibuprofen 10% gel | weak_nsaid |
| 39308978 | Ibuprofen 10% gel | weak_nsaid |
| 81664998 | Ibuprofen 10% gel | weak_nsaid |
| 89499998 | Ibuprofen 10% gel | weak_nsaid |
| 91155998 | Ibuprofen 10% gel | weak_nsaid |
| 91438979 | Ibuprofen 10% gel | weak_nsaid |
| 91523998 | Ibuprofen 10% gel | weak_nsaid |
| 91777998 | Ibuprofen 10% gel | weak_nsaid |
| 93235990 | Ibuprofen 10% gel | weak_nsaid |
| 94152998 | Ibuprofen 10% gel | weak_nsaid |
| 95172990 | Ibuprofen 10% gel | weak_nsaid |
| 98137998 | Ibuprofen 10% gel | weak_nsaid |
| 34034978 | Ibuprofen 100mg chewable capsules | weak_nsaid |
| 34035978 | Ibuprofen 100mg chewable capsules | weak_nsaid |
| 61416979 | Ibuprofen 100mg/5ml oral suspension | weak_nsaid |
| 81796998 | Ibuprofen 100mg/5ml oral suspension | weak_nsaid |
| 81797998 | Ibuprofen 100mg/5ml oral suspension | weak_nsaid |
| 93549998 | Ibuprofen 100mg/5ml oral suspension | weak_nsaid |
| 99868997 | Ibuprofen 100mg/5ml oral suspension | weak_nsaid |
| 85816979 | Ibuprofen 100mg/5ml oral suspension 5ml sachets sugar free | weak_nsaid |
| 85818979 | Ibuprofen 100mg/5ml oral suspension 5ml sachets sugar free | weak_nsaid |
| 85821979 | Ibuprofen 100mg/5ml oral suspension 5ml sachets sugar free | weak_nsaid |
| 85822979 | Ibuprofen 100mg/5ml oral suspension 5ml sachets sugar free | weak_nsaid |
| 86886998 | Ibuprofen 100mg/5ml oral suspension 5ml sachets sugar free | weak_nsaid |
| 91082998 | Ibuprofen 100mg/5ml oral suspension 5ml sachets sugar free | weak_nsaid |
| 39312978 | Ibuprofen 100mg/5ml oral suspension sugar free | weak_nsaid |
| 42596978 | Ibuprofen 100mg/5ml oral suspension sugar free | weak_nsaid |
| 50908978 | Ibuprofen 100mg/5ml oral suspension sugar free | weak_nsaid |
| 78568978 | Ibuprofen 100mg/5ml oral suspension sugar free | weak_nsaid |
| 78569978 | Ibuprofen 100mg/5ml oral suspension sugar free | weak_nsaid |
| 81958998 | Ibuprofen 100mg/5ml oral suspension sugar free | weak_nsaid |
| 84586998 | Ibuprofen 100mg/5ml oral suspension sugar free | weak_nsaid |
| 85815979 | Ibuprofen 100mg/5ml oral suspension sugar free | weak_nsaid |
| 86209998 | Ibuprofen 100mg/5ml oral suspension sugar free | weak_nsaid |
| 88012998 | Ibuprofen 100mg/5ml oral suspension sugar free | weak_nsaid |
| 88042998 | Ibuprofen 100mg/5ml oral suspension sugar free | weak_nsaid |
| 88894998 | Ibuprofen 100mg/5ml oral suspension sugar free | weak_nsaid |
| 89158998 | Ibuprofen 100mg/5ml oral suspension sugar free | weak_nsaid |
| 89420998 | Ibuprofen 100mg/5ml oral suspension sugar free | weak_nsaid |
| 90360998 | Ibuprofen 100mg/5ml oral suspension sugar free | weak_nsaid |
| 91813979 | Ibuprofen 100mg/5ml oral suspension sugar free | weak_nsaid |
| 91814979 | Ibuprofen 100mg/5ml oral suspension sugar free | weak_nsaid |
| 91815979 | Ibuprofen 100mg/5ml oral suspension sugar free | weak_nsaid |
| 91816979 | Ibuprofen 100mg/5ml oral suspension sugar free | weak_nsaid |
| 91819979 | Ibuprofen 100mg/5ml oral suspension sugar free | weak_nsaid |
| 91822979 | Ibuprofen 100mg/5ml oral suspension sugar free | weak_nsaid |
| 91823979 | Ibuprofen 100mg/5ml oral suspension sugar free | weak_nsaid |
| 91824979 | Ibuprofen 100mg/5ml oral suspension sugar free | weak_nsaid |
| 91825979 | Ibuprofen 100mg/5ml oral suspension sugar free | weak_nsaid |
| 91826979 | Ibuprofen 100mg/5ml oral suspension sugar free | weak_nsaid |
| 91827979 | Ibuprofen 100mg/5ml oral suspension sugar free | weak_nsaid |
| 91828979 | Ibuprofen 100mg/5ml oral suspension sugar free | weak_nsaid |
| 91829979 | Ibuprofen 100mg/5ml oral suspension sugar free | weak_nsaid |
| 93262998 | Ibuprofen 100mg/5ml oral suspension sugar free | weak_nsaid |
| 94016990 | Ibuprofen 100mg/5ml oral suspension sugar free | weak_nsaid |
| 94838990 | Ibuprofen 100mg/5ml oral suspension sugar free | weak_nsaid |
| 94886990 | Ibuprofen 100mg/5ml oral suspension sugar free | weak_nsaid |
| 94901990 | Ibuprofen 100mg/5ml oral suspension sugar free | weak_nsaid |
| 95634990 | Ibuprofen 100mg/5ml oral suspension sugar free | weak_nsaid |
| 96140990 | Ibuprofen 100mg/5ml oral suspension sugar free | weak_nsaid |
| 96140996 | Ibuprofen 100mg/5ml oral suspension sugar free | weak_nsaid |
| 96140997 | Ibuprofen 100mg/5ml oral suspension sugar free | weak_nsaid |
| 96341990 | Ibuprofen 100mg/5ml oral suspension sugar free | weak_nsaid |
| 98084998 | Ibuprofen 100mg/5ml oral suspension sugar free | weak_nsaid |
| 86329998 | Ibuprofen 10mg/2ml solution for infusion ampoules | weak_nsaid |
| 39313978 | Ibuprofen 200mg / Codeine 12.8mg tablets | weak_opioid |
| 86126998 | Ibuprofen 200mg / Codeine 12.8mg tablets | weak_opioid |
| 87495998 | Ibuprofen 200mg / Codeine 12.8mg tablets | weak_opioid |
| 89745998 | Ibuprofen 200mg / Codeine 12.8mg tablets | weak_opioid |
| 92121997 | Ibuprofen 200mg / Codeine 12.8mg tablets | weak_opioid |
| 92122998 | Ibuprofen 200mg / Codeine 12.8mg tablets | weak_opioid |
| 93048992 | Ibuprofen 200mg / Codeine 12.8mg tablets | weak_opioid |
| 39313978 | Ibuprofen 200mg / Codeine 12.8mg tablets | weak_opioid |
| 86126998 | Ibuprofen 200mg / Codeine 12.8mg tablets | weak_opioid |
| 87495998 | Ibuprofen 200mg / Codeine 12.8mg tablets | weak_opioid |
| 89745998 | Ibuprofen 200mg / Codeine 12.8mg tablets | weak_opioid |
| 92121997 | Ibuprofen 200mg / Codeine 12.8mg tablets | weak_opioid |
| 92122998 | Ibuprofen 200mg / Codeine 12.8mg tablets | weak_opioid |
| 93048992 | Ibuprofen 200mg / Codeine 12.8mg tablets | weak_opioid |
| 21744978 | Ibuprofen 200mg capsules | weak_nsaid |
| 39049978 | Ibuprofen 200mg capsules | weak_nsaid |
| 54501979 | Ibuprofen 200mg capsules | weak_nsaid |
| 82183978 | Ibuprofen 200mg capsules | weak_nsaid |
| 83155998 | Ibuprofen 200mg capsules | weak_nsaid |
| 84433998 | Ibuprofen 200mg capsules | weak_nsaid |
| 84553998 | Ibuprofen 200mg capsules | weak_nsaid |
| 88174998 | Ibuprofen 200mg capsules | weak_nsaid |
| 88527997 | Ibuprofen 200mg capsules | weak_nsaid |
| 90231979 | Ibuprofen 200mg capsules | weak_nsaid |
| 91081997 | Ibuprofen 200mg capsules | weak_nsaid |
| 92169998 | Ibuprofen 200mg capsules | weak_nsaid |
| 95143992 | Ibuprofen 200mg capsules | weak_nsaid |
| 81659998 | Ibuprofen 200mg effervescent tablets | weak_nsaid |
| 17775978 | Ibuprofen 200mg medicated plasters | weak_nsaid |
| 17776978 | Ibuprofen 200mg medicated plasters | weak_nsaid |
| 39311978 | Ibuprofen 200mg modified-release capsules | weak_nsaid |
| 89014998 | Ibuprofen 200mg modified-release capsules | weak_nsaid |
| 98764998 | Ibuprofen 200mg modified-release capsules | weak_nsaid |
| 89621998 | Ibuprofen 200mg orodispersible tablets sugar free | weak_nsaid |
| 92290998 | Ibuprofen 200mg orodispersible tablets sugar free | weak_nsaid |
| 94887998 | Ibuprofen 200mg orodispersible tablets sugar free | weak_nsaid |
| 17293978 | Ibuprofen 200mg tablets | weak_nsaid |
| 70267978 | Ibuprofen 200mg tablets | weak_nsaid |
| 83500998 | Ibuprofen 200mg tablets | weak_nsaid |
| 84155998 | Ibuprofen 200mg tablets | weak_nsaid |
| 84160998 | Ibuprofen 200mg tablets | weak_nsaid |
| 84490998 | Ibuprofen 200mg tablets | weak_nsaid |
| 84554998 | Ibuprofen 200mg tablets | weak_nsaid |
| 84555998 | Ibuprofen 200mg tablets | weak_nsaid |
| 86953998 | Ibuprofen 200mg tablets | weak_nsaid |
| 88233998 | Ibuprofen 200mg tablets | weak_nsaid |
| 88527998 | Ibuprofen 200mg tablets | weak_nsaid |
| 89117998 | Ibuprofen 200mg tablets | weak_nsaid |
| 89580998 | Ibuprofen 200mg tablets | weak_nsaid |
| 89691998 | Ibuprofen 200mg tablets | weak_nsaid |
| 90116998 | Ibuprofen 200mg tablets | weak_nsaid |
| 90125998 | Ibuprofen 200mg tablets | weak_nsaid |
| 91081996 | Ibuprofen 200mg tablets | weak_nsaid |
| 91120998 | Ibuprofen 200mg tablets | weak_nsaid |
| 91856979 | Ibuprofen 200mg tablets | weak_nsaid |
| 91863979 | Ibuprofen 200mg tablets | weak_nsaid |
| 91864979 | Ibuprofen 200mg tablets | weak_nsaid |
| 93218998 | Ibuprofen 200mg tablets | weak_nsaid |
| 93272997 | Ibuprofen 200mg tablets | weak_nsaid |
| 93272998 | Ibuprofen 200mg tablets | weak_nsaid |
| 93866998 | Ibuprofen 200mg tablets | weak_nsaid |
| 94240992 | Ibuprofen 200mg tablets | weak_nsaid |
| 94460990 | Ibuprofen 200mg tablets | weak_nsaid |
| 94627990 | Ibuprofen 200mg tablets | weak_nsaid |
| 95340990 | Ibuprofen 200mg tablets | weak_nsaid |
| 95348990 | Ibuprofen 200mg tablets | weak_nsaid |
| 96238998 | Ibuprofen 200mg tablets | weak_nsaid |
| 96310990 | Ibuprofen 200mg tablets | weak_nsaid |
| 96369990 | Ibuprofen 200mg tablets | weak_nsaid |
| 96405998 | Ibuprofen 200mg tablets | weak_nsaid |
| 96583989 | Ibuprofen 200mg tablets | weak_nsaid |
| 96939990 | Ibuprofen 200mg tablets | weak_nsaid |
| 96984989 | Ibuprofen 200mg tablets | weak_nsaid |
| 97056998 | Ibuprofen 200mg tablets | weak_nsaid |
| 97104990 | Ibuprofen 200mg tablets | weak_nsaid |
| 97106998 | Ibuprofen 200mg tablets | weak_nsaid |
| 97126998 | Ibuprofen 200mg tablets | weak_nsaid |
| 97594998 | Ibuprofen 200mg tablets | weak_nsaid |
| 97358998 | Ibuprofen 200mg tablets | weak_nsaid |
| 97551990 | Ibuprofen 200mg tablets | weak_nsaid |
| 97674990 | Ibuprofen 200mg tablets | weak_nsaid |
| 97906998 | Ibuprofen 200mg tablets | weak_nsaid |
| 97923990 | Ibuprofen 200mg tablets | weak_nsaid |
| 98529990 | Ibuprofen 200mg tablets | weak_nsaid |
| 98530990 | Ibuprofen 200mg tablets | weak_nsaid |
| 98555990 | Ibuprofen 200mg tablets | weak_nsaid |
| 98673988 | Ibuprofen 200mg tablets | weak_nsaid |
| 99301996 | Ibuprofen 200mg tablets | weak_nsaid |
| 99466997 | Ibuprofen 200mg tablets | weak_nsaid |
| 99557990 | Ibuprofen 200mg tablets | weak_nsaid |
| 99558990 | Ibuprofen 200mg tablets | weak_nsaid |
| 99868998 | Ibuprofen 200mg tablets | weak_nsaid |
| 84104998 | Ibuprofen 200mg/5ml oral suspension | weak_nsaid |
| 14567978 | Ibuprofen 200mg/5ml oral suspension sugar free | weak_nsaid |
| 96238997 | Ibuprofen 300mg modified release capsules | weak_nsaid |
| 97083998 | Ibuprofen 300mg modified-release / codeine 20mg tablets | weak_opioid |
| 97083998 | Ibuprofen 300mg modified-release / codeine 20mg tablets | weak_opioid |
| 84758998 | Ibuprofen 300mg modified-release capsules | weak_nsaid |
| 87070998 | Ibuprofen 300mg modified-release capsules | weak_nsaid |
| 89760998 | Ibuprofen 300mg modified-release capsules | weak_nsaid |
| 96140998 | Ibuprofen 300mg modified-release capsules | weak_nsaid |
| 96278992 | Ibuprofen 300mg modified-release capsules | weak_nsaid |
| 98578998 | Ibuprofen 300mg modified-release capsules | weak_nsaid |
| 83154998 | Ibuprofen 400mg capsules | weak_nsaid |
| 84091978 | Ibuprofen 400mg capsules | weak_nsaid |
| 84435998 | Ibuprofen 400mg capsules | weak_nsaid |
| 85741998 | Ibuprofen 400mg capsules | weak_nsaid |
| 85783998 | Ibuprofen 400mg capsules | weak_nsaid |
| 85784998 | Ibuprofen 400mg capsules | weak_nsaid |
| 91081998 | Ibuprofen 400mg granules | weak_nsaid |
| 53244979 | Ibuprofen 400mg sachets | weak_nsaid |
| 17291978 | Ibuprofen 400mg tablets | weak_nsaid |
| 73226978 | Ibuprofen 400mg tablets | weak_nsaid |
| 83433998 | Ibuprofen 400mg tablets | weak_nsaid |
| 83499998 | Ibuprofen 400mg tablets | weak_nsaid |
| 84153998 | Ibuprofen 400mg tablets | weak_nsaid |
| 86171998 | Ibuprofen 400mg tablets | weak_nsaid |
| 88145996 | Ibuprofen 400mg tablets | weak_nsaid |
| 88284998 | Ibuprofen 400mg tablets | weak_nsaid |
| 89593997 | Ibuprofen 400mg tablets | weak_nsaid |
| 89691997 | Ibuprofen 400mg tablets | weak_nsaid |
| 90119997 | Ibuprofen 400mg tablets | weak_nsaid |
| 90869998 | Ibuprofen 400mg tablets | weak_nsaid |
| 91843979 | Ibuprofen 400mg tablets | weak_nsaid |
| 91844979 | Ibuprofen 400mg tablets | weak_nsaid |
| 91847979 | Ibuprofen 400mg tablets | weak_nsaid |
| 91850979 | Ibuprofen 400mg tablets | weak_nsaid |
| 91851979 | Ibuprofen 400mg tablets | weak_nsaid |
| 91853979 | Ibuprofen 400mg tablets | weak_nsaid |
| 91854979 | Ibuprofen 400mg tablets | weak_nsaid |
| 92059990 | Ibuprofen 400mg tablets | weak_nsaid |
| 92965998 | Ibuprofen 400mg tablets | weak_nsaid |
| 93756990 | Ibuprofen 400mg tablets | weak_nsaid |
| 94459990 | Ibuprofen 400mg tablets | weak_nsaid |
| 94626990 | Ibuprofen 400mg tablets | weak_nsaid |
| 94651992 | Ibuprofen 400mg tablets | weak_nsaid |
| 94678990 | Ibuprofen 400mg tablets | weak_nsaid |
| 95347990 | Ibuprofen 400mg tablets | weak_nsaid |
| 95367990 | Ibuprofen 400mg tablets | weak_nsaid |
| 96310989 | Ibuprofen 400mg tablets | weak_nsaid |
| 96405997 | Ibuprofen 400mg tablets | weak_nsaid |
| 96418989 | Ibuprofen 400mg tablets | weak_nsaid |
| 96558988 | Ibuprofen 400mg tablets | weak_nsaid |
| 96583988 | Ibuprofen 400mg tablets | weak_nsaid |
| 96939989 | Ibuprofen 400mg tablets | weak_nsaid |
| 96984988 | Ibuprofen 400mg tablets | weak_nsaid |
| 97005990 | Ibuprofen 400mg tablets | weak_nsaid |
| 97056997 | Ibuprofen 400mg tablets | weak_nsaid |
| 97104989 | Ibuprofen 400mg tablets | weak_nsaid |
| 97106997 | Ibuprofen 400mg tablets | weak_nsaid |
| 97107997 | Ibuprofen 400mg tablets | weak_nsaid |
| 97126997 | Ibuprofen 400mg tablets | weak_nsaid |
| 97594997 | Ibuprofen 400mg tablets | weak_nsaid |
| 97358997 | Ibuprofen 400mg tablets | weak_nsaid |
| 97551989 | Ibuprofen 400mg tablets | weak_nsaid |
| 97674989 | Ibuprofen 400mg tablets | weak_nsaid |
| 97678997 | Ibuprofen 400mg tablets | weak_nsaid |
| 97906997 | Ibuprofen 400mg tablets | weak_nsaid |
| 97963990 | Ibuprofen 400mg tablets | weak_nsaid |
| 98516998 | Ibuprofen 400mg tablets | weak_nsaid |
| 98529989 | Ibuprofen 400mg tablets | weak_nsaid |
| 98530989 | Ibuprofen 400mg tablets | weak_nsaid |
| 98555989 | Ibuprofen 400mg tablets | weak_nsaid |
| 98673989 | Ibuprofen 400mg tablets | weak_nsaid |
| 99466996 | Ibuprofen 400mg tablets | weak_nsaid |
| 99557989 | Ibuprofen 400mg tablets | weak_nsaid |
| 99558988 | Ibuprofen 400mg tablets | weak_nsaid |
| 59248978 | Ibuprofen 5% / Levomenthol 3% gel | weak_nsaid |
| 91098998 | Ibuprofen 5% / Levomenthol 3% gel | weak_nsaid |
| 92599998 | Ibuprofen 5% / Levomenthol 3% gel | weak_nsaid |
| 92600998 | Ibuprofen 5% / Levomenthol 3% gel | weak_nsaid |
| 84058998 | Ibuprofen 5% cream | weak_nsaid |
| 93625998 | Ibuprofen 5% cream | weak_nsaid |
| 93626998 | Ibuprofen 5% cream | weak_nsaid |
| 88205998 | Ibuprofen 5% foam | weak_nsaid |
| 94916998 | Ibuprofen 5% foam | weak_nsaid |
| 39056978 | Ibuprofen 5% gel | weak_nsaid |
| 39309978 | Ibuprofen 5% gel | weak_nsaid |
| 47189978 | Ibuprofen 5% gel | weak_nsaid |
| 82686998 | Ibuprofen 5% gel | weak_nsaid |
| 84019998 | Ibuprofen 5% gel | weak_nsaid |
| 84174998 | Ibuprofen 5% gel | weak_nsaid |
| 86759998 | Ibuprofen 5% gel | weak_nsaid |
| 86940998 | Ibuprofen 5% gel | weak_nsaid |
| 88143998 | Ibuprofen 5% gel | weak_nsaid |
| 88145998 | Ibuprofen 5% gel | weak_nsaid |
| 89139998 | Ibuprofen 5% gel | weak_nsaid |
| 89291998 | Ibuprofen 5% gel | weak_nsaid |
| 89472997 | Ibuprofen 5% gel | weak_nsaid |
| 89993998 | Ibuprofen 5% gel | weak_nsaid |
| 91473979 | Ibuprofen 5% gel | weak_nsaid |
| 91475979 | Ibuprofen 5% gel | weak_nsaid |
| 91476979 | Ibuprofen 5% gel | weak_nsaid |
| 91477979 | Ibuprofen 5% gel | weak_nsaid |
| 93626997 | Ibuprofen 5% gel | weak_nsaid |
| 95992990 | Ibuprofen 5% gel | weak_nsaid |
| 96237998 | Ibuprofen 5% gel | weak_nsaid |
| 96583990 | Ibuprofen 5% gel | weak_nsaid |
| 97062997 | Ibuprofen 5% gel | weak_nsaid |
| 97062998 | Ibuprofen 5% gel | weak_nsaid |
| 97801996 | Ibuprofen 5% gel | weak_nsaid |
| 88204998 | Ibuprofen 5% mousse | weak_nsaid |
| 82272998 | Ibuprofen 5% spray | weak_nsaid |
| 93626996 | Ibuprofen 5% spray | weak_nsaid |
| 97062996 | Ibuprofen 5% spray | weak_nsaid |
| 97079998 | Ibuprofen 5% spray | weak_nsaid |
| 94805997 | Ibuprofen 600mg effervescent granules sachets | weak_nsaid |
| 99301997 | Ibuprofen 600mg effervescent granules sachets | weak_nsaid |
| 91836979 | Ibuprofen 600mg tablets | weak_nsaid |
| 93390990 | Ibuprofen 600mg tablets | weak_nsaid |
| 96139998 | Ibuprofen 600mg tablets | weak_nsaid |
| 96405996 | Ibuprofen 600mg tablets | weak_nsaid |
| 96558990 | Ibuprofen 600mg tablets | weak_nsaid |
| 96939988 | Ibuprofen 600mg tablets | weak_nsaid |
| 97056996 | Ibuprofen 600mg tablets | weak_nsaid |
| 97104988 | Ibuprofen 600mg tablets | weak_nsaid |
| 97126996 | Ibuprofen 600mg tablets | weak_nsaid |
| 97593996 | Ibuprofen 600mg tablets | weak_nsaid |
| 97906996 | Ibuprofen 600mg tablets | weak_nsaid |
| 98150990 | Ibuprofen 600mg tablets | weak_nsaid |
| 98151990 | Ibuprofen 600mg tablets | weak_nsaid |
| 98515998 | Ibuprofen 600mg tablets | weak_nsaid |
| 98528990 | Ibuprofen 600mg tablets | weak_nsaid |
| 98529988 | Ibuprofen 600mg tablets | weak_nsaid |
| 98530988 | Ibuprofen 600mg tablets | weak_nsaid |
| 98673990 | Ibuprofen 600mg tablets | weak_nsaid |
| 99557988 | Ibuprofen 600mg tablets | weak_nsaid |
| 99558989 | Ibuprofen 600mg tablets | weak_nsaid |
| 83471998 | Ibuprofen 800mg modified-release tablets | weak_nsaid |
| 91833979 | Ibuprofen 800mg modified-release tablets | weak_nsaid |
| 91834979 | Ibuprofen 800mg modified-release tablets | weak_nsaid |
| 94805998 | Ibuprofen 800mg modified-release tablets | weak_nsaid |
| 99301998 | Ibuprofen 800mg modified-release tablets | weak_nsaid |
| 94874998 | Ibuprofen 800mg tablets | weak_nsaid |
| 94875998 | Ibuprofen 800mg tablets | weak_nsaid |
| 92121998 | Ibuprofen and codeine 200mg + 12.5mg tablet | weak_opioid |
| 92121998 | Ibuprofen and codeine 200mg + 12.5mg tablet | weak_opioid |
| 82366998 | Ibuprofen lysine 200mg tablets | weak_nsaid |
| 84437998 | Ibuprofen lysine 200mg tablets | weak_nsaid |
| 85891979 | Ibuprofen lysine 200mg tablets | weak_nsaid |
| 86594998 | Ibuprofen lysine 200mg tablets | weak_nsaid |
| 88228998 | Ibuprofen lysine 200mg tablets | weak_nsaid |
| 89890998 | Ibuprofen lysine 200mg tablets | weak_nsaid |
| 53245979 | Ibuprofen lysine 400mg oral powder sachets | weak_nsaid |
| 84434998 | Ibuprofen lysine 400mg tablets | weak_nsaid |
| 86170998 | Ibuprofen lysine 400mg tablets | weak_nsaid |
| 93272996 | Ibuprofen lysine 400mg tablets | weak_nsaid |
| 13633978 | Ibuprofen Seven Plus Pain Relief 200mg/5ml oral suspension | weak_nsaid |
| 38862978 | Ibuprofen sodium dihydrate 200mg tablets | weak_nsaid |
| 38863978 | Ibuprofen sodium dihydrate 200mg tablets | weak_nsaid |
| 83494998 | Ibuprofen sodium dihydrate 200mg tablets | weak_nsaid |
| 83497998 | Ibuprofen sodium dihydrate 200mg tablets | weak_nsaid |
| 14566978 | Ibuprofen Twelve Plus Pain Relief 200mg/5ml oral suspension | weak_nsaid |
| 97608992 | Ipecacuanha & morphine conc 1-4 mix | strong_opioid |
| 93845998 | Ipecacuanha and morphine mixture | strong_opioid |
| 98049998 | Kaolin and Morphine mixture | strong_opioid |
| 99542990 | Kaolin and Morphine mixture | strong_opioid |
| 94104998 | Kaolin light 1.5g/5ml / codeine 5mg/5ml oral suspension sugar free | weak_opioid |
| 60313979 | Morphine 0.1% gel | strong_opioid |
| 79909978 | Morphine 0.1% in intrasite gel | strong_opioid |
| 76364978 | Morphine 0.2% in intrasite gel | strong_opioid |
| 83354998 | Morphine 100mg modified-release capsules | strong_opioid |
| 88473998 | Morphine 100mg modified-release capsules | strong_opioid |
| 89678996 | Morphine 100mg modified-release capsules | strong_opioid |
| 89679996 | Morphine 100mg modified-release capsules | strong_opioid |
| 90759998 | Morphine 100mg modified-release capsules | strong_opioid |
| 91187998 | Morphine 100mg modified-release capsules | strong_opioid |
| 92773997 | Morphine 100mg modified-release granules sachets sugar free | strong_opioid |
| 92774997 | Morphine 100mg modified-release granules sachets sugar free | strong_opioid |
| 89903998 | Morphine 100mg modified-release tablets | strong_opioid |
| 95789998 | Morphine 100mg modified-release tablets | strong_opioid |
| 96095992 | Morphine 100mg modified-release tablets | strong_opioid |
| 96344992 | Morphine 100mg modified-release tablets | strong_opioid |
| 98443998 | Morphine 100mg modified-release tablets | strong_opioid |
| 99395998 | Morphine 100mg modified-release tablets | strong_opioid |
| 83356998 | Morphine 10mg modified-release capsules | strong_opioid |
| 88474998 | Morphine 10mg modified-release capsules | strong_opioid |
| 91201998 | Morphine 10mg modified-release capsules | strong_opioid |
| 89904998 | Morphine 10mg modified-release tablets | strong_opioid |
| 91856998 | Morphine 10mg modified-release tablets | strong_opioid |
| 92536998 | Morphine 10mg modified-release tablets | strong_opioid |
| 95160998 | Morphine 10mg modified-release tablets | strong_opioid |
| 95421979 | Morphine 10mg modified-release tablets | strong_opioid |
| 95790998 | Morphine 10mg modified-release tablets | strong_opioid |
| 99396998 | Morphine 10mg modified-release tablets | strong_opioid |
| 93183998 | Morphine 10mg tablets | strong_opioid |
| 93184998 | Morphine 10mg tablets | strong_opioid |
| 95792998 | Morphine 10mg/ml tincture | strong_opioid |
| 83352998 | Morphine 120mg modified-release capsules | strong_opioid |
| 90327998 | Morphine 120mg modified-release capsules | strong_opioid |
| 90353998 | Morphine 120mg modified-release capsules | strong_opioid |
| 83351998 | Morphine 150mg modified-release capsules | strong_opioid |
| 90327997 | Morphine 150mg modified-release capsules | strong_opioid |
| 90353997 | Morphine 150mg modified-release capsules | strong_opioid |
| 96840992 | Morphine 15mg modified-release tablets | strong_opioid |
| 97791998 | Morphine 15mg modified-release tablets | strong_opioid |
| 97793998 | Morphine 15mg modified-release tablets | strong_opioid |
| 93123998 | Morphine 1mg/5ml / peppermint oil 1.5microlitres/5ml oral solution | strong_opioid |
| 93277997 | Morphine 1mg/5ml / Peppermint oil 1.5microlitres/5ml oral solution | strong_opioid |
| 83349998 | Morphine 200mg modified-release capsules | strong_opioid |
| 88473997 | Morphine 200mg modified-release capsules | strong_opioid |
| 90327996 | Morphine 200mg modified-release capsules | strong_opioid |
| 90353996 | Morphine 200mg modified-release capsules | strong_opioid |
| 91187997 | Morphine 200mg modified-release capsules | strong_opioid |
| 92773996 | Morphine 200mg modified-release granules sachets sugar free | strong_opioid |
| 92774996 | Morphine 200mg modified-release granules sachets sugar free | strong_opioid |
| 95789997 | Morphine 200mg modified-release tablets | strong_opioid |
| 96343992 | Morphine 200mg modified-release tablets | strong_opioid |
| 96837992 | Morphine 200mg modified-release tablets | strong_opioid |
| 99395997 | Morphine 200mg modified-release tablets | strong_opioid |
| 95412979 | Morphine 20mg modified-release capsules | strong_opioid |
| 96849992 | Morphine 20mg modified-release granules sachets sugar free | strong_opioid |
| 96854992 | Morphine 20mg modified-release granules sachets sugar free | strong_opioid |
| 97791997 | Morphine 20mg modified-release granules sachets sugar free | strong_opioid |
| 97793997 | Morphine 20mg modified-release granules sachets sugar free | strong_opioid |
| 93183997 | Morphine 20mg tablets | strong_opioid |
| 93184997 | Morphine 20mg tablets | strong_opioid |
| 83359998 | Morphine 30mg modified-release capsules | strong_opioid |
| 88474997 | Morphine 30mg modified-release capsules | strong_opioid |
| 90352998 | Morphine 30mg modified-release capsules | strong_opioid |
| 90354998 | Morphine 30mg modified-release capsules | strong_opioid |
| 91201997 | Morphine 30mg modified-release capsules | strong_opioid |
| 96848992 | Morphine 30mg modified-release granules sachets sugar free | strong_opioid |
| 96853992 | Morphine 30mg modified-release granules sachets sugar free | strong_opioid |
| 97791996 | Morphine 30mg modified-release granules sachets sugar free | strong_opioid |
| 97793996 | Morphine 30mg modified-release granules sachets sugar free | strong_opioid |
| 89905998 | Morphine 30mg modified-release tablets | strong_opioid |
| 91855998 | Morphine 30mg modified-release tablets | strong_opioid |
| 92524998 | Morphine 30mg modified-release tablets | strong_opioid |
| 95160997 | Morphine 30mg modified-release tablets | strong_opioid |
| 95790997 | Morphine 30mg modified-release tablets | strong_opioid |
| 96078992 | Morphine 30mg modified-release tablets | strong_opioid |
| 96839992 | Morphine 30mg modified-release tablets | strong_opioid |
| 99396997 | Morphine 30mg modified-release tablets | strong_opioid |
| 93183996 | Morphine 50mg tablets | strong_opioid |
| 93322996 | Morphine 50mg tablets | strong_opioid |
| 95789996 | Morphine 5mg modified-release tablets | strong_opioid |
| 96841992 | Morphine 5mg modified-release tablets | strong_opioid |
| 99395996 | Morphine 5mg modified-release tablets | strong_opioid |
| 96347992 | Morphine 60 mg sup | strong_opioid |
| 83624998 | Morphine 60mg modified-release capsules | strong_opioid |
| 88474996 | Morphine 60mg modified-release capsules | strong_opioid |
| 90352997 | Morphine 60mg modified-release capsules | strong_opioid |
| 90354997 | Morphine 60mg modified-release capsules | strong_opioid |
| 91201996 | Morphine 60mg modified-release capsules | strong_opioid |
| 92773998 | Morphine 60mg modified-release granules sachets sugar free | strong_opioid |
| 92774998 | Morphine 60mg modified-release granules sachets sugar free | strong_opioid |
| 89955998 | Morphine 60mg modified-release tablets | strong_opioid |
| 95790996 | Morphine 60mg modified-release tablets | strong_opioid |
| 96838992 | Morphine 60mg modified-release tablets | strong_opioid |
| 99148998 | Morphine 60mg modified-release tablets | strong_opioid |
| 99396996 | Morphine 60mg modified-release tablets | strong_opioid |
| 95791998 | Morphine 8.4mg/ml elixir | strong_opioid |
| 95788998 | Morphine 8.4mg/ml injection | strong_opioid |
| 83355998 | Morphine 90mg modified-release capsules | strong_opioid |
| 90352996 | Morphine 90mg modified-release capsules | strong_opioid |
| 90354996 | Morphine 90mg modified-release capsules | strong_opioid |
| 97771992 | Morphine anhydrous 8.4 mg eli | strong_opioid |
| 96346992 | Morphine anhydrous 8.4 mg inj | strong_opioid |
| 96345992 | Morphine hcl 10 mg inj | strong_opioid |
| 97770992 | Morphine hcl 30 mg inj | strong_opioid |
| 93350992 | Morphine hcl 5mg/chloroform water to 5ml sol | strong_opioid |
| 95663998 | Morphine hcl and activated attapulgite and attapulgite tablet | strong_opioid |
| 93291998 | Morphine hcl and kaolin and belladonna tablet | strong_opioid |
| 93282998 | Morphine hcl and light kaolin and calcium carbonate tablet | strong_opioid |
| 93122998 | Morphine hcl light kaolin, belladonna and aluminium hydroxide chewable tablet | strong_opioid |
| 98817989 | Morphine hcl sugar free oral solution | strong_opioid |
| 99489989 | Morphine hcl sugar free oral solution | strong_opioid |
| 68975979 | Morphine hydrochloride 100mg/5ml oral solution | strong_opioid |
| 68972979 | Morphine hydrochloride 10mg/5ml oral solution | strong_opioid |
| 68973979 | Morphine hydrochloride 10mg/5ml oral solution | strong_opioid |
| 98081998 | Morphine hydrochloride 15mg suppositories | strong_opioid |
| 53708979 | Morphine hydrochloride 1mg/1ml solution for injection ampoules | strong_opioid |
| 98081997 | Morphine hydrochloride 30mg suppositories | strong_opioid |
| 68965979 | Morphine hydrochloride 5mg/5ml oral solution | strong_opioid |
| 53704979 | Morphine hydrochloride 5mg/5ml solution for injection ampoules | strong_opioid |
| 98817990 | Morphine hydrochloride powder | strong_opioid |
| 68927979 | Morphine sulfate 1.5mg/5ml oral solution | strong_opioid |
| 92796998 | Morphine sulfate 100mg modified release tablets | strong_opioid |
| 91634997 | Morphine sulfate 100mg/50ml solution for infusion vials | strong_opioid |
| 98418998 | Morphine sulfate 100mg/5ml oral solution unit dose vials sugar free | strong_opioid |
| 94806996 | Morphine sulfate 100mg/5ml unit dose vial | strong_opioid |
| 91271998 | Morphine sulfate 10mg modified release capsules | strong_opioid |
| 92800998 | Morphine sulfate 10mg modified release tablets | strong_opioid |
| 93184996 | Morphine sulfate 10mg suppositories | strong_opioid |
| 97019996 | Morphine sulfate 10mg suppositories | strong_opioid |
| 97847990 | Morphine sulfate 10mg suppositories | strong_opioid |
| 39423978 | Morphine sulfate 10mg/10ml solution for injection ampoules | strong_opioid |
| 53700979 | Morphine sulfate 10mg/10ml solution for injection ampoules | strong_opioid |
| 83756978 | Morphine sulfate 10mg/10ml solution for injection ampoules | strong_opioid |
| 85680998 | Morphine sulfate 10mg/1ml solution for injection ampoules | strong_opioid |
| 90258979 | Morphine sulfate 10mg/1ml solution for injection ampoules | strong_opioid |
| 90260979 | Morphine sulfate 10mg/1ml solution for injection ampoules | strong_opioid |
| 90261979 | Morphine sulfate 10mg/1ml solution for injection ampoules | strong_opioid |
| 93447990 | Morphine sulfate 10mg/1ml solution for injection ampoules | strong_opioid |
| 96240990 | Morphine sulfate 10mg/1ml solution for injection ampoules | strong_opioid |
| 97044998 | Morphine sulfate 10mg/1ml solution for injection ampoules | strong_opioid |
| 99147989 | Morphine sulfate 10mg/1ml solution for injection ampoules | strong_opioid |
| 83052998 | Morphine sulfate 10mg/1ml suspension for injection vials | strong_opioid |
| 68111979 | Morphine sulfate 10mg/2ml solution for injection ampoules | strong_opioid |
| 30020978 | Morphine sulfate 10mg/5ml oral solution | strong_opioid |
| 53668979 | Morphine sulfate 10mg/5ml oral solution | strong_opioid |
| 84319998 | Morphine sulfate 10mg/5ml oral solution | strong_opioid |
| 92302998 | Morphine sulfate 10mg/5ml oral solution | strong_opioid |
| 93349992 | Morphine sulfate 10mg/5ml oral solution | strong_opioid |
| 94772998 | Morphine sulfate 10mg/5ml oral solution | strong_opioid |
| 94807998 | Morphine sulfate 10mg/5ml oral solution | strong_opioid |
| 95646979 | Morphine sulfate 10mg/5ml oral solution | strong_opioid |
| 95648979 | Morphine sulfate 10mg/5ml oral solution | strong_opioid |
| 96078990 | Morphine sulfate 10mg/5ml oral solution | strong_opioid |
| 94772997 | Morphine sulfate 10mg/5ml oral solution unit dose vials sugar free | strong_opioid |
| 94806998 | Morphine sulfate 10mg/5ml oral solution unit dose vials sugar free | strong_opioid |
| 94211998 | Morphine sulfate 15mg suppositories | strong_opioid |
| 97019998 | Morphine sulfate 15mg suppositories | strong_opioid |
| 99488988 | Morphine sulfate 15mg suppositories | strong_opioid |
| 99806989 | Morphine sulfate 15mg suppositories | strong_opioid |
| 93445990 | Morphine sulfate 15mg/1ml solution for injection ampoules | strong_opioid |
| 97044997 | Morphine sulfate 15mg/1ml solution for injection ampoules | strong_opioid |
| 86202998 | Morphine sulfate 1mg/ml prefilled syringes | strong_opioid |
| 86203998 | Morphine sulfate 1mg/ml prefilled syringes | strong_opioid |
| 18669978 | Morphine sulfate 2.5mg/5ml oral solution | strong_opioid |
| 68107979 | Morphine sulfate 2.5mg/5ml solution for injection ampoules | strong_opioid |
| 89195998 | Morphine sulfate 20mg modified release capsules | strong_opioid |
| 89679998 | Morphine sulfate 20mg modified release capsules | strong_opioid |
| 97761998 | Morphine sulfate 20mg suppositories | strong_opioid |
| 91634996 | Morphine sulfate 20mg/1ml solution for injection ampoules | strong_opioid |
| 96784990 | Morphine sulfate 20mg/1ml solution for injection ampoules | strong_opioid |
| 68105979 | Morphine sulfate 20mg/2ml solution for injection ampoules | strong_opioid |
| 68687979 | Morphine sulfate 20mg/5ml oral solution | strong_opioid |
| 92302997 | Morphine sulfate 20mg/ml oral solution sugar free | strong_opioid |
| 93609998 | Morphine sulfate 20mg/ml oral solution sugar free | strong_opioid |
| 94800998 | Morphine sulfate 20mg/ml oral solution sugar free | strong_opioid |
| 68921979 | Morphine sulfate 2mg/5ml oral solution | strong_opioid |
| 92800997 | Morphine sulfate 30mg modified release tablets | strong_opioid |
| 94211997 | Morphine sulfate 30mg suppositories | strong_opioid |
| 97019997 | Morphine sulfate 30mg suppositories | strong_opioid |
| 86104998 | Morphine sulfate 30mg/1ml solution for injection ampoules | strong_opioid |
| 93552990 | Morphine sulfate 30mg/1ml solution for injection ampoules | strong_opioid |
| 97044996 | Morphine sulfate 30mg/1ml solution for injection ampoules | strong_opioid |
| 99146990 | Morphine sulfate 30mg/1ml solution for injection ampoules | strong_opioid |
| 99430989 | Morphine sulfate 30mg/1ml solution for injection ampoules | strong_opioid |
| 86201998 | Morphine sulfate 30mg/30ml solution for infusion vials | strong_opioid |
| 94772996 | Morphine sulfate 30mg/5ml oral solution unit dose vials sugar free | strong_opioid |
| 94806997 | Morphine sulfate 30mg/5ml vials | strong_opioid |
| 90737998 | Morphine sulfate 35mg tampons | strong_opioid |
| 68297979 | Morphine sulfate 500micrograms/5ml oral solution | strong_opioid |
| 88728998 | Morphine sulfate 50mg modified release capsules | strong_opioid |
| 89679997 | Morphine sulfate 50mg modified release capsules | strong_opioid |
| 79939978 | Morphine sulfate 50mg/1ml solution for injection ampoules | strong_opioid |
| 86200998 | Morphine sulfate 50mg/50ml solution for infusion vials | strong_opioid |
| 91634998 | Morphine sulfate 50mg/50ml solution for infusion vials | strong_opioid |
| 53316979 | Morphine sulfate 50mg/5ml solution for injection ampoules | strong_opioid |
| 68095979 | Morphine sulfate 5mg/10ml solution for injection ampoules | strong_opioid |
| 68093979 | Morphine sulfate 5mg/1ml solution for injection ampoules | strong_opioid |
| 68946979 | Morphine sulfate 5mg/5ml oral solution | strong_opioid |
| 68091979 | Morphine sulfate 5mg/5ml solution for injection ampoules | strong_opioid |
| 92800996 | Morphine sulfate 60mg modified release tablets | strong_opioid |
| 86103998 | Morphine sulfate 60mg/2ml solution for injection ampoules | strong_opioid |
| 96079992 | Morphine sulfate 60mg/2ml solution for injection ampoules | strong_opioid |
| 98835989 | Morphine sulfate 60mg/2ml solution for injection ampoules | strong_opioid |
| 98835990 | Morphine sulfate 60mg/2ml solution for injection ampoules | strong_opioid |
| 93051992 | Morphine sulphate 100 mg cap | strong_opioid |
| 96350992 | Morphine sulphate 100 mg sup | strong_opioid |
| 89678998 | Morphine sulphate 12 20mg modified-release capsule | strong_opioid |
| 89678997 | Morphine sulphate 12 50mg modified-release capsule | strong_opioid |
| 93094992 | Morphine sulphate 120 mg cap | strong_opioid |
| 99945992 | Morphine sulphate 15 mg tab | strong_opioid |
| 93104992 | Morphine sulphate 150 mg cap | strong_opioid |
| 93042992 | Morphine sulphate 20 mg cap | strong_opioid |
| 93103992 | Morphine sulphate 200 mg cap | strong_opioid |
| 93456998 | Morphine sulphate 20mg/ml injection | strong_opioid |
| 97779992 | Morphine sulphate 4 mg inj | strong_opioid |
| 97772992 | Morphine sulphate 5 mg inj | strong_opioid |
| 97778992 | Morphine sulphate 5 mg sup | strong_opioid |
| 93041992 | Morphine sulphate 50 mg cap | strong_opioid |
| 97780992 | Morphine sulphate 50 mg sup | strong_opioid |
| 97775992 | Morphine sulphate 60 mg sup | strong_opioid |
| 93095992 | Morphine sulphate 90 mg cap | strong_opioid |
| 97631998 | Morphine sulphate and atropine 10mg + 600microgram/ml injection | strong_opioid |
| 96349992 | Morphine sulphate epidural 2 mg inj | strong_opioid |
| 97776992 | Morphine sulphate sup | strong_opioid |
| 96053992 | Morphine sulphate susp c/r 30 mg | strong_opioid |
| 95445998 | Morphine tartrate 10mg/1ml / Cyclizine tartrate 50mg/1ml solution for injection ampoules | strong_opioid |
| 96590998 | Morphine tartrate 10mg/1ml / Cyclizine tartrate 50mg/1ml solution for injection ampoules | strong_opioid |
| 97305998 | Morphine tartrate 10mg/1ml / Cyclizine tartrate 50mg/1ml solution for injection ampoules | strong_opioid |
| 95445997 | Morphine tartrate 15mg/1ml / Cyclizine tartrate 50mg/1ml solution for injection ampoules | strong_opioid |
| 96590997 | Morphine tartrate 15mg/1ml / Cyclizine tartrate 50mg/1ml solution for injection ampoules | strong_opioid |
| 96716992 | Morphine tartrate 15mg/1ml / Cyclizine tartrate 50mg/1ml solution for injection ampoules | strong_opioid |
| 97304998 | Morphine tartrate 15mg/1ml / Cyclizine tartrate 50mg/1ml solution for injection ampoules | strong_opioid |
| 93844998 | Morphine with ipecacuanha mixture | strong_opioid |
| 95313998 | Naproxen & misoprostol (1x56+56) 500mg+200mcg tablets | strong_nsaid |
| 88127998 | Naproxen & misoprostol 500mg+200mcg tablets | strong_nsaid |
| 68284979 | Naproxen 100mg/5ml oral suspension | strong_nsaid |
| 31117978 | Naproxen 125mg/5ml oral suspension | strong_nsaid |
| 31118978 | Naproxen 125mg/5ml oral suspension | strong_nsaid |
| 95754997 | Naproxen 125mg/5ml oral suspension | strong_nsaid |
| 97566997 | Naproxen 125mg/5ml oral suspension | strong_nsaid |
| 83070998 | Naproxen 200mg/5ml oral suspension | strong_nsaid |
| 95300992 | NAPROXEN 250 MG CAP | strong_nsaid |
| 38697978 | Naproxen 250mg effervescent tablets sugar free | strong_nsaid |
| 59406979 | Naproxen 250mg gastro-resistant tablets | strong_nsaid |
| 83984998 | Naproxen 250mg gastro-resistant tablets | strong_nsaid |
| 87978998 | Naproxen 250mg gastro-resistant tablets | strong_nsaid |
| 88139998 | Naproxen 250mg gastro-resistant tablets | strong_nsaid |
| 93169998 | Naproxen 250mg gastro-resistant tablets | strong_nsaid |
| 93170998 | Naproxen 250mg gastro-resistant tablets | strong_nsaid |
| 95093998 | Naproxen 250mg gastro-resistant tablets | strong_nsaid |
| 96879990 | Naproxen 250mg gastro-resistant tablets | strong_nsaid |
| 97100990 | Naproxen 250mg gastro-resistant tablets | strong_nsaid |
| 97180990 | Naproxen 250mg gastro-resistant tablets | strong_nsaid |
| 97550988 | Naproxen 250mg gastro-resistant tablets | strong_nsaid |
| 98674988 | Naproxen 250mg gastro-resistant tablets | strong_nsaid |
| 38698978 | Naproxen 250mg tablets | strong_nsaid |
| 42595978 | Naproxen 250mg tablets | strong_nsaid |
| 52437979 | Naproxen 250mg tablets | strong_nsaid |
| 91751979 | Naproxen 250mg tablets | strong_nsaid |
| 91752979 | Naproxen 250mg tablets | strong_nsaid |
| 91757979 | Naproxen 250mg tablets | strong_nsaid |
| 92638990 | Naproxen 250mg tablets | strong_nsaid |
| 92950997 | Naproxen 250mg tablets | strong_nsaid |
| 96451998 | Naproxen 250mg tablets | strong_nsaid |
| 96452998 | Naproxen 250mg tablets | strong_nsaid |
| 97712998 | Naproxen 250mg tablets | strong_nsaid |
| 97228998 | Naproxen 250mg tablets | strong_nsaid |
| 97566998 | Naproxen 250mg tablets | strong_nsaid |
| 97657998 | Naproxen 250mg tablets | strong_nsaid |
| 97734990 | Naproxen 250mg tablets | strong_nsaid |
| 97833998 | Naproxen 250mg tablets | strong_nsaid |
| 98674990 | Naproxen 250mg tablets | strong_nsaid |
| 99728990 | Naproxen 250mg tablets | strong_nsaid |
| 99730990 | Naproxen 250mg tablets | strong_nsaid |
| 99731990 | Naproxen 250mg tablets | strong_nsaid |
| 24128978 | Naproxen 250mg/5ml oral suspension | strong_nsaid |
| 24803978 | Naproxen 250mg/5ml oral suspension | strong_nsaid |
| 26231978 | Naproxen 250mg/5ml oral suspension | strong_nsaid |
| 79451979 | Naproxen 250mg/5ml oral suspension | strong_nsaid |
| 93169996 | Naproxen 375mg gastro-resistant tablets | strong_nsaid |
| 95093997 | Naproxen 375mg gastro-resistant tablets | strong_nsaid |
| 96625989 | Naproxen 375mg gastro-resistant tablets | strong_nsaid |
| 97658998 | Naproxen 375mg modified release tablets | strong_nsaid |
| 93135997 | Naproxen 375mg modified-release tablet | strong_nsaid |
| 97228996 | Naproxen 375mg tablet | strong_nsaid |
| 93267998 | Naproxen 375mg tablets | strong_nsaid |
| 81744998 | Naproxen 500mg / Esomeprazole 20mg modified-release tablets | strong_nsaid |
| 81745998 | Naproxen 500mg / Esomeprazole 20mg modified-release tablets | strong_nsaid |
| 88138998 | Naproxen 500mg gastro-resistant tablets | strong_nsaid |
| 91733979 | Naproxen 500mg gastro-resistant tablets | strong_nsaid |
| 92738990 | Naproxen 500mg gastro-resistant tablets | strong_nsaid |
| 92950996 | Naproxen 500mg gastro-resistant tablets | strong_nsaid |
| 93169997 | Naproxen 500mg gastro-resistant tablets | strong_nsaid |
| 93170997 | Naproxen 500mg gastro-resistant tablets | strong_nsaid |
| 95093996 | Naproxen 500mg gastro-resistant tablets | strong_nsaid |
| 96625988 | Naproxen 500mg gastro-resistant tablets | strong_nsaid |
| 97100989 | Naproxen 500mg gastro-resistant tablets | strong_nsaid |
| 97180989 | Naproxen 500mg gastro-resistant tablets | strong_nsaid |
| 97187989 | Naproxen 500mg gastro-resistant tablets | strong_nsaid |
| 97700990 | Naproxen 500mg gastro-resistant tablets | strong_nsaid |
| 99728988 | Naproxen 500mg gastro-resistant tablets | strong_nsaid |
| 93135998 | Naproxen 500mg granules | strong_nsaid |
| 93029998 | Naproxen 500mg modified release tablets | strong_nsaid |
| 97658997 | Naproxen 500mg modified release tablets | strong_nsaid |
| 93135996 | Naproxen 500mg modified-release tablets | strong_nsaid |
| 95754998 | Naproxen 500mg suppositories | strong_nsaid |
| 97566996 | Naproxen 500mg suppositories | strong_nsaid |
| 91746979 | Naproxen 500mg tablets | strong_nsaid |
| 92950998 | Naproxen 500mg tablets | strong_nsaid |
| 96108990 | Naproxen 500mg tablets | strong_nsaid |
| 96451997 | Naproxen 500mg tablets | strong_nsaid |
| 96452997 | Naproxen 500mg tablets | strong_nsaid |
| 97712997 | Naproxen 500mg tablets | strong_nsaid |
| 97228997 | Naproxen 500mg tablets | strong_nsaid |
| 97565998 | Naproxen 500mg tablets | strong_nsaid |
| 97657997 | Naproxen 500mg tablets | strong_nsaid |
| 97833997 | Naproxen 500mg tablets | strong_nsaid |
| 98674989 | Naproxen 500mg tablets | strong_nsaid |
| 99728989 | Naproxen 500mg tablets | strong_nsaid |
| 99730989 | Naproxen 500mg tablets | strong_nsaid |
| 99731989 | Naproxen 500mg tablets | strong_nsaid |
| 95312998 | Naproxen 500mg tablets and misoprostol 200microgram tablets | strong_nsaid |
| 79449979 | Naproxen 500mg/5ml oral suspension | strong_nsaid |
| 98907998 | Naproxen 500mg/sachet granules | strong_nsaid |
| 68279979 | Naproxen 75mg/5ml oral suspension | strong_nsaid |
| 88133998 | Naproxen and misoprostol 500mgwith200microgram combined tablet | strong_nsaid |
| 85272998 | Naproxen oral solution | strong_nsaid |
| 95753998 | Naproxen sodium 275mg tablets | strong_nsaid |
| 97564998 | Naproxen sodium 275mg tablets | strong_nsaid |
| 83122998 | Oxycodone 10mg / Naloxone 5mg modified-release tablets | strong_opioid |
| 83124998 | Oxycodone 10mg / Naloxone 5mg modified-release tablets | strong_opioid |
| 78212978 | Oxycodone 10mg capsules | strong_opioid |
| 83284978 | Oxycodone 10mg capsules | strong_opioid |
| 90957997 | Oxycodone 10mg capsules | strong_opioid |
| 93455998 | Oxycodone 10mg capsules | strong_opioid |
| 95445979 | Oxycodone 10mg capsules | strong_opioid |
| 95447979 | Oxycodone 10mg capsules | strong_opioid |
| 19152978 | Oxycodone 10mg modified-release tablets | strong_opioid |
| 24877978 | Oxycodone 10mg modified-release tablets | strong_opioid |
| 37719978 | Oxycodone 10mg modified-release tablets | strong_opioid |
| 42684978 | Oxycodone 10mg modified-release tablets | strong_opioid |
| 50925978 | Oxycodone 10mg modified-release tablets | strong_opioid |
| 56161979 | Oxycodone 10mg modified-release tablets | strong_opioid |
| 57815979 | Oxycodone 10mg modified-release tablets | strong_opioid |
| 74514978 | Oxycodone 10mg modified-release tablets | strong_opioid |
| 91424998 | Oxycodone 10mg modified-release tablets | strong_opioid |
| 92305998 | Oxycodone 10mg modified-release tablets | strong_opioid |
| 95435979 | Oxycodone 10mg modified-release tablets | strong_opioid |
| 95436979 | Oxycodone 10mg modified-release tablets | strong_opioid |
| 95437979 | Oxycodone 10mg modified-release tablets | strong_opioid |
| 95439979 | Oxycodone 10mg modified-release tablets | strong_opioid |
| 26106978 | Oxycodone 10mg/1ml solution for injection ampoules | strong_opioid |
| 85359998 | Oxycodone 10mg/1ml solution for injection ampoules | strong_opioid |
| 85361998 | Oxycodone 10mg/1ml solution for injection ampoules | strong_opioid |
| 89250979 | Oxycodone 10mg/1ml solution for injection ampoules | strong_opioid |
| 89251979 | Oxycodone 10mg/1ml solution for injection ampoules | strong_opioid |
| 26126978 | Oxycodone 10mg/ml oral solution sugar free | strong_opioid |
| 90956997 | Oxycodone 10mg/ml oral solution sugar free | strong_opioid |
| 92306996 | Oxycodone 10mg/ml oral solution sugar free | strong_opioid |
| 46962978 | Oxycodone 120mg modified-release tablets | strong_opioid |
| 81565998 | Oxycodone 120mg modified-release tablets | strong_opioid |
| 81566998 | Oxycodone 120mg modified-release tablets | strong_opioid |
| 42509978 | Oxycodone 15mg modified-release tablets | strong_opioid |
| 47111978 | Oxycodone 15mg modified-release tablets | strong_opioid |
| 50882978 | Oxycodone 15mg modified-release tablets | strong_opioid |
| 59238978 | Oxycodone 15mg modified-release tablets | strong_opioid |
| 81550998 | Oxycodone 15mg modified-release tablets | strong_opioid |
| 81553998 | Oxycodone 15mg modified-release tablets | strong_opioid |
| 84265978 | Oxycodone 15mg modified-release tablets | strong_opioid |
| 53906979 | Oxycodone 20mg / Naloxone 10mg modified-release tablets | strong_opioid |
| 83121998 | Oxycodone 20mg / Naloxone 10mg modified-release tablets | strong_opioid |
| 83123998 | Oxycodone 20mg / Naloxone 10mg modified-release tablets | strong_opioid |
| 78211978 | Oxycodone 20mg capsules | strong_opioid |
| 83283978 | Oxycodone 20mg capsules | strong_opioid |
| 90957996 | Oxycodone 20mg capsules | strong_opioid |
| 93455997 | Oxycodone 20mg capsules | strong_opioid |
| 95440979 | Oxycodone 20mg capsules | strong_opioid |
| 42683978 | Oxycodone 20mg modified-release tablets | strong_opioid |
| 50924978 | Oxycodone 20mg modified-release tablets | strong_opioid |
| 56160979 | Oxycodone 20mg modified-release tablets | strong_opioid |
| 57814979 | Oxycodone 20mg modified-release tablets | strong_opioid |
| 74511978 | Oxycodone 20mg modified-release tablets | strong_opioid |
| 91424997 | Oxycodone 20mg modified-release tablets | strong_opioid |
| 92305997 | Oxycodone 20mg modified-release tablets | strong_opioid |
| 95430979 | Oxycodone 20mg modified-release tablets | strong_opioid |
| 95431979 | Oxycodone 20mg modified-release tablets | strong_opioid |
| 95432979 | Oxycodone 20mg modified-release tablets | strong_opioid |
| 95434979 | Oxycodone 20mg modified-release tablets | strong_opioid |
| 26107978 | Oxycodone 20mg/2ml solution for injection ampoules | strong_opioid |
| 85358998 | Oxycodone 20mg/2ml solution for injection ampoules | strong_opioid |
| 85360998 | Oxycodone 20mg/2ml solution for injection ampoules | strong_opioid |
| 89254979 | Oxycodone 20mg/2ml solution for injection ampoules | strong_opioid |
| 89350998 | Oxycodone 20mg/2ml solution for injection ampoules | strong_opioid |
| 89351998 | Oxycodone 20mg/2ml solution for injection ampoules | strong_opioid |
| 42510978 | Oxycodone 30mg modified-release tablets | strong_opioid |
| 47112978 | Oxycodone 30mg modified-release tablets | strong_opioid |
| 50883978 | Oxycodone 30mg modified-release tablets | strong_opioid |
| 59239978 | Oxycodone 30mg modified-release tablets | strong_opioid |
| 81549998 | Oxycodone 30mg modified-release tablets | strong_opioid |
| 81552998 | Oxycodone 30mg modified-release tablets | strong_opioid |
| 84266978 | Oxycodone 30mg modified-release tablets | strong_opioid |
| 82725998 | Oxycodone 40mg / Naloxone 20mg modified-release tablets | strong_opioid |
| 82727998 | Oxycodone 40mg / Naloxone 20mg modified-release tablets | strong_opioid |
| 24875978 | Oxycodone 40mg modified-release tablets | strong_opioid |
| 42682978 | Oxycodone 40mg modified-release tablets | strong_opioid |
| 50923978 | Oxycodone 40mg modified-release tablets | strong_opioid |
| 56159979 | Oxycodone 40mg modified-release tablets | strong_opioid |
| 57813979 | Oxycodone 40mg modified-release tablets | strong_opioid |
| 74508978 | Oxycodone 40mg modified-release tablets | strong_opioid |
| 91424996 | Oxycodone 40mg modified-release tablets | strong_opioid |
| 92305996 | Oxycodone 40mg modified-release tablets | strong_opioid |
| 26008978 | Oxycodone 50mg/1ml solution for injection ampoules | strong_opioid |
| 78774978 | Oxycodone 50mg/1ml solution for injection ampoules | strong_opioid |
| 82796998 | Oxycodone 50mg/1ml solution for injection ampoules | strong_opioid |
| 82797998 | Oxycodone 50mg/1ml solution for injection ampoules | strong_opioid |
| 82726998 | Oxycodone 5mg / Naloxone 2.5mg modified-release tablets | strong_opioid |
| 82728998 | Oxycodone 5mg / Naloxone 2.5mg modified-release tablets | strong_opioid |
| 78213978 | Oxycodone 5mg capsules | strong_opioid |
| 83285978 | Oxycodone 5mg capsules | strong_opioid |
| 90956996 | Oxycodone 5mg capsules | strong_opioid |
| 90957998 | Oxycodone 5mg capsules | strong_opioid |
| 95448979 | Oxycodone 5mg capsules | strong_opioid |
| 95450979 | Oxycodone 5mg capsules | strong_opioid |
| 95451979 | Oxycodone 5mg capsules | strong_opioid |
| 42544978 | Oxycodone 5mg modified-release tablets | strong_opioid |
| 50891978 | Oxycodone 5mg modified-release tablets | strong_opioid |
| 56116979 | Oxycodone 5mg modified-release tablets | strong_opioid |
| 57755979 | Oxycodone 5mg modified-release tablets | strong_opioid |
| 74447978 | Oxycodone 5mg modified-release tablets | strong_opioid |
| 89372979 | Oxycodone 5mg modified-release tablets | strong_opioid |
| 89373979 | Oxycodone 5mg modified-release tablets | strong_opioid |
| 89374979 | Oxycodone 5mg modified-release tablets | strong_opioid |
| 93448998 | Oxycodone 5mg modified-release tablets | strong_opioid |
| 93450998 | Oxycodone 5mg modified-release tablets | strong_opioid |
| 26127978 | Oxycodone 5mg/5ml oral solution sugar free | strong_opioid |
| 42686978 | Oxycodone 5mg/5ml oral solution sugar free | strong_opioid |
| 84334998 | Oxycodone 5mg/5ml oral solution sugar free | strong_opioid |
| 90956998 | Oxycodone 5mg/5ml oral solution sugar free | strong_opioid |
| 92306997 | Oxycodone 5mg/5ml oral solution sugar free | strong_opioid |
| 91804990 | Oxycodone 5mg/5ml oral solution sugar free | strong_opioid |
| 95454979 | Oxycodone 5mg/5ml oral solution sugar free | strong_opioid |
| 42508978 | Oxycodone 60mg modified-release tablets | strong_opioid |
| 47110978 | Oxycodone 60mg modified-release tablets | strong_opioid |
| 59237978 | Oxycodone 60mg modified-release tablets | strong_opioid |
| 81548998 | Oxycodone 60mg modified-release tablets | strong_opioid |
| 81551998 | Oxycodone 60mg modified-release tablets | strong_opioid |
| 42681978 | Oxycodone 80mg modified-release tablets | strong_opioid |
| 56158979 | Oxycodone 80mg modified-release tablets | strong_opioid |
| 57812979 | Oxycodone 80mg modified-release tablets | strong_opioid |
| 74503978 | Oxycodone 80mg modified-release tablets | strong_opioid |
| 74505978 | Oxycodone 80mg modified-release tablets | strong_opioid |
| 92304998 | Oxycodone 80mg modified-release tablets | strong_opioid |
| 92306998 | Oxycodone 80mg modified-release tablets | strong_opioid |
| 95422979 | Oxycodone 80mg modified-release tablets | strong_opioid |
| 95423979 | Oxycodone 80mg modified-release tablets | strong_opioid |
| 93910998 | Oxycodone hydrochloride 30mg suppositories | strong_opioid |
| 90729998 | Paracetamo & codeine phosphate 1000mg+60mg/sachet powder | weak_opioid |
| 89015998 | Paracetamol & codeine phosphate 500mg+30mg/sachet powder | weak_opioid |
| 89015998 | Paracetamol & codeine phosphate 500mg+30mg/sachet powder | weak_opioid |
| 86540998 | Paracetamol & dihydrocodeine tartrate 500mg+7.46mg effervescent tablets | weak_opioid |
| 86540998 | Paracetamol & dihydrocodeine tartrate 500mg+7.46mg effervescent tablets | weak_opioid |
| 88777998 | Paracetamol & domperidone 500mg+10mg tablets | paracetamol |
| 90545998 | Paracetamol & methionine 500mg+100mg tablets | paracetamol |
| 97243998 | Paracetamol & methionine 500mg+250mg tablets | paracetamol |
| 97862992 | Paracetamol 1 gm sup | paracetamol |
| 93403992 | Paracetamol 1 gm tab | paracetamol |
| 97859992 | Paracetamol 100 mg sup | paracetamol |
| 89827996 | Paracetamol 1000mg & ascorbic acid 77mg powder | paracetamol |
| 91246998 | Paracetamol 1000mg with codeine phosphate 60mg effervescent powder sugar free | weak_opioid |
| 91246998 | Paracetamol 1000mg with codeine phosphate 60mg effervescent powder sugar free | weak_opioid |
| 21083978 | Paracetamol 100mg/10ml solution for infusion ampoules | paracetamol |
| 92653998 | Paracetamol 100mg/ml oral solution | paracetamol |
| 92654998 | Paracetamol 100mg/ml oral solution sugar free | paracetamol |
| 90120998 | Paracetamol 120 mg capsule | paracetamol |
| 97741997 | Paracetamol 120mg soluble tablets | paracetamol |
| 37645978 | Paracetamol 120mg soluble tablets sugar free | paracetamol |
| 97744998 | Paracetamol 120mg soluble tablets sugar free | paracetamol |
| 93473998 | Paracetamol 120mg suppositories | paracetamol |
| 95765979 | Paracetamol 120mg suppositories | paracetamol |
| 97016990 | Paracetamol 120mg suppositories | paracetamol |
| 97970988 | Paracetamol 120mg suppositories | paracetamol |
| 98437990 | Paracetamol 120mg suppositories | paracetamol |
| 90262998 | Paracetamol 120mg/5ml oral solution | paracetamol |
| 97197998 | Paracetamol 120mg/5ml oral solution | paracetamol |
| 90928998 | Paracetamol 120mg/5ml oral solution paediatric sugar free | paracetamol |
| 95669990 | Paracetamol 120mg/5ml oral solution paediatric sugar free | paracetamol |
| 95741979 | Paracetamol 120mg/5ml oral solution paediatric sugar free | paracetamol |
| 95743979 | Paracetamol 120mg/5ml oral solution paediatric sugar free | paracetamol |
| 95745979 | Paracetamol 120mg/5ml oral solution paediatric sugar free | paracetamol |
| 95746979 | Paracetamol 120mg/5ml oral solution paediatric sugar free | paracetamol |
| 95776979 | Paracetamol 120mg/5ml oral solution paediatric sugar free | paracetamol |
| 95777979 | Paracetamol 120mg/5ml oral solution paediatric sugar free | paracetamol |
| 97226997 | Paracetamol 120mg/5ml oral solution paediatric sugar free | paracetamol |
| 97868990 | Paracetamol 120mg/5ml oral solution paediatric sugar free | paracetamol |
| 98661990 | Paracetamol 120mg/5ml oral solution paediatric sugar free | paracetamol |
| 98662990 | Paracetamol 120mg/5ml oral solution paediatric sugar free | paracetamol |
| 98741989 | Paracetamol 120mg/5ml oral solution paediatric sugar free | paracetamol |
| 99141990 | Paracetamol 120mg/5ml oral solution paediatric sugar free | paracetamol |
| 99475989 | Paracetamol 120mg/5ml oral solution paediatric sugar free | paracetamol |
| 99477990 | Paracetamol 120mg/5ml oral solution paediatric sugar free | paracetamol |
| 92927997 | Paracetamol 120mg/5ml oral suspension | paracetamol |
| 78805979 | Paracetamol 120mg/5ml oral suspension 5ml sachets | paracetamol |
| 99863998 | Paracetamol 120mg/5ml oral suspension 5ml sachets | paracetamol |
| 78806979 | Paracetamol 120mg/5ml oral suspension 5ml sachets sugar free | paracetamol |
| 86649979 | Paracetamol 120mg/5ml oral suspension 5ml sachets sugar free | paracetamol |
| 86652979 | Paracetamol 120mg/5ml oral suspension 5ml sachets sugar free | paracetamol |
| 89416998 | Paracetamol 120mg/5ml oral suspension 5ml sachets sugar free | paracetamol |
| 95733979 | Paracetamol 120mg/5ml oral suspension 5ml sachets sugar free | paracetamol |
| 97396998 | Paracetamol 120mg/5ml oral suspension 5ml sachets sugar free | paracetamol |
| 97741998 | Paracetamol 120mg/5ml oral suspension 5ml sachets sugar free | paracetamol |
| 97744997 | Paracetamol 120mg/5ml oral suspension 5ml sachets sugar free | paracetamol |
| 52675979 | Paracetamol 120mg/5ml oral suspension paediatric | paracetamol |
| 95761979 | Paracetamol 120mg/5ml oral suspension paediatric | paracetamol |
| 95762979 | Paracetamol 120mg/5ml oral suspension paediatric | paracetamol |
| 95178990 | Paracetamol 120mg/5ml oral suspension paediatric | paracetamol |
| 95456998 | Paracetamol 120mg/5ml oral suspension paediatric | paracetamol |
| 96363990 | Paracetamol 120mg/5ml oral suspension paediatric | paracetamol |
| 98065990 | Paracetamol 120mg/5ml oral suspension paediatric | paracetamol |
| 99141988 | Paracetamol 120mg/5ml oral suspension paediatric | paracetamol |
| 37724978 | Paracetamol 120mg/5ml oral suspension paediatric sugar free | paracetamol |
| 56163979 | Paracetamol 120mg/5ml oral suspension paediatric sugar free | paracetamol |
| 68657978 | Paracetamol 120mg/5ml oral suspension paediatric sugar free | paracetamol |
| 81615998 | Paracetamol 120mg/5ml oral suspension paediatric sugar free | paracetamol |
| 81794998 | Paracetamol 120mg/5ml oral suspension paediatric sugar free | paracetamol |
| 83442998 | Paracetamol 120mg/5ml oral suspension paediatric sugar free | paracetamol |
| 83443998 | Paracetamol 120mg/5ml oral suspension paediatric sugar free | paracetamol |
| 92412990 | Paracetamol 120mg/5ml oral suspension paediatric sugar free | paracetamol |
| 92927998 | Paracetamol 120mg/5ml oral suspension paediatric sugar free | paracetamol |
| 93480996 | Paracetamol 120mg/5ml oral suspension paediatric sugar free | paracetamol |
| 93480998 | Paracetamol 120mg/5ml oral suspension paediatric sugar free | paracetamol |
| 94521998 | Paracetamol 120mg/5ml oral suspension paediatric sugar free | paracetamol |
| 95760979 | Paracetamol 120mg/5ml oral suspension paediatric sugar free | paracetamol |
| 94004990 | Paracetamol 120mg/5ml oral suspension paediatric sugar free | paracetamol |
| 94904990 | Paracetamol 120mg/5ml oral suspension paediatric sugar free | paracetamol |
| 95731979 | Paracetamol 120mg/5ml oral suspension paediatric sugar free | paracetamol |
| 95735979 | Paracetamol 120mg/5ml oral suspension paediatric sugar free | paracetamol |
| 95736979 | Paracetamol 120mg/5ml oral suspension paediatric sugar free | paracetamol |
| 95737979 | Paracetamol 120mg/5ml oral suspension paediatric sugar free | paracetamol |
| 95738979 | Paracetamol 120mg/5ml oral suspension paediatric sugar free | paracetamol |
| 95740979 | Paracetamol 120mg/5ml oral suspension paediatric sugar free | paracetamol |
| 96614989 | Paracetamol 120mg/5ml oral suspension paediatric sugar free | paracetamol |
| 97461998 | Paracetamol 120mg/5ml oral suspension paediatric sugar free | paracetamol |
| 97698990 | Paracetamol 120mg/5ml oral suspension paediatric sugar free | paracetamol |
| 97946990 | Paracetamol 120mg/5ml oral suspension paediatric sugar free | paracetamol |
| 99863997 | Paracetamol 120mg/5ml oral suspension paediatric sugar free | paracetamol |
| 88152998 | Paracetamol 120mg/5ml oral suspension sugar free | paracetamol |
| 92875998 | Paracetamol 120mg/5ml oral suspension sugar free | paracetamol |
| 94776998 | Paracetamol 120mg/5ml oral suspension sugar free | paracetamol |
| 98468998 | Paracetamol 120mg/5ml oral suspension sugar free | paracetamol |
| 97679998 | Paracetamol 120mg/5ml sugar free suspension | paracetamol |
| 94268992 | Paracetamol 125 mg eli | paracetamol |
| 94267992 | Paracetamol 125 mg mix | paracetamol |
| 95382992 | Paracetamol 125 mg tab | paracetamol |
| 93473997 | Paracetamol 125mg suppositories | paracetamol |
| 97238998 | Paracetamol 125mg suppositories | paracetamol |
| 90120997 | Paracetamol 125mg/5ml syrup | paracetamol |
| 96379992 | Paracetamol 150 mg sup | paracetamol |
| 14527978 | Paracetamol 1g effervescent tablets sugar free | paracetamol |
| 85827979 | Paracetamol 1g oral powder sachets | paracetamol |
| 90120996 | Paracetamol 1g oral powder sachets | paracetamol |
| 79018979 | Paracetamol 1g suppositories | paracetamol |
| 82408998 | Paracetamol 1g tablets | paracetamol |
| 82409998 | Paracetamol 1g tablets | paracetamol |
| 24037978 | Paracetamol 1g/100ml solution for infusion bottles | paracetamol |
| 87524998 | Paracetamol 1g/100ml solution for infusion bottles | paracetamol |
| 87523998 | Paracetamol 1g/100ml solution for infusion vials | paracetamol |
| 66547979 | Paracetamol 1g/5ml oral solution | paracetamol |
| 66545979 | Paracetamol 1g/5ml oral suspension | paracetamol |
| 96731992 | Paracetamol 20 mg sup | paracetamol |
| 95379992 | Paracetamol 240 mg eli | paracetamol |
| 95381992 | Paracetamol 240 mg sus | paracetamol |
| 97226996 | Paracetamol 240mg oral powder sachets sugar free | paracetamol |
| 90123998 | Paracetamol 240mg suppositories | paracetamol |
| 97970989 | Paracetamol 240mg suppositories | paracetamol |
| 94266992 | Paracetamol 250 mg tab | paracetamol |
| 88199998 | Paracetamol 250mg orodispersible tablets sugar free | paracetamol |
| 90341998 | Paracetamol 250mg orodispersible tablets sugar free | paracetamol |
| 90123996 | Paracetamol 250mg suppositories | paracetamol |
| 97238996 | Paracetamol 250mg suppositories | paracetamol |
| 95454998 | Paracetamol 250mg with aspirin 250mg & codeine 6.8mg tablet | weak_opioid |
| 95454998 | Paracetamol 250mg with aspirin 250mg & codeine 6.8mg tablet | weak_opioid |
| 66542979 | Paracetamol 250mg/5ml oral solution | paracetamol |
| 95179990 | Paracetamol 250mg/5ml oral solution | paracetamol |
| 95468997 | Paracetamol 250mg/5ml oral solution | paracetamol |
| 95775979 | Paracetamol 250mg/5ml oral solution | paracetamol |
| 96363989 | Paracetamol 250mg/5ml oral solution | paracetamol |
| 96872990 | Paracetamol 250mg/5ml oral solution | paracetamol |
| 99475990 | Paracetamol 250mg/5ml oral solution | paracetamol |
| 92496998 | Paracetamol 250mg/5ml oral suspension | paracetamol |
| 98235997 | Paracetamol 250mg/5ml oral suspension | paracetamol |
| 98235998 | Paracetamol 250mg/5ml oral suspension | paracetamol |
| 85256979 | Paracetamol 250mg/5ml oral suspension 5ml sachets sugar free | paracetamol |
| 85257979 | Paracetamol 250mg/5ml oral suspension 5ml sachets sugar free | paracetamol |
| 66543979 | Paracetamol 250mg/5ml oral suspension sugar free | paracetamol |
| 83444998 | Paracetamol 250mg/5ml oral suspension sugar free | paracetamol |
| 93480997 | Paracetamol 250mg/5ml oral suspension sugar free | paracetamol |
| 94521997 | Paracetamol 250mg/5ml oral suspension sugar free | paracetamol |
| 95722979 | Paracetamol 250mg/5ml oral suspension sugar free | paracetamol |
| 95723979 | Paracetamol 250mg/5ml oral suspension sugar free | paracetamol |
| 95725979 | Paracetamol 250mg/5ml oral suspension sugar free | paracetamol |
| 95726979 | Paracetamol 250mg/5ml oral suspension sugar free | paracetamol |
| 95727979 | Paracetamol 250mg/5ml oral suspension sugar free | paracetamol |
| 95728979 | Paracetamol 250mg/5ml oral suspension sugar free | paracetamol |
| 96614990 | Paracetamol 250mg/5ml oral suspension sugar free | paracetamol |
| 96872989 | Paracetamol 250mg/5ml oral suspension sugar free | paracetamol |
| 97075992 | Paracetamol 250mg/5ml oral suspension sugar free | paracetamol |
| 97698989 | Paracetamol 250mg/5ml oral suspension sugar free | paracetamol |
| 97744996 | Paracetamol 250mg/5ml oral suspension sugar free | paracetamol |
| 98020989 | Paracetamol 250mg/5ml oral suspension sugar free | paracetamol |
| 98204998 | Paracetamol 250mg/5ml oral suspension sugar free | paracetamol |
| 97863992 | Paracetamol 30 mg sup | paracetamol |
| 95380992 | Paracetamol 300 mg tab | paracetamol |
| 91258998 | Paracetamol 325mg / Isometheptene 65mg capsules | paracetamol |
| 94197998 | Paracetamol 325mg / Isometheptene 65mg capsules | paracetamol |
| 96100998 | Paracetamol 325mg / Isometheptene 65mg capsules | paracetamol |
| 96401998 | Paracetamol 325mg / Isometheptene 65mg capsules | paracetamol |
| 89827998 | Paracetamol 350mg & ascorbic acid 50mg powder | paracetamol |
| 90508996 | Paracetamol 450mg with codeine phosphate 8.1mg tablet | weak_opioid |
| 90508996 | Paracetamol 450mg with codeine phosphate 8.1mg tablet | weak_opioid |
| 96732992 | PARACETAMOL 450MG/CODEINE 8.1MG TAB | weak_opioid |
| 96732992 | PARACETAMOL 450MG/CODEINE 8.1MG TAB | weak_opioid |
| 94034992 | Paracetamol 500 mg eli | paracetamol |
| 86541998 | Paracetamol 500mg / dihydrocodeine 7.46mg effervescent tablets sugar free | weak_opioid |
| 86541998 | Paracetamol 500mg / dihydrocodeine 7.46mg effervescent tablets sugar free | weak_opioid |
| 95458997 | Paracetamol 500mg / dihydrocodeine 7.46mg tablets | weak_opioid |
| 95458997 | Paracetamol 500mg / dihydrocodeine 7.46mg tablets | weak_opioid |
| 88778998 | Paracetamol 500mg / domperidone 10mg tablets | paracetamol |
| 28967978 | Paracetamol 500mg / ibuprofen 150mg tablets | weak_nsaid |
| 28967978 | Paracetamol 500mg / ibuprofen 150mg tablets | weak_nsaid |
| 81276998 | Paracetamol 500mg / Ibuprofen 200mg tablets | weak_nsaid |
| 81279998 | Paracetamol 500mg / Ibuprofen 200mg tablets | weak_nsaid |
| 81276998 | Paracetamol 500mg / Ibuprofen 200mg tablets | weak_nsaid |
| 81279998 | Paracetamol 500mg / Ibuprofen 200mg tablets | weak_nsaid |
| 94924997 | Paracetamol 500mg / Metoclopramide 5mg effervescent powder sachets sugar free | paracetamol |
| 95832997 | Paracetamol 500mg / Metoclopramide 5mg effervescent powder sachets sugar free | paracetamol |
| 98358997 | Paracetamol 500mg / Metoclopramide 5mg effervescent powder sachets sugar free | paracetamol |
| 60693979 | Paracetamol 500mg / Metoclopramide 5mg tablets | paracetamol |
| 94924998 | Paracetamol 500mg / Metoclopramide 5mg tablets | paracetamol |
| 95832998 | Paracetamol 500mg / Metoclopramide 5mg tablets | paracetamol |
| 98358998 | Paracetamol 500mg / Metoclopramide 5mg tablets | paracetamol |
| 52674979 | Paracetamol 500mg capsules | paracetamol |
| 61467979 | Paracetamol 500mg capsules | paracetamol |
| 90264998 | Paracetamol 500mg capsules | paracetamol |
| 92269990 | Paracetamol 500mg capsules | paracetamol |
| 92701998 | Paracetamol 500mg capsules | paracetamol |
| 92995990 | Paracetamol 500mg capsules | paracetamol |
| 93410990 | Paracetamol 500mg capsules | paracetamol |
| 93556990 | Paracetamol 500mg capsules | paracetamol |
| 94487990 | Paracetamol 500mg capsules | paracetamol |
| 94686990 | Paracetamol 500mg capsules | paracetamol |
| 95468996 | Paracetamol 500mg capsules | paracetamol |
| 95577990 | Paracetamol 500mg capsules | paracetamol |
| 95747979 | Paracetamol 500mg capsules | paracetamol |
| 95750979 | Paracetamol 500mg capsules | paracetamol |
| 95753979 | Paracetamol 500mg capsules | paracetamol |
| 95755979 | Paracetamol 500mg capsules | paracetamol |
| 95756979 | Paracetamol 500mg capsules | paracetamol |
| 98020990 | Paracetamol 500mg capsules | paracetamol |
| 98469997 | Paracetamol 500mg capsules | paracetamol |
| 98508989 | Paracetamol 500mg capsules | paracetamol |
| 99476988 | Paracetamol 500mg capsules | paracetamol |
| 21286978 | Paracetamol 500mg effervescent tablets | paracetamol |
| 52678979 | Paracetamol 500mg effervescent tablets | paracetamol |
| 93945992 | Paracetamol 500mg effervescent tablets | paracetamol |
| 94488990 | Paracetamol 500mg effervescent tablets | paracetamol |
| 95030990 | Paracetamol 500mg effervescent tablets | paracetamol |
| 95440990 | Paracetamol 500mg effervescent tablets | paracetamol |
| 95468998 | Paracetamol 500mg effervescent tablets | paracetamol |
| 95576990 | Paracetamol 500mg effervescent tablets | paracetamol |
| 95608990 | Paracetamol 500mg effervescent tablets | paracetamol |
| 97858992 | Paracetamol 500mg effervescent tablets | paracetamol |
| 99476989 | Paracetamol 500mg effervescent tablets | paracetamol |
| 82980998 | Paracetamol 500mg soluble tablets | paracetamol |
| 90118998 | Paracetamol 500mg soluble tablets | paracetamol |
| 93891990 | Paracetamol 500mg soluble tablets | paracetamol |
| 95779979 | Paracetamol 500mg soluble tablets | paracetamol |
| 95780979 | Paracetamol 500mg soluble tablets | paracetamol |
| 98467998 | Paracetamol 500mg soluble tablets | paracetamol |
| 93473996 | Paracetamol 500mg suppositories | paracetamol |
| 97016988 | Paracetamol 500mg suppositories | paracetamol |
| 97860992 | Paracetamol 500mg suppositories | paracetamol |
| 97970990 | Paracetamol 500mg suppositories | paracetamol |
| 20228978 | Paracetamol 500mg tablets | paracetamol |
| 30021978 | Paracetamol 500mg tablets | paracetamol |
| 42691978 | Paracetamol 500mg tablets | paracetamol |
| 47264978 | Paracetamol 500mg tablets | paracetamol |
| 51975978 | Paracetamol 500mg tablets | paracetamol |
| 52680979 | Paracetamol 500mg tablets | paracetamol |
| 70311978 | Paracetamol 500mg tablets | paracetamol |
| 72726978 | Paracetamol 500mg tablets | paracetamol |
| 73253978 | Paracetamol 500mg tablets | paracetamol |
| 76285978 | Paracetamol 500mg tablets | paracetamol |
| 76286978 | Paracetamol 500mg tablets | paracetamol |
| 79802978 | Paracetamol 500mg tablets | paracetamol |
| 82578978 | Paracetamol 500mg tablets | paracetamol |
| 83324998 | Paracetamol 500mg tablets | paracetamol |
| 84357978 | Paracetamol 500mg tablets | paracetamol |
| 89193998 | Paracetamol 500mg tablets | paracetamol |
| 89948998 | Paracetamol 500mg tablets | paracetamol |
| 90264997 | Paracetamol 500mg tablets | paracetamol |
| 90290998 | Paracetamol 500mg tablets | paracetamol |
| 91585998 | Paracetamol 500mg tablets | paracetamol |
| 92042990 | Paracetamol 500mg tablets | paracetamol |
| 93925990 | Paracetamol 500mg tablets | paracetamol |
| 94475990 | Paracetamol 500mg tablets | paracetamol |
| 94689990 | Paracetamol 500mg tablets | paracetamol |
| 95038990 | Paracetamol 500mg tablets | paracetamol |
| 95076990 | Paracetamol 500mg tablets | paracetamol |
| 95104990 | Paracetamol 500mg tablets | paracetamol |
| 95334998 | Paracetamol 500mg tablets | paracetamol |
| 95516990 | Paracetamol 500mg tablets | paracetamol |
| 95782979 | Paracetamol 500mg tablets | paracetamol |
| 95783979 | Paracetamol 500mg tablets | paracetamol |
| 95786979 | Paracetamol 500mg tablets | paracetamol |
| 95788979 | Paracetamol 500mg tablets | paracetamol |
| 95791979 | Paracetamol 500mg tablets | paracetamol |
| 95792979 | Paracetamol 500mg tablets | paracetamol |
| 95793979 | Paracetamol 500mg tablets | paracetamol |
| 95794979 | Paracetamol 500mg tablets | paracetamol |
| 95795979 | Paracetamol 500mg tablets | paracetamol |
| 95796979 | Paracetamol 500mg tablets | paracetamol |
| 96105990 | Paracetamol 500mg tablets | paracetamol |
| 96106990 | Paracetamol 500mg tablets | paracetamol |
| 96204990 | Paracetamol 500mg tablets | paracetamol |
| 96204998 | Paracetamol 500mg tablets | paracetamol |
| 96290990 | Paracetamol 500mg tablets | paracetamol |
| 96417990 | Paracetamol 500mg tablets | paracetamol |
| 96565990 | Paracetamol 500mg tablets | paracetamol |
| 96572990 | Paracetamol 500mg tablets | paracetamol |
| 96940990 | Paracetamol 500mg tablets | paracetamol |
| 97226998 | Paracetamol 500mg tablets | paracetamol |
| 97807998 | Paracetamol 500mg tablets | paracetamol |
| 97936990 | Paracetamol 500mg tablets | paracetamol |
| 97946989 | Paracetamol 500mg tablets | paracetamol |
| 98436990 | Paracetamol 500mg tablets | paracetamol |
| 98469998 | Paracetamol 500mg tablets | paracetamol |
| 98508990 | Paracetamol 500mg tablets | paracetamol |
| 98661989 | Paracetamol 500mg tablets | paracetamol |
| 98741988 | Paracetamol 500mg tablets | paracetamol |
| 99139990 | Paracetamol 500mg tablets | paracetamol |
| 99140989 | Paracetamol 500mg tablets | paracetamol |
| 99141989 | Paracetamol 500mg tablets | paracetamol |
| 99476990 | Paracetamol 500mg tablets | paracetamol |
| 99477989 | Paracetamol 500mg tablets | paracetamol |
| 90508997 | Paracetamol 500mg with codeine phosphate 10mg capsule | weak_opioid |
| 90508997 | Paracetamol 500mg with codeine phosphate 10mg capsule | weak_opioid |
| 90508998 | Paracetamol 500mg with codeine phosphate 10mg tablet | weak_opioid |
| 93330998 | Paracetamol 500mg with codeine phosphate 10mg tablet | weak_opioid |
| 90508998 | Paracetamol 500mg with codeine phosphate 10mg tablet | weak_opioid |
| 93330998 | Paracetamol 500mg with codeine phosphate 10mg tablet | weak_opioid |
| 94768996 | Paracetamol 500mg with codeine phosphate 13.5mg tablet | weak_opioid |
| 94768996 | Paracetamol 500mg with codeine phosphate 13.5mg tablet | weak_opioid |
| 91246997 | Paracetamol 500mg with codeine phosphate 30mg effervescent powder sugar free | weak_opioid |
| 91246997 | Paracetamol 500mg with codeine phosphate 30mg effervescent powder sugar free | weak_opioid |
| 94212997 | Paracetamol 500mg with methionine 100 mg tablet | paracetamol |
| 94212998 | Paracetamol 500mg with methionine 250 mg tablet | paracetamol |
| 89256998 | Paracetamol 500mg with sodium bicarbonate 1342 mg effervescent tablet | paracetamol |
| 86673998 | Paracetamol 500mg/50ml solution for infusion vials | paracetamol |
| 81100998 | Paracetamol 500mg/5ml oral solution | paracetamol |
| 46092978 | Paracetamol 500mg/5ml oral solution sugar free | paracetamol |
| 76407978 | Paracetamol 500mg/5ml oral solution sugar free | paracetamol |
| 77005978 | Paracetamol 500mg/5ml oral solution sugar free | paracetamol |
| 52131979 | Paracetamol 500mg/5ml oral suspension sugar free | paracetamol |
| 53314979 | Paracetamol 500mg/5ml oral suspension sugar free | paracetamol |
| 81080998 | Paracetamol 500mg/5ml oral suspension sugar free | paracetamol |
| 94521996 | Paracetamol 500mg/5ml oral suspension sugar free | paracetamol |
| 99475988 | Paracetamol 500mg/5ml oral suspension sugar free | paracetamol |
| 93296992 | PARACETAMOL 500MG/CODEINE 10MG MG TAB | weak_opioid |
| 93296992 | PARACETAMOL 500MG/CODEINE 10MG MG TAB | weak_opioid |
| 91080998 | Paracetamol 50mg oral powder sachets sugar free | paracetamol |
| 90123997 | Paracetamol 60mg suppositories | paracetamol |
| 97238997 | Paracetamol 60mg suppositories | paracetamol |
| 97861992 | Paracetamol 60mg suppositories | paracetamol |
| 89827997 | Paracetamol 650mg / ascorbic acid 50mg oral powder sachets | paracetamol |
| 90264996 | Paracetamol 650mg / ascorbic acid 50mg oral powder sachets | paracetamol |
| 82487998 | Paracetamol 650mg oral powder sachets | paracetamol |
| 95383992 | Paracetamol 75 mg sus | paracetamol |
| 73293978 | Paracetamol 80mg suppositories | paracetamol |
| 97864992 | Paracetamol co tab | paracetamol |
| 84827998 | Paracetamol oral liquid | paracetamol |
| 98740990 | Paracetamol powder | paracetamol |
| 93553998 | Paracetamol with codeine phosphate peadiatric elixir | weak_opioid |
| 93553998 | Paracetamol with codeine phosphate peadiatric elixir | weak_opioid |
| 86910998 | Paracetamol with dihydrocodeine 500mg +10mg/5ml suspension sugar free | weak_opioid |
| 86910998 | Paracetamol with dihydrocodeine 500mg +10mg/5ml suspension sugar free | weak_opioid |
| 95580998 | Pentazocine 15mg with paracetamol 500mg tablet | paracetamol |
| 28544978 | Pregabalin 100mg capsules | gabapentinoid |
| 51898978 | Pregabalin 100mg capsules | gabapentinoid |
| 51899978 | Pregabalin 100mg capsules | gabapentinoid |
| 55715978 | Pregabalin 100mg capsules | gabapentinoid |
| 87398998 | Pregabalin 100mg capsules | gabapentinoid |
| 87405998 | Pregabalin 100mg capsules | gabapentinoid |
| 89097979 | Pregabalin 100mg capsules | gabapentinoid |
| 8683978 | Pregabalin 100mg tablets | gabapentinoid |
| 28543978 | Pregabalin 150mg capsules | gabapentinoid |
| 46243978 | Pregabalin 150mg capsules | gabapentinoid |
| 47123978 | Pregabalin 150mg capsules | gabapentinoid |
| 51895978 | Pregabalin 150mg capsules | gabapentinoid |
| 51897978 | Pregabalin 150mg capsules | gabapentinoid |
| 55714978 | Pregabalin 150mg capsules | gabapentinoid |
| 87397998 | Pregabalin 150mg capsules | gabapentinoid |
| 87404998 | Pregabalin 150mg capsules | gabapentinoid |
| 89087979 | Pregabalin 150mg capsules | gabapentinoid |
| 89089979 | Pregabalin 150mg capsules | gabapentinoid |
| 89091979 | Pregabalin 150mg capsules | gabapentinoid |
| 8943978 | Pregabalin 150mg tablets | gabapentinoid |
| 64019979 | Pregabalin 150mg/5ml oral solution | gabapentinoid |
| 64017979 | Pregabalin 150mg/5ml oral suspension | gabapentinoid |
| 28542978 | Pregabalin 200mg capsules | gabapentinoid |
| 51892978 | Pregabalin 200mg capsules | gabapentinoid |
| 51894978 | Pregabalin 200mg capsules | gabapentinoid |
| 55713978 | Pregabalin 200mg capsules | gabapentinoid |
| 87396998 | Pregabalin 200mg capsules | gabapentinoid |
| 87403998 | Pregabalin 200mg capsules | gabapentinoid |
| 89079979 | Pregabalin 200mg capsules | gabapentinoid |
| 8941978 | Pregabalin 200mg tablets | gabapentinoid |
| 63818979 | Pregabalin 200mg/5ml oral solution | gabapentinoid |
| 63816979 | Pregabalin 200mg/5ml oral suspension | gabapentinoid |
| 58118979 | Pregabalin 20mg/ml oral solution sugar free | gabapentinoid |
| 58119979 | Pregabalin 20mg/ml oral solution sugar free | gabapentinoid |
| 28537978 | Pregabalin 225mg capsules | gabapentinoid |
| 51877978 | Pregabalin 225mg capsules | gabapentinoid |
| 55708978 | Pregabalin 225mg capsules | gabapentinoid |
| 84233998 | Pregabalin 225mg capsules | gabapentinoid |
| 84234998 | Pregabalin 225mg capsules | gabapentinoid |
| 8933978 | Pregabalin 225mg tablets | gabapentinoid |
| 28541978 | Pregabalin 25mg capsules | gabapentinoid |
| 51889978 | Pregabalin 25mg capsules | gabapentinoid |
| 51891978 | Pregabalin 25mg capsules | gabapentinoid |
| 55712978 | Pregabalin 25mg capsules | gabapentinoid |
| 87401998 | Pregabalin 25mg capsules | gabapentinoid |
| 87408998 | Pregabalin 25mg capsules | gabapentinoid |
| 89078979 | Pregabalin 25mg capsules | gabapentinoid |
| 8691978 | Pregabalin 25mg tablets | gabapentinoid |
| 63277979 | Pregabalin 25mg/5ml oral solution | gabapentinoid |
| 63275979 | Pregabalin 25mg/5ml oral suspension | gabapentinoid |
| 28540978 | Pregabalin 300mg capsules | gabapentinoid |
| 42522978 | Pregabalin 300mg capsules | gabapentinoid |
| 47120978 | Pregabalin 300mg capsules | gabapentinoid |
| 51886978 | Pregabalin 300mg capsules | gabapentinoid |
| 51887978 | Pregabalin 300mg capsules | gabapentinoid |
| 51888978 | Pregabalin 300mg capsules | gabapentinoid |
| 55711978 | Pregabalin 300mg capsules | gabapentinoid |
| 87395998 | Pregabalin 300mg capsules | gabapentinoid |
| 87402998 | Pregabalin 300mg capsules | gabapentinoid |
| 89070979 | Pregabalin 300mg capsules | gabapentinoid |
| 8687978 | Pregabalin 300mg tablets | gabapentinoid |
| 21844978 | Pregabalin 300mg/5ml oral suspension | gabapentinoid |
| 28539978 | Pregabalin 50mg capsules | gabapentinoid |
| 51883978 | Pregabalin 50mg capsules | gabapentinoid |
| 51885978 | Pregabalin 50mg capsules | gabapentinoid |
| 55710978 | Pregabalin 50mg capsules | gabapentinoid |
| 87400998 | Pregabalin 50mg capsules | gabapentinoid |
| 87407998 | Pregabalin 50mg capsules | gabapentinoid |
| 89063979 | Pregabalin 50mg capsules | gabapentinoid |
| 8939978 | Pregabalin 50mg tablets | gabapentinoid |
| 30141978 | Pregabalin 50mg/5ml oral suspension | gabapentinoid |
| 28538978 | Pregabalin 75mg capsules | gabapentinoid |
| 51880978 | Pregabalin 75mg capsules | gabapentinoid |
| 51882978 | Pregabalin 75mg capsules | gabapentinoid |
| 55709978 | Pregabalin 75mg capsules | gabapentinoid |
| 87399998 | Pregabalin 75mg capsules | gabapentinoid |
| 87406998 | Pregabalin 75mg capsules | gabapentinoid |
| 89056979 | Pregabalin 75mg capsules | gabapentinoid |
| 89058979 | Pregabalin 75mg capsules | gabapentinoid |
| 89060979 | Pregabalin 75mg capsules | gabapentinoid |
| 8937978 | Pregabalin 75mg tablets | gabapentinoid |
| 64665979 | Pregabalin 75mg/5ml oral solution | gabapentinoid |
| 64663979 | Pregabalin 75mg/5ml oral suspension | gabapentinoid |
| 85080998 | Tramadol (roi) tablet | weak_opioid |
| 85081998 | Tramadol (roi) tablet | weak_opioid |
| 91733997 | Tramadol 100mg effervescent powder sachets sugar free | weak_opioid |
| 83127998 | Tramadol 100mg modified-release capsules | weak_opioid |
| 84342978 | Tramadol 100mg modified-release capsules | weak_opioid |
| 84541998 | Tramadol 100mg modified-release capsules | weak_opioid |
| 88547997 | Tramadol 100mg modified-release capsules | weak_opioid |
| 91153997 | Tramadol 100mg modified-release capsules | weak_opioid |
| 92370990 | Tramadol 100mg modified-release capsules | weak_opioid |
| 95470979 | Tramadol 100mg modified-release capsules | weak_opioid |
| 95472979 | Tramadol 100mg modified-release capsules | weak_opioid |
| 23073978 | Tramadol 100mg modified-release tablets | weak_opioid |
| 52655979 | Tramadol 100mg modified-release tablets | weak_opioid |
| 53206979 | Tramadol 100mg modified-release tablets | weak_opioid |
| 73249978 | Tramadol 100mg modified-release tablets | weak_opioid |
| 76932978 | Tramadol 100mg modified-release tablets | weak_opioid |
| 76933978 | Tramadol 100mg modified-release tablets | weak_opioid |
| 82792978 | Tramadol 100mg modified-release tablets | weak_opioid |
| 83069998 | Tramadol 100mg modified-release tablets | weak_opioid |
| 83259998 | Tramadol 100mg modified-release tablets | weak_opioid |
| 84237998 | Tramadol 100mg modified-release tablets | weak_opioid |
| 84265998 | Tramadol 100mg modified-release tablets | weak_opioid |
| 84353978 | Tramadol 100mg modified-release tablets | weak_opioid |
| 85306998 | Tramadol 100mg modified-release tablets | weak_opioid |
| 85307998 | Tramadol 100mg modified-release tablets | weak_opioid |
| 86100998 | Tramadol 100mg modified-release tablets | weak_opioid |
| 86928998 | Tramadol 100mg modified-release tablets | weak_opioid |
| 91158998 | Tramadol 100mg modified-release tablets | weak_opioid |
| 91159998 | Tramadol 100mg modified-release tablets | weak_opioid |
| 92468997 | Tramadol 100mg modified-release tablets | weak_opioid |
| 94283990 | Tramadol 100mg modified-release tablets | weak_opioid |
| 95497979 | Tramadol 100mg modified-release tablets | weak_opioid |
| 95502979 | Tramadol 100mg modified-release tablets | weak_opioid |
| 95541990 | Tramadol 100mg modified-release tablets | weak_opioid |
| 89680997 | Tramadol 100mg/2ml solution for injection ampoules | weak_opioid |
| 92676997 | Tramadol 100mg/2ml solution for injection ampoules | weak_opioid |
| 92677997 | Tramadol 100mg/2ml solution for injection ampoules | weak_opioid |
| 81303998 | Tramadol 100mg/ml oral drops | weak_opioid |
| 83126998 | Tramadol 150mg modified-release capsules | weak_opioid |
| 84540998 | Tramadol 150mg modified-release capsules | weak_opioid |
| 88547996 | Tramadol 150mg modified-release capsules | weak_opioid |
| 91153996 | Tramadol 150mg modified-release capsules | weak_opioid |
| 92381990 | Tramadol 150mg modified-release capsules | weak_opioid |
| 95463979 | Tramadol 150mg modified-release capsules | weak_opioid |
| 95464979 | Tramadol 150mg modified-release capsules | weak_opioid |
| 95465979 | Tramadol 150mg modified-release capsules | weak_opioid |
| 23072978 | Tramadol 150mg modified-release tablets | weak_opioid |
| 52653979 | Tramadol 150mg modified-release tablets | weak_opioid |
| 58617979 | Tramadol 150mg modified-release tablets | weak_opioid |
| 83258998 | Tramadol 150mg modified-release tablets | weak_opioid |
| 84236998 | Tramadol 150mg modified-release tablets | weak_opioid |
| 84264998 | Tramadol 150mg modified-release tablets | weak_opioid |
| 84351978 | Tramadol 150mg modified-release tablets | weak_opioid |
| 86073998 | Tramadol 150mg modified-release tablets | weak_opioid |
| 86097998 | Tramadol 150mg modified-release tablets | weak_opioid |
| 86574998 | Tramadol 150mg modified-release tablets | weak_opioid |
| 86927998 | Tramadol 150mg modified-release tablets | weak_opioid |
| 89615998 | Tramadol 150mg modified-release tablets | weak_opioid |
| 90086998 | Tramadol 150mg modified-release tablets | weak_opioid |
| 91158997 | Tramadol 150mg modified-release tablets | weak_opioid |
| 91159997 | Tramadol 150mg modified-release tablets | weak_opioid |
| 91966998 | Tramadol 150mg modified-release tablets | weak_opioid |
| 92468996 | Tramadol 150mg modified-release tablets | weak_opioid |
| 94282990 | Tramadol 150mg modified-release tablets | weak_opioid |
| 95487979 | Tramadol 150mg modified-release tablets | weak_opioid |
| 95489979 | Tramadol 150mg modified-release tablets | weak_opioid |
| 95492979 | Tramadol 150mg modified-release tablets | weak_opioid |
| 95496979 | Tramadol 150mg modified-release tablets | weak_opioid |
| 95540990 | Tramadol 150mg modified-release tablets | weak_opioid |
| 76920978 | Tramadol 200mg modified-release capsules | weak_opioid |
| 83125998 | Tramadol 200mg modified-release capsules | weak_opioid |
| 84539998 | Tramadol 200mg modified-release capsules | weak_opioid |
| 88546998 | Tramadol 200mg modified-release capsules | weak_opioid |
| 91150998 | Tramadol 200mg modified-release capsules | weak_opioid |
| 92380990 | Tramadol 200mg modified-release capsules | weak_opioid |
| 95458979 | Tramadol 200mg modified-release capsules | weak_opioid |
| 95460979 | Tramadol 200mg modified-release capsules | weak_opioid |
| 23071978 | Tramadol 200mg modified-release tablets | weak_opioid |
| 53204979 | Tramadol 200mg modified-release tablets | weak_opioid |
| 73247978 | Tramadol 200mg modified-release tablets | weak_opioid |
| 83257998 | Tramadol 200mg modified-release tablets | weak_opioid |
| 84235998 | Tramadol 200mg modified-release tablets | weak_opioid |
| 84263998 | Tramadol 200mg modified-release tablets | weak_opioid |
| 84348978 | Tramadol 200mg modified-release tablets | weak_opioid |
| 85305998 | Tramadol 200mg modified-release tablets | weak_opioid |
| 86072998 | Tramadol 200mg modified-release tablets | weak_opioid |
| 86096998 | Tramadol 200mg modified-release tablets | weak_opioid |
| 86573998 | Tramadol 200mg modified-release tablets | weak_opioid |
| 86926998 | Tramadol 200mg modified-release tablets | weak_opioid |
| 89615997 | Tramadol 200mg modified-release tablets | weak_opioid |
| 90086997 | Tramadol 200mg modified-release tablets | weak_opioid |
| 91158996 | Tramadol 200mg modified-release tablets | weak_opioid |
| 91159996 | Tramadol 200mg modified-release tablets | weak_opioid |
| 91966997 | Tramadol 200mg modified-release tablets | weak_opioid |
| 92467998 | Tramadol 200mg modified-release tablets | weak_opioid |
| 94281990 | Tramadol 200mg modified-release tablets | weak_opioid |
| 95477979 | Tramadol 200mg modified-release tablets | weak_opioid |
| 95479979 | Tramadol 200mg modified-release tablets | weak_opioid |
| 95480979 | Tramadol 200mg modified-release tablets | weak_opioid |
| 95481979 | Tramadol 200mg modified-release tablets | weak_opioid |
| 95483979 | Tramadol 200mg modified-release tablets | weak_opioid |
| 95486979 | Tramadol 200mg modified-release tablets | weak_opioid |
| 95539990 | Tramadol 200mg modified-release tablets | weak_opioid |
| 85304998 | Tramadol 300mg modified-release tablets | weak_opioid |
| 86572998 | Tramadol 300mg modified-release tablets | weak_opioid |
| 89615996 | Tramadol 300mg modified-release tablets | weak_opioid |
| 90086996 | Tramadol 300mg modified-release tablets | weak_opioid |
| 91966996 | Tramadol 300mg modified-release tablets | weak_opioid |
| 82625998 | Tramadol 37.5mg / Paracetamol 325mg effervescent tablets sugar free | weak_opioid |
| 82625998 | Tramadol 37.5mg / Paracetamol 325mg effervescent tablets sugar free | weak_opioid |
| 82402998 | Tramadol 37.5mg / Paracetamol 325mg tablets | weak_opioid |
| 87614998 | Tramadol 37.5mg / Paracetamol 325mg tablets | weak_opioid |
| 87615998 | Tramadol 37.5mg / Paracetamol 325mg tablets | weak_opioid |
| 87616998 | Tramadol 37.5mg / Paracetamol 325mg tablets | weak_opioid |
| 82402998 | Tramadol 37.5mg / Paracetamol 325mg tablets | weak_opioid |
| 87614998 | Tramadol 37.5mg / Paracetamol 325mg tablets | weak_opioid |
| 87615998 | Tramadol 37.5mg / Paracetamol 325mg tablets | weak_opioid |
| 87616998 | Tramadol 37.5mg / Paracetamol 325mg tablets | weak_opioid |
| 86571998 | Tramadol 400mg modified-release tablets | weak_opioid |
| 89614998 | Tramadol 400mg modified-release tablets | weak_opioid |
| 90085998 | Tramadol 400mg modified-release tablets | weak_opioid |
| 91969998 | Tramadol 400mg modified-release tablets | weak_opioid |
| 60185979 | Tramadol 50mg capsules | weak_opioid |
| 89680998 | Tramadol 50mg capsules | weak_opioid |
| 90124998 | Tramadol 50mg capsules | weak_opioid |
| 92676998 | Tramadol 50mg capsules | weak_opioid |
| 92677998 | Tramadol 50mg capsules | weak_opioid |
| 94660990 | Tramadol 50mg capsules | weak_opioid |
| 95368990 | Tramadol 50mg capsules | weak_opioid |
| 95507979 | Tramadol 50mg capsules | weak_opioid |
| 95509979 | Tramadol 50mg capsules | weak_opioid |
| 95511979 | Tramadol 50mg capsules | weak_opioid |
| 95513979 | Tramadol 50mg capsules | weak_opioid |
| 96601990 | Tramadol 50mg capsules | weak_opioid |
| 96723990 | Tramadol 50mg capsules | weak_opioid |
| 96858990 | Tramadol 50mg capsules | weak_opioid |
| 96949990 | Tramadol 50mg capsules | weak_opioid |
| 97031990 | Tramadol 50mg capsules | weak_opioid |
| 97099990 | Tramadol 50mg capsules | weak_opioid |
| 97713990 | Tramadol 50mg capsules | weak_opioid |
| 97695990 | Tramadol 50mg capsules | weak_opioid |
| 97863990 | Tramadol 50mg capsules | weak_opioid |
| 91733998 | Tramadol 50mg effervescent powder sachets sugar free | weak_opioid |
| 83128998 | Tramadol 50mg modified-release capsules | weak_opioid |
| 84542998 | Tramadol 50mg modified-release capsules | weak_opioid |
| 88547998 | Tramadol 50mg modified-release capsules | weak_opioid |
| 91153998 | Tramadol 50mg modified-release capsules | weak_opioid |
| 95475979 | Tramadol 50mg modified-release capsules | weak_opioid |
| 95476979 | Tramadol 50mg modified-release capsules | weak_opioid |
| 84470998 | Tramadol 50mg modified-release tablets | weak_opioid |
| 84471998 | Tramadol 50mg modified-release tablets | weak_opioid |
| 59214978 | Tramadol 50mg orodispersible tablets sugar free | weak_opioid |
| 87863998 | Tramadol 50mg orodispersible tablets sugar free | weak_opioid |
| 87864998 | Tramadol 50mg orodispersible tablets sugar free | weak_opioid |
| 89751998 | Tramadol 50mg soluble tablets sugar free | weak_opioid |
| 92677996 | Tramadol 50mg soluble tablets sugar free | weak_opioid |
| 53420978 | Tramadol 50mg/5ml oral suspension | weak_opioid |
| 23070978 | Tramadol 75mg modified-release tablets | weak_opioid |
| 91150997 | Tramadol 75mg modified-release tablets | weak_opioid |
| 92468998 | Tramadol 75mg modified-release tablets | weak_opioid |
| 89079997 | Tramadol hydrochloride 100mg/sachet powder | weak_opioid |
| 89079998 | Tramadol hydrochloride 50mg/sachet powder | weak_opioid |

# Section 3: Mental health and substance misuse survival analysis: sub-stratified exposures

**Supplementary Table S11**: hazard ratios by sub-stratified exposure for substance misuse and mental illness outcomes. Proportional hazards are adjusted for exposure to the other pain phenotypes and analgesic classes, as well as gender, body mass index, year of birth, smoking status, alcohol consumption, prior mental illness, and prior substance misuse.

| Outcome | Exposure | Number exposed | Unadjusted HR (95% CI) | Adjusted HR (95% CI) | p-value (corrected for 22 comparisons) |
| --- | --- | --- | --- | --- | --- |
| Substance misuse | Musculoskeletal | 2364 | 2·07 (1·61-2·65) | 1·28 (0·96-1·70) | 1·00 |
|  | Migraine | 56629 | 1·04 (0·97-1·12) | 0·99 (0·91-1·09) | 1·00 |
|  | CRPS/erythromelalgia | 138 | 3·55 (1·60-7·91) | 5·43 (2·39-12·33) | 0·0011 |
|  | Trigeminal autonomic cephalalgia | 2156 | 1·27 (0·92-1·75) | 1·17 (0·79-1·73) | 1·00 |
|  | Other headache | 110 | 0·99 (0·14-7·06) | -* | - |
|  | Primary bladder pain | 95 | -* | -* | - |
|  | Breast pain | 1293 | 0·68 (0·38-1·23) | 0·64 (0·32-1·29) | 1·00 |
|  | Trigeminal neuralgia | 436 | 1·92 (1·06-3·46) | 1·55 (0·69-3·45) | 1·00 |
|  | Psychogenic pain | 67 | 0·96 (0·13-6·79) | -* | - |
|  | Fibromyalgia | 1162 | 1·28 (0·83-1·99) | 0·81 (0·47-1·41) | 1·00 |
|  | Primary orofacial pain | 1815 | 1·26 (0·89-1·79) | 0·89 (0·55-1·43) | 1·00 |
|  | Neuropathic pain | 10006 | 1·29 (1·11-1·50) | 1·08 (0·91-1·29) | 1·00 |
|  | Unspecified chronic pain | 2989 | 1·71 (1·31-2·23) | 1·37 (1·00-1·88) | 1·00 |
|  | Chronic or recurrent abdominal pain | 32257 | 0·86 (0·77-0·95) | 0·87 (0·77-0·98) | 0·46 |
|  | Dysmenorrhoea | 37574 | 0·76 (0·69-0·85) | 1·02 (0·91-1·14) | 1·00 |
|  | Paracetamol | 13640 | 1·76 (1·57-1·98) | 1·36 (1·17-1·58) | 0·0017 |
|  | Weak NSAID | 8340 | 1·73 (1·50-2·00) | 1·11 (0·93-1·33) | 1·00 |
|  | Strong NSAID | 8150 | 1·69 (1·46-1·95) | 0·95 (0·79-1·15) | 1·00 |
|  | Weak opioid | 4239 | 5·30 (4·70-5·98) | 2·34 (1·98-2·76) | <0·0001 |
|  | Strong opioid | 511 | 4·23 (2·81-6·37) | 1·09 (0·61-1·94) | 1·00 |
|  | Antidepressant | 4593 | 2·40 (2·01-2·86) | 1·29 (1·02-1·62) | 0·74 |
|  | Gabapentinoid | 1359 | 2·98 (2·19-4·05) | 0·82 (0·54-1·24) | 1·00 |
| Mental illness | Musculoskeletal | 2364 | 1·90 (1·76-2·05) | 1·14 (1·04-1·24) | 0·077 |
|  | Migraine | 56629 | 1·75 (1·72-1·78) | 1·18 (1·16-1·20) | <0·0001 |
|  | CRPS/erythromelalgia | 138 | 1·93 (1·40-2·65) | 1·05 (0·73-1·51) | 1·00 |
|  | Trigeminal autonomic cephalalgia | 2156 | 1·68 (1·55-1·83) | 1·14 (1·04-1·25) | 0·11 |
|  | Other headache | 110 | 1·68 (1·09-2·57) | 0·69 (0·42-1·13) | 1·00 |
|  | Primary bladder pain | 95 | 2·10 (1·43-3·08) | 1·00 (0·65-1·54) | 1·00 |
|  | Breast pain | 1293 | 2·55 (2·32-2·80) | 1·22 (1·11-1·35) | 0·0019 |
|  | Trigeminal neuralgia | 436 | 1·95 (1·64-2·33) | 1·31 (1·09-1·59) | 0·11 |
|  | Psychogenic pain | 67 | 1·95 (1·29-2·97) | 0·93 (0·59-1·48) | 1·00 |
|  | Fibromyalgia | 1162 | 2·28 (2·06-2·51) | 1·18 (1·06-1·32) | 0·053 |
|  | Primary orofacial pain | 1815 | 1·68 (1·53-1·83) | 1·13 (1·02-1·25) | 0·38 |
|  | Neuropathic pain | 10006 | 1·94 (1·87-2·01) | 1·21 (1·17-1·27) | <0·0001 |
|  | Unspecified chronic pain | 2989 | 2·20 (2·05-2·35) | 1·18 (1·09-1·28) | 0·00092 |
|  | Chronic or recurrent abdominal pain | 32257 | 2·16 (2·11-2·20) | 1·31 (1·28-1·34) | <0·0001 |
|  | Dysmenorrhoea | 37574 | 2·07 (2·03-2·11) | 1·12 (1·09-1·14) | <0·0001 |
|  | Paracetamol | 13640 | 1·57 (1·51-1·63) | 1·08 (1·04-1·13) | 0·0062 |
|  | Weak NSAID | 8340 | 1·66 (1·59-1·73) | 1·06 (1·00-1·11) | 0·8 |
|  | Strong NSAID | 8150 | 1·76 (1·69-1·83) | 1·13 (1·08-1·19) | <0·0001 |
|  | Weak opioid | 4239 | 2·57 (2·44-2·70) | 1·18 (1·10-1·26) | <0·0001 |
|  | Strong opioid | 511 | 2·89 (2·49-3·36) | 0·89 (0·75-1·07) | 1·00 |
|  | Antidepressant | 4593 | 2·20 (2·09-2·33) | 1·20 (1·12-1·28) | <0·0001 |
|  | Gabapentinoid | 1359 | 2·53 (2·30-2·80) | 0·96 (0·85-1·09) | 1·00 |

*No outcome events recorded

HR = hazard ratio

# Section 4: Opioid prescription recurrent event analysis

**Supplementary Table S12**: rate ratios for opioid prescriptions with covariate data shown

| Variable | Stratum | Rate ratio; negative binomial model (95% CI) | P-value; negative binomial model | Rate ratio; LWYY model | P-value; LWYY model |
| --- | --- | --- | --- | --- | --- |
| Group | Unexposed | 1 (Ref) | NA | 1 (Ref) | NA |
|  | Diagnosis | 2·02 (1·95-2·10) | <0·0001 | 1·80 (1·68-1·92) | <0·0001 |
|  | Prescription | 8·16 (7·56-8·81) | <0·0001 | 5·90 (5·33-6·54) | <0·0001 |
|  | Both | 13·85 (12·62-15·20) | <0·0001 | 8·52 (7·76-9·36) | <0·0001 |
|  | Exposed total | 3·85 (3·74-3·96) | <0·0001 | 2·88 (2·74-3·03) | <0·0001 |
| Gender | Male | 1 (Ref) | NA | 1 (Ref) | NA |
|  | Female | 1·35 (1·31-1·39) | <0·0001 | 1·30 (1·22-1·39) | <0·0001 |
| Smoking status | Non-smoker | 1 (Ref) | NA | 1 (Ref) | NA |
|  | Ex-Smoker | 1·84 (1·76-1·93) | <0·0001 | 1·56 (1·43-1·71) | <0·0001 |
|  | Smoker | 2·19 (2·12-2·26) | <0·0001 | 1·86 (1·73-1·98) | <0·0001 |
|  | Unknown | 2·1 (2·02-2·18) | <0·0001 | 1·92 (1·78-2·07) | <0·0001 |
| Alcohol use status | Non-drinker | 1 (Ref) | NA | 1 (Ref) | NA |
|  | Ex-Drinker | 1·59 (1·4-1·8) | <0·0001 | 1·36 (1·11-1·67) | 0·0027 |
|  | Drinker | 0·87 (0·84-0·9) | <0·0001 | 0·81 (0·75-0·87) | <0·0001 |
|  | Unknown | 1·09 (1·04-1·14) | 0·00017 | 0·91 (0·83-1) | 0·043 |
| Townsend score quintile | 1 | 1 (Ref) | NA | 1 (Ref) | NA |
|  | 2 | 1·06 (0·97-1·15) | 0·20 | 1 (0·83-1·22) | 0·97 |
|  | 3 | 1·13 (1·05-1·23) | 0·0018 | 1·17 (0·98-1·39) | 0·075 |
|  | 4 | 1·51 (1·4-1·63) | <0·0001 | 1·41 (1·19-1·67) | <0·0001 |
|  | 5 | 1·72 (1·59-1·86) | <0·0001 | 1·66 (1·42-1·95) | <0·0001 |
|  | Unknown | 1·38 (1·3-1·47) | <0·0001 | 1·31 (1·14-1·51) | 0·00016 |
| Body mass index | rms::rcs(BMI)BMI | 0·97 (0·95-0·99) | 0·0015 | 0·97 (0·93-1·01) | 0·15 |
|  | rms::rcs(BMI)BMI' | 1·53 (1·15-2·04) | 0·0034 | 1·92 (1·08-3·43) | 0·027 |
|  | rms::rcs(BMI)BMI'' | 0·81 (0·32-2·04) | 0·66 | 0·24 (0·04-1·43) | 0·12 |
|  | rms::rcs(BMI)BMI''' | 0·37 (0·15-0·91) | 0·031 | 1·92 (0·38-9·69) | 0·43 |
| Year of birth |  | 0·94 (0·94-0·95) | <0·0001 | 1·01 (1·00-1·02) | 0·029 |
| Substance misuse | Absent | 1 (Ref) | NA | 1 (Ref) | NA |
|  | Present | 3·21 (2·94-3·51) | <0·0001 | 2·77 (2·45-3·12) | <0·0001 |
| Mental illness | Absent | 1 (Ref) | NA | 1 (Ref) | NA |
|  | Present | 2·69 (2·61-2·77) | <0·0001 | 2·40 (2·27-2·54) | <0·0001 |

**Supplementary Table S13**: rate ratios for opioid prescriptions, by sub-stratified exposure. Estimates are adjusted for exposure to the other pain phenotypes and analgesic classes, as well as gender, body mass index, year of birth, smoking status, alcohol consumption, prior mental illness, and prior substance misuse

| Exposure | Rate ratio; negative binomial model (95% CI) | P-value; negative binomial model (corrected for 22 comparisons) | Rate ratio; LWYY model (95% CI) | P-value; LWYY model (corrected for 22 comparisons) |
| --- | --- | --- | --- | --- |
| Migraine | 1·67 (1·60-1·75) | <0·0001 | 1·35 (1·25-1·46) | <0·0001 |
| CRPS/erythromelalgia | 7·19 (3·17-16·30) | <0·0001 | 1·95 (0·76-5·00) | 1·00 |
| Trigeminal autonomic cephalalgia | 1·72 (1·37-2·15) | <0·0001 | 1·31 (0·98-1·76) | 1·00 |
| Other headache | 3·93 (1·61-9·56) | 0·058 | 3·44 (1·51-7·87) | 0·074 |
| Primary bladder pain | 2·31 (0·86-6·20) | 1·00 | 0·86 (0·47-1·58) | 1·00 |
| Musculoskeletal | 4·17 (3·40-5·11) | <0·0001 | 1·57 (1·19-2·09) | 0·038 |
| Trigeminal neuralgia | 1·20 (0·73-1·95) | 1·00 | 1·13 (0·58-2·22) | 1·00 |
| Breast pain | 1·41 (1·08-1·84) | 0·25 | 1·17 (0·77-1·76) | 1·00 |
| Psychogenic pain | 2·98 (0·89-10·00) | 1·00 | 3·95 (0·97-16·00) | 1·00 |
| Fibromyalgia | 1·92 (1·45-2·54) | 0·00012 | 1·30 (0·98-1·72) | 1·00 |
| Primary orofacial pain | 1·41 (1·11-1·79) | 0·12 | 1·01 (0·74-1·37) | 1·00 |
| Neuropathic pain | 2·52 (2·28-2·79) | <0·0001 | 1·59 (1·37-1·85) | <0·0001 |
| Unspecified chronic pain | 3·14 (2·61-3·78) | <0·0001 | 2·10 (1·67-2·65) | <0·0001 |
| Chronic/recurrent abdominal | 1·56 (1·48-1·66) | <0·0001 | 1·25 (1·14-1·37) | <0·0001 |
| Dysmenorrhoea | 1·30 (1·24-1·38) | <0·0001 | 1·23 (1·13-1·35) | 0·00012 |
| Paracetamol | 1·56 (1·42-1·72) | <0·0001 | 1·27 (1·10-1·46) | 0·021 |
| Weak NSAID | 1·95 (1·73-2·19) | <0·0001 | 1·37 (1·16-1·62) | 0·0052 |
| Strong NSAID | 2·18 (1·94-2·46) | <0·0001 | 1·18 (1·00-1·38) | 0·97 |
| Weak opioid | 15·96 (13·62-18·71) | <0·0001 | 9·50 (8·23-10·97) | <0·0001 |
| Strong opioid | 11·71 (7·54-18·18) | <0·0001 | 2·96 (2·26-3·87) | <0·0001 |
| Antidepressant | 2·35 (2·02-2·74) | <0·0001 | 1·48 (1·22-1·81) | 0·0018 |
| Gabapentinoid | 2·47 (1·86-3·28) | <0·0001 | 0·97 (0·76-1·24) | 1·00 |

# Section 5: Exposure subgroup condition prevalence comparison

**Supplementary table S14:** Conditions over- and under-represented within the prescription-only exposure subgroup; main analysis, males, full results table

| Condition name | Count in prescription-exposed subgroup | Count in remainder of exposed group | Category | Period prevalence OR (99% CIs) | Uncorrected p-value | Corrected p-value <0.01 |
| --- | --- | --- | --- | --- | --- | --- |
| Intellectual disability | 469 | 236 | Mental Health Disorders | 5·98 (4·85-7·4) | <0·0001 | * |
| Epilepsy | 511 | 674 | Neurological conditions | 2·26 (1·93-2·64) | <0·0001 | * |
| Dermatitis (atopc/contact/other/... | 2422 | 7816 | Skin conditions | 0·87 (0·81-0·93) | <0·0001 | * |
| Peptic ulcer disease | 39 | 108 | Diseases of the Digestive System | 1·05 (0·62-1·7) | 0·79 |  |
| Gastro-oesophageal reflux disease | 389 | 1256 | Diseases of the Digestive System | 0·9 (0·77-1·04) | 0·06 |  |
| Fracture of wrist | 378 | 1156 | Musculoskeletal conditions | 0·95 (0·81-1·11) | 0·37 |  |
| Asthma | 2583 | 8128 | Diseases of the Respiratory System | 0·9 (0·84-0·96) | <0·0001 | * |
| Allergic and chronic rhinitis | 2201 | 7498 | Diseases of the Respiratory System | 0·81 (0·76-0·87) | <0·0001 | * |
| Anxiety disorders | 857 | 3419 | Mental Health Disorders | 0·7 (0·63-0·78) | <0·0001 | * |
| Depression | 1278 | 4055 | Mental Health Disorders | 0·9 (0·83-0·99) | 0·0028 |  |
| Hypo or hyperthyroidism | 83 | 148 | Diseases of the Endocrine System | 1·63 (1·13-2·34) | 0·00052 |  |
| Seborrheic dermatitis | 542 | 1442 | Skin conditions | 1·1 (0·96-1·25) | 0·078 |  |
| Sickle-cell trait | 9 | 16 | Haematological/Immunological conditions | 1·63 (0·47-5·02) | 0·25 |  |
| Other haemolytic anaemias | 9 | 19 | Haematological/Immunological conditions | 1·38 (0·41-4·03) | 0·43 |  |
| Abdominal Hernia | 457 | 1344 | Diseases of the Digestive System | 0·99 (0·85-1·14) | 0·81 |  |
| Volvulus | <5 | 6 | Diseases of the Digestive System | 0·97 (0·04-8·36) | 0·99 |  |
| Prematurity | 56 | 104 | Perinatal conditions | 1·57 (1-2·42) | 0·0084 |  |
| Neonatal jaundice (excl haemolyt... | 18 | 34 | Perinatal conditions | 1·54 (0·67-3·32) | 0·15 |  |
| Splenomegaly | 11 | 29 | Haematological/Immunological conditions | 1·1 (0·38-2·77) | 0·77 |  |
| Urticaria | 565 | 1863 | Skin conditions | 0·87 (0·77-0·99) | 0·0058 |  |
| Hearing loss | 729 | 1862 | Diseases of the Ear | 1·15 (1·02-1·29) | 0·0027 |  |
| Rheumatoid Arthritis | 75 | 21 | Musculoskeletal conditions | 10·43 (5·54-21·19) | <0·0001 | * |
| Gout | 65 | 73 | Musculoskeletal conditions | 2·59 (1·64-4·09) | <0·0001 | * |
| Alcohol Problems | 242 | 556 | Mental Health Disorders | 1·27 (1·03-1·55) | 0·0025 |  |
| Ulcerative colitis | 61 | 113 | Diseases of the Digestive System | 1·57 (1·02-2·38) | 0·0057 |  |
| Crohn's disease | 110 | 171 | Diseases of the Digestive System | 1·88 (1·35-2·59) | <0·0001 | * |
| Scleritis and episcleritis | 18 | 49 | Diseases of the Eye | 1·07 (0·48-2·18) | 0·8 |  |
| Tinnitus | 93 | 330 | Diseases of the Ear | 0·82 (0·59-1·11) | 0·082 |  |
| Autism and Asperger's syndrome | 186 | 171 | Mental Health Disorders | 3·19 (2·41-4·23) | <0·0001 | * |
| Glomerulonephritis | 30 | 62 | Diseases of the Genitourinary system | 1·41 (0·76-2·52) | 0·13 |  |
| Hypertension | 85 | 160 | Diseases of the Circulatory System | 1·55 (1·08-2·2) | 0·0016 |  |
| Urolithiasis | 111 | 235 | Diseases of the Genitourinary system | 1·37 (1·01-1·86) | 0·0069 |  |
| Gastritis and duodenitis | 382 | 1411 | Diseases of the Digestive System | 0·78 (0·67-0·91) | <0·0001 | * |
| Secondary Malignancy_Lung | <5 | <5 | Cancers | 1·45 (0-67·21) | 0·75 |  |
| Primary Malignancy_Other Organs | 5 | 15 | Cancers | 0·97 (0·18-3·69) | 0·98 |  |
| Anterior and Intermediate Uveitis | 75 | 93 | Diseases of the Eye | 2·35 (1·55-3·55) | <0·0001 | * |
| Congenital malformations of card... | 62 | 128 | Perinatal conditions | 1·41 (0·92-2·11) | 0·03 |  |
| Chronic sinusitis | 382 | 1524 | Diseases of the Respiratory System | 0·72 (0·62-0·83) | <0·0001 | * |
| Juvenile arthritis | 102 | 40 | Musculoskeletal conditions | 7·46 (4·6-12·51) | <0·0001 | * |
| Iron deficiency anaemia | 113 | 243 | Haematological/Immunological conditions | 1·35 (1-1·82) | 0·0093 |  |
| Cerebral Palsy | 144 | 64 | Neurological conditions | 6·6 (4·47-9·94) | <0·0001 | * |
| Urinary Incontinence | 104 | 144 | Diseases of the Genitourinary system | 2·11 (1·49-2·96) | <0·0001 | * |
| Undescended testicle | 215 | 428 | Diseases of the Genitourinary system | 1·47 (1·17-1·83) | <0·0001 | * |
| Psoriasis | 354 | 970 | Skin conditions | 1·06 (0·9-1·25) | 0·35 |  |
| Psoriatic arthropathy | 39 | 26 | Musculoskeletal conditions | 4·37 (2·22-8·82) | <0·0001 | * |
| Acne | 2332 | 7679 | Skin conditions | 0·85 (0·79-0·91) | <0·0001 | * |
| Hyposplenism | 20 | 18 | Haematological/Immunological conditions | 3·23 (1·32-7·99) | 0·00043 |  |
| Anal fissure | 178 | 662 | Diseases of the Digestive System | 0·78 (0·62-0·97) | 0·0025 |  |
| Hyperkinetic disorders | 169 | 331 | Mental Health Disorders | 1·49 (1·16-1·91) | <0·0001 | * |
| Oesophagitis and oesophageal ulcer | 192 | 619 | Diseases of the Digestive System | 0·9 (0·72-1·11) | 0·2 |  |
| Supraventricular tachycardia | 14 | 57 | Diseases of the Circulatory System | 0·71 (0·29-1·52) | 0·26 |  |
| Carpal tunnel syndrome | 29 | 74 | Musculoskeletal conditions | 1·14 (0·62-2·01) | 0·55 |  |
| Other psychoactive substance misuse | 530 | 989 | Mental Health Disorders | 1·58 (1·37-1·83) | <0·0001 | * |
| Ankylosing spondylitis | 86 | 53 | Musculoskeletal conditions | 4·74 (2·99-7·62) | <0·0001 | * |
| Osteoarthritis (excl spine) | 65 | 59 | Musculoskeletal conditions | 3·21 (1·99-5·21) | <0·0001 | * |
| Erectile dysfunction | 161 | 470 | Diseases of the Genitourinary system | 0·99 (0·78-1·26) | 0·96 |  |
| Other anaemias | 137 | 249 | Haematological/Immunological conditions | 1·6 (1·21-2·12) | <0·0001 | * |
| Cystic Fibrosis | 13 | 11 | Diseases of the Endocrine System | 3·43 (1·1-11·13) | 0·0033 |  |
| Portal hypertension | <5 | <5 | Diseases of the Digestive System | 2·18 (0·17-21·76) | 0·33 |  |
| Osteoporosis | 32 | 19 | Musculoskeletal conditions | 4·9 (2·27-11·12) | <0·0001 | * |
| Diabetes | 117 | 187 | Diseases of the Endocrine System | 1·82 (1·33-2·49) | <0·0001 | * |
| Pericardial effusion (noninflamm... | 5 | <5 | Diseases of the Circulatory System | 7·26 (0·74-181·13) | 0·016 |  |
| Intracranial hypertension | <5 | 5 | Neurological conditions | 1·74 (0·14-14·21) | 0·46 |  |
| Appendicitis | 496 | 1595 | Diseases of the Digestive System | 0·9 (0·78-1·03) | 0·04 |  |
| Liver fibrosis, sclerosis and ci... | 7 | 6 | Diseases of the Digestive System | 3·39 (0·68-18·1) | 0·034 |  |
| Nasal polyp | 47 | 137 | Diseases of the Respiratory System | 1 (0·62-1·54) | 0·99 |  |
| Spondylosis | 16 | 48 | Musculoskeletal conditions | 0·97 (0·42-2·04) | 0·93 |  |
| Heart failure | 20 | 16 | Diseases of the Circulatory System | 3·63 (1·45-9·35) | 0·00016 |  |
| Atrial fibrillation | 11 | 30 | Diseases of the Circulatory System | 1·06 (0·37-2·66) | 0·84 |  |
| Obesity | 287 | 754 | Diseases of the Endocrine System | 1·11 (0·92-1·33) | 0·15 |  |
| Rosacea | 208 | 558 | Skin conditions | 1·08 (0·87-1·34) | 0·33 |  |
| Intracerebral haemorrhage | 11 | <5 | Diseases of the Circulatory System | 15·99 (2·46-350·09) | <0·0001 | * |
| Visual impairment and blindness | 57 | 120 | Diseases of the Eye | 1·38 (0·89-2·1) | 0·049 |  |
| End stage renal disease | 25 | 13 | Diseases of the Genitourinary system | 5·59 (2·26-15·11) | <0·0001 | * |
| Vitiligo | 38 | 123 | Skin conditions | 0·9 (0·53-1·45) | 0·56 |  |
| Pilonidal cyst/sinus | 214 | 647 | Skin conditions | 0·96 (0·78-1·18) | 0·61 |  |
| Obsessive-compulsive disorder | 46 | 245 | Mental Health Disorders | 0·54 (0·35-0·82) | <0·0001 |  |
| Ptosis of eyelid | 25 | 63 | Diseases of the Eye | 1·15 (0·59-2·13) | 0·54 |  |
| Hidradenitis suppurativa | 28 | 54 | Skin conditions | 1·51 (0·79-2·78) | 0·085 |  |
| Respiratory distress of newborn | 23 | 35 | Perinatal conditions | 1·91 (0·9-3·91) | 0·02 |  |
| Primary Malignancy_Bone and arti... | 8 | 6 | Cancers | 3·87 (0·84-20·02) | 0·015 |  |
| Hydrocoele (incl infected) | 201 | 597 | Diseases of the Genitourinary system | 0·98 (0·78-1·21) | 0·78 |  |
| Folate deficiency anaemia | 20 | 42 | Haematological/Immunological conditions | 1·38 (0·64-2·82) | 0·24 |  |
| Haemangioma, any site | 65 | 194 | Benign Neoplasm/CIN | 0·97 (0·66-1·41) | 0·85 |  |
| Pneumothorax | 59 | 168 | Diseases of the Respiratory System | 1·02 (0·67-1·51) | 0·89 |  |
| Slow fetal growth or low birth w... | 10 | 27 | Perinatal conditions | 1·08 (0·35-2·82) | 0·83 |  |
| Benign neoplasm of colon, rectum... | 13 | 31 | Benign Neoplasm/CIN | 1·22 (0·46-2·89) | 0·55 |  |
| Hepatic failure | <5 | <5 | Diseases of the Digestive System | 4·35 (0·26-124·24) | 0·13 |  |
| Schizophrenia, schizotypal and d... | 101 | 167 | Mental Health Disorders | 1·76 (1·26-2·45) | <0·0001 | * |
| Postinfective and reactive arthr... | 41 | 29 | Musculoskeletal conditions | 4·12 (2·15-8·04) | <0·0001 | * |
| Sleep apnoea | 37 | 60 | Diseases of the Respiratory System | 1·79 (1·01-3·11) | 0·0067 |  |
| Coeliac disease | 18 | 42 | Diseases of the Digestive System | 1·24 (0·55-2·6) | 0·44 |  |
| Obstructive and reflux uropathy | 68 | 104 | Diseases of the Genitourinary system | 1·9 (1·25-2·87) | <0·0001 |  |
| Down's syndrome | 39 | 8 | Perinatal conditions | 14·19 (5·35-47·8) | <0·0001 | * |
| Neuromuscular dysfunction of bla... | 31 | 80 | Diseases of the Genitourinary system | 1·13 (0·62-1·95) | 0·57 |  |
| Subarachnoid haemorrhage | 9 | <5 | Diseases of the Circulatory System | 8·71 (1·53-92·73) | 0·00052 |  |
| Stable angina | <5 | <5 | Diseases of the Circulatory System | 5·81 (0·49-152·14) | 0·046 |  |
| Coronary heart disease not other... | <5 | <5 | Diseases of the Circulatory System | 1·45 (0·06-17·32) | 0·66 |  |
| Peritonitis | 18 | 43 | Diseases of the Digestive System | 1·22 (0·54-2·53) | 0·48 |  |
| Chronic viral hepatitis | 12 | 19 | Infectious Diseases | 1·83 (0·63-4·97) | 0·11 |  |
| Bipolar affective disorder and m... | 17 | 49 | Mental Health Disorders | 1·01 (0·45-2·09) | 0·96 |  |
| Alopecia areata | 46 | 86 | Skin conditions | 1·55 (0·94-2·51) | 0·018 |  |
| Cataract | 32 | 43 | Diseases of the Eye | 2·16 (1·14-4·04) | 0·0014 |  |
| Glaucoma | 9 | 19 | Diseases of the Eye | 1·38 (0·41-4·03) | 0·43 |  |
| Fracture of hip | 32 | 41 | Musculoskeletal conditions | 2·27 (1·19-4·27) | 0·00077 |  |
| Diaphragmatic hernia | 60 | 146 | Diseases of the Digestive System | 1·19 (0·79-1·78) | 0·25 |  |
| Patent ductus arteriosus | 16 | 14 | Perinatal conditions | 3·32 (1·21-9·34) | 0·0014 |  |
| Nonrheumatic aortic valve disorders | 13 | 43 | Diseases of the Circulatory System | 0·88 (0·34-1·98) | 0·7 |  |
| Scoliosis | 158 | 194 | Musculoskeletal conditions | 2·38 (1·79-3·16) | <0·0001 | * |
| Raynaud's syndrome | 53 | 150 | Diseases of the Circulatory System | 1·03 (0·66-1·55) | 0·86 |  |
| Autoimmune liver disease | 6 | 5 | Diseases of the Digestive System | 3·48 (0·59-22·46) | 0·047 |  |
| Cholangitis | <5 | 5 | Diseases of the Digestive System | 1·16 (0·05-11·4) | 0·83 |  |
| Primary Malignancy_Other Skin an... | <5 | 5 | Cancers | 1·16 (0·05-11·4) | 0·83 |  |
| Vitamin B12 deficiency anaemia | 20 | 52 | Haematological/Immunological conditions | 1·12 (0·53-2·21) | 0·67 |  |
| Venous thromboembolic disease (E... | 41 | 41 | Diseases of the Circulatory System | 2·91 (1·6-5·28) | <0·0001 | * |
| Bell's palsy | 44 | 142 | Neurological conditions | 0·9 (0·56-1·4) | 0·55 |  |
| COPD | <5 | 7 | Diseases of the Respiratory System | 0·83 (0·04-6·54) | 0·87 |  |
| Hypertrophic Cardiomyopathy | <5 | 6 | Diseases of the Circulatory System | 1·94 (0·24-12·31) | 0·32 |  |
| Benign neoplasm of brain and oth... | 7 | 19 | Benign Neoplasm/CIN | 1·07 (0·27-3·39) | 0·86 |  |
| Bacterial sepsis of newborn | <5 | 5 | Perinatal conditions | 1·16 (0·05-11·4) | 0·83 |  |
| Anorectal fistula | 17 | 68 | Diseases of the Digestive System | 0·73 (0·33-1·45) | 0·23 |  |
| Primary Malignancy_Brain, Other ... | 15 | 18 | Cancers | 2·42 (0·9-6·33) | 0·015 |  |
| Primary Malignancy_Malignant Mel... | 6 | 8 | Cancers | 2·18 (0·42-10·1) | 0·17 |  |
| Personality disorders | 61 | 203 | Mental Health Disorders | 0·87 (0·58-1·27) | 0·35 |  |
| Agranulocytosis | 25 | 65 | Haematological/Immunological conditions | 1·12 (0·57-2·06) | 0·63 |  |
| Essential tremor | 10 | 51 | Neurological conditions | 0·57 (0·2-1·36) | 0·092 |  |
| Tuberculosis | 25 | 51 | Infectious Diseases | 1·42 (0·72-2·7) | 0·15 |  |
| Diabetic ophthalmic complications | 25 | 42 | Diseases of the Eye | 1·73 (0·85-3·38) | 0·035 |  |
| Myocardial infarction | <5 | <5 | Diseases of the Circulatory System | 3·87 (0·39-49·62) | 0·091 |  |
| Barrett's oesophagus | 8 | 7 | Diseases of the Digestive System | 3·32 (0·75-15·4) | 0·025 |  |
| Keratitis | 36 | 71 | Diseases of the Eye | 1·47 (0·84-2·52) | 0·064 |  |
| Spina bifida | 26 | 48 | Perinatal conditions | 1·57 (0·8-2·99) | 0·069 |  |
| Other Cardiomyopathy | 8 | 17 | Diseases of the Circulatory System | 1·37 (0·37-4·27) | 0·47 |  |
| Pancreatitis | 30 | 42 | Diseases of the Digestive System | 2·08 (1·07-3·93) | 0·0031 |  |
| Cholelithiasis | 25 | 46 | Diseases of the Digestive System | 1·58 (0·79-3·04) | 0·073 |  |
| Right bundle branch block | <5 | 18 | Diseases of the Circulatory System | 0·64 (0·1-2·57) | 0·45 |  |
| Retinal detachments and breaks | 14 | 28 | Diseases of the Eye | 1·45 (0·56-3·45) | 0·26 |  |
| Bronchiectasis | 13 | 12 | Diseases of the Respiratory System | 3·15 (1·02-9·82) | 0·0054 |  |
| Aspiration pneumonitis | 19 | 14 | Diseases of the Respiratory System | 3·94 (1·51-10·76) | 0·00012 |  |
| Pleural effusion | 11 | 15 | Diseases of the Respiratory System | 2·13 (0·67-6·34) | 0·066 |  |
| Ventricular tachycardia | <5 | <5 | Diseases of the Circulatory System | 1·45 (0·06-17·32) | 0·66 |  |
| Macular degeneration | <5 | <5 | Diseases of the Eye | 2·9 (0·21-40·94) | 0·22 |  |
| Fibromatoses | 8 | 11 | Musculoskeletal conditions | 2·11 (0·53-7·7) | 0·12 |  |
| Male infertility | 17 | 61 | Diseases of the Genitourinary system | 0·81 (0·36-1·63) | 0·45 |  |
| Hypertrophy of nasal turbinates | 11 | 31 | Diseases of the Respiratory System | 1·03 (0·36-2·56) | 0·91 |  |
| Ischaemic stroke | 6 | 10 | Diseases of the Circulatory System | 1·74 (0·35-7·22) | 0·3 |  |
| Thrombophilia | 5 | 8 | Haematological/Immunological conditions | 1·81 (0·3-8·93) | 0·31 |  |
| Intrauterine hypoxia | 9 | 12 | Perinatal conditions | 2·18 (0·6-7·41) | 0·089 |  |
| Primary Malignancy_Testicular | 12 | 20 | Cancers | 1·74 (0·6-4·65) | 0·14 |  |
| Hyperparathyroidism | 7 | 6 | Diseases of the Endocrine System | 3·39 (0·68-18·1) | 0·034 |  |
| HIV | 7 | 17 | Infectious Diseases | 1·2 (0·3-3·9) | 0·68 |  |
| Female infertility | 10 | 42 | Diseases of the Genitourinary system | 0·69 (0·24-1·69) | 0·3 |  |
| Primary or Idiopathic Thrombocyt... | 11 | 36 | Haematological/Immunological conditions | 0·89 (0·32-2·15) | 0·75 |  |
| Multiple sclerosis | 13 | 17 | Neurological conditions | 2·22 (0·77-6·1) | 0·036 |  |
| Aplastic anaemias | 5 | <5 | Haematological/Immunological conditions | 3·63 (0·5-30·56) | 0·065 |  |
| Postcoital and contact bleeding | <5 | <5 | Diseases of the Genitourinary system | 0·97 (0-23·28) | 0·97 |  |
| Leukaemia | 20 | 17 | Cancers | 3·42 (1·38-8·62) | 0·00027 |  |
| Collapsed vertebra | 6 | 11 | Musculoskeletal conditions | 1·58 (0·33-6·3) | 0·37 |  |
| Delirium, not induced by alcohol... | 6 | 13 | Mental Health Disorders | 1·34 (0·29-5·01) | 0·55 |  |
| Pulmonary embolism | 16 | 24 | Diseases of the Circulatory System | 1·94 (0·77-4·62) | 0·047 |  |
| Pulmonary collapse (excl pneumot... | <5 | 8 | Diseases of the Respiratory System | 1·45 (0·19-7·75) | 0·54 |  |
| Non-Hodgkin Lymphoma | 9 | 14 | Cancers | 1·87 (0·53-6) | 0·16 |  |
| Sickle-cell anaemia | 14 | 10 | Haematological/Immunological conditions | 4·07 (1·3-13·61) | 0·00083 |  |
| Thalassaemia trait | 12 | 45 | Haematological/Immunological conditions | 0·77 (0·29-1·77) | 0·44 |  |
| Polycythaemia vera | <5 | 10 | Cancers | 1·16 (0·16-5·59) | 0·78 |  |
| Anorectal prolapse | 13 | 32 | Diseases of the Digestive System | 1·18 (0·45-2·78) | 0·61 |  |
| Hyperplasia of prostate | <5 | 12 | Diseases of the Genitourinary system | 0·48 (0·02-3·05) | 0·36 |  |
| Lupus erythematosus (local and s... | 8 | 6 | Musculoskeletal conditions | 3·87 (0·84-20·02) | 0·015 |  |
| Secondary or other Thrombocytopa... | 9 | 27 | Haematological/Immunological conditions | 0·97 (0·3-2·62) | 0·96 |  |
| Primary Malignancy_Kidney and Ur... | <5 | <5 | Cancers | Inf (0·01-Inf) | 0·26 |  |
| Lichen planus | 10 | 31 | Skin conditions | 0·94 (0·31-2·39) | 0·88 |  |
| Primary pulmonary hypertension | 6 | <5 | Diseases of the Circulatory System | 8·71 (1·01-208·69) | 0·0053 |  |
| Thalassaemia | 14 | 20 | Haematological/Immunological conditions | 2·03 (0·75-5·23) | 0·049 |  |
| Primary Malignancy_Oro-pharyngeal | <5 | <5 | Cancers | 2·9 (0·09-95·95) | 0·33 |  |
| Anorexia and bulimia nervosa | 11 | 32 | Mental Health Disorders | 1 (0·35-2·47) | 0·98 |  |
| Transient ischaemic attack | <5 | 11 | Diseases of the Circulatory System | 0·79 (0·08-4·16) | 0·76 |  |
| Parkinson's disease | <5 | <5 | Neurological conditions | Inf (0·6-Inf) | 0·017 |  |
| Nonrheumatic mitral valve disorders | 7 | 27 | Diseases of the Circulatory System | 0·75 (0·2-2·21) | 0·52 |  |
| Oesophageal varices | <5 | 6 | Diseases of the Digestive System | 0·48 (0-6·31) | 0·56 |  |
| Meniere disease | <5 | 8 | Diseases of the Ear | 1·09 (0·1-6·56) | 0·87 |  |
| Cholecystitis | 7 | 13 | Diseases of the Digestive System | 1·56 (0·37-5·55) | 0·35 |  |
| Atrioventricular block, first de… | <5 | <5 | Diseases of the Circulatory System | 1·45 (0-67·21) | 0·75 |  |
| Other interstitial pulmonary dis... | <5 | <5 | Diseases of the Respiratory System | 1·45 (0-67·21) | 0·75 |  |
| Motor neuron disease | <5 | <5 | Neurological conditions | 2·9 (0·01-1134·67) | 0·51 |  |
| Giant Cell arteritis | <5 | 5 | Musculoskeletal conditions | 0·58 (0-8·53) | 0·69 |  |
| Sarcoidosis | <5 | 11 | Haematological/Immunological conditions | 1·06 (0·15-4·89) | 0·9 |  |
| Myelodysplastic syndromes | <5 | <5 | Cancers | Inf (0·01-Inf) | 0·26 |  |
| Hodgkin Lymphoma | 6 | 12 | Cancers | 1·45 (0·3-5·58) | 0·46 |  |
| Immunodeficiencies | 6 | 11 | Haematological/Immunological conditions | 1·58 (0·33-6·3) | 0·37 |  |
| Secondary polycythaemia | <5 | 7 | Haematological/Immunological conditions | 1·24 (0·11-8·06) | 0·73 |  |
| Benign neoplasm of stomach and d... | <5 | <5 | Benign Neoplasm/CIN | 1·94 (0·07-32·14) | 0·49 |  |
| Diverticular disease of intestin... | <5 | 12 | Diseases of the Digestive System | 0·97 (0·14-4·34) | 0·99 |  |
| Spondylolisthesis | 6 | 34 | Musculoskeletal conditions | 0·51 (0·12-1·54) | 0·12 |  |
| Secondary Malignancy_Other organs | <5 | <5 | Cancers | 5·81 (0·13-1691·73) | 0·18 |  |
| Monoclonal gammopathy of undeter... | <5 | <5 | Cancers | Inf (0·01-Inf) | 0·26 |  |
| Alcoholic liver disease | <5 | <5 | Diseases of the Digestive System | 5·81 (0·13-1691·73) | 0·18 |  |
| Left bundle branch block | <5 | <5 | Diseases of the Circulatory System | 2·9 (0·01-1134·67) | 0·51 |  |
| Primary Malignancy_colorectal an... | <5 | <5 | Cancers | 2·9 (0·09-95·95) | 0·33 |  |
| Menorrhagia and polymenorrhoea | <5 | <5 | Diseases of the Genitourinary system | 1·45 (0-67·21) | 0·75 |  |
| Retinal vascular occlusions | <5 | 5 | Diseases of the Eye | 0·58 (0-8·53) | 0·69 |  |
| Multiple valve dz | <5 | <5 | Diseases of the Circulatory System | 5·81 (0·13-1691·73) | 0·18 |  |
| Systemic sclerosis | <5 | <5 | Musculoskeletal conditions | 0·97 (0-23·28) | 0·97 |  |
| Respiratory failure | 5 | <5 | Diseases of the Respiratory System | 3·63 (0·5-30·56) | 0·065 |  |
| Actinic keratosis | <5 | 7 | Skin conditions | 0·41 (0-4·98) | 0·45 |  |
| Rheumatic fever | <5 | 6 | Infectious Diseases | 1·94 (0·24-12·31) | 0·32 |  |
| Subdural haemato–a - nontraumatic | <5 | 5 | Diseases of the Circulatory System | 0·58 (0-8·53) | 0·69 |  |
| Female genital prolapse | <5 | <5 | Diseases of the Genitourinary system | 8·71 (0·36-2240·52) | 0·059 |  |
| Rheumatic valve dz | <5 | 5 | Diseases of the Circulatory System | 1·74 (0·14-14·21) | 0·46 |  |
| Tubulo-interstitial nephritis | <5 | <5 | Diseases of the Genitourinary system | 2·9 (0·01-1134·67) | 0·51 |  |
| Spinal stenosis | <5 | 8 | Musculoskeletal conditions | 0·73 (0·03-5·35) | 0·74 |  |
| Sjog’en's disease | <5 | <5 | Musculoskeletal conditions | 2·9 (0·01-1134·67) | 0·51 |  |
| Atrioventricular block, complete | <5 | <5 | Diseases of the Circulatory System | Inf (0·01-Inf) | 0·26 |  |
| Secondary pulmonary hypertension | <5 | <5 | Diseases of the Circulatory System | 2·9 (0·01-1134·67) | 0·51 |  |
| Posterior Uveitis | <5 | 10 | Diseases of the Eye | 0·58 (0·03-3·89) | 0·52 |  |
| Dementia | <5 | <5 | Mental Health Disorders | 0 (0-14·08) | 0·41 |  |
| Enteropathic arthropathy | <5 | <5 | Musculoskeletal conditions | 0 (0-14·08) | 0·41 |  |
| Fatty Liver | <5 | 11 | Diseases of the Digestive System | 0 (0-1·8) | 0·039 |  |
| Asbestosis | <5 | <5 | Diseases of the Respiratory System | 0 (0-38·14) | 0·55 |  |
| Myasthenia gravis | <5 | <5 | Neurological conditions | 0 (0-38·14) | 0·55 |  |
| Polymyalgia Rheumatica | <5 | <5 | Musculoskeletal conditions | 0 (0-569·43) | 0·74 |  |
| Dilated cardiomyopathy | <5 | <5 | Diseases of the Circulatory System | 0 (0-8·01) | 0·31 |  |
| Angiodysplasia of colon | <5 | <5 | Diseases of the Digestive System | 0 (0-569·43) | 0·74 |  |
| Primary Malignancy_Lung and trachea | <5 | <5 | Cancers | 0 (0-569·43) | 0·74 |  |
| Primary Malignancy_Oesophageal | <5 | <5 | Cancers | 0 (0-569·43) | 0·74 |  |
| Primary Malignancy_Stomach | <5 | <5 | Cancers | 0 (0-569·43) | 0·74 |  |
| Benign neoplasm and polyp of uterus | <5 | <5 | Benign Neoplasm/CIN | 0 (0-569·43) | 0·74 |  |
| Carcinoma in situ_cervical | <5 | <5 | Benign Neoplasm/CIN | 0 (0-569·43) | 0·74 |  |
| Primary Malignancy_Thyroid | <5 | <5 | Cancers | 0 (0-38·14) | 0·55 |  |

**Supplementary table S15:** Conditions over- and under-represented within the prescription-only exposure subgroup; main analysis, females, full results table

| Condition name | Count in prescription-exposed subgroup | Count in remainder of exposed group | Category | Period prevalence OR (99% CIs) | Uncorrected p-value | Corrected p-value <0.01 |
| --- | --- | --- | --- | --- | --- | --- |
| Bronchiectasis | 5 | 40 | Diseases of the Respiratory System | 1·26 (0·26-3·99) | 0·6 |  |
[truncated: 63,527 more chars]
